# Systematic Study of Heteroarene Stacking Using a Congeneric Set of Molecular Glues for Procaspase-6

**DOI:** 10.1021/acs.jmedchem.3c00590

**Published:** 2023-07-05

**Authors:** Takaya Togo, Linh Tram, Laura G. Denton, Xochina ElHilali-Pollard, Jun Gu, Jinglei Jiang, Chenglei Liu, Yan Zhao, Yanlong Zhao, Yinzhe Zheng, Yunping Zheng, Jingjing Yang, Panpan Fan, Michelle R. Arkin, Harri Härmä, Deqian Sun, Stacie S. Canan, Steven E. Wheeler, Adam R. Renslo

**Affiliations:** † Department of Pharmaceutical Chemistry, University of California, 600 16th Street, San Francisco, California 94143, United States; ‡ Department of Chemistry, University of Georgia, Athens, Georgia 30602, United States; § Departments of Chemistry and Biology, Viva Biotech, Pu Dong New Area, 201203 Shanghai, China; # Department of Chemistry, University of Turku, 20500 Turku, Finland; ⊥̂ Departments of Chemistry and Structural Biology, Elgia Therapeutics, La Jolla, California 92037, United States

## Abstract

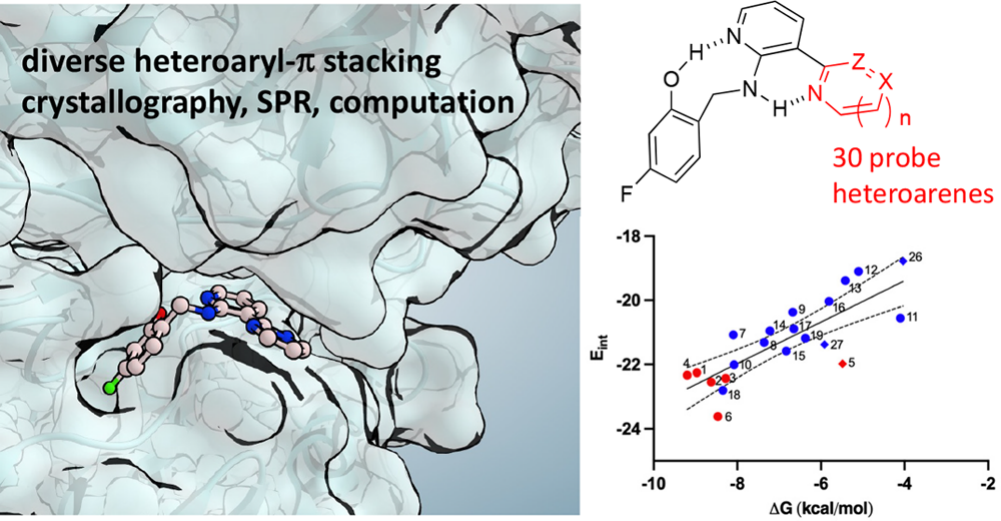

Heteroaromatic stacking interactions are important in drug binding, supramolecular chemistry, and materials science, making protein–ligand model systems of these interactions of considerable interest. Here we studied 30 congeneric ligands that each present a distinct heteroarene for stacking between tyrosine residues at the dimer interface of procaspase-6. Complex X-ray crystal structures of 10 analogs showed that stacking geometries were well conserved, while high-accuracy computations showed that heteroarene stacking energy was well correlated with predicted overall ligand binding energies. Empirically determined *K*_D_ values in this system thus provide a useful measure of heteroarene stacking with tyrosine. Stacking energies are discussed in the context of torsional strain, the number and positioning of heteroatoms, tautomeric state, and coaxial orientation of heteroarene in the stack. Overall, this study provides an extensive data set of empirical and high-level computed binding energies in a versatile new protein–ligand system amenable to studies of other intermolecular interactions.

## Introduction

Stacking interactions involving aromatic ring systems abound in chemistry and biology, playing key roles in the structure and stability of proteins, ligand binding in medicinal chemistry, supramolecular assemblies, and materials science. While there have been significant advances in our understanding of these interactions in recent decades from both experiment and theory, many questions remain about the factors controlling the strength and preferred geometries of stacked aromatic systems.

The empirical study of (hetero)aryl–aryl stacking in proteins and in ligand–protein interactions has largely been approached through bioinformatic analyses of large crystallographic databases.^1−3^ When taken in the aggregate, these data provide useful insights into preferred stacking orientations and geometries, despite sometimes confounding crystal packing effects. Such analyses are necessarily qualitative in nature, however, since interaction energies cannot be extracted from the purely structural information. Various computational studies have provided additional insight, from the early work of Hunter and Sanders^4^ to more recent studies exploring substituent effects^5−9^ and the effects of introducing heteroatoms^10−16^ (i.e., heteroarene–aryl stacking). Most recently, Wheeler and co-workers have introduced heterocycle descriptors derived from computed molecular electrostatic potentials (ESPs) that enable predictions of the maximum strength of diverse heteroarene stacking interactions without costly ab initio computations.^15−18^

**Figure 1:**
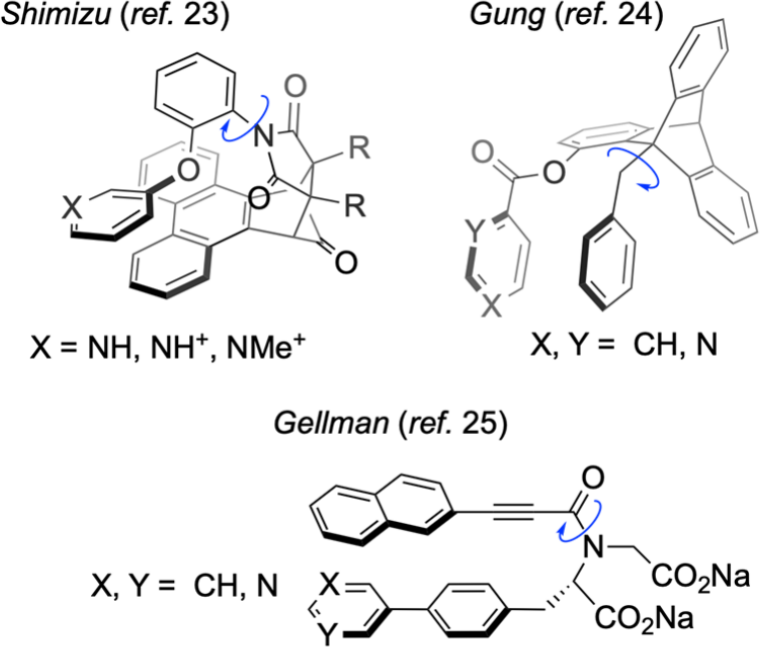
Examples of molecular torsion balances employed to study heteroarene–aryl stacking. Balances are shown in their folded conformations, with the relevant dihedral involved in equilibration to the open state indicated with a blue arrow.

Among experimental model systems used to study heteroarene–aryl stacking are synthetic host–guest systems^19^ and molecular “torsion balances”.^20−22^ Applications of the latter to heteroarene stacking include Shimizu’s cleft-like *N-*aryl imides,^23^ Gung’s triptycenes,^24^ and Gellman’s tertiary amide foldamers^25^ (Figure 1). In these model systems, conformational equilibrium between “closed” and “open” states, typically determined by NMR, is used to infer the binding enthalpy of the stacking interaction present in the closed state. A key advantage of torsion balances over host–guest systems is the ability to study solvent effects on an interaction of interest, as recently reviewed by Cockroft.^26^ On the other hand, the particular architectures of these single-molecule systems impose constraints on the orientation and approach of the interacting groups, such that stacking interactions may form with suboptimal interaction geometries and distances. Their often-challenging syntheses also limit applications to large numbers of diverse stacking interactions.

The use of a protein–ligand system to study intermolecular interactions is attractive in principle but also fraught with experimental pitfalls. Isolating the enthalpic contributions of an interaction of interest from global binding free energy is challenging in general and more so if one aims to study interacting groups with significantly different solvation energies. Ligand–protein systems were used successfully by Dougherty^27^ and Diederich^28^ to study cation−π interactions, where the geometric requirements of the interaction are less stringent. However, efforts by Diederich and co-workers^29,30^ to study heteroarene–amide stacking using factor Xa and cathepsin B as model systems were complicated by confounding effects on the distal ligand–protein interaction as the central heteroarene core was modified systematically.

Inspired by these seminal efforts, we have sought to identify new protein–ligand systems well suited for the empirical study of intermolecular interactions. In such systems, the protein component should adopt a shape-persistent binding site and be amenable to structure determination, while the ligand components should adopt a conserved binding mode, with the interacting heteroarene of interest displayed from a distal position. We recently described the use of the bacterial serine hydrolase CTX-M and 20 congeneric ligands to study heteroarene–amide backbone π stacking,^31^ providing the first experimental confirmation of a decade of computational work^19,32−34^ concerning this under-appreciated intermolecular interaction.

**Figure 2:**
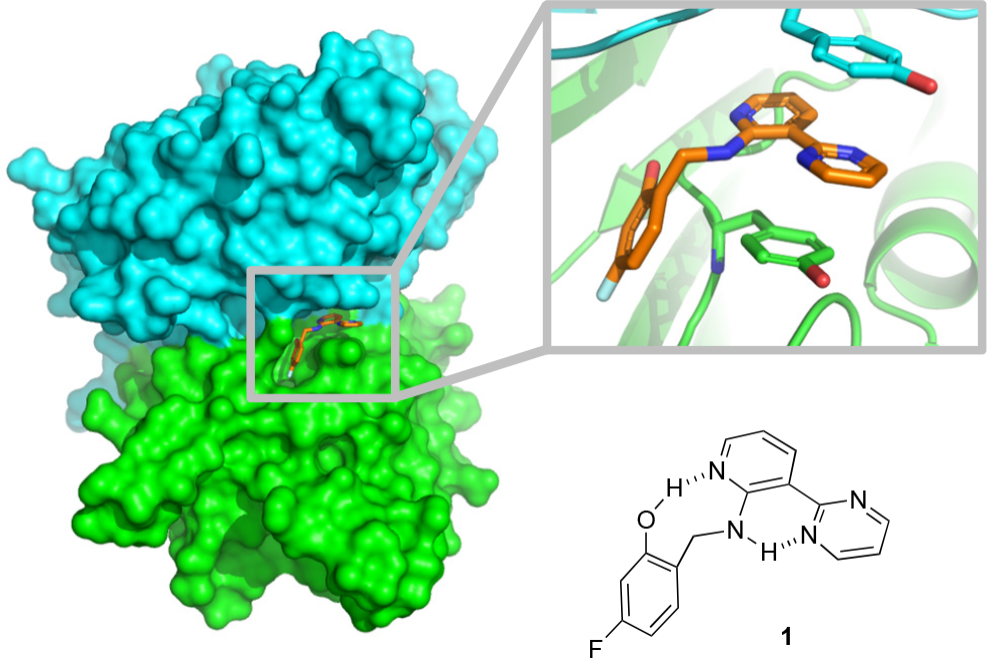
Structure and bound conformation of compound **1**, a molecular glue that binds the dimer interface of procaspase-6.^35^ The pyrimidine ring of **1** stacks between tyrosine 198^A^ and 198^B^ from the respective halves of the *C*_2_-symmetric dimer (PDB: 4NBL).

To better study heteroarene–aryl stacking interactions in solution, we focus here on compound **1**, a “molecular glue” that we previously found can stabilize the caspase-6 zymogen (proenzyme) toward proteolytic processing and activation, including in cells.^35,36^ Upon binding a site at the interface of the procaspase-6 dimer, the distal pyrimidine ring of **1** is presented for productive stacking between symmetry-related tyrosine residues 198^A^ and 198^B^, as revealed by X-ray crystallography (Figure 2).

**Chart 1:**
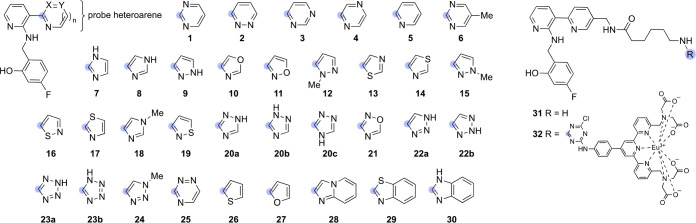
Compounds **1–30** Synthesized to Study Heteroarene–Aryl Stacking of Diverse Probe Heterocyclesa

To exploit this system for empirical studies of heteroarene–aryl stacking, we describe here the synthesis of 30 molecular glues based on **1**, each bearing a distinct five-membered, six-membered, or bicyclic “probe” heteroarene (Chart 1). We confirmed using X-ray crystallography that ligand binding is highly conserved across representative members of the test set. We determined empirical binding constants by SPR, and we evaluated these data in the context of high-accuracy computations of the same interactions. Overall, this study provides an exhaustive empirical data set of heteroarene–aryl stacking interactions and validates a powerful new model system for future studies of diverse intermolecular interactions under physiological conditions.

a The blue sphere indicates the site of connection to the shared ligand scaffold present in progenitor ligand **1**. Alternate tautomeric forms of **20**, **22**, and **23** are shown explicitly and were evaluated separately in the calculations. Analog **31** was used to prepare europium(III)-labeled probe **32** for time-resolved luminescence (TRL) studies of ligand binding in solution.

## Results and Discussion

**Scheme 1:**
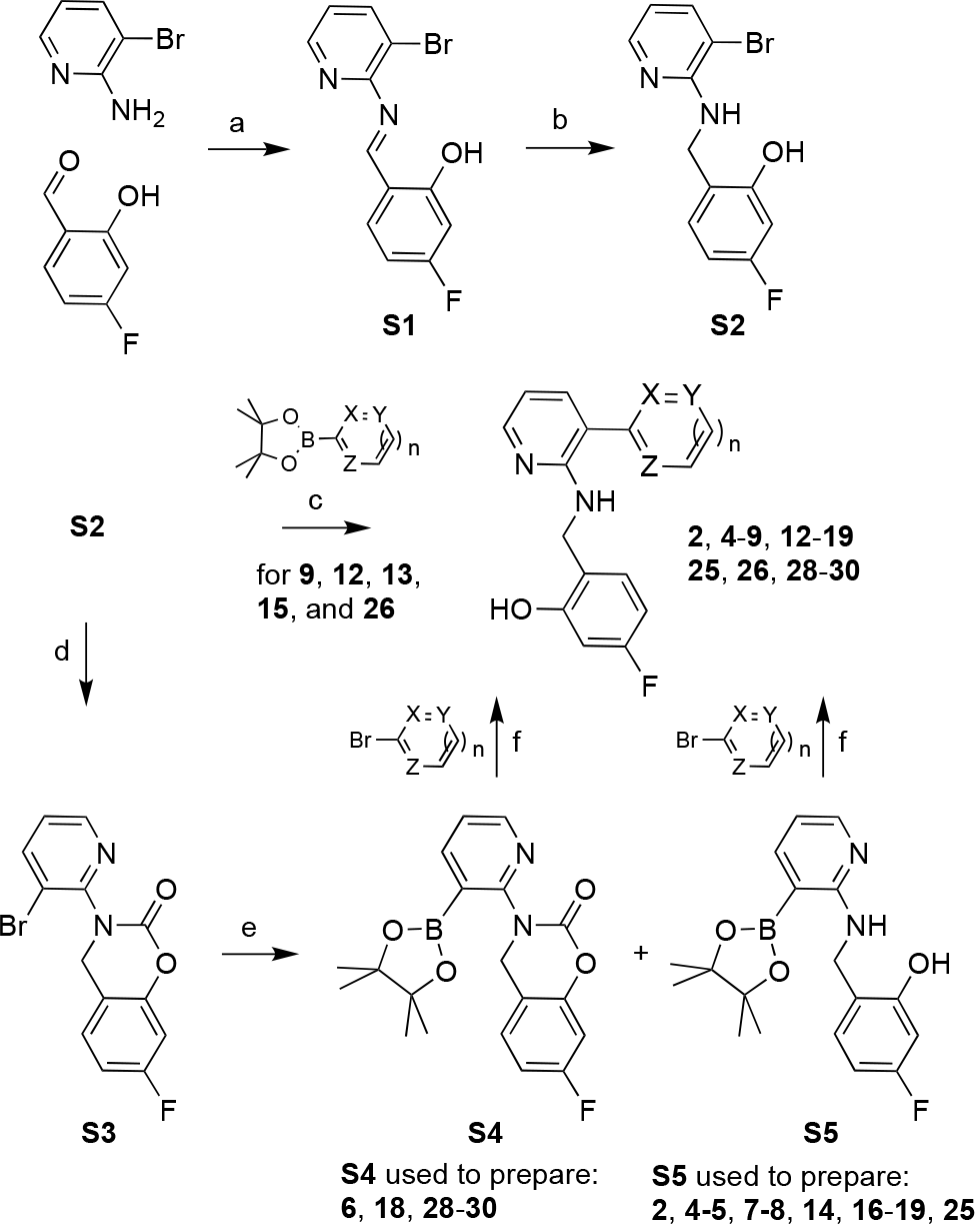
General Synthetic Approach to Compounds **2**, **4**–**9**, **12**–**20**, **25**, **26**, and **28**–**30** via Intermediates **S1**–**S5** and Based on Suzuki–Miyaura Coupling Reactions to Introduce the Probe Heteroarene Conditions: (a) (±)-camphor sulfonic acid, toluene, reflux; (b) NaBH_4_, THF, r.t.; (c) Pd(dppf)Cl_2_, K_2_CO_3_, dioxane, H_2_O; (d) triphosgene, CH_3_CN, 80 °C; (e) B_2_Pin_2_, Pd(dppf)Cl_2_, KOAc, dioxane, 85 °C; (f) Pd(dppf)Cl_2_, K_2_CO_3_, dioxane, H_2_O, 100 °C. Additional experimental details and synthetic schemes for all analogs are provided as Supporting Information.

A test set of 30 molecular glues was designed and synthesized wherein the pyrimidine ring of **1** was replaced with diverse heterocycles representing common ring systems from materials, supramolecular, and medicinal chemistry (**1–30**, Chart 1). The majority of the compounds could be prepared from common intermediates via late-stage Suzuki–Miyaura coupling reactions of heterocyclic bromides or boronate esters (Scheme 1 and Schemes S1–S9). A small number of analogs (**10**, **11**, **21**, **22**, and **27**) required bespoke synthetic routes to construct the desired heterocyclic ring system (see the Supporting Information).

Evident in the complex structure of **1** is an intramolecular hydrogen bond (IMHB) donated by the aminopyridine N–H and accepted by the proximal N atom of the pyrimidine ring (Figure 2). This IMHB promotes adoption of a favorable ligand conformation for π stacking, and so we retained a proximal N or other heteroatom at the analogous position in the various test ligands. The *N*-methyl pyrazole **12**, being unable to form the key IMHB, was prepared as a negative control to test the predicted importance of ligand preorganization for stacking.

**Figure 3:**
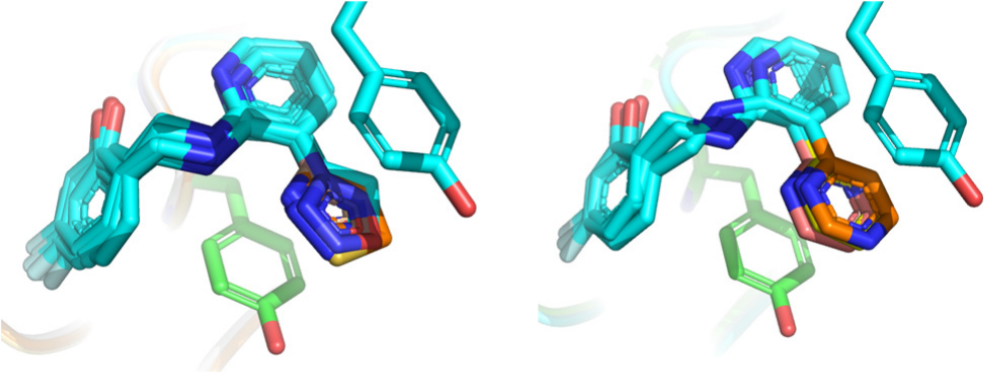
Left, aligned complex crystal structures of five-membered-ring analogs **7**, **8**, **10**, **11**, **19**, **20**, and **21** bound to procaspase-6. Right, aligned complex crystal structures of six-membered-ring analogs **1**, **3**, and **5** bound to procaspase-6. Probe heterocycles are colored differently from the shared ligand scaffold (cyan). Tyrosine side chains shown in cyan and green are from the procaspase-6 complex structures of **11** (left) and **3** (right). PDB IDs: 8F78 (**1**), 8F96 (**3**), 8F97 (**5**), 8FBV (**7**), 8F98 (**8**), 8F99 (**10**), 8F9A (**11**), 8F9B (**19**), 8F9C (**20**), and 8F9D (**21**).

To confirm that binding poses and stacking interactions were conserved across analogs **1**–**30**, we solved procaspase-6 complex crystal structures for 10 representative analogs spanning 3 orders of magnitude in measured *K*_D_ value (Table 1). Importantly, all 10 structures revealed binding poses almost exactly analogous to that adopted by **1**, with the probe heterocycles positioned in apparently productive stacking interactions with tyrosine 198^A^ and 198^B^ (Figure 3). A slightly different linker conformation was modeled into the density for one analog, but this did not noticeably impact the stacking interaction. These structures confirmed a shape-persistent binding site and a conserved ligand binding mode across diverse analogs.

**Table 1:** Experimentally Determined Procaspase-6 Binding Affinities of **1**–**30** Expressed as *K*_D_ (μM) and Δ*G* (kcal/mol) and Computed Binding Affinity Expressed as *E*_int_

Compd	*K*_D_^a^ (μM)	Δ*G* (kcal/mol)	*E*_int_		Compd	*K*_D_^a^ (μM)	Δ*G* (kcal/mol)	*E*_int_
**1**	0.27 ± 0.30	–8.96	–22.3		**16**	55.1 ± 20	–5.81	–20.0
**2**	0.47 ± 0.50	–8.62	–22.5		**17**	13.3 ± 2.5	–6.65	–20.9
**3**	0.85 ± 0.13	–8.28	–22.4		**18**	0.76 ± 0.19	–8.34	–22.8
**4**	0.18 ± 0.015	–9.20	–22.3		**19**	21.2 ± 1.2	–6.37	–21.2
**5**	95.3 ± 6.3	–5.48	–22.0		**20**	0.22 ± 0.041	–9.08	–20.5
**6**	0.62 ± 0.079	–8.47	–23.6		**21**	1.16 ± 0.80	–8.09	–20.9
**7**	1.15 ± 0.14	–8.10	–21.1		**22**	1.36 ± 0.29	–8.00	–20.7
**8**	4.01 ± 1.5	–7.36	–21.3		**23**	2.13 ± 0.11	–7.73	–21.0
**9**	12.8 ± 0.92	–6.67	–20.4		**24**	0.31 ± 0.052	–8.87	–22.6
**10**	1.19 ± 0.074	–8.08	–22.0		**25**	0.080 ± 0.012	–9.67	–22.3
**11**	980^†^	–4.10^†^	–20.6		**26**	1100^†^	–4.03^†^	–18.8
**12**	182^†^	–5.10^†^	–19.1		**27**	46.1 ± 11	–5.91	–21.4
**13**	106 ± 53	–5.42	–19.4		**28**	0.97 ± 0.22	–8.20	–24.7
**14**	5.08 ± 0.48	–7.22	–21.0		**29**	9.24 ± 3.0	–6.86	–23.9
**15**	9.79 ± 2.6	–6.83	–21.6		**30**	4.79 ± 1.2	–7.25	–23.7

a *K*_D_ values shown are the mean of three determinations ± SD, except as noted (†) for weak-binding analogs, where the *K*_D_ values are estimates.

**Figure 4:**
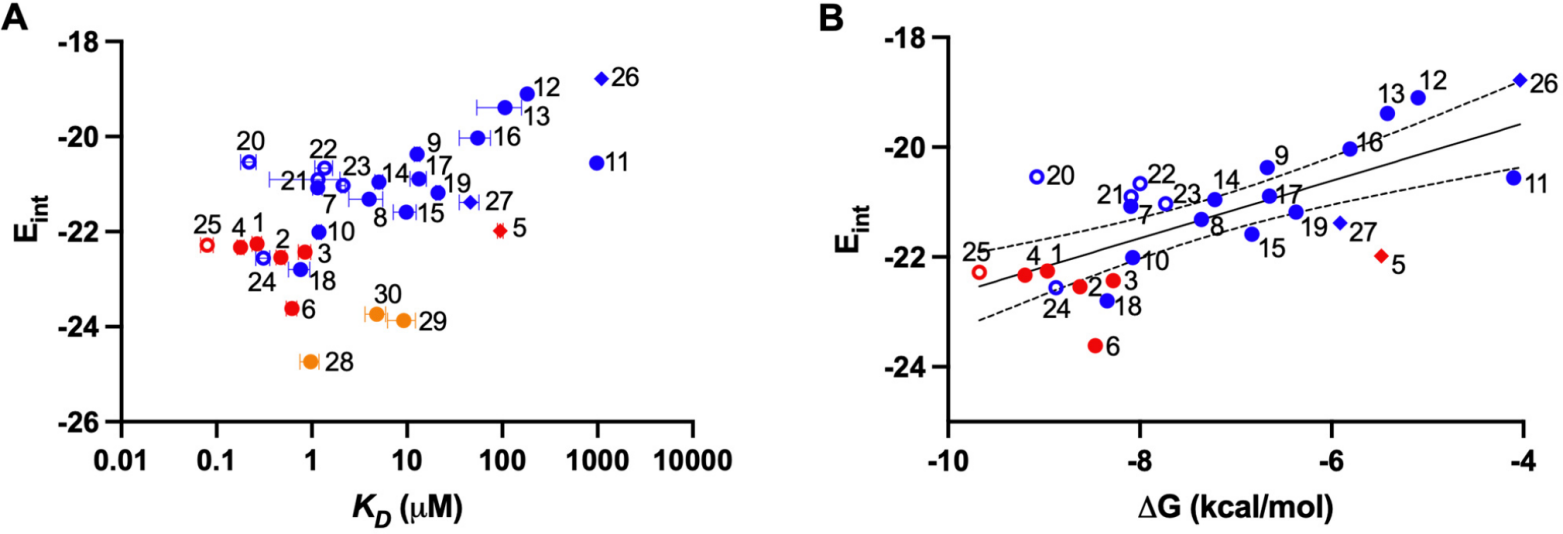
Computed interaction energies (*E*_int_) compared to experimental binding constants *K*_D_ (panel A) or the derived binding free energies Δ*G* (panel B). Analogs **28**–**30** bearing bicyclic heteroarenes were excluded from the linear regression analysis shown in panel B (*R*^2^ = 0.49; 95% confidence limits shown as dotted lines). Compound numbers are shown next to data points rendered in blue (five-membered heteroarenes), red (six-membered), or orange (bicyclic). Data symbols indicate the number of heteroatoms present in the probe heteroarene (diamonds = one; filled circles = two; open circles = three or four).

We next used SPR to assess the affinity of test ligands for a catalytically dead C163A procaspase-6 construct (preserving the zymogen fold). Initial sensorgrams were recorded with a 50 μM top concentration of the ligand to provide preliminary *K*_D_ estimates. Next, a definitive set of *K*_D_ values was determined in triplicate using an optimal concentration range for each ligand based on the preliminary *K*_D_ values. The average of the triplicate *K*_D_ values for each analog was furthermore used to calculate the experimental binding free energies Δ*G* reported in Table 1 and Figure 4.

For three weak-binding analogs (**11**, **12**, and **26**), *K*_D_ was estimated based on the SPR response measured at the highest concentration and the theoretical maximum response (*R*_max_) for the series. The poor *K*_D_ value for compound **12** was expected, since adoption of the preferred coplanar conformation that promotes π stacking cannot be stabilized by IMHB formation (*vide supra*). The likely reasons for the weak binding of analogs **11** and **26** are discussed later. Overall, the measured affinities spanned several orders of magnitude, affording a rich data set to analyze.

**Figure 5:**
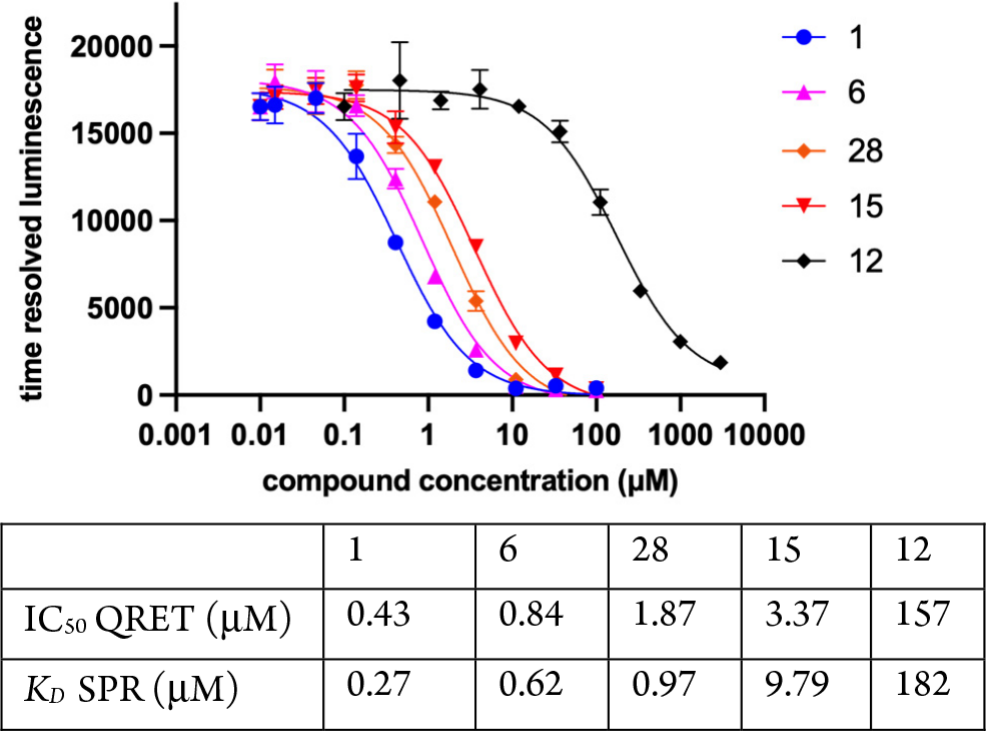
Dose–response curves (top) and IC_50_ values for procaspase-6 binding as determined using homogeneous time-resolved QRET with europium-labeled probe **32**. *K*_D_ values determined by SPR are provided for reference.

Binding assays using soluble target proteins lacking labels or surface immobilization arguably provide the most robust approach to the study of ligand–target interactions. Therefore, we further investigated the binding of selected analogs in solution using the single-label homogeneous quenching resonance energy transfer (QRET) assay format.^37,38^ To enable the QRET assay, we synthesized compound **31** bearing a short spacer to a primary amine end group for europium chelate conjugation to form probe **32** (Chart 1). In this assay, **32** binds to intact, unlabeled procaspase-6, leading to a high time-resolved luminescence (TRL) signal. Test compounds displace **32** and are quenched in solution with a signal modulator, and TRL is monitored. Using this approach, we measured IC_50_ values for **1**, **6**, **12**, **15**, and **28** that were in excellent agreement with the *K*_D_ values determined by SPR (Figure 5), lending further confidence in the robustness of the larger SPR data set.

**Figure 6:**
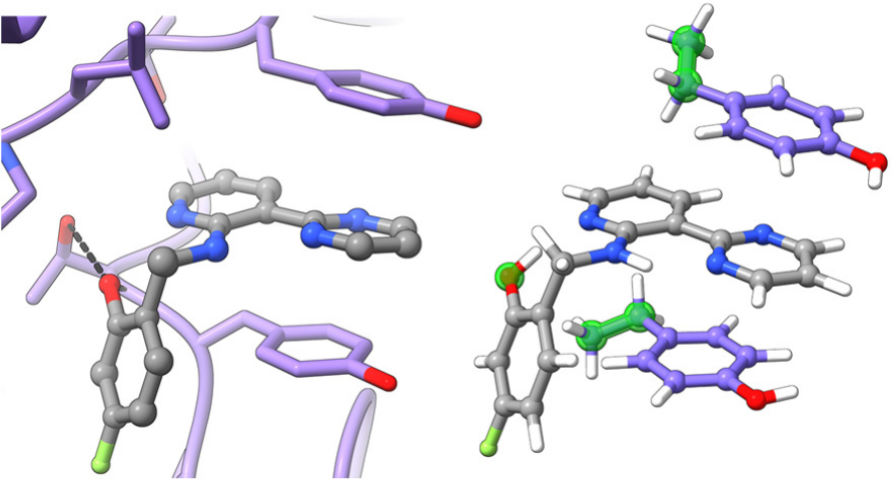
Left, reported crystal structure of compound **1** (PDB: 4NBL) and right, minimal biding model used for the DFT calculations described herein. The highlighted atoms are constrained during the DFT geometry optimizations.

To complement and better inform the interpretation of the experimental data, we performed computations using density functional theory (DFT) at the SMD(water)-wB97X-D/def2TZVP//SMD(water)-wB97X-D/def2SVP level of theory on a small model of the procaspase-6 binding site. This model (Figure 6) comprises the side chains of tyrosines 198^A^ and 198^B^ and the full ligand. Starting from the crystal structure reported^35^ for **1** (PDB: 4NBL), the probe heteroarene was modified as required, and the geometries were optimized with constraints on C_α_ and C_β_ of the Tyr side chains as well as the hydroxyl group of the ligand (to mimic the H-bond between this group and the protein backbone). We considered all four combinations of planar orientations of the Tyr hydroxyl group and both orientations of the heterocycle in the case of nonsymmetrical heterocycles. Interaction energies (*E*_int_) were calculated as the difference between the lowest-energy optimized structure for each ligand and the sum of the energies of the lowest-energy conformation of the ligand and two Tyr198 side chains in the optimized complex geometry. For ligands with multiple tautomeric states capable of IMHB formation (i.e., **20**, **22**, and **23**), *E*_int_ is calculated based on the lowest-energy tautomer in the bound and unbound states (*vide infra*).

The computed geometries indicated that nearly all ligands adopt the expected conformation in which the intramolecular NH–X bond is maintained and the probe heteroarene and pyridine ring are nearly coplanar. However, there are notable exceptions. For instance, the most favorable bound geometries for both thiophene (**26**) and furan (**27**) feature heteroarene orientations like that of **12**, in which a heteroarene C–H group is facing the pyridine N–H and, as a result, the heteroarene and pyridine rings are farther from coplanarity than in the other systems. The ligands with thiazole and isothiazole rings (**13** and **16**, respectively) also adopt conformations in which there is no apparent NH–X bond and the heterocycle is not coplanar with the pyridine. This behavior of **26**, **13**, and **16** in the computed geometries is consistent with current understanding^39^ of the heterocyclic sulfur atom as more analogous to an H-bond donor (“σ-hole” effect) than an H-bond acceptor.

Despite these differences, all of the ligands studied exhibited well-formed stacking interactions with tyrosine 198^A^ and 198^B^. However, formation of this stacking comes at some cost in terms of conformational strain. Not surprisingly, this is most extreme for **12**, for which there is a 20° reduction in the dihedral angle between the pyrazole and pyridine rings upon binding, leading to 2.9 kcal/mol of conformation strain (due primarily to a steric clash between the *N*-methyl of the pyrazole and C_4_–H on pyridine). For the other systems that lack the IMHB, the associated strain upon binding is predicted to be smaller (1–2 kcal/mol). For the ligands that maintain the IMHB, computations indicate much smaller and relatively constant strain energies of 0.5 ± 0.2 kcal/mol. Thus, while all measured binding affinities will reflect strain induced in the ligand upon binding, this effect is relatively constant for those ligands that feature the IMHB.

For ligands **20**, **22**, and **23** the triazole/tetrazole can adopt multiple tautomeric states while maintaining the intramolecular H-bond. We considered each of these tautomers for both the bound and unbound ligands. While for **22** and **23** the favored tautomer is the same in the bound and unbound states (**22b** and **23b**, respectively), for **20** the computations predict that the tautomeric state changes upon binding. That is, while **20c** is predicted to be the dominant tautomer in solution, **20b** is slightly favored in the bound state. This is an example of the favored tautomeric state changing purely through differences in the stacking interactions. Such behavior was previously predicted by An et al.^40^ for tetrazole stacking with 9-methyladenine based on model stacked dimers.

A comparison of computed *E*_int_ values and experimental ΔG values across all test ligands showed only a modest correlation (*R*^2^ = 0.33). A substantially improved correlation (*R*^2^ = 0.49; Figure 4B) was achieved by excluding the bicyclic analogs **28**–**30**, which when considered separately also showed an improved *R*^2^ value of 0.84 (Figure S2). Least well correlated were analogs bearing three or four ring heteroatoms, for reasons that are unclear. Excluding these analogs and analyzing the remaining 21 analogs bearing either one or two heteroatoms in five- or six-membered rings (i.e., **1**–**19**, **26**, and **27**) returned a quite reasonable *R*^2^ value of 0.64 (Figure S1).

To test our hypothesis that the experimental binding energies reflect relative differences in stacking energies, we performed computational analysis quantifying the stacking contribution to the total binding energy *E*_int_. This analysis indicated a strong correlation (*R*^2^ = 0.73) between heteroarene–tyrosine stacking energies [*E*_int_(Stack)] and total *E*_int_ across all ligands (Figure S3). Excluding **12**, for which *E*_int_ includes a substantial conformational strain penalty, the correlation improves further (*R*^2^ = 0.87). Accordingly, the computations support our expectation that experimental binding energies in this model system would reflect the relative strength of the stacking of heteroarenes with tyrosine side chains.

To provide further insight, we performed symmetry-adapted perturbation theory (SAPT) computations on the Tyr-Het-Tyr trimers.^41−43^ SAPT computations provide accurate interaction energies decomposed into contributions from electrostatics (Elec), dispersion (Disp), induction (Ind), and exchange-repulsion (Exch). These components are provided for all compounds in Table S5. In this case, the computed stacking energy [*E*(SAPT0)] is most strongly correlated with the electrostatic component (*R*^2^ = 0.86), in agreement with previous computational studies of model stacking interactions.^16^ To further quantify the importance of each component in determining trends in the total stacking energy, we also looked at the correlation of the sum of the other components to the total. For instance, “not *R*^2^ ” for Elec would be the correlation coefficient of (Exch + Ind + Disp) with *E*(SAPT0). From these values, we see that even though induction is modestly correlated with the total stacking energy (*R*^2^ = 0.52), the sum of the other three components is very strongly correlated with the total value (not *R*^2^ = 0.97). Thus, even though induction is modestly correlated with the total stacking energies, it contributes essentially nothing to the overall trend. On the other hand, eliminating either Elec or Disp completely eliminates any correlation with the total stacking energy (not *R*^2^ = 0.09 and 0.06, respectively), so these two components are vital contributors to the overall trend in stacking energies.

Finally, we sought to evaluate the experimental binding data using qualitative rules of thumb often employed by medicinal chemists when considering heteroarene stacking interactions. The significantly improved *K*_D_ values in the progression from pyridine (**5**) to isosteric diazenes (**1**–**4**) and finally to triazene **25**, for example, was fully consistent with guidelines from the Stahl^2^ and Wheeler^16^ groups that increasing numbers of heteroatoms lead to more favorable stacking with aromatic amino acid side chains. Also consistent with established guidance was the trend of increasing potencies from thiophene (**26**) to furan (**27**), to pyrazole (**9**) and imidazole (**7** and **8**) as well as the favorable effect of *N*-methylation of nitrogen-bearing heterocycles in the context of indazole (cf. **9** vs **15**), imidazole (cf. **8** vs **18**), and triazole (cf. **22** vs **24**). The latter observation suggests the utility of this model system for an expanded study of peripheral substituent effects in heteroarene stacking, an area of considerable recent interest in the computational arena.

**Figure 7:**
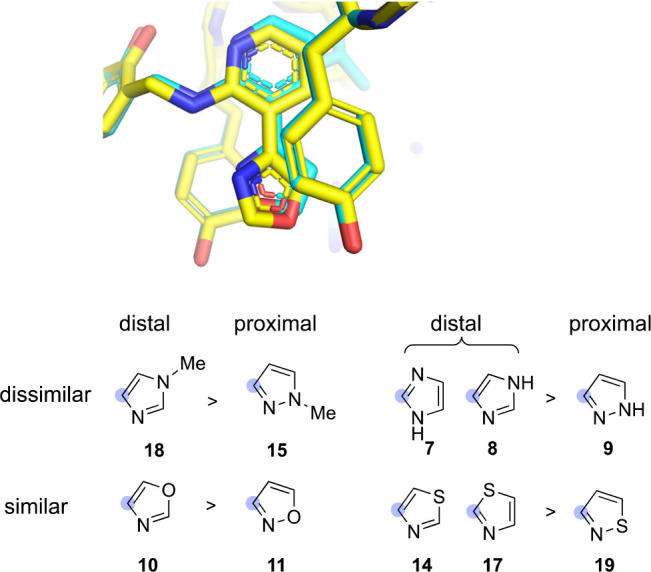
Alignment of the complex structures of **10** in yellow and **11** in cyan (top). Analogs with distal positioning of heteroatoms were, in all cases, more potent than comparators with proximal positioning of the same heteroatoms (bottom).

Recently, Bootsma et al.^16^ noted the importance in heteroarene stacking of the relative positioning of ring heteroatoms in otherwise similar heterocycles. The prediction, based on ESP descriptors, is that stacking will be enhanced in the case of proximal heteroatoms when the heteroatoms are “similar” (S, O, and imine-like N). By contrast, the distal arrangement is favored when the heteroatoms are “dissimilar” (i.e., one is an amine-like nitrogen atom). We evaluated these predictions across the four relevant comparator sets, confining the analysis to analogs bearing an ortho N atom capable of IMHB formation. In the case of rings with dissimilar heteroatoms, we observe distal positioning to be favored, as predicted (cf. **7** and **8** vs **9** and **18** vs **15**, Figure 7).

Interestingly, the analog pairs with “similar” heteroatoms also showed a preference for distal over proximal positioning of heteroatoms (cf. **14** and **17** vs **19** and **10** vs **11**, Figure 7), contrary to expectations. Most striking was the case of oxazole analog **10** and isoxazole **11**, with a difference in experimental binding energies of nearly 4 kcal/mol (!), despite a nearly identical binding mode in their complex crystal structures (Figure 7). While the dramatically enhanced binding of **10** compared to **11** was correctly predicted by DFT (*E*_int_ values), the trend was nonetheless contrary to the general guidelines from Bootsma et al. based on ESP descriptors.^16^ However, the descriptors and methods underlying these guidelines reflect the binding of an isolated heteroarene in which the heterocycle *is free to adopt any coaxial relationship* relative to the aromatic side chain. In the ligands considered here, various binding contacts of the larger ligand must be satisfied, and this severely constrains the number of coaxial orientations available to the heteroarene. Based on predicted^16^ coaxial preferences for oxazole and isoxazole stacking on tyrosine side chains, we conclude that stacking in **10** is more favorable because oxazole is more agnostic in its coaxial stacking orientation, while the isoxazole ring in **11** is constrained by the global ligand binding mode to adopt a locally nonoptimal orientation with heteroatoms placed in proximity to the tyrosine O–H bond (Figure 7).

## Conclusions

Here we employed a congeneric set of molecular glues for procaspase-6 to study diverse heteroarene stacking on tyrosine side chains under physiologically relevant conditions. As a model system, this protein–ligand interaction exhibits desirable features such as a shape-persistent binding site, a conserved ligand binding mode across diverse analogs, and the positioning of the probe heteroarene at the terminus of the ligand structure. The probe ligands are synthetically accessible and thus well suited to the study of diverse heteroarenes and also substitution effects only briefly explored here. Site-directed mutagenesis of the procaspase-6 protein construct at residue 198 should also enable future applications to the study of π stacking on other amino acid side chains and possibly also the study of cation−π and C–H−π interactions under physiological conditions. Noted limitations of this experimental construct include the restriction of heteroarene coaxial orientation which is enforced by global ligand binding. Accordingly, the findings of this study regarding distal and proximal heteroatom positioning in heteroarenes may not apply to stacking in other protein–ligand systems where coaxial stacking orientations are different. With these caveats, this study nevertheless provides what is perhaps the most extensive data set available of empirical and computed binding energies for a single protein–ligand system. The data and insights provided should be of use to those studying or optimizing stacking interactions generally and may also find utility in testing and improving computational approaches aimed at modeling these important and ubiquitous intermolecular interactions.

## Experimental Section

Reactions were magnetically stirred or microwave irradiated unless otherwise indicated. Air- and/or moisture-sensitive reactions were carried out under an argon atmosphere using anhydrous solvents from commercial suppliers. Air- and/or moisture-sensitive reagents were transferred via syringe or cannula and were introduced into reaction vessels through rubber septa. Reaction product solutions and chromatographic fractions were concentrated by rotary evaporation. Thin-phase chromatography was performed on an EMD precoated glass-backed silica gel 60 F-254 0.25 mm plate. All chemical reagents and solvents used were purchased from commercial sources, such as Sigma-Aldrich, TCI, Ambeed, or Fisher Scientific. Anhydrous DMF, dichloromethane, and tetrahydrofuran (EMD Drisolv) were used without further purification. Intermediates **S1** and **S2** and compound **1** were prepared by the reported procedures.^35^ The nonadentate europium chelate, europium(III) 2,2′,2″,2‴-(((4′-(4-((4,6-dichloro-1,3,5-triazin-2-yl)amino)phenyl)-[2,2′:6′,2″-terpyridine]-6,6″-diyl)bis(methylene))bis(azanetriyl))tetraacetate, and the soluble quencher molecule MT2 were obtained from QRET Technologies (Turku, Finland). ^1^H NMR spectra were recorded with Varian INOVA-400 or Bruker Biospin 400 MHz spectrometers. Chemical shifts are reported in δ units (ppm). NMR spectra were referenced relative to residual NMR solvent peaks. Coupling constants (*J*) are reported in hertz (Hz). Column chromatography was performed on Silicycle Sili-prep cartridges using a Biotage Isolera Four automated flash chromatography system. Compound purity and molecular weight were determined using a Waters Acquity UPLC/MS system, equipped with Waters ELSD and PDA detectors. Separations were carried out with an XTerra MS C18, 5 μm, 4.6 mm × 50 mm column, at ambient temperature (unregulated) using a mobile phase of water–methanol containing a constant 0.1% formic acid. All final compounds were ≥95% pure as determined by analytic UPLC.

### Synthesis of 3-(3-bromopyridin-2-yl)-7-fluoro-3,4-dihydro-2*H*-benzo[*e*][1,3]oxazin-2-one (**S3**)

To a stirred solution of 2-(((3-bromopyridin-2-yl)amino)methyl)-5-fluorophenol (**S2**) (10g, 33.6 mmol) in acetonitrile (15 mL) in a round-bottom flask was added triphosgene solution (50.4 mmol) portion-wise. The reaction mixture was heated to 80 °C for 1 h and monitored using thin-layer chromatography. Once the reaction was judged complete, 5% NaHCO_3_ aqueous solution (25 mL) was added, and the reaction mixture was cooled to 0 °C. The crude product **S3** (10.4 g, 96% crude) was obtained as a white solid after vacuum filtration. This material was used in the next step without further purification. ^1^H NMR (400 MHz, DMSO-*d*6) δ 8.58 (dd, *J* = 4.7, 1.6 Hz, 1H), 8.28 (dd, *J* = 8.0, 1.6 Hz, 1H), 7.46–7.38 (m, 2H), 7.18 (dd, *J* = 9.5, 2.5 Hz, 1H), 7.11 (td, *J* = 8.4, 2.4 Hz, 1H), 5.12 (d, *J* = 14.4 Hz, 1H), 4.70 (d, *J* = 14.5 Hz, 1H).

### Synthesis of 7-fluoro-3-(3-(4,4,5,5-tetramethyl-1,3,2-dioxaborolan-2-yl)pyridin-2-yl)-3,4-dihydro-2*H*-benzo[*e*][1,3]oxazin-2-one (**S4**)

A dried round-bottom under argon was charged with 3-(3-bromopyridin-2-yl)-7-fluoro-3,4-dihydro-2*H*-benzo[*e*][1,3]oxazin-2-one (**S3**) (6.54 g, 20.2 mmol), B_2_Pin_2_ (10.3 g, 40.5 mmol), Pd(dppf)Cl_2_ (1.48 g, 2.0 mmol), and KOAc (5.96 g, 60.7 mmol) and fitted with a reflux condensor and a balloon filled with Ar(g). The mixture was dissolved in anhydrous dioxane (50 mL) and heated with stirring overnight at 85 °C. The crude reaction was then filtered over a pad of silica and celite, the filtrated concentrated *in vacuo*, and the crude product purified by silica gel chromatography (EtOAc in hexane = 0–40%) to provide **S4** as a yellow solid (3.6 g, 48%). ^1^H NMR (400 MHz, CDCl_3_) δ 8.54 (dd, *J* = 4.9, 2.0 Hz, 1H), 8.14 (dd, *J* = 7.3, 2.1 Hz, 1H), 7.26 (m, 1H), 7.22–7.17 (m, 1H), 6.96–6.87 (m, 2H), 5.04 (s, 2H), 1.30 (s, 12H). ^1^H NMR (400 MHz, DMSO-*d*_6_) δ 8.58 (dd, 1H), 7.99 (dd, 1H), 7.44 (dd, 1H), 7.37 (dd, 1H), 7.18 (dd, 1H), 7.10 (m, 1H), 4.97 (s, 2H), 1.19 (s, 12H). ^13^C NMR (100 MHz, CDCl_3_) δ 162.52 (d, *J* = 247.2 Hz), 156.51, 151.30, 150.44 (d, *J* = 12.1 Hz), 149.68, 144.57, 126.83 (d, *J* = 9.6 Hz), 121.87, 114.84 (d, *J* = 3.4 Hz), 111.65 (d, *J* = 22.1 Hz), 103.90 (d, *J* = 25.6 Hz), 84.15, 47.40, 24.98. The ^13^C resonance for a carbon atom bearing boron is not observed. LC-MS (ESI) calcd for C_19_H_20_BFN_2_O_4_*m*/*z* [M+H]^+^ = 371.19, found 371.19.

### Synthesis of 5-fluoro-2-(((3-(4,4,5,5-tetramethyl-1,3,2-dioxaborolan-2-yl)pyridin-2-yl)amino)methyl)phenol (**S5**)

In the following preparation, in which dioxane solvent was not anhydrous, the hydrolyzed product **S5** was obtained instead of **S4**. A mixture of 3-(3-bromopyridin-2-yl)-7-fluoro-3,4-dihydro-*2H*-benzo[*e*][1,3]oxazin-2-one **S3** (10 g, 31.0 mmol), B_2_Pin_2_ (15.7 g, 61.9 mmol), Pd(dppf)Cl_2_ (2.25 g, 3.10 mmol), KOAc (9.1 g, 92.9 mmol), and 120 mL of dioxane was stirred at 85 °C overnight under a nitrogen atmosphere. After being cooled, the reaction mixture was concentrated, and the crude product was purified by silica gel chromatography to provide **S5** (5.2 g, 49%). ^1^H NMR (400 MHz, DMSO-*d*_6_) δ 10.76 (s, 1H), 8.11 (dd, *J* = 5.0, 1.8 Hz, 1H), 7.71 (d, *J* = 5.3 Hz, 1H), 7.19 (m, 1H), 6.83 (t, *J* = 6 Hz, 1H), 6.61–6.52 (m, 3H), 4.45 (d, *J* = 6 Hz, 2H), 1.30 (s, 12H).

### Synthesis of 5-fluoro-2-(((3-(pyridazin-3-yl)pyridin-2-yl)amino)methyl)phenol (**2**)

To a solution of **S5** (400 mg, 1.16 mmol), 3-bromopyridazine (220 mg, 1.40 mmol), and K_2_CO_3_ (93 mg, 1.40 mmol) in dioxane (5 mL), and H_2_O (0.5 mL) was added Pd(dppf)Cl_2_ (85 mg, 0.116 mmol) under a nitrogen atmosphere. The reaction was stirred at 100 °C for 2 h. The cooled solution was partitioned between CH_2_Cl_2_ (10 mL) and water (10 mL), and the organic layer was separated. The aqueous layer was extracted with CH_2_Cl_2_ (2 × 10 mL). The combined organic layer was washed with brine (20 mL), dried over anhydrous Na_2_SO_4_, filtered, and concentrated *in vacuo*. The crude product was purified by prep-HPLC to afford **2** (120 mg, yield: 35%) as a white solid. ^1^H NMR (400 MHz, DMSO-*d*_6_) δ 10.68 (br s, 1H), 9.55 (t, *J* = 5.6 Hz, 1H), 9.18 (t, *J* = 3.6 Hz, 1H), 8.31 (d, *J* = 1.6 Hz, 1H), 8.21–8.16 (m, 2H), 7.84–7.79 (m, 1H), 7.25 (t, *J* = 8.4 Hz, 1H), 6.78–6.75 (m, 1H), 6.62–6.54 (m, 2H), 4.62 (d, *J* = 5.6 Hz, 2H). ^13^C NMR (101 MHz, DMSO-*d*_6_) δ 162.4 (d, *J* = 241.4 Hz), 159.8, 157.4 (d, *J* = 11.4 Hz), 156.2, 150.3, 149.7, 138.0, 130.9 (d, *J* = 10.2 Hz), 128.3, 126.1, 123.0 (d, *J* = 2.8 Hz), 113.3, 112.3, 105.6 (d, *J* = 21.0 Hz), 103.0 (d, *J* = 23.6 Hz), 40.6. MS calcd: 296.11; found: 297.2 [M+H]^+^.

### Synthesis of 5-fluoro-2-(((3-(pyrazin-2-yl)pyridin-2-yl)amino)methyl)phenol (**4**)

A solution of **S5** (0.5 g, 1.31 mmol), 2-bromopyrazine (177 mg, 1.19 mmol), Pd(dppf)Cl_2_ (137 mg, 0.19 mmol), and K_2_CO_3_ (328.5 mg, 2.38 mmol) in dioxane (3 mL) and H_2_O (0.3 mL) was stirred at 110 °C under microwave irradiation for 2 h. After cooling, the solution was concentrated, and the crude product was purified by silica gel chromatography to provide **4** (332 mg, yield: 94%). ^1^H NMR (400 MHz, DMSO-*d*_6_) δ 10.66 (s, 1H), 9.26 (d, *J* = 1.2 Hz, 1H), 9.11 (t, *J* = 5.9 Hz, 1H), 8.70–8.63 (m, 1H), 8.59 (d, *J* = 2.6 Hz, 1H), 8.24 (dd, *J* = 7.6, 1.5 Hz, 1H), 8.17 (dd, *J* = 4.9, 1.7 Hz, 1H), 7.21 (t, *J* = 8.4 Hz, 1H), 6.73 (dd, *J* = 7.6, 4.9 Hz, 1H), 6.63–6.51 (m, 2H), 4.57 (d, *J* = 5.9 Hz, 2H). MS (ESI^+^, *m*/*z*) calcd for C_16_H_13_FN_4_O: 296.1; found: 297.1 [M+H]^+^.

### Synthesis of 2-(([2,3′-bipyridin]-2′-ylamino)methyl)-5-fluorophenol (**5**)

To a solution of **S5** (400 mg, 1.16 mmol), 2-bromopyridine (220 mg, 1.40 mmol), and K_2_CO_3_ (193 mg, 1.40 mmol) in dioxane (5 mL) and H_2_O (0.5 mL) was added Pd(dppf)Cl_2_ (91 mg, 0.127 mmol) under a nitrogen atmosphere. The reaction mixture was stirred at 100 °C for 2 h. The cooled mixture was partitioned between CH_2_Cl_2_ (10 mL) and water (10 mL), and the organic layer was separated. The aqueous layer was extracted with CH_2_Cl_2_ (2 × 10 mL). The combined organic layer was washed with brine (20 mL), dried over anhydrous Na_2_SO_4_, filtered, and concentrated *in vacuo*. The crude product was purified by prep-HPLC to afford **5** (150 mg, 44%) as a white solid. ^1^H NMR (400 MHz, DMSO-*d*_6_) δ 10.86 (s, 1H), 9.67 (t, *J* = 6.0 Hz, 1H), 8.65–8.63 (m, 1H), 8.11–8.08 (m, 2H), 7.97–7.78 (m, 2H), 7.38–7.36 (m, 1H), 7.23–7.21 (m, 1H), 6.71–6.68 (m, 1H), 6.61–6.53 (m, 2H), 4.56 (d, *J* = 6.0 Hz, 1H). ^13^C NMR (101 MHz, DMSO-*d*_6_) δ 162.4 (d, *J* = 241.3 Hz), 157.4 (d, *J* = 11.4 Hz), 156.7, 156.3, 148.4, 148.0, 138.1, 137.2, 130.9 (d, *J* = 10.3 Hz), 123.4 (d, *J* = 2.8 Hz), 122.3, 122.3, 116.1, 112.2, 105.7 (d, *J* = 21.0 Hz), 103.1 (d, *J* = 23.4 Hz), 40.6. MS calcd for C_17_H_14_FN_3_O: 295.11; found: 296.2 [M+H]^+^.

### Synthesis of 5-fluoro-2-(((3-(5-methylpyrimidin-2-yl)pyridin-2-yl)amino)methyl)phenol (**6**)

A mixture of 2-bromo-5-methylpyrimidine (9.3 mg, 0.054 mmol), intermediate **S4** (24 mg, 0.065 mmol), Pd(dppf)Cl_2_ (3.0 mg, 4.1 μmol), and Cs_2_CO_3_ (44 mg, 0.135 mmol) in 0.23 mL of mixed solvent (10:1 dioxane:H_2_O) was stirred at 130 °C for 1 h using microwave irradiation. After cooling, the mixture was treated with water and extracted with EtOAc. The combined organic layers were dried and filtered, and the residue was purified by silica gel chromatography (0–50% EtOAc in hexanes) to afford **6** (16 mg, 95%). ^1^H NMR (400 MHz, CDCl_3_) δ 10.04 (br s, 1H), 8.81 (dd, *J* = 7.7, 1.8 Hz, 1H), 8.63 (s, 2H), 8.19 (dd, *J* = 5.1, 1.8 Hz, 1H), 7.23 (dd, *J* = 8.4, 6.7 Hz, 1H), 6.72 (dd, *J* = 7.7, 5.1 Hz, 1H), 6.63 (dd, *J* = 10.7, 2.7 Hz, 1H), 6.54 (ddd, *J* = 8.4, 8.4, 2.7 Hz, 1H), 4.58 (d, *J* = 6.4 Hz, 2H), 2.36 (s, 3H). ^13^C NMR (100 MHz, methanol-*d*_4_) δ 163.2 (d, *J* = 242.8 Hz), 160.9, 157.3 (d, *J* = 11.6 Hz), 156.6, 156.4, 148.2, 139.0, 131.2 (d, *J* = 10.2 Hz), 128.3, 122.6 (d, *J* = 3.0 Hz), 114.9, 111.3, 105.6 (d, *J* = 21.4 Hz), 103.2 (d, *J* = 23.6 Hz), 40.1, 13.9. LRMS (ESI^+^) calcd for C_17_H_16_FN_4_O ([M+H]^+^): 311.13; found: 311.15

### Synthesis of 2-(((3-(1*H*-imidazol-2-yl)pyridin-2-yl)amino)methyl)-5-fluorophenol (**7**)

To a solution of **S5** (400 mg, 1.16 mmol), 2-bromo-1*H*-imidazole (200 mg, 1.40 mmol), and K_2_CO_3_ (93 mg, 1.40 mmol) in dioxane (5 mL) and H_2_O (0.5 mL) was added Pd(dppf)Cl_2_ (85 mg, 0.116 mmol) under a nitrogen atmosphere. The reaction mixture was stirred at 100 °C for 2 h in a microwave reactor. The cooled solution was partitioned between CH_2_Cl_2_ (10 mL) and water (10 mL). The organic layer was separated and extracted with CH_2_Cl_2_ (2 × 10 mL). The combined organic layer was washed with brine (20 mL), dried over anhydrous Na_2_SO_4_, filtered, and concentrated *in vacuo*. The crude product was purified by prep-HPLC to afford compound **7** (110 mg, 33%) as a white solid. ^1^H NMR (400 MHz, DMSO-*d*_6_) δ 12.63 (br s, 1H), 10.80 (br s, 1H), 9.69 (t, *J* = 5.6 Hz, 1H), 8.06–8.02 (m, 2H), 7.26–7.13 (m, 3H), 6.68–6.56 (m, 3H), 4.57 (d, *J* = 3.6 Hz, 2H). ^13^C NMR (101 MHz, DMSO-*d*_6_) δ 162.4 (d, *J* = 242.4 Hz), 157.4 (d, *J* = 11.0 Hz), 154.8, 147.0, 144.7, 133.4, 130.9 (d, *J* = 10.2 Hz), 128.0, 123.3 (d, *J* = 2.7 Hz), 118.0, 111.5, 108.8, 105.7 (d, *J* = 21.1 Hz), 103.1 (d, *J* = 23.5 Hz), 39.8. LC-MS calcd: 284.11; found: 285.2 [M+H]^+^.

### Synthesis of 2-(((3-(1*H*-imidazol-4-yl)pyridin-2-yl)amino)methyl)-5-fluorophenol (**8**)

A solution of **S5** (0.40 g, 1.162 mmol), 4-bromo-1*H*-imidazole (200 mg, 1.36 mmol), Pd(dppf)Cl_2_ (85 mg, 0.116 mmol), and K_2_CO_3_ (193 mg, 1.395 mmol) in dioxane (5 mL) and H_2_O (0.5 mL) was stirred at 100 °C for 2 h in a microwave reactor. The solution was concentrated, and the crude residue was purified by silica gel chromatography (0–20% EtOAc in petroleum ether) and further purified by prep-HPLC to afford **8** as a white solid (70 mg, 21%). ^1^H NMR (400 MHz, DMSO-*d*_6_) δ 11.92 (br s, 1H), 9.30 (s, 1H), 7.90 (dd, *J* = 5.0, 1.7 Hz, 1H), 7.85–7.83 (m, 2H), 7.72 (d, *J* = 1.0 Hz, 1H), 7.25 (t, *J* = 7.6 Hz, 1H), 6.60–6.50 (m, 3H), 4.51 (s, 2H). MS (ESI^+^, *m*/*z*): calcd for C_15_H_13_FN_4_O: 284.1; found 285.2 [M+H]^+^.

### Synthesis of 2-(((3-(1*H*-pyrazol-3-yl)pyridin-2-yl)amino)methyl)-5-fluorophenol (**9**)

A mixture of intermediate **S2** (200 mg, 0.67 mmol), 3-(4,4,5,5-tetramethyl-1,3,2-dioxaborolan-2-yl)-1*H*-pyrazole (160 mg, 0.81 mmol), Pd(dppf)Cl_2_ (50 mg, 0.067 mmol), and KOAc (80 mg, 0.81 mmol) in 2 mL of mixed solvent (dioxane:H_2_O = 10:1) was stirred at 100 °C for 1 h. After cooling, the reaction mixture was extracted with EtOAc three times, and the combined organic phase was washed with brine, dried over Na_2_SO_4_, and concentrated *in vacuo*. The crude product was purified by silica gel chromatography (20–40% EtOAc in petroleum ether) followed by prep-HPLC to provide the desired product (**9**, 40 mg, 21%). ^1^H NMR (400 MHz, DMSO-*d*_6_) δ 13.06 (br, 1H), 10.93 (br s, 1H), 8.71 (s, 1H), 8.01 - 7.99 (m, 2H), 7.87 (d, *J* = 2.0 Hz), 7.25 (t, *J* = 8.4 Hz, 1H), 6.86 (d, *J* = 6 Hz, 1H), 6.65 (dd, *J* = 7.6, 5.2 Hz, 1H), 6.61–6.53 (m, 2H), 4.56 (d, *J* = 5.6 Hz, 2H). MS (ESI^+^, *m*/*z*): calcd for C_15_H_13_FN_4_O: 284.1; found 285.2 [M+H]^+^.

### Synthesis of 5-fluoro-2-(((3-(1-methyl-1*H*-pyrazol-5-yl)pyridin-2-yl)amino)methyl)phenol (**12**)

A mixture of 2-(((2-bromophenyl)amino)methyl)-5-fluorophenol (**S2**) (50 mg, 0.17 mmol), 1-methyl-5-(4,4,5,5-tetramethyl-1,3,2-dioxaborolan-2-yl)-1*H*-pyrazole (88 mg, 0.42 mmol), Pd(dppf)Cl_2_ (3.1 mg, 4.2 μmol), and Cs_2_CO_3_ (140 mg, 0.42 mmol) in 0.58 mL of mixed solvent (dioxane:H_2_O = 10:1) in a sealed tube was stirred at 130 °C for 1 h with microwave irradiation. After cooling, the reaction mixture was extracted with EtOAc, the organic extracts were combined and concentrated, and the crude product was purified by silica gel chromatography (0–50% EtOAc in hexanes) to afford 5-fluoro-2-(((3-(1-methyl-1*H*-pyrazol-5-yl)pyridin-2-yl)amino)methyl)phenol (**12**) as a yellow solid (40.7 mg, 81%). ^1^H NMR (400 MHz, CDCl_3_) δ 8.16 (d, *J* = 5.4, 1.8 Hz, 1H), 7.59 (d, *J* = 1.6 Hz, 1H), 7.37 (dd, *J* = 7.3, 1.8 Hz, 1H), 7.07 (dd, *J* = 8.3, 6.6 Hz, 1H), 6.75 (dd, *J* = 7.2, 5.4 Hz, 1H), 6.64 (d, *J* = 10.6, 2.6 Hz, 1H), 6.53 (ddd, *J* = 8.3, 8.3, 2.6 Hz, 1H), 6.32 (d, *J* = 1.6 Hz, 1H), 5.28 (br s, 1H), 4.41 (d, *J* = 6.4 Hz, 2H), 3.68 (s, 3H). ^13^C NMR (100 MHz, acetone-*d*_6_) δ 163.3 (d, *J* = 242.4 Hz), 158.2(d, *J* = 12.9 Hz), 155.6, 146.7, 139.9, 138.4, 137.3, 132.5 (d, *J* = 10.3 Hz), 123.0 (d, *J* = 3.1 Hz),112.2, 111.9, 106.8, 105.8 (d, *J* = 21.4 Hz), 104.1 (d, *J* = 23.3 Hz), 40.6, 36.2. LRMS (ESI^+^) calcd for C_16_H_16_FN_4_O ([M+H]^+^): 299.12; found: 299.14

### Synthesis of 5-fluoro-2-(((3-(thiazol-5-yl)pyridin-2-yl)amino)methyl)phenol (**13**)

To a solution of **S2** (704 mg, 2.37 mmol), 5-(4,4,5,5-tetramethyl-1,3,2-dioxaborolan-2-yl)thiazole (500 mg, 2.37 mmol), and K_2_CO_3_ (654 mg, 4.734 mmol) in dioxane (5 mL) and H_2_O (0.5 mL) was added Pd(dppf)Cl_2_ (173 mg, 0.237 mmol) under a nitrogen atmosphere in a sealed tube. The reaction mixture was stirred at 100 °C under microwave irradiation for 4 h. After cooling, the reaction mixture was partitioned between CH_2_Cl_2_ (10 mL) and water (10 mL). The organic layer was separated, and the aqueous layer was extracted with CH_2_Cl_2_ (2 × 10 mL). The combined organic phases were washed with brine (20 mL), dried over anhydrous Na_2_SO_4_, filtered, and concentrated *in vacuo*. The crude product was purified by silica gel chromatography (0–10% EtOAc in petroleum ether) to afford **13** (50 mg, 7%) as a white solid. ^1^H NMR (400 MHz, DMSO-*d*_6_) δ 10.82 (s, 1H), 9.21 (s, 1H), 8.06–8.05 (m, 2H), 7.52–7.50 (m, 1H), 7.15 (d, *J* = 4.0 Hz, 1H), 6.69–6.53 (m, 4H), 4.39 (d, *J* = 8.0 Hz, 2H). ^13^C NMR (101 MHz, DMSO-*d*_6_) δ 162.3 (d, *J* = 242.3 Hz), 157.2 (d, *J* = 11.3 Hz), 155.7, 155.1, 147.8, 142.5, 139.6, 133.7, 130.8 (d, *J* = 9.9 Hz), 123.2 (d, *J* = 2.8 Hz), 112.7, 111.7, 105.7 (d, *J* = 21.0 Hz), 103.2 (d, *J* = 23.5 Hz), 40.4. MS calcd for C_15_H_12_FN_3_OS: 301.07; found: 302.1 [M+H]^+^.

### Synthesis of 5-fluoro-2-(((3-(thiazol-4-yl)pyridin-2-yl)amino)methyl)phenol (**14**)

To a solution of **S5** (475 mg, 1.39 mmol), 4-bromothiazole (206 mg, 1.26 mmol), and K_2_CO_3_ (350 mg, 2.53 mmol) in dioxane (5 mL) and H_2_O (0.5 mL) was added Pd(dppf)Cl_2_ (91 mg, 0.127 mmol) under a nitrogen atmosphere. The reaction was stirred at 110 °C with microwave for 2 h. The mixture was partitioned between CH_2_Cl_2_ (10 mL) and water (10 mL). The organic layer was separated and extracted with CH_2_Cl_2_ (2 × 10 mL). The combined organic layer was washed with brine (20 mL), dried over anhydrous Na_2_SO_4_, filtered, and concentrated *in vacuo*. The crude product was purified by silica gel chromatography using a mixture of petroleum ether–ethyl acetate (0–25% EtOAc in petroleum ether) to afford **14** (198 mg, 52%) as a white solid. ^1^H NMR (400 MHz, DMSO-*d*_6_) δ 10.82 (s, 1H), 9.31 (d, *J* = 4.0 Hz, 1H), 8.20–8.19 (m, 1H), 8.07–8.05 (m, 1H), 8.00–7.98 (m, 2H), 7.23 (t, *J* = 8.0 Hz, 1H), 6.69–6.66 (m, 1H), 6.60–6.53 (m, 2H), 4.53 (d, *J* = 8.0 Hz, 2H). ^13^C NMR (101 MHz, DMSO-*d*_6_) δ 162.6 (d, *J* = 242.4 Hz), 157.4 (d, *J* = 11.4 Hz), 155.0, 154.7, 153.5, 147.4, 136.6, 131.0 (d, *J* = 10.3 Hz), 123.3 (d, *J* = 2.8 Hz), 116.5, 113.4, 112.4, 105.7 (d, *J* = 21.1 Hz), 103.2 (d, *J* = 23.6 Hz), 40.1. MS calcd for C_15_H_12_FN_3_OS: 301.07; found: 302.0 [M+H]^+^.

### Synthesis of 5-fluoro-2-(((3-(1-methyl-1*H*-pyrazol-3-yl)pyridin-2-yl)amino)methyl)phenol (**15**)

A mixture of 2-(((2-bromophenyl)amino)methyl)-5-fluorophenol (**S2**) (30 mg, 0.10 mmol), 1-methyl-3-(4,4,5,5-tetramethyl-1,3,2-dioxaborolan-2-yl)-1*H*-pyrazole (53 mg, 0.25 mmol), Pd(dppf)Cl_2_ (3.7 mg, 5.0 μmol), and Cs_2_CO_3_ (82 mg, 0.25 mmol) in 0.35 mL of mixed solvent (dioxane:H_2_O = 10:1) was stirred at 130 °C for 1 h in a sealed tube under microwave irradiation. After cooling, the mixture was treated with water and extracted with EtOAc. The combined organic layers were dried and concentrated, and the crude residue was purified by silica gel chromatography (0–50% EtOAc in hexanes) to provide 5-fluoro-2-(((3-(1-methyl-1*H*-pyrazol-3-yl)pyridin-2-yl)amino)methyl)phenol (**15**) (25.2 mg, 84%). ^1^H NMR (400 MHz, CDCl_3_) δ 8.79 (t, *J* = 5.9 Hz, 1H), 8.03 (dd, *J* = 5.2, 1.8 Hz, 1H), 7.76 (dd, *J* = 7.5, 1.8 Hz, 1H), 7.39 (d, *J* = 2.4 Hz, 1H), 7.22 (dd, *J* = 8.4, 6.8 Hz, 1H), 6.66–6.61 (m, 2H), 6.57 (d, *J* = 2.4 Hz, 1H), 6.53 (ddd, *J* = 8.4, 8.4, 2.6 Hz, 1H), 4.56 (d, *J* = 6.5 Hz, 2H), 3.98 (s, 3H). ^13^C NMR (100 MHz, acetone-*d*_6_) δ 164.2 (d, *J* = 242.3 Hz), 159.4, 155.1 (d, *J* = 7.7 Hz), 149.7, 145.3, 136.3, 133.2 (d, *J* = 10.3 Hz), 132.5, 124.3 (d, *J* = 3.1 Hz),113.8, 112.6, 106.4 (d, *J* = 21.5 Hz), 104.9 (d, *J* = 23.1 Hz), 104.1, 41.5, 39.2. LRMS (ESI^+^) calcd for C_16_H_16_FN_4_O ([M+H]^+^): 299.12; found: 299.14.

### Synthesis of 5-fluoro-2-(((3-(isothiazol-5-yl)pyridin-2-yl)amino)methyl)phenol (**16**)

A solution of **S5** (1.04 g, 3.02 mmol), 5-bromoisothiazole (450 mg, 2.74 mmol), Pd(dppf)Cl_2_ (200 mg, 0.274 mmol), and K_2_CO_3_ (756 mg, 5.486 mmol) in dioxane (5 mL) and H_2_O (0.5 mL) was stirred for 2 h at 110 °C in a microwave reactor. After cooling, the solution was concentrated, and the crude product was purified by silica gel chromatography to provide **16** (264 mg, 32%). ^1^H NMR (400 MHz, DMSO-*d*_6_) δ 10.67 (s, 1H), 8.66 (d, *J* = 1.7 Hz, 1H), 8.10 (dd, *J* = 5.0, 1.8 Hz, 1H), 7.65 (d, *J* = 1.8 Hz, 1H), 7.61 (dd, *J* = 7.4, 1.7 Hz, 1H), 7.17 (t, *J* = 7.6 Hz, 1H), 6.71 (dd, *J* = 7.4, 5.0 Hz, 1H), 6.63 (t, *J* = 5.8 Hz, 1H), 6.60–6.51 (m, 2H), 4.42 (d, *J* = 5.6 Hz, 2H). MS (ESI^+^, *m*/*z*): calcd for C_15_H_12_FN_3_OS: 301.1; found, 302.2 [M+H]^+^.

### Synthesis of 5-fluoro-2-(((3-(thiazol-2-yl)pyridin-2-yl)amino)methyl)phenol (**17**)

To a solution of **S5** (475 mg, 1.39 mmol), 2-bromothiazole (206 mg, 1.26 mmol), and K_2_CO_3_ (350 mg, 2.53 mmol) in dioxane (5 mL) and H_2_O (0.5 mL) was added Pd(dppf)Cl_2_ (91 mg, 0.127 mmol) under a nitrogen atmosphere. The reaction was stirred at 110 °C under microwave irradiation for 2 h. After cooling, the mixture was partitioned between CH_2_Cl_2_ (10 mL) and water (10 mL). The organic layer was separated and extracted with CH_2_Cl_2_ (2 × 10 mL). The combined organic layer was washed with brine (20 mL), dried over anhydrous Na_2_SO_4_, filtered, and concentrated *in vacuo*. The crude product was purified by silica gel chromatography (0–25% EtOAc in hexanes) to afford **17** (88 mg, 23%) as a white solid. ^1^H NMR (400 MHz, DMSO-*d*_6_) δ 10.47 (s, 1H), 9.29 (t, *J* = 4.0 Hz, 1H), 8.17–8.16 (m, 1H), 8.05–8.03 (m, 1H), 7.94–7.93 (m, 1H), 7.79 (d, *J* = 4.0 Hz, 1H), 7.22–7.20 (m, 1H), 6.71–6.68 (m, 1H), 6.62–6.54 (m, 2H), 4.53 (d, *J* = 8.0 Hz, 2H). ^13^C NMR (101 MHz, DMSO-*d*_6_) δ 167.5, 162.3 (d, *J* = 242.6 Hz), 157.2 (d, *J* = 11.2 Hz), 154.3, 149.8, 142.3, 137.4, 130.6 (d, *J* = 10.3 Hz), 123.1, 122.9 (d, *J* = 2.8 Hz), 112.2, 111.1, 105.7 (d, *J* = 21.1 Hz), 102.9 (d, *J* = 23.8 Hz), 39.8. MS calcd for C_15_H_12_FN_3_OS: 301.07; found: 302.0 [M+H]^+^.

### Synthesis of 5-fluoro-2-(((3-(1-methyl-1*H*-imidazol-4-yl)pyridin-2-yl)amino)methyl)phenol (**18**)

A mixture of intermediate **S4** (60.0 mg, 0.160 mmol), 4-bromo-1-methyl-1*H*-imidazole, HBr (47.0 mg, 0.190 mmol), Pd(dppf)Cl_2_ (12.0 mg, 16.0 μmol), and K_2_CO_3_ (27 mg, 0.190 mmol) in 0.690 mL of 10:1 dioxane:H_2_O was stirred at 100 °C for 2 h using microwave irradiation. After cooling, the mixture was treated with water and extracted with EtOAc. The combined organic layers were dried and concentrated, and the residue was purified by silica gel chromatography (0–5% MeOH in CH_2_Cl_2_) and then by prep-HPLC to provide **18** (11.5 mg, 24%). ^1^H NMR (400 MHz, DMSO-*d*_6_) δ 11.27 (br s, 1H), 9.19 (t, *J* = 6.1 Hz, 1H), 7.91 (dd, *J* = 5.0, 1.8 Hz, 1H), 7.77 (dd, *J* = 7.5, 1.6 Hz, 2H), 7.71 (d, *J* = 1.3 Hz, 1H), 7.26 (dd, *J* = 8.3, 7.1 Hz, 1H), 6.62–6.53 (m, 3H), 4.51 (d, *J* = 6.0 Hz, 2H), 3.73 (s, 3H). ^13^C NMR (100 MHz, DMSO-*d*_6_) δ 162.46 (d, *J* = 241.4 Hz), 157.65, 157.53, 154.75, 144.92, 139.15, 137.56, 133.50, 131.22 (d, *J* = 10.3 Hz), 123.70 (d, *J* = 2.8 Hz), 118.44, 113.33, 112.03, 105.70 (d, *J* = 21.0 Hz), 103.38 (d, *J* = 23.2 Hz), 33.76. MS calcd for C_16_H_15_FN_4_O: 298.12; found: 299.19 [M+H]^+^.

### Synthesis of 5-fluoro-2-(((3-(isothiazol-3-yl)pyridin-2-yl)amino)methyl)phenol (**19**)

To a solution of **S5** (1.03 g, 3.02 mmol), 3-bromoisothiazole (450 mg, 2.74 mmol), and K_2_CO_3_ (756 mg, 5.49 mmol) in dioxane (5 mL) and H_2_O (0.5 mL) was added Pd(dppf)Cl_2_ (198 mg, 0.274 mmol) under a nitrogen atmosphere. The reaction was stirred at 110 °C with microwave irradiation for 2 h. The mixture was then partitioned between CH_2_Cl_2_ (10 mL) and water (10 mL), and the organic layer was separated and extracted with CH_2_Cl_2_ (2 × 10 mL). The combined organic layer was washed with brine (20 mL), dried over anhydrous Na_2_SO_4_, filtered, and concentrated *in vacuo*. The crude product was purified by silica gel chromatography (0–25% EtOAc, petroleum ether) to afford **19** (100 mg, 12%) as a white solid. ^1^H NMR (400 MHz, DMSO-*d*_6_) δ 10.70 (br s, 1H), 9.22–9.15 (m, 2H), 8.23–8.20 (m, 1H), 8.13–8.12 (m, 1H), 8.05 (d, *J* = 4.0 Hz, 1H), 7.24 (t, *J* = 8.0 Hz, 1H), 6.71–6.68 (m, 1H), 6.61–6.54 (m, 2H), 4.61 (d, *J* = 4.0 Hz, 2H). ^13^C NMR (101 MHz, DMSO-*d*_6_) δ 166.6, 162.4 (d, *J* = 242.5 Hz), 157.4 (d, *J* = 11.3 Hz), 155.6, 150.0, 148.6, 138.0, 130.9 (d, *J* = 10.3 Hz), 123.1, 123.0 (d, *J* = 3.5 Hz), 112.7, 111.9, 105.7 (d, *J* = 21.1 Hz), 103.1 (d, *J* = 23.7 Hz), 40.0. MS calcd for C_15_H_12_FN_3_OS: 301.07; found: 302.1 [M+H]^+^.

### Synthesis of 5-fluoro-2-(((3-(1-methyl-1*H*-1,2,3-triazol-4-yl)pyridin-2-yl)amino)methyl)phenol (**24**)

A mixture of 4-bromo-1-methyl-1*H*-1,2,3-triazole (32.0 mg, 0.190 mmol), intermediate **S4** (60.0 mg, 0.160 mmol), Pd(dppf)Cl_2_ (12.0 mg, 16.0 μmol), and K_2_CO_3_ (27 mg, 0.190 mmol) in 0.690 mL of 7:1 dioxane:H_2_O was stirred at 100 °C for 2 h using microwave irradiation. Subsequently, the mixture was extracted with EtOAc, and the crude compound was purified by silica gel chromatography (0 to 5% MeOH in CH_2_Cl_2_) to provide compound **24** (5.3 mg, 11%). ^1^H NMR (400 MHz, DMSO-*d*_6_) δ 10.87 (s, 1H), 8.64 (s, 1H), 8.47 (t, *J* = 5.9 Hz, 1H), 8.05 (dd, *J* = 5.0, 1.8 Hz, 1H), 7.86 (dd, *J* = 7.4, 1.8 Hz, 1H), 7.27 (dd, *J* = 8.4, 7.0 Hz, 1H), 6.68 (dd, *J* = 7.4, 4.9 Hz, 1H), 6.64–6.53 (m, 2H), 4.59 (d, *J* = 5.8 Hz, 2H), 4.13 (s, 3H). ^13^C NMR (100 MHz, DMSO-*d*_6_) δ 162.40 (d, *J* = 241.6 Hz), 157.44 (d, *J* = 11.4 Hz), 154.61, 146.96, 145.63, 135.45, 131.07, 130.96, 123.79, 123.26 (d, *J* = 2.9 Hz), 112.15, 109.62, 105.66 (d, *J* = 21.0 Hz), 103.12 (d, *J* = 23.5 Hz), 37.13. MS calcd for C_15_H_14_FN_5_O: 299.12; found: 300.19 [M+H]^+^.

### Synthesis of 2-(((3-(1,2,4-triazin-3-yl)pyridin-2-yl)amino)methyl)-5-fluorophenol (**25**)

To a solution of intermediate **S5** (400 mg, 1.16 mmol), 3-(methylthio)-1,2,4-triazine (74 mg, 0.58 mmol), and copper(I) 3-methylsalicylate (124 mg, 0.58 mmol) in dioxane (5 mL) was added Pd(PPh_3_)_4_ (85 mg, 0.116 mmol) under a nitrogen atmosphere. The reaction mixture was stirred at 80 °C for 4 h, cooled, and partitioned between EtOAc (10 mL) and water (10 mL). The organic layer was separated and extracted with EtOAc (2 × 10 mL). The combined organic layer was washed with brine (20 mL), dried over anhydrous Na_2_SO_4_, filtered, and concentrated *in vacuo*. The crude product was purified by prep-HPLC to afford **25** (12 mg, 7% yield) as a light green solid. ^1^H NMR (400 MHz, DMSO-*d*_6_) δ 10.53 (s, 1H), 9.49 (t, *J* = 6.0 Hz, 1H), 9.33 (d, *J* = 2.0 Hz, 1H), 8.96 (d, *J* = 1.6 Hz, 1H), 8.72–8.70 (m, 1H), 8.31–8.30 (m, 1H), 7.26 (t, *J* = 8.0 Hz, 1H), 6.81–6.78 (m, 1H), 6.63–6.54 (m, 2H), 4.68 (d, *J* = 6.0 Hz, 2H). MS calcd for C_15_H_12_FN_5_O: 297.10; found: 298.2 [M+H]^+^.

### Synthesis of 5-fluoro-2-(((3-(thiophen-2-yl)pyridin-2-yl)amino)methyl)phenol (**26**)

To a solution of **S2** (400 mg, 1.35 mmol), 4,4,5,5-tetramethyl-2-(thiophen-2-yl)-1,3,2-dioxaborolane (311 mg, 1.48 mmol), and K_2_CO_3_ (372 mg, 2.7 mmol) in dioxane (5 mL) and H_2_O (0.5 mL) was added Pd(dppf)Cl_2_ (99 mg, 0.135 mmol) under a nitrogen atmosphere. The reaction mixture was stirred at 120 °C with microwave irradiation for 1 h. After cooling, the solution was partitioned between CH_2_Cl_2_ (10 mL) and water (10 mL). The organic layer was separated, and the aqueous layer was extracted with CH_2_Cl_2_ (2 × 10 mL). The combined organic layer was washed with brine (20 mL), dried over anhydrous Na_2_SO_4_, filtered, and concentrated *in vacuo*. The crude product was purified by silica gel chromatography (0–10% EtOAc in petroleum ether) and further purified by prep-HPLC to afford compound **26** (60 mg, 15% yield) as a white solid. ^1^H NMR (400 MHz, DMSO-*d*_6_) δ 10.93 (s, 1H), 8.03–8.02 (m, 1H), 7.66–7.65 (m, 1H), 7.50–7.47 (m, 1H), 7.30–7.29 (m, 1H), 7.21–7.19 (m, 2H), 6.68–6.64 (m, 1H), 6.58–6.53 (m, 3H), 4.41 (d, *J* = 8.0 Hz, 2H). ^13^C NMR (101 MHz, DMSO-*d*_6_) δ 162.4 (d, *J* = 242.4 Hz), 157.4 (d, *J* = 11.1 Hz), 155.2, 146.9, 138.7, 138.6, 131.1 (d, *J* = 13.0 Hz), 128.6, 127.0, 126.9, 123.3 (d, *J* = 2.7 Hz), 115.2, 112.8, 105.7 (d, *J* = 21.0 Hz), 103.2 (d, *J* = 23.9 Hz), 40.6. MS calcd for C_16_H_13_FN_2_OS: 300.07; found: 301.3 [M+H]^+^.

### Synthesis of 5-fluoro-2-(((3-(1-methyl-1*H*-imidazol-4-yl)pyridin-2-yl)amino)methyl)phenol (**28**)

A mixture of 2-bromoimidazo[1,2-*a*]pyridine (47.0 mg, 0.190 mmol), intermediate **S4** (60.0 mg, 0.160 mmol), Pd(dppf)Cl_2_ (12.0 mg, 16.0 μmol), and K_2_CO_3_ (27 mg, 0.190 mmol) in 0.69 mL of 7:1 dioxane:H_2_O was stirred at 100 °C for 2 h using microwave irradiation. After cooling, the mixture was diluted with water and extracted with EtOAc. The combined organic layer was dried and concentrated, and the crude residue was purified by silica gel chromatography (0–50% EtOAc in hexanes) and then by prep-HPLC to provide 5-fluoro-2-(((3-(1-methyl-1*H*-imidazol-4-yl)pyridin-2-yl)amino)methyl)phenol (19.8 mg, 37%). ^1^H NMR (400 MHz, CDCl_3_) δ 9.67 (br s, 1H), 8.12 (dt, *J* = 6.8, 1.3 Hz, 1H), 8.04 (dd, *J* = 5.4, 1.7 Hz, 1H), 7.81 (s, 1H), 7.75 (dd, *J* = 7.5, 1.7 Hz, 1H), 7.63 (dd, *J* = 9.1, 1.0 Hz, 1H), 7.27–7.20 (m, 2H), 6.84 (ddd, *J* = 6.8, 6.8, 1.2 Hz, 1H), 6.65–6.60 (m, 2H), 6.53 (ddd, *J* = 8.4, 8.4, 2.6 Hz, 1H), 4.58 (d, *J* = 5.4 Hz, 2H). ^13^C NMR (100 MHz, acetone-*d*_6_) δ 163.3 (d, *J* = 242 Hz), 158.6 (d, *J* = 13.5 Hz), 155.1, 145.1, 144.1, 143.2, 135.4, 132.4 (d, *J* = 10.5 Hz), 126.4, 125.1, 123.5 (d, *J* = 3.1 Hz), 116.7, 113.0, 111.7, 109.6, 105.6 (d, *J* = 21.5 Hz), 104.1 (d, *J* = 23.1 Hz), 40.6. MS (ESI^+^) calcd for C_19_H_16_FN_4_O ([M+H]^+^): 335.13; found: 335.25

### Synthesis of 2-(((3-(benzo[*d*]thiazol-2-yl)pyridin-2-yl)amino)methyl)-5-fluorophenol (**29**)

A mixture of intermediate **S4** (45 mg, 0.12 mmol), 2-bromobenzo[*d*]thiazole (13 mg, 60.7 μmol), Pd(dppf)Cl_2_ (1.11 mg, 1.52 μmol), and Cs_2_CO_3_ (82 mg, 0.25 mmol) in 0.23 mL of 10:1 dioxane:H_2_O was stirred at 130 °C for 1 h using microwave irradiation. Subsequently, the mixture was extracted with EtOAc, and this crude compound was purified by silica gel chromatography (20–40% EtOAc in hexanes) to provide **29** as an off-white solid (6.5 mg, 30%). ^1^H NMR (400 MHz, methylene chloride-*d*_2_ and methanol-*d*_4_, 3:1) δ 8.28–8.19 (m, 1H), 8.18–8.12 (m, 1H), 8.09 (d, *J* = 8.2 Hz, 1H), 7.96 (d, *J* = 7.9 Hz, 1H), 7.59–7.53 (m, 1H), 7.51–7.44 (m, 1H), 7.38 (dd, *J* = 8.1, 6.8 Hz, 1H), 6.82 (dd, *J* = 7.6, 5.4 Hz, 1H), 6.68–6.57 (m, 2H), 4.73 (s, 1H). ^13^C NMR (100 MHz, methylene chloride-*d*_2_ and methanol-*d*_4_, 3:1) δ 152.89, 139.90, 133.26, 131.80 (d, *J* = 10.0 Hz), 126.65, 125.96, 122.81, 121.41, 113.06, 111.74, 106.41 (d, *J* = 21.6 Hz), 104.19 (d, *J* = 23.7 Hz), 41.13. LC-MS (ESI) calcd for C_19_H_14_FN_3_OS *m*/*z* [M+H]^+^ = 352.4; found: 352.08.

### Synthesis of 2-(((3-(1*H*-benzo[*d*]imidazol-2-yl)pyridin-2-yl)amino)methyl)-5-fluorophenol (**30**)

A mixture of intermediate **S4** (84.5 mg, 0.23 mmol), 2-bromo-1*H*-benzo[*d*]imidazole (30 mg, 0.15 mmol), Pd(dppf)Cl_2_ (11.1 mg, 15.2 μmol), and Cs_2_CO_3_ (124 mg, 0.38 mmol) in 0.46 mL of 10:1 dioxane:H_2_O was stirred at 130 °C for 1 h using microwave irradiation. Subsequently, the mixture was extracted with 1% MeOH in CH_2_Cl_2_, dried, and concentrated, and the crude residue was purified by prep-HPLC to provide compound **30** as a white solid (15 mg, 30%). ^1^H NMR (400 MHz, methylene chloride-*d*_2_ and methanol-*d*_*4*_, 3:1) δ 8.17–8.10 (m, 1H), 7.66 (s, 1H), 7.36 (td, *J* = 7.4, 6.7, 2.1 Hz, 1H), 7.32–7.26 (m, 1H), 6.75 (dd, *J* = 7.5, 5.1 Hz, 1H), 6.62–6.56 (m, 1H), 4.64 (s, 1H). ^13^C NMR (100 MHz, methylene chloride-*d*_2_ and methanol-*d*_*4*_, 3:1) δ 163.40 (d, *J* = 243.7 Hz), 157.65 (d, *J* = 12.2 Hz), 155.29, 149.97, 146.91, 135.72, 132.19 (d, *J* = 10.3 Hz), 122.81 (d, *J* = 3.1 Hz), 111.39, 108.93, 106.32 (d, *J* = 21.5 Hz), 104.47 (d, *J* = 23.1 Hz), 40.86. LC-MS (ESI) calculated for C_19_H_15_FN_4_O *m*/*z* [M+H]^+^ = 335.35; found: 335.18.

### Synthesis of 6-amino-*N*-((2′-((4-fluoro-2-hydroxybenzyl)amino)-[2,3′-bipyridin]-5-yl)methyl)hexanamide (**31**, Scheme S9)

Step 1: *tert*-butyl ((6-bromopyridin-3-yl)methyl)carbamate (**31a**, 100 mg, 0.348 mmol) was taken in a mixture of TFA and dichloromethane (1.25 mL, TFA/DCM = 1:4) and stirred at 25 °C for 4 h. Subsequently, the solvent was removed by rotary evaporation followed by high vacuum. This crude residue **31b** was used in the next reaction without further purification. ^1^H NMR (400 MHz, methanol-*d*_*4*_) δ 8.47 (d, *J* = 2.5 Hz, 1H), 7.82 (dd, *J* = 8.3, 2.5 Hz, 1H), 7.72 (d, *J* = 8.3 Hz, 1H), 4.19 (s, 2H).

Step 2: **31b** (105 mg, 0.348 mmol, 1.0 equiv), 6-((*tert*-butoxycarbonyl)amino)hexanoic acid (96.6 mg, 0.418 mmol, 1.20 equiv), 3-(((ethylimino)methylene)amino)-*N*,*N*-dimethylpropan-1-amine hydrochloride (EDC, 200 mg, 1.04 mmol, 3.0 equiv), DIPEA (145.4 μL, 0.836 mmol, 2.4 equiv), and *N*,*N*-dimethylpyridin-4-amine (DMAP, 42.6 mg, 0.348 mmol, 1.0 equiv) were mixed in 2.0 mL of DMF and stirred at 25 °C for 48 h. Water (5 mL) was then added, and the mixture was extracted with EtOAc (5 mL). The organic layer was separated, and the aqueous layer was extracted with more EtOAc (2 × 5 mL). The combined organic layers were washed with 1 M HCl (5 mL), sat. NaHCO_3_ (5 mL), and brine (5 mL), dried over anhydrous Na_2_SO_4_, filtered, and concentrated *in vacuo*. The crude product was purified by silica gel chromatography using a mixture of hexane–ethyl acetate (90:10 to 0:100 v/v) as eluent to afford **31c** (73 mg, yield: 53%). ^1^H NMR (400 MHz, CDCl_3_) δ 8.30 (d, *J* = 2.5 Hz, 1H), 7.54 (dd, *J* = 8.2, 2.5 Hz, 1H), 7.46 (d, *J* = 8.1 Hz, 1H), 6.02 (s, 1H), 4.54 (s, 1H), 4.41 (d, *J* = 6.0 Hz, 2H), 3.10 (t, *J* = 7.0 Hz, 2H), 2.23 (t, *J* = 7.5 Hz, 2H), 1.71–1.63 (m, 2H), 1.53–1.46 (m, 2H), 1.43 (s, 9H), 1.38–1.30 (m, 2H).

Step 3: A mixture of **31c** (40 mg, 0.10 mmol), intermediate **S4** (44 mg, 0.12 mmol), Pd(dppf)Cl_2_ (5.5 mg, 7.5 μmol), and Cs_2_CO_3_ (81 mg, 0.25 mmol) in 0.46 mL of mixed solvent (dioxane/H_2_O = 10/1) was stirred at 60 °C for 4 h. Subsequently, the mixture was extracted with EtOAc three times, and the combined organic phase was washed with sat. NaHCO_3_, H_2_O, and brine, dried over Na_2_SO_4_, and concentrated *in vacuo*. The crude residue was purified by prep-HPLC to provide compound **31d** as a white solid (16.2 mg, 28%) as a white solid. ^1^H NMR (400 MHz, methanol-*d*_*4*_) δ 8.56 (d, *J* = 7.2 Hz, 1H), 8.30 (br s, 1H), 8.08–8.00 (m, 2H), 7.88–7.78 (m, 2H), 7.33–7.21 (m, 1H), 6.76–6.70 (m, 1H), 6.56–6.50 (m, 2H), 4.54 (s, 2H), 4.43 (s, 2H), 3.03 (t, *J* = 7.0 Hz, 2H), 2.28 (t, *J* = 7.3 Hz, 2H), 1.71–1.63 (m, 2H), 1.53–1.45 (m, 2H), 1.43 (s, 9H), 1.39–1.27 (m, 2H).

Step 4: In a 4-mL vial equipped with a stirring bar and a cap was placed 16.2 mg (27.8 μmol) of **31d**, and the vial was cooled to 0 °C. Next a solution of 4 M HCl in dioxane (0.150 mL), dichloromethane (0.10 mL), and methanol (0.10 mL) was added, and the reaction mixture was stirred for 4 h at room temperature. At this time the reaction was judged complete, and the reaction mixture was concentrated *in vacuo*. The crude product was purified by prep-HPLC (0.1% formic acid MeCN/H_2_O 0% to 50%) to afford **31** (14.3 mg, 97%) as a white solid. ^1^H NMR (400 MHz, methanol-*d*_*4*_) δ 8.57 (s, 1H), 8.07–8.03 (m, 2H), 7.87–7.83 (m, 2H), 7.27 (t, *J* = 7.4 Hz, 1H), 6.74 (dd, *J* = 7.5, 5.2 Hz, 1H), 6.59–6.52 (m, 2H), 4.55 (s, 1H), 4.44 (s, 2H), 2.93 (t, *J* = 7.8 Hz, 2H), 2.32 (t, *J* = 7.4 Hz, 2H), 1.77–1.63 (m, 4H), 1.46–1.41 (m, 2H). ^13^C NMR (101 MHz, methanol-*d*_*4*_) δ 174.5, 163.2 (d, *J* = 243.2 Hz), 157.3, 155.0, 154.6, 146.6, 144.4, 137.7, 136.7, 133.3, 131.1, 121.9, 121.4, 117.5, 111.6, 105.6, (d, *J* = 21.6 Hz), 103.1 (d, *J* = 23.8 Hz), 40.0, 39.13, 39.05, 35.1, 26.9, 25.6, 24.8. MS (ESI^+^, *m*/*z*): calcd for C_24_H_28_FN_5_O_2_: 437.2; found: 438.4 [M+H]^+^.

### Solution-Phase Labeling of **31** to Form Europium Chelate **32**

For the homogeneous solution-based QRET assay, compound **31** was labeled with the commercial reagent europium(III) 2,2′,2″,2‴-(((4′-(4-((4,6-dichloro-1,3,5-triazin-2-yl)amino)phenyl)-[2,2′:6′,2″-terpyridine]-6,6″-diyl)bis(methylene))bis(azanetriyl))tetraacetate. Compound **31** (0.45 mg, 1.0 mmol) was dissolved into 30 μL of DMSO and further diluted with 50 μL of 50 mM Na_2_CO_3_/NaHCO_3_ buffer, pH 9.8, and the europium chelate reagent (0.9 mg, 1.0 mmol) was dissolved into 100 μL of 50 mM Na_2_CO_3_/NaHCO_3_ buffer, pH 9.8. The solutions were combined and incubated at room temperature for 18 h. Eu-chelate-labeled conjugate **32** was purified using reversed-phase chromatography on a Dionex ultimate 3000 LC system from Thermo Fischer Scientific and Ascentis RP-amide C18 column from Sigma-Aldrich. The HPLC purification was performed using a linear CH_3_CN gradient from 10 to 60% (over 15 min) in 40 mM triethylammonium acetate at a flow rate of 1 mL/min.

The mass of the Eu-chelate-labeled **32** was confirmed with LC-MS analyses using a Waters Acquity RDa system. Column: XBridge BEH C18, 130 Å, 3.5 μm, 4.6 mm × 30 mm (Waters, Milford, MA). Eluent A: H_2_O with 0.1% formic acid. Eluent B: MeOH with 0.1% formic acid. Gradient/time: initial composition 0 min 2% B, linear increase over 2.2 min to 100% B, 2.6 min 100% B, linear decrease until 2.8 min to 5% B, equilibrating until 3 min 2% B. High-resolution mass calculated for C_58_H_52_ClEuFN_14_O_10_ was 1311.2859, [M+H]^+^ product ion calculated was 1312.2937 and found 1312.3039, and [M+2H]^2+^ product ion calculated was 656.6508 and found 656.6482.

### Computational Methods

All computations were performed using Gaussian16 with input file generation, structure manipulation, and output file parsing done using AaronTools.^44,45^ To construct a minimal binding site model, we started from the previously reported structure for **1** (PDB: 4NBL), removed all of the protein except tyrosines 198^A^ and 198^B^ and the ligand, and then added hydrogens. We considered all four combinations of the OH directions for the two Tyr hydroxyl groups. The heterocycle in these four structures was then modified as needed to construct analogous bound structures for the ligands **2**–**30**. For nonsymmetric six-membered heterocycles with heteroatoms at both *ortho*-positions, we constructed four additional structures by rotating the heterocycle 180°. We did this for all five-membered heterocycles regardless of the position of the heteroatoms. For heterocycles in conformations in which the IMHB is not feasible, we considered two different nonplanar conformations of the heterocycle (i.e., tilted toward Tyr 198^A^ or 198^B^). So, depending on the heterocycle, we considered between 4 and 16 initial starting conformations. All of these structures were optimized to local energy minima at the SMD(water)-wB97X-D/def2SVP level of theory with constraints on C_α_ and C_β_ of the Tyr sidechains and the oxygen of the ligand. These geometry optimizations were followed by single-point energies at the SMD(water)-wB97X-D/def2TZVP level of theory.^46−48^ We did a systematic search of low-lying conformations of the unbound ligands using Crest^49^ at the GFN2-XTB level of theory^50^ using an energy window of 10 kcal/mol and RMSD cutoff of 0.125 Å. The structures of all unique conformers were then fully optimized at the SMD(water)-wB97X-D/def2SVP level of theory, followed by single points using SMD(water)-wB97X-D/def2TZVP. The reported *E*_int_ values are calculated as the difference in energy between the energy of the lowest-energy bound complex (including possible tautomers, where applicable) and the sum of the energies of the lowest-energy unbound ligand (again, including possible tautomers) and two Tyr side chains in the optimized bound geometry.

To estimate the stacking contribution to *E*_int_, we took the lowest-energy optimized bound complex for each ligand and removed all but the heterocycle of the ligand using AaronTools (see Figure S4). The resulting open valence was capped with hydrogens whose position was optimized at the wB97X-D/def2SVP level of theory with the remaining atoms fixed. *E*_int_(stack) was then calculated as the difference in energy of the truncated complex and the separated tyrosines and heterocycle in the same geometry at the wB97X-D/def2TZVP level of theory.

## Supporting Information Available

Supplementary Figures S1–S3, Tables S1–S5, and Schemes S1–S9; synthetic procedures for **3**, **10**, **11**, and **20**–**24**; methods for protein purification, crystallography, SPR, and QRET; and ^1^H NMR and LC/MS spectra (PDF)
Tabular data file with *K*_D_, Δ*G*, and *E*_int_ values (XLSX)
Tabular data file of IC_50_ values (with SD) for QRET studies (XLSX)
Molecular formula strings (CSV)
Exemplar Gaussian input text file (TXT)

## Accession Codes

PDB codes: 8F78, 8F96, 8F97, 8FBV, 8F98, 8F99, 8F9A, 8F9B, 8F9C, and 8F9D.

## Abbreviations Used

DFT: density functional theory
ESP: electrostatic potential
IMHB: intramolecular hydrogen bond
PDB: Protein Data Bank
SAPT: symmetry-adapted perturbation theory
SD: standard deviation
SPR: surface plasmon resonance
TRL: time-resolved luminescence
QRET: quenching resonance energy transfer
