# Supplementary material for: Systematic Study of Heteroarene Stacking Using a Congeneric Set of Molecular Glues for Procaspase-6
Source: J Med Chem. 2023 Jul 5;66(14):9784–96. doi: 10.1021/acs.jmedchem.3c00590 (PMC10388292; doi:10.1021/acs.jmedchem.3c00590)
Supplement: Supplementary file 1 — jm3c00590_si_003.pdf [file jm3c00590_si_003.pdf]

Supporting Information

A Systematic Study of Heteroarene Stacking Using a Congeneric Set of Molecular Glues for Procaspase-6

Takaya Togo[†], Linh Tram[†], Laura G. Denton[‡], Xochina ElHilali-Pollard[†], Jun Gu[§], Jinglei Jiang[§], Chenglei Liu[§], Yan Zhao[§], Yanlong Zhao[§], Yinzhe Zheng[§], Yunping Zheng[§] Jingjing Yang[§], Panpan Fan[§], Michelle R. Arkin[†], Harri Härmä[#], Deqian Sun[§], Stacie S. Canan[^], Steven E. Wheeler, [‡,*] and Adam R. Renslo[†,*]

[†]Department of Pharmaceutical Chemistry, University of California, San Francisco, 600 16th Street, San Francisco, California 94143, United States

[‡]Department of Chemistry, University of Georgia, Athens, Georgia 30602, United States

[§]Departments of Chemistry and Biology, Viva Biotech, Pu Dong New Area, 201203 Shanghai, China

[#]Department of Chemistry, University of Turku, 20500 Turku, Finland

[^]Departments of Chemistry and Structural Biology, Elgia Therapeutics, La Jolla, CA 92037, United States

corresponding author email

swheele2@uga.edu

adam.renslo@ucsf.edu

Table of Contents

Protein preparation, crystallography, SPR methods..2–4

Supplementary Tables S1-5 5–11

Supplementary Figures 1-3 and Schemes S1-S9..12–17

Synthetic Procedures for compounds **3, 10-11, 20-23, 27, and 31**..18–25

Scans of 1H NMR and LC/MS Spectra..26–103

References..104

Expression and purification of procaspase-6: A cDNA encoding caspase 6 1-293 with the catalytic Cys mutated to Ala (C163A) was cloned into pET-21b vector (Novagen), adding a TEV protease cleavage site, a C-terminal hexahistidine tag, and an Avi tag. The protein was expressed as soluble protein from Escherichia coli using standard protocols [BL21-Gold (DE3) cells (Stratagene), LB (Luria–Bertani) medium and induction at 37 °C to an OD of ~ 0.6 and were subsequently cooled to 16°C before overnight induction with 0.5 mM IPTG]. Cells were harvested by centrifugation, resuspended in 500 mM NaCl, 50 mM Tris-HCl, pH 8.0, 10 % glycerol, 5 mM DTT, and lysed. The supernatant was loaded onto a HisTrap FF column (GE Healthcare), washed with buffer A (200 mM NaCl, 50 mM Tris-HCl, pH8.0, 10% glycerol, 5 mM DTT), 10% buffer B (200 mM NaCl, 50 mM Tris-HCl, pH8.0, 10% glycerol, 5 mM DTT, 300mM imidazole.), and eluted with 100% buffer B.

The hexahistidine tag and Avi tag were removed by incubation with TEV protease (~ 1 mg/10 mg protein, overnight at 4°C) while being dialyzed against 200 mM NaCl, 50 mM Tris-HCl, pH8.0, 10% glycerol, 5 mM DTT. Dialyzed protein sample was passed through a second Ni^{2+}-nitrilotriacetic acid column to remove the tags and the undigested protein. This step was omitted for protein preps used in SPR studies where the Avi tag is used for protein immobilization.

The dialyzed protein was then diluted with 50 mM Tris, pH 8.0, 10% glycerol, 5 mM DTT, loaded onto a 6 mL Resource Q column (GE Healthcare), and eluted in a gradient of 0-50% 50 mM Tris, pH 8.0, 1 M NaCl, 10% glycerol, 5 mM DTT over 30 column volumes. Caspase-6 is typically eluted with ~200 mM NaCl.

The protein was further purified on a size-exclusion Superdex 200 column (GE Healthcare) in a buffer containing 200 mM NaCl, 50 mM Tris, pH 8.0, 5% glycerol, and 5 mM DTT. Fractions were collected, concentrated by ultrafiltration, and assayed for purity by denaturing gel electrophoresis and by mass spectrometry (LCT Premier; Waters). Expression of procaspase-6 by this approach yielded 3-10 mg of pure protein per 1 liter of culture.

Co-crystallization of procaspase-6 with ligands: All crystals were obtained by vapor diffusion in hanging drops at 4°C.

Complexes of procaspase-6 with compounds **1**, **5**, **7**, and **8** were generated by mixing 12mg/ml procaspase-6 with a 5-fold molar excess of compound for 2 hours on ice. The reaction mixture was crystallized by hanging drop method with 1.7 µL drops, and the crystals grew from a drop containing 0.5 µl of the complex (12mg/ml), 1 µl of well solution (0.1 M HEPES pH 7; 4-15% w/v PEG 8000) and 0.2 µl seed at 4°C. Crystals were cryopreserved in well solution plus 25% ethylene glycol. Diffraction data for the different complexes were collected at Diamond I03, APS 21-ID-D, PETRAIII P11, respectively. The data were indexed, integrated and scaled using XDS. The structure was solved by molecular replacement using the Caspase-6 structure as the search model (PDB-ID 2WDP). The initial FoFc electron density maps show unambiguous density bound compound in the active sites. The compound was fit to the density and the model was subjected to iterative cycles of refinement and rebuilding using Refmac5 and Coot (Table S1).

Complexes of procaspase-6 with compounds **3**, **10**, **11**, **19**, **20**, and **21** were generated by mixing 0.5mg/ml procaspase-6 with a 5-fold molar excess of compound for 2 hours on ice, then the reaction mixture was concentrated to 12mg/ml, and crystallized by hanging drop method with 1.7 µL drops. The crystals grew from a drop containing 0.5 µl of the complex (12mg/ml), 1 µl of well solution (0.1 M HEPES pH 7; 4-15% w/v PEG 8000) and 0.2 µl seed at 4°C. Crystals were cryopreserved in well solution plus 25% ethylene glycol. Diffraction data for the different complexes were collected at APS 19-ID-D, Diamond I03, Spring8 BL45XU, and ALS 5.0.2, respectively. The data were indexed, integrated and scaled using XDS. The structure was solved by molecular replacement using the Caspase-6 structure as the search model (PDB-ID 2WDP). The initial FoFc electron density maps show unambiguous density bound compound in the active sites. The compound was fit to the density and the model was subjected to iterative cycles of refinement and rebuilding using Refmac5 and Coot (Table S2).

Surface Plasmon Resonance Methods

All Surface Plasmon Resonance experiments were conducted according to previously recommended procedures.[1] Full-length Avi-tagged procaspase-6 was expressed as described above except that the Avi tag was retained. Proteins were captured to the surface of neutravidin-

coated CM5 sensor chips using a Biacore 8K instrument (GE Healthcare). The CM5 chip was processed sequentially on Channel 1-8 with both reference and active flow cell with EDC/NHS (Amine Coupling Kit, Cytiva, BRI100050; flow rate was 10 µL/min, 420s injection time), neutravidin (Sigma, S4762) in sodium acetate (pH 5.0; 50 µg/ml, flow rate was 10 µL/min, 750s injection), and finally ethanolamine-HCl (Amine Coupling Kit, Cytiva, BRI100050; 10µL/min, 420s injection). Procaspase-6 protein was then captured on active flow cell, 144 seconds capture time, 5 µl/min as flow rate.

All compounds were diluted to 100 mM in 100% DMSO prior to being diluted with suitable top concentration in 1.05 X assay buffer (10 mM HEPES pH 7.4, 150 mM NaCl, 0.5 mM TCEP, 0.01% BSA, 0.05% P20, 5% DMSO). Analytes were run using the following conditions: 20°C analysis temperature, Assay Steps = all set to LMW kinetics; cycle types = LMW kinetics (60s contact time, 120s dissociation time, 30ul/min flow rate, Channel 1-8 with both reference and active flow cell); Flow cell detection = active flow cell – reference flow cell). Data evaluation was performed using the Biacore 8K Evaluation software and data fit to 1:1 binding model.

Solution Phase Time-Resolved Luminescence by QRET Methods

Procaspase-6 was studied in solution with ligands **1**, **6**, **15**, and **28** at a concentration range of 0.015 to 100 µM and for the much weaker-binding ligand **12** from 0.460 to 3000 µM to determine IC_{50} values in a 384 microtiter plate format. Ligands (5 µL) in 10 mM HEPES (pH 7.5) supplemented with 0.001% Triton X-100 were first added to wells followed by the procaspase-6 enzyme (5 µL, 1000 nM) in the same HEPES/Triton buffer and finally the detection solution (5 µL) containing 3 nM of Eu-chelate labeled ligand **32** and 6 µM of soluble quencher reagent MT2 (QRET Technologies, Turku, Finland) in the HEPES buffer containing 0.03% Triton X-100. The reaction components were incubated for 15 min and time-resolved luminescence (TRL) was measured at 340 nm excitation and 615 nm emission wavelengths (800 µs delay and 400 µs decay) with the Spark 20M plate reader from Tecan Life Sciences (Männedorf, Switzerland).

Supplementary Tables

Table S1. Data collection and refinement statistics.

	**8F78** (procaspase-6 + compound **1**)	**8F96** (procaspase-6 + compound **3**)	**8F97** (procaspase-6 + compound **5**)	**8FBV** (procaspase-6 + compound **7**)	**8F98** (procaspase-6 + compound **8**)
Wavelength	0.97628	1.000000	1.03320	1.12713	1.03320
Resolution range	46.08 - 2.62 (2.714 - 2.62)	45.81 - 2.95 (3.056 - 2.95)	46.33 - 2.32 (2.403 - 2.32)	40.69 - 2.864 (2.966 - 2.864)	45.97 - 2.7 (2.797 - 2.7)
Space group	P 61	P 61	P 61	P 61	P 61
Unit cell	102.094 102.094 321.123 90 90 120	101.333 101.333 321.538 90 90 120	102.66 102.66 322.767 90 90 120	101.57 101.57 321.26 90 90 120	101.762 101.762 321.897 90 90 120
Total reflections	105116 (7888)	76880 (7732)	164599 (16477)	402198(2895)	102366 (10235)
Unique reflections	52658 (5255)	39064 (3902)	82746 (8265)	26336 (141)	51504 (5129)
Multiplicity	2.0 (2.0)	2.0 (2.0)	2.0 (2.0)	7.6(5.1)	2.0 (2.0)
Completeness (%)	99.01 (93.67)	99.68 (99.72)	99.87 (99.82)	61.22 (3.31)	99.85 (99.90)
Mean I/sigma(I)	27.31 (2.54)	5.51 (1.90)	21.83 (2.00)	11.9(3.3)	17.56 (2.19)
Wilson B-factor	58.02	71.80	60.77	39.65	70.35
R-merge	0.01454 (0.1953)	0.09847 (0.3697)	0.0229 (0.3213)	0.188(0.653)	0.03492 (0.3616)
R-meas	0.02056 (0.2761)	0.1393 (0.5228)	0.03238 (0.4544)	0.202(0.727)	0.04939 (0.5114)
R-pim	0.01454 (0.1953)	0.09847 (0.3697)	0.0229 (0.3213)	0.073(0.315)	0.03492 (0.3616)
CC1/2	1 (0.961)	0.978 (0.911)	0.999 (0.937)	0.998(0.950)	0.999 (0.917)
CC*	1 (0.99)	0.994 (0.976)	1 (0.983)		1 (0.978)
Reflections used in refinement	56019 (5257)	39020 (3904)	82684 (8264)	26299 (142)	51452 (5133)

Reflections used for R-free	2912 (328)	2040 (223)	4031 (391)	1329 (2)	2559 (284)
R-work	0.1868 (0.2473)	0.2055 (0.3528)	0.1839 (0.3412)	0.1980 (0.2922)	0.1977 (0.3478)
R-free	0.2139 (0.3245)	0.2273 (0.3392)	0.2062 (0.3272)	0.2350 (0.2949)	0.2205 (0.3647)
CC(work)	0.860 (0.510)	0.865 (0.420)	0.884 (0.426)		0.873 (0.409)
CC(free)	0.878 (0.364)	0.909 (0.430)	0.902 (0.402)		0.882 (0.453)
Number of non-hydrogen atoms	8257	8091	8603	8102	8217
macromolecules	8027	7907	8038	7925	8001
ligands	92	88	130	84	84
solvent	138	96	435	93	132
Protein residues	996	984	996	986	994
RMS(bonds)	0.228	0.173	0.214	0.202	0.197
RMS(angles)	4.60	4.84	5.53	5.09	5.23
Ramachandran favored (%)	94.18	94.73	96.12	91.44	95.81
Ramachandran allowed (%)	5.61	5.17	3.67	8.45	4.19
Ramachandran outliers (%)	0.20	0.10	0.20	0.10	0.00
Rotamer outliers (%)	1.72	3.86	2.41	7.46	3.93
Clashscore	5.06	3.55	5.46	6.71	5.90
Average B-factor	60.69	75.70	68.78	42.55	78.11
macromolecules	61.10	75.71	68.73	42.62	78.26

ligands	58.04	102.41	85.91	61.31	90.69
solvent	38.88	50.08	64.49	19.40	60.75

Table S2. Data collection and refinement statistics

	**8F99** (procaspase-6 + compound **10**)	**8F9A** (procaspase-6 + compound **11**)	**8F9B** (procaspase-6 + compound **19**)	**8F9C** (procaspase-6 + compound **20**)	**8F9D** (procaspase-6 + compound **21**)
Wavelength	1.000050	0.97625	0.97625	1.03316	0.979180
Resolution range	45.86 - 2.86 (2.962 - 2.86)	44.05 - 2.55 (2.641 - 2.55)	44.14 - 2.65 (2.745 - 2.65)	45.85 - 2.8 (2.9 - 2.8)	43.96 - 2.65 (2.745 - 2.65)
Space group	P 61	P 61	P 61	P 61	P 61
Unit cell	101.481 101.481 321.539 90 90 120	101.73 101.73 321.6 90 90 120	101.94 101.94 321.98 90 90 120	101.423 101.423 321.949 90 90 120	101.529 101.529 321.926 90 90 120
Total reflections	85589 (8541)	120973 (12063)	108652 (10928)	91115 (9126)	107763 (10894)
Unique reflections	43079 (4289)	60981 (6060)	54664 (5486)	45854 (4582)	54216 (5469)
Multiplicity	2.0 (2.0)	2.0 (2.0)	2.0 (2.0)	2.0 (2.0)	2.0 (2.0)
Completeness (%)	99.86 (99.98)	99.87 (99.92)	99.87 (99.98)	99.80 (99.70)	99.84 (99.96)
Mean I/sigma(I)	10.58 (1.87)	12.90 (2.11)	15.43 (2.40)	10.91 (2.02)	15.21 (1.85)
Wilson B-factor	63.15	65.38	69.70	71.79	67.74
R-merge	0.06295 (0.4457)	0.03746 (0.3542)	0.03211 (0.2958)	0.04527 (0.3077)	0.03769 (0.4369)
R-meas	0.08902 (0.6304)	0.05298 (0.5009)	0.04541 (0.4183)	0.06402 (0.4352)	0.0533 (0.6179)
R-pim	0.06295 (0.4457)	0.03746 (0.3542)	0.03211 (0.2958)	0.04527 (0.3077)	0.03769 (0.4369)
CC1/2	0.997 (0.837)	0.999 (0.927)	0.999 (0.941)	0.998 (0.983)	0.999 (0.889)
CC*	0.999 (0.955)	1 (0.981)	1 (0.985)	0.999 (0.996)	1 (0.97)

Reflections used in refinement	43032 (4289)	60948 (6062)	54627 (5487)	45808 (4584)	54176 (5473)
Reflections used for R-free	2205 (219)	3098 (287)	2643 (269)	2329 (207)	2766 (279)
R-work	0.2079 (0.3513)	0.1996 (0.3830)	0.2007 (0.3915)	0.2055 (0.3784)	0.2038 (0.4046)
R-free	0.2434 (0.4107)	0.2369 (0.4182)	0.2288 (0.4107)	0.2300 (0.4027)	0.2275 (0.3931)
CC(work)	0.882 (0.509)	0.868 (0.398)	0.864 (0.403)	0.841 (0.348)	0.851 (0.392)
CC(free)	0.891 (0.342)	0.810 (0.260)	0.875 (0.459)	0.841 (0.143)	0.878 (0.471)
Number of non-hydrogen atoms	8143	8360	8291	8137	8283
macromolecules	7925	7997	7997	7878	7984
ligands	84	84	84	84	111
solvent	134	279	210	175	188
Protein residues	986	992	992	981	991
RMS(bonds)	0.163	0.167	0.174	0.167	0.197
RMS(angles)	4.95	4.75	4.82	4.29	4.07
Ramachandran favored (%)	95.57	94.06	94.47	95.13	95.28
Ramachandran allowed (%)	4.33	5.94	5.23	4.77	4.72
Ramachandran outliers (%)	0.10	0.00	0.31	0.10	0.00
Rotamer outliers (%)	3.50	5.31	4.61	2.93	3.58
Clashscore	4.05	6.02	5.14	4.26	3.56
Average B-factor	69.40	72.11	74.04	77.71	77.22
macromolecules	69.64	72.21	74.17	78.04	77.25

ligands	83.41	94.76	92.71	89.55	104.18
solvent	46.78	62.43	61.87	57.42	59.97

Table S3. Root mean square deviation (RMSD) calculated for the aligned co-crystal structures as compared to the co-complex structure of compound **1**.

Compound	RMSD (Å)
**1**	-
**3**	0.2833
**5**	0.2829
**7**	0.2421
**8**	0.2156
**10**	0.1907
**11**	0.2736
**19**	0.2770
**20**	0.2976
**21**	0.1900

Table S4. Absolute energies for the lowest-energy optimized complex [E(Complex)] as well as the Tyrs in the complex geometry [E(Tyrs)], relaxed ligand, trunated complex, and truncated ligand in hartrees as well as the corresponding interaction energies (E_{int}) and stacking contributions to E_{int} [E_{int}(Stack)] in kcal/mol.

Ligand	E(Complex)	E(Tyrs)	Relaxed Ligand	Truncated Complex	Truncated Lignad	E_{int}	E_{int}(Stack)
**1**	-1783.953273	-772.243335	-1011.674471	-1036.593985	-264.333733	-22.3	-10.6
**2**	-1783.918734	-772.244178	-1011.638636	-1036.560625	-264.298831	-22.5	-11.1
**3**	-1783.953925	-772.243828	-1011.674349	-1036.594719	-264.333558	-22.4	-10.9
**4**	-1783.944421	-772.243952	-1011.664879	-1036.586428	-264.325267	-22.3	-10.8
**5**	-1767.907797	-772.244062	-995.628701	-1020.549946	-248.288948	-22.0	-10.6
**6**	-1823.276030	-772.243382	-1050.995016	-1075.916667	-303.654351	-23.6	-11.9
**7**	-1745.861670	-772.244441	-973.583644	-998.498876	-226.240788	-21.1	-8.6
**8**	-1745.859964	-772.243746	-973.582250	-998.499161	-226.240927	-21.3	-9.1
**9**	-1745.837896	-772.243845	-973.561584	-998.477107	-226.219675	-20.4	-8.5
**10**	-1765.706711	-772.243781	-993.427854	-1018.348052	-246.088551	-22.0	-9.9
**11**	-1765.668209	-772.243843	-993.391605	-1018.308780	-246.050713	-20.6	-8.9
**12**	-1785.144143	-772.244553	-1012.869149	-1037.789341	-265.528129	-19.1	-10.5
**13**	-2088.681956	-772.244421	-1316.406641	-1341.327771	-569.069552	-19.4	-8.7
**14**	-2088.685955	-772.243834	-1316.408729	-1341.328328	-569.069546	-21.0	-9.4
**15**	-1785.149599	-772.243235	-1012.871962	-1037.788301	-265.529400	-21.6	-9.8
**16**	-2088.679720	-772.244447	-1316.403350	-1341.323531	-569.065502	-20.0	-8.5
**17**	-2088.687057	-772.243241	-1316.410525	-1341.327101	-569.069431	-20.9	-9.1
**18**	-1785.170928	-772.243958	-1012.890638	-1037.810169	-265.548779	-22.8	-10.9
**19**	-2088.685196	-772.244301	-1316.407139	-1341.325523	-569.065710	-21.2	-9.7
**20a**	-1761.896957	-772.243529	-989.621021	-1014.536443	-242.279113	-20.3	-8.7
**20b**	-1761.897569	-772.243819	-989.620658	-1014.536742	-242.279060	-20.5	-8.7
**20c**	-1761.896045	-772.244533	-989.617047	-1014.533355	-242.274202	-19.1	-9.2
**21**	-1781.727268	-772.243553	-1009.450405	-1034.367314	-262.109005	-20.9	-9.3
**22a**	-1761.868983	-772.243840	-989.591482	-1014.508964	-242.250799	-20.1	-9.0
**22b**	-1761.869777	-772.243734	-989.593115	-1014.509868	-242.252924	-20.7	-8.3
**23a**	-1777.904499	-772.243751	-1005.627236	-1030.543677	-258.286257	-21.0	-8.6
**23b**	-1777.900975	-772.243631	-1005.625621	-1030.541418	-258.285122	-18.9	-7.9
**24**	-1801.182651	-772.244003	-1028.902697	-1053.822656	-281.561570	-22.6	-10.7
**25**	-1799.957083	-772.243443	-1027.678133	-1052.596965	-280.336470	-22.3	-10.7
**26**	-2072.632398	-772.244860	-1300.357609	-1325.277705	-553.019927	-18.8	-8.1
**27**	-1749.651168	-772.244359	-977.372734	-1002.289121	-230.031570	-21.4	-8.3
**28**	-1899.488888	-772.243585	-1127.205888	-1152.127741	-379.863891	-24.7	-12.7
**29**	-2242.341460	-772.243111	-1470.060307	-1494.980037	-722.717627	-23.9	-12.1
**30**	-1899.513463	-772.243453	-1127.232184	-1152.150855	-379.888504	-23.7	-11.9

Table S5. SAPT0/jun-cc-pVDZ decomposition of interaction energies [E(SAPT0)] into contributions from electrostatic (Elec), exchange-repulsion (Exch), induction (Ind), and dispersion (Disp), in kcal/mol. R^2 is the correlation coefficient of each component with E(SAPT0), while "not R^2" is the correlation coefficient of all components except the one listed with E(SAPT0).

Inhib	Elec	Exch	Ind	Disp	E(SAPT0)
**1**	-7.6	20.1	-2.1	-18.4	-7.9
**2**	-10.3	20.2	-2.6	-18.9	-11.5
**3**	-8.0	18.6	-2.1	-18.0	-9.5
**4**	-6.2	18.5	-1.9	-18.3	-7.9
**5**	-7.0	18.6	-2.1	-18.3	-8.8
**6**	-7.1	21.6	-2.3	-20.4	-8.1
**7**	-6.8	15.3	-2.4	-13.5	-7.4
**8**	-6.1	16.5	-2.0	-15.6	-7.2
**9**	-4.1	14.8	-1.6	-14.7	-5.6
**10**	-6.7	18.0	-1.9	-15.9	-6.5
**11**	-8.0	16.6	-1.8	-14.8	-8.0
**12**	-3.8	17.4	-1.9	-17.8	-6.1
**13**	-8.1	18.2	-1.8	-16.8	-8.4
**14**	-6.2	16.7	-1.7	-16.6	-7.7
**15**	-4.3	17.9	-2.1	-17.7	-6.2
**16**	-7.6	14.6	-1.8	-14.0	-8.8
**17**	-8.1	20.4	-2.1	-18.1	-7.9
**18**	-3.8	18.5	-2.4	-18.0	-5.7
**19**	-7.7	16.8	-2.0	-16.6	-9.6
**20a**	-6.7	15.7	-1.7	-15.1	-7.8
**20b**	-8.5	16.4	-2.2	-14.8	-9.1
**20c**	-6.8	15.1	-2.6	-13.3	-7.5
**21**	-7.5	16.5	-1.8	-14.7	-7.6
**22a**	-8.6	17.0	-2.2	-15.7	-9.5
**22b**	-5.0	15.0	-1.4	-14.8	-6.1
**23a**	-9.1	16.1	-2.7	-14.6	-10.3
**23b**	-7.7	15.9	-1.6	-14.9	-8.4
**24**	-5.1	18.4	-2.5	-17.9	-7.1
**25**	-7.1	19.5	-2.2	-18.2	-8.0
**26**	-5.8	17.2	-1.7	-16.8	-7.1
**27**	-4.3	13.5	-1.6	-12.4	-4.7
**28**	-8.6	23.0	-2.9	-22.6	-11.1
**29**	-11.4	24.6	-2.9	-23.6	-13.3
**30**	-11.2	21.8	-3.2	-21.0	-13.7
**R^2**	0.86	0.39	0.52	0.34	
**not R^2**	0.09	0.77	0.97	0.06	

Supplementary Figures

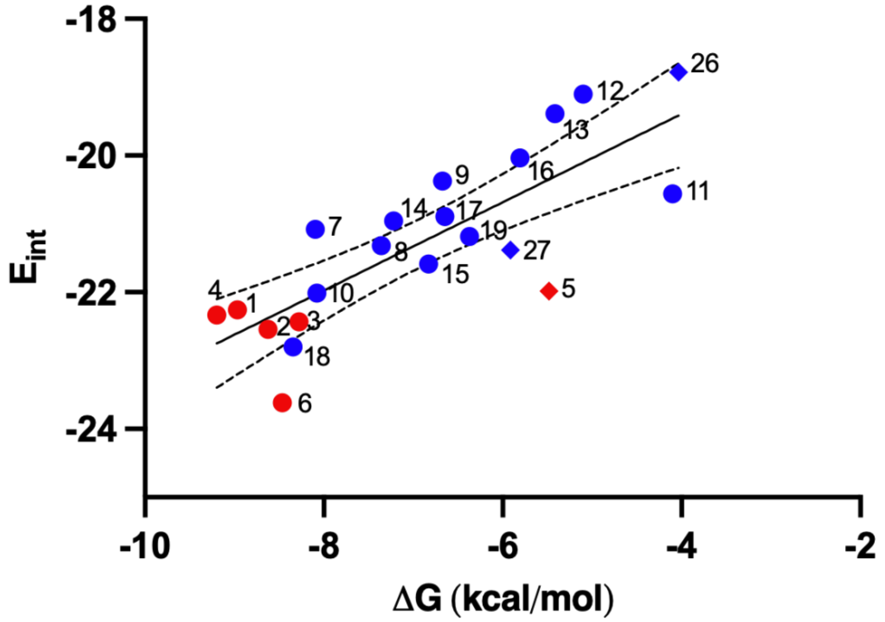

Figure S1. Correlation of experimental and computed binding energies for five or six-membered heteroarenes bearing one or two heteroatoms ($R^2 = 0.63$; 95% confidence intervals in dotted lines). Data points are labelled with compound number, colored according to the ring size (red for six-membered, blue for five-membered) and shaped number of heteroatoms (diamond = one; circle = two).

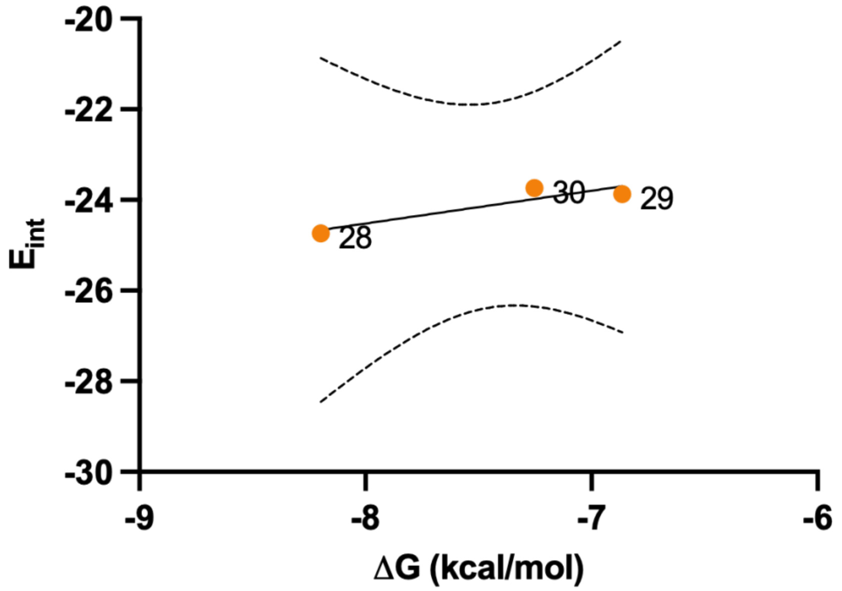

Figure S2. Correlation of experimental and computed binding energies for 5/6-bicyclic heteroarene analogs **28-30** (R^2 = 0.84; 95% confidence intervals in dotted lines).

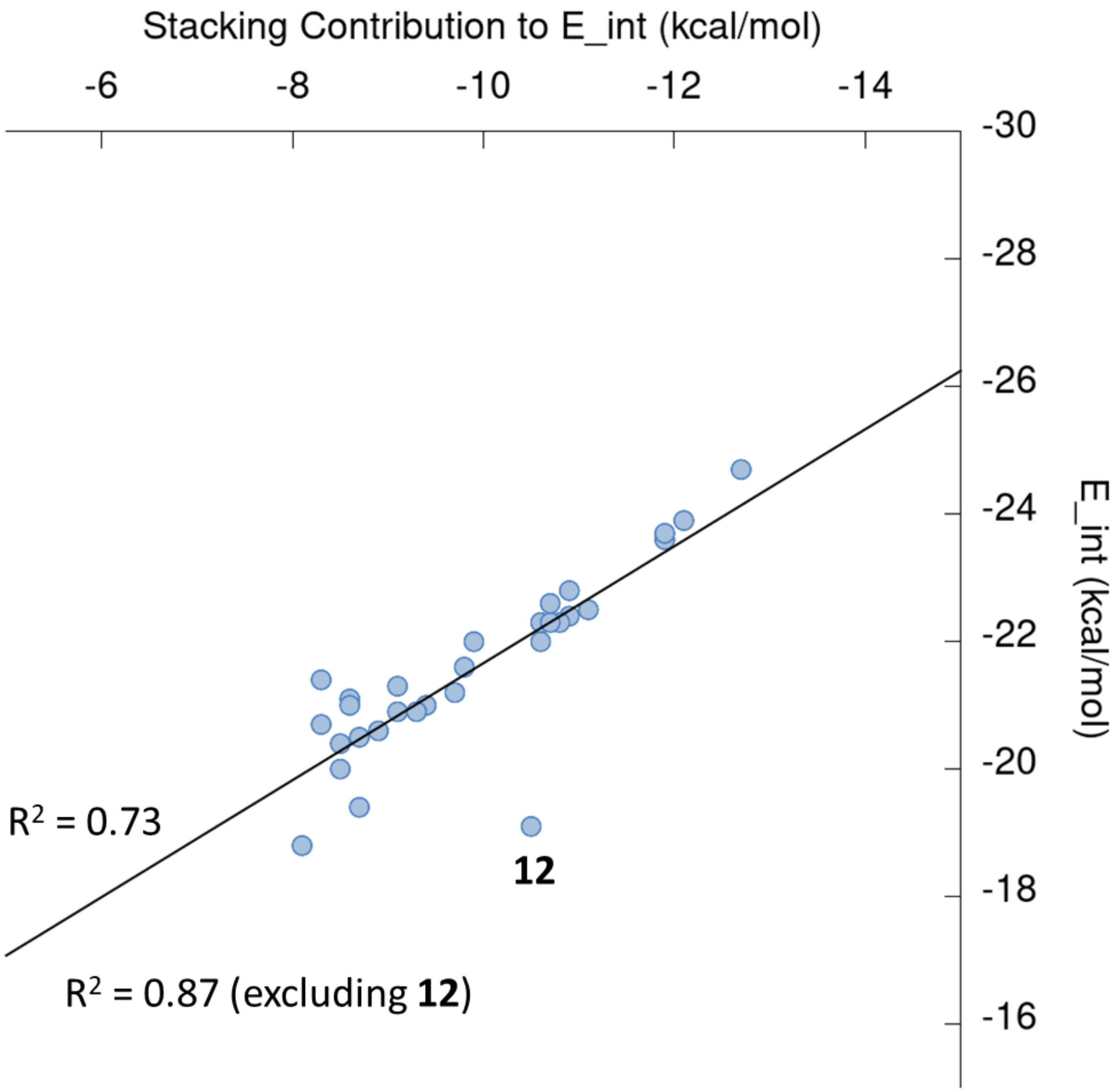

Figure S3. Correlation of E_{int} (E_int) with the stacking contribution to E_{int}.

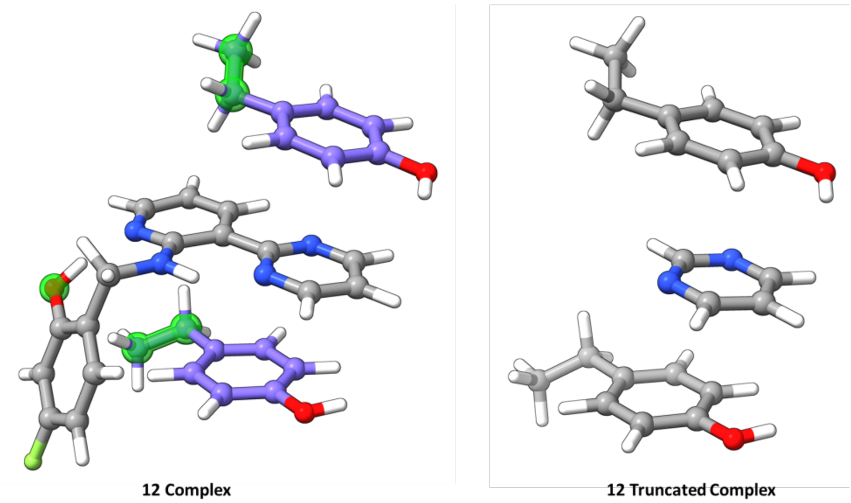

Figure S4. Example demonstrating truncation of complex for computations demonstrating only the contribution of the stacking interaction in the ligand interaction.

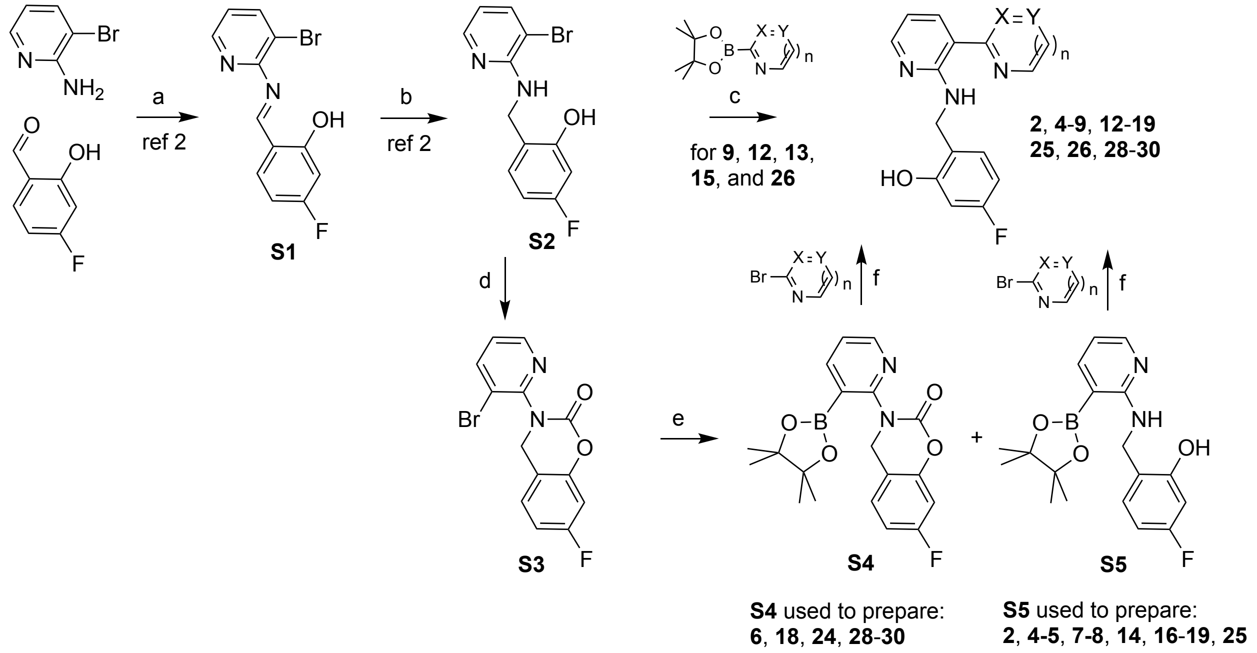

Scheme S1. General synthetic approach to compounds **2**, **4-9**, **12-20**, **25**, **26**, and **28-30** via intermediates **S1-S5** and based on Suzuki-Miyaura coupling reactions to introduce the probe heteroarene. Conditions: (a) (±)-camphor sulfonic acid, toluene, reflux; (b) $NaBH_4$, THF, r.t.; (c) $Pd(dppf)Cl_2$, K_2CO_3, dioxane, H_2O; (d) triphosgene, CH_3CN, 80 °C; (e) B_2Pin_2, $Pd(dppf)Cl_2$, KOAc, dioxane, 85 °C; (f) $Pd(dppf)Cl_2$, K_2CO_3, dioxane, H_2O, 100 °C.

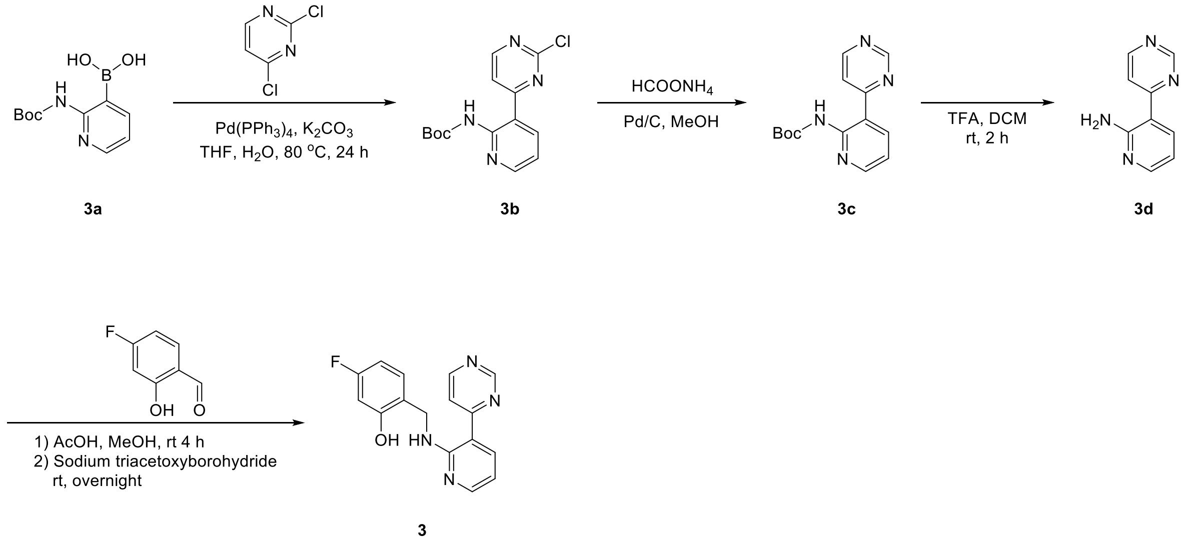

Scheme S2. Synthesis of compound **3**.

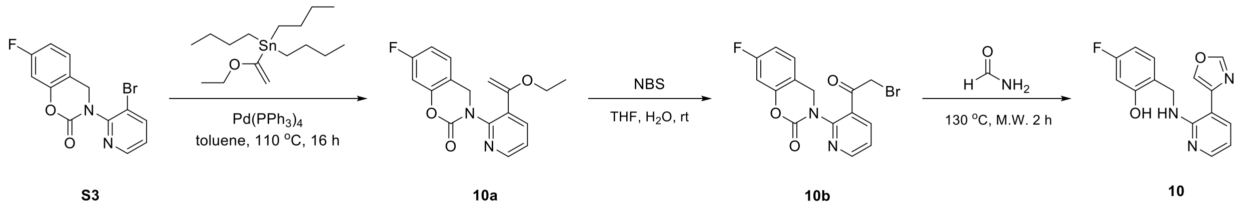

Scheme S3. Synthesis of compound **10**.

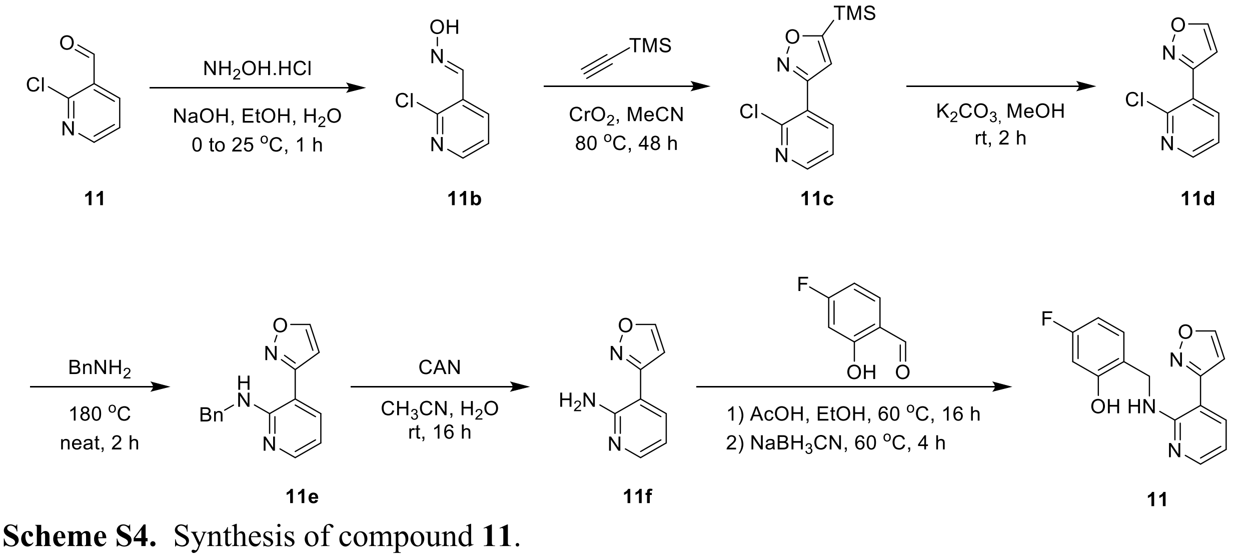

Scheme S4. Synthesis of compound **11**.

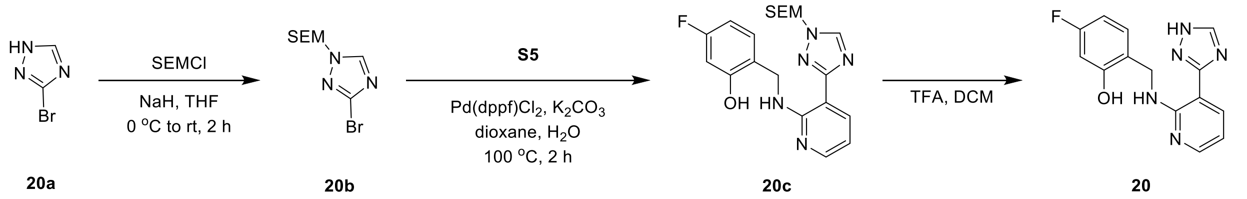

Scheme S5. Synthesis of compound **20**.

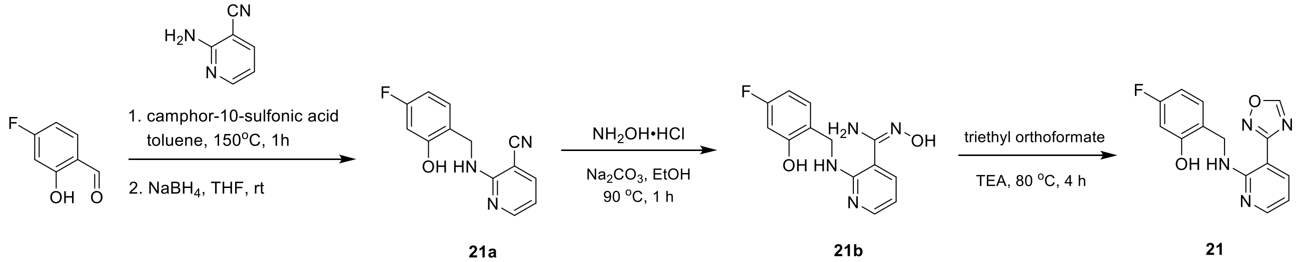

Scheme S6. Synthesis of compound **21**.

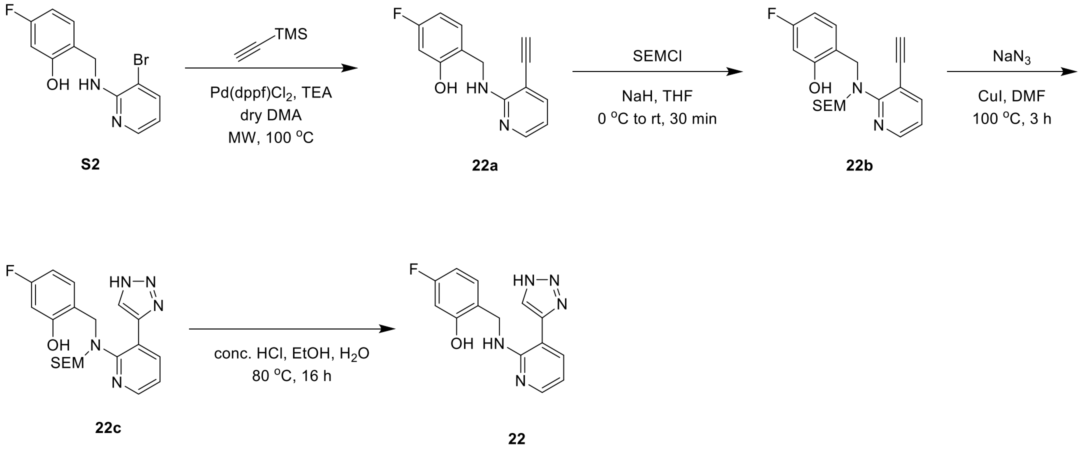

Scheme S7. Synthesis of compound **22**.

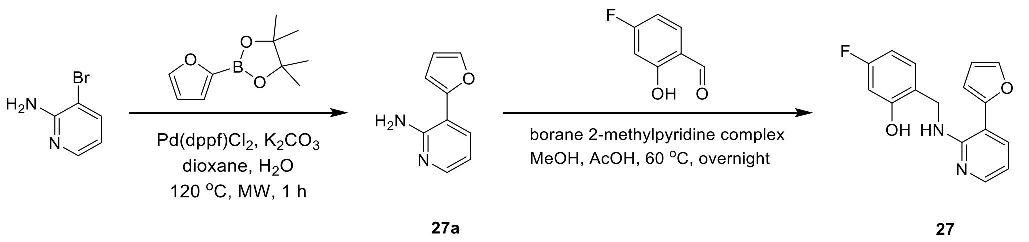

Scheme S8. Synthesis of compound **27**.

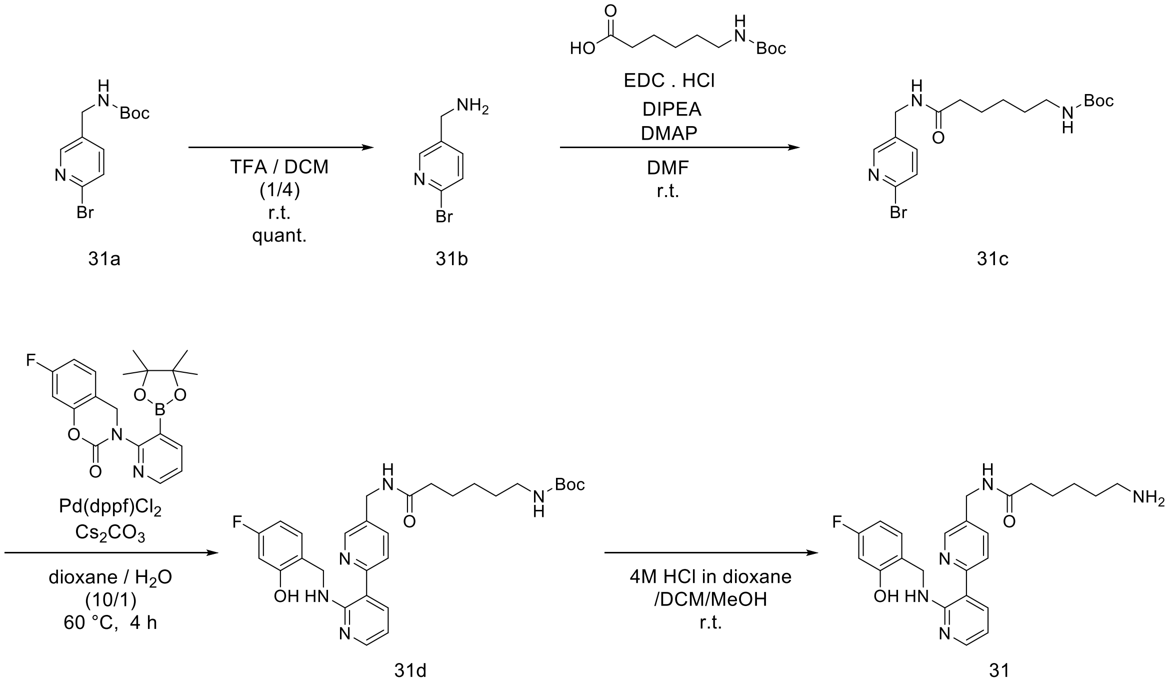

Scheme S9. Synthesis of compound **31**.

Synthetic Procedures for compounds 3, 10-11, 20-23, 27, and 31.

Synthesis of 5-fluoro-2-(((3-(pyrimidin-4-yl)pyridin-2-yl)amino)methyl)phenol (3).

Step 1: To a solution of **3a** (3.10 g, 13.03 mmol), 2,4-dichloropyrimidine (1.51 g, 10.07 mmol) and K_2CO_3 (3.46 g, 25.07 mmol) in THF (80 mL) and water (40 mL) was added $Pd(PPh_3)_4$ (0.75 g, 1.03 mmol) under nitrogen atmosphere. The reaction was stirred at 85 °C for 2 hours. The mixture was then treated with water (30 mL) and extracted with EtOAc (80 mL). The organic layers were combined and washed with brine (20 mL), dried over anhydrous Na_2SO_4, filtered and concentrated *in vacuo*. The crude product was purified by silica gel chromatography using a mixture of petroleum ether-ethyl acetate (1:10 v/v) to afford **3b** (900 mg, yield: 29.4%). ^{1}H NMR (400 MHz, $CDCl_3$) δ 10.32 (s, 1H), 8.70 (d, *J* = 5.4 Hz, 1H), 8.60 (d, *J* = 3.4 Hz, 1H), 8.06 (dd, *J* = 7.9, 1.7 Hz, 1H), 7.64 (d, *J* = 5.4 Hz, 1H), 7.16-7.12 (m, 1H), 1.51 (s, 9H).

Step 2: A mixture of **3b** (840 mg, 2.75 mmol), ammonium formate (1.74 g, 27.59 mmol) and Pd/C (800 mg) in MeOH (80 mL) was stirred at room temperature overnight. The mixture was filtered

through silica gel, washiing with EtOAc (90 mL). The filtrate was concentrated and purified by silica gel chromatography using a mixture of petroleum ether-ethyl acetate (1:10 v/v) as eluent to afford **3c** which was used without further purification (533 mg, yield: 71.3%).^{1}H NMR (400 MHz, $CDCl_3$) δ 11.14 (s, 1H), 9.32 (s, 1H), 8.86 (d, *J* = 4 Hz, 1H), 8.63 (d, *J* = 2 Hz, 1H), 8.09 (d, *J* = 4 Hz, 1H), 7.73 (d, *J* = 2 Hz, 1H), 7.13 (dd, *J* = 4, 2 Hz, 1H).

Step 3: A solution of **3c** (533 mg, 1.96 mmol) and TFA (6 mL) in CH_2Cl_2 (6 mL) was stirred at room temperature for 1h. The mixture was concentrated to give 533 mg crude product **3d** which was used without purification. ^{1}H NMR (400 MHz, $CDCl_3$) δ 9.22 (d, *J* = 1.2 Hz, 1H), 8.76 (d, *J* = 5.6 Hz, 1H), 8.20 (dd, *J* = 4.8, 1.7 Hz, 1H), 7.99 (dd, *J* = 7.8, 1.7 Hz, 1H), 7.76 – 7.67 (m, 1H), 7.11 (dd, *J* = 7.8, 4.8 Hz, 2H), 6.74 (dd, *J* = 7.8, 4.8 Hz, 1H).

Step 4: To a solution of **3d** (350 mg, 2.03 mmol), 4-fluoro-2-hydroxybenzaldehyde (1.4 g, 9.99 mmol) in MeOH (40 mL) was added AcOH (0.1 mL). The mixture was stirred at room temperature for 4 hours. Then, sodium triacetoxyborohydride (2.97 g, 14.01 mmol) was added at 0 ^{o}C. The reaction mixture was stirred at room temperature overnight. Water (3.0 mL) was then added, the mixture was concentrated to remove MeOH and the resulting aqueous solution extracted with CH_2Cl_2 (2×50 mL). The combined organic layer was dried over anhydrous Na_2SO_4, filtered and concentrated *in vacuo*. The crude product was purified by silica gel chromatography using a mixture of petroleum ether-ethyl acetate (1/1 v/v) as eluent to afford **3** (160 mg, 27%) as a white solid. ^{1}H NMR (400 MHz, DMSO-*d*$_6$) δ 10.56 (br s, 1H), 9.73 (t, *J* = 5.6 Hz, 1H), 9.21 (s, 1H), 8.84 (d, *J* = 5.6 Hz, 1H), 8.29 (dd, *J* = 7.6, 2.0 Hz, 1H), 8.22 (dd, *J* = 4.8, 1.6 Hz, 1H), 8.11 (dd, *J* = 5.6, 2.0 Hz, 1H), 7.22 (dd, *J* = 8.4, 7.2 Hz, 1H), 6.74-6.71 (m, 1H), 6.62-6.53 (m, 2H), 4.62 (d, *J* = 6.0 Hz, 2H). ^{13}C NMR (101 MHz, DMSO-*d*$_6$) δ 163.4, 162.3 (d, *J* = 242.4 Hz), 157.8, 157.5, 157.2 (d, *J* = 11.1 Hz), 156.8, 151.2, 138.4, 130.5 (d, *J* = 10.3 Hz), 123.0 (d, *J* = 2.8 Hz), 118.3, 112.6, 112.2, 105.6 (d, *J* = 21.2 Hz), 103.0 (d, *J* = 24.2 Hz), 39.9. MS Calcd for $C_{16}H_{13}FN_4O$: 296.11; MS Found: 297.2 $[M+H]^+$.

Synthesis of 5-fluoro-2-(((3-(oxazol-4-yl)pyridin-2-yl)amino)methyl)phenol (10).

Step 1: To a solution of **S3** (10 g, 30.95 mmol) and tributyl(1-ethoxyvinyl)stannane (10.2 g, 28.1 mmol) in toluene (80 mL) and was added $Pd(PPh_3)_4$ (3.6 g, 3.10 mmol) under a nitrogen atmosphere. The reaction mixture was stirred at 110 ^{o}C for 16 hours and then cooled to ambient temperature. Saturated KF solution (100 mL) was then added and the mixture was stirred at room temperature overnight and extracted with EtOAc (200 mL). The organic layer was washed with brine (30 mL), dried over anhydrous Na_2SO_4, filtered and concentrated in *vacuo*. The crude product was purified by silica gel chromatography using a mixture of petroleum ether-ethyl acetate (1:1 v/v) as eluent to afford **10a** (10 g, yield: 91%). ^{1}H NMR (400 MHz, DMSO-*d*$_6$) δ 8.53 (dd, *J* = 4.8, 1.8 Hz, 1H), 7.97 (dd, *J* = 7.7, 1.8 Hz, 1H), 7.49-7.38 (m, 2H), 7.18 – 7.03 (m, 2H), 4.45 (d, *J* = 2.7 Hz, 1H), 4.35 (d, *J* = 2.7 Hz, 1H), 3.74-3.64 (m, 2H), 0.94 (t, *J* = 7.0 Hz, 3H).

Step 2: To a solution of **10a** (4.5 g, 14.33 mmol) in dioxane/H_2O (50 mL/15 mL) was added N-bromosuccinimide (3.06 g, 17.2 mmol) at 0 ^{o}C. The mixture was stirred at room temperature for 2 hours. The mixture was extracted with EtOAc (90 mL) and the combined organic layer was washed with brine (30 mL), dried over anhydrous Na_2SO_4, filtered and concentrated in *vacuo*. The crude product was purified by silica gel chromatography using a mixture of petroleum ether-ethyl acetate

(3:1 v/v) as eluent to afford **10b**. A mixture of **10b** (400 mg, 1.10 mmol) and formamide (10 mL) was heated at 130 °C in a microwave reactor for 4 hours under an inert atmosphere. The reaction mixture was then cooled, and extracted with EtOAc (60 mL). The organic layer was washed with brine (30 mL), dried over anhydrous Na_2SO_4, filtered and concentrated in *vacuo*. The crude product was purified by silica gel chromatography using a mixture of petroleum ether-ethyl acetate (5:1 v/v) as eluent to afford compound **10** (33 mg, yield: 10%) as a white solid. ^{1}H NMR (400 MHz, DMSO-d_6) δ 10.75 (br s, 1H), 8.66 (s, 1H), 8.62 (s, 1H), 8.04 (dd, *J* = 5.2, 2.0 Hz, 1H), 7.87-7.84 (m, 2H), 7.23 (t, *J* = 8.0Hz, 1H), 6.67-6.64 (m, 1H), 6.61-6.53 (m, 2H), 4.52 (d, *J* = 6.0 Hz, 2H). ^{13}C NMR (101 MHz, DMSO-d_6) δ 162.4 (d, *J* = 242.2 Hz), 157.4 (d, *J* = 11.7 Hz), 155.8, 152.6, 147.4, 137.4, 136.7, 135.7, 131.0 (d, *J* = 10.3 Hz), 123.2 (d, *J* = 2.1 Hz), 112.3, 109.9, 105.7 (d, *J* = 21.2 Hz), 103.1 (d, *J* = 23.7 Hz), 40.0. MS Calcd for $C_{15}H_{12}FN_3O_2$: 285.09; MS Found: 286.2 $[M+H]^+$.

Synthesis of 5-fluoro-2-(((3-(isoxazol-3-yl)pyridin-2-yl)amino)methyl)phenol (11).

Step 1: To a solution of **11a** (10.0 g, 71 mmol) and hydroxylamine hydrochloride (5.49 g, 78 mmol) in EtOH/H_2O (70 mL/70 mL) was added a solution of NaOH (6.8 g, 170 mmol) in H_2O (70 mL) under nitrogen atmosphere at 0 °C. After 10 min, the reaction was allowed to warm to room temperature and stirred for 1 h. The mixture was neutralized to pH = 7 with 6N aqueous HCl and the crude product **11b** (9.7 g, 87% crude) was obtained by filtration, washed with water and dried *in vacuo*. ^{1}H NMR (400 MHz, DMSO-d_6) δ 11.96 (s, 1H), 8.48 – 8.39 (m, 1H), 8.30 (s, 1H), 8.20 (dd, *J* = 7.8, 1.9 Hz, 1H), 7.53 – 7.44 (m, 1H).

Step 2: To a solution of **11b** (5 g, 31.8 mmol) and ethynyltrimethylsilane (31.2 g, 318 mmol) in CH_3CN (100 mL) was added CrO_2 (26.7 g, 318 mmol) under nitrogen atmosphere. The reaction mixture was stirred at 80 °C for 48 h and then cooled to room temperature and filtered. The filtrate was concentrated *in vacuo*, and the crude product was purified by silica gel chromatography using a mixture of petroleum ether-ethyl acetate (95:5 v/v) as eluent to afford **11c** (3.43 g, yield: 43%). ^{1}H NMR (400 MHz, DMSO-d_6) δ 8.56 (dd, *J* = 4.8, 1.9 Hz, 1H), 8.16 (dd, *J* = 7.6, 1.9 Hz, 1H), 7.59 (dd, *J* = 7.6, 4.8 Hz, 1H), 7.22 (s, 1H), 0.38 (s, 9H).

Step 3: To a mixture of **11c** (3.4 g, 13.43 mmol) in MeOH (100 mL) was added K_2CO_3 (5.56 g, 40.3 mmol) under a nitrogen atmosphere. The reaction was stirred for 1 h at room temperature. The mixture was then filtered, and the filtrate was concentrated *in vacuo*. The residue was purified by silica gel chromatography using a mixture of petroleum ether-ethyl acetate (95:5 v/v) as eluent to afford **11d** (1.40 g, yield: 58%). ^{1}H NMR (400 MHz, DMSO-d_6) δ 9.14 (d, *J* = 1.7 Hz, 1H), 8.58 (dd, *J* = 4.8, 1.9 Hz, 1H), 8.18 (dd, *J* = 7.6, 1.9 Hz, 1H), 7.61 (dd, *J* = 7.6, 4.8 Hz, 1H), 7.09 (d, *J* = 1.7 Hz, 1H).

Step 4: A mixture of **11d** (400 mg, 2.2 mmol) and $BnNH_2$ (7.2 mL) was stirred at 180 °C for 2 h under nitrogen atmosphere in a sealed tube. After cooling, EtOAc (200 mL) was added and the solution concentrated under reduced pressure. The residue was purified by silica gel chromatography using a mixture of petroleum ether-ethyl acetate (92:8 v/v) as eluent to afford **11e**

(500 mg, yield: 68%). ^{1}H NMR (400 MHz, DMSO-d_6) δ 9.04 (d, *J* = 1.8 Hz, 1H), 8.19 (dd, *J* = 4.8, 1.8 Hz, 1H), 8.11 (dd, *J* = 7.6, 1.8 Hz, 1H), 7.87 (t, *J* = 5.6 Hz, 1H), 7.40 – 7.28 (m, 5H), 7.27-7.20 (m, 1H), 6.75 (dd, *J* = 7.6, 4.8 Hz, 1H), 4.77 (d, *J* = 5.7 Hz, 2H).

Step 5: To a solution of **11e** (1 g, 3.97 mmol) in CH_3CN (30 mL) and H_2O (6 mL) was added diammonium cerium(IV) nitrate (4.34 g, 7.94 mmol) at 0 °C under a nitrogen atmosphere. The reaction mixture was stirred at room temperature for 16 h and quenched with aqueous Na_2CO_3. The mixture was extracted with EtOAc (3×50 mL). The combined organic layer was washed with brine (50 mL), dried over anhydrous Na_2SO_4, filtered and concentrated *in vacuo*. The crude product was purified by silica gel chromatography using a mixture of petroleum ether-ethyl acetate (75:25 v/v) as eluent to afford **11f** (590 mg, yield: 92%). ^{1}H NMR (400 MHz, DMSO-d_6) δ 9.02 (d, *J* = 1.8 Hz, 1H), 8.11 (dd, *J* = 4.8, 1.8 Hz, 1H), 8.05 (dd, *J* = 7.7, 1.8 Hz, 1H), 7.27 (d, *J* = 1.8 Hz, 1H), 6.92 (s, 2H), 6.72 (dd, *J* = 7.6, 4.8 Hz, 1H).

Step 6: To a solution of **11f** (200 mg, 1.24 mmol) and 4-fluoro-2-hydroxybenzaldehyde (1.04 g, 7.45 mmol) in EtOH (20 mL) was added AcOH (2 drops) under nitrogen atmosphere. The mixture was stirred at 60 °C for 16 h. Subsequently, the mixture was cooled to 0 °C, and $NaBH_3CN$ (547 mg, 8.68 mmol) was added. After stirring for 10 min, the reaction was heated to 60 °C and stirred for 4 h. After cooling and addition of aqueous K_2CO_3 (15 mL), the mixture was extracted with EtOAc by three times. The organic layer was washed with brine (20 mL), dried over anhydrous Na_2SO_4, filtered and concentrated *in vacuo*. The crude product was purified by silica gel chromatography using a mixture of petroleum ether-ethyl acetate (92:8 v/v) followed by further silica gel chromatography using petroleum ether-CH_2Cl_2 (66:34 v/v) to afford pure **11** (100 mg, yield: 28%) as a white solid. ^{1}H NMR (400 MHz, DMSO-d_6) δ 10.53 (s, 1H), 9.03 (s, 1H), 8.20–8.18 (m, 1H), 8.12–8.10 (m, 1H), 7.91–7.88 (m, 1H), 7.31 (d, *J* = 4.0 Hz, 1H), 7.26–7.22 (m, 1H), 6.75 (dd, *J* = 8.0, 4.0 Hz, 1H), 6.62–6.53 (m, 2H), 4.62 (d, *J* = 4.0 Hz, 1H^{13}C NMR (101 MHz, DMSO-d_6) δ 162.4 (d, J = 242.4 Hz), 161.2, 160.7, 159.9, 157.3 (d, *J* = 10.1 Hz), 155.0, 149.6, 138.5, 131.0 (d, *J* = 10.3 Hz), 122.8 (d, *J* = 2.8 Hz), 112.2, 107.0, 105.7 (d, J = 21.2 Hz), 103.0 (d, *J* = 24.2 Hz), 102.8, 40.2. MS Calcd for $C_{15}H_{12}FN_3O_2$: 285.09. MS Found: 286.2 $[M+H]^+$.

Synthesis of 2-(((3-(1*H*-1,2,4-triazol-3-yl)pyridin-2-yl)amino)methyl)-5-fluorophenol (20).

Step 1: To a solution of **20a** (400 mg, 2.70 mmol) in THF (10 mL) was added NaH (130 mg, 3.24 mmol) at 0 °C under nitrogen atmosphere. The mixture was stirred at 0 °C for 10 min, followed by addition of SEMCl (540 mg, 3.24 mmol). The reaction was stirred at room temperature for 2 h, and then quenched with saturated NH_4Cl. The mixture was extracted with EtOAc (3×20 mL), the combined organic layer was washed with brine (50 mL), dried over anhydrous Na_2SO_4, filtered, and concentrated *in vacuo* to afford **20b** (450 mg, yield: 60%). This material was used directly without further purification.

Step 2: To a solution of **20b** (400 mg, 1.162 mmol), intermediate **S5** (388 mg, 1.12 mmol) and K_2CO_3 (95 mg, 1.395 mmol) in dioxane (5 mL) and H_2O (0.5 mL) was added Pd(dppf)Cl_2 (85 mg, 0.116 mmol) under nitrogen atmosphere. The reaction was stirred at 100 °C for 2 hours. The mixture was partitioned between EtOAc (10 mL) and water (10 mL). The organic layer was separated. The aqueous layer was extracted with EtOAc (2×10 mL). The combined organic layer was washed with brine (20 mL), dried over anhydrous Na_2SO_4, filtered and concentrated *in vacuo*.

The crude product was purified by silica gel chromatography using a mixture of petroleum ether-ethyl acetate (75:15 v/v) as eluent to afford **20c** (250 mg, yield: 52%).

Step 3: To a solution of **20c** (200 mg, 0.482 mmol) in CH_2Cl_2 (2 mL) was added TFA (8 mL) at 0 °C. The reaction was allowed to stir at room temperature for 4 h. Aqueous Na_2CO_3 was added until pH to 8. The mixture was extracted with EtOAc. The organic layer was washed with brine (20 mL), dried over anhydrous Na_2SO_4, filtered and concentrated *in vacuo*. The crude product was purified by prep-HPLC to afford **20** (30 mg, yield: 22%) as a white solid.

^{1}H NMR (400 MHz, DMSO-d_6) δ 8.73 (s, 1H), 8.52 (s, 1H), 8.25 (dd, J = 7.6, 1.6 Hz, 1H), 8.11 (dd, J = 5.2, 2.0 Hz, 1H), 7.25 (t, J = 7.6 Hz, 1H), 6.71–6.68 (m, 1H), 6.62–6.54 (m, 2H), 4.59 (d, J = 4.4 Hz, 2H). ^{13}C NMR (101 MHz, DMSO-d_6) δ 162.4 (d, J = 241.6 Hz), 157.9, 157.5 (d, J = 11.3 Hz), 155.1, 148.4, 146.2, 135.8, 130.9 (d, J = 10.3 Hz), 123.2 (d, J = 2.8 Hz), 112.0, 108.9, 105.6 (d, J = 21.0 Hz), 103.1 (d, J = 23.4 Hz), 40.6. MS Calcd for $C_{14}H_{12}FN_5O$: 285.10; MS Found: 286.2 $[M+H]^+$.

Synthesis of 2-(((3-(1,2,4-oxadiazol-3-yl)pyridin-2-yl)amino)methyl)-5-fluorophenol (21).

Step 1: To a solution of 4-fluoro-2-hydroxybenzaldehyde (3.00 g, 25.2 mmol), 2-aminonicotinonitrile (5.3 g, 37.8 mmol) in toluene (100 mL) was added camphor-10-sulfonic acid (0.9 g, 3.78 mmol). A Dean–Stark apparatus was equipped, and the reaction mixture was heated at reflux until LCMS showed 3-bromopyridin-2-amine was consumed completely. The reaction mixture was then cooled to 0 °C and $NaBH_4$ (3.39 g, 89.3 mmol) was added in portions. Subsequently, the reaction was allowed to stir at room temperature overnight. Water (100 mL) was then added and the mixture was extracted with CH_2Cl_2 (200 mL). The organic layer was separated and the aqueous layer was extracted with more CH_2Cl_2 (2×50 mL). The combined organic layers were washed with brine (50 mL), dried over anhydrous Na_2SO_4, filtered and concentrated *in vacuo*. The crude product was purified by silica gel chromatography using a mixture of petroleum ether-ethyl acetate (90:10 to 80:20 v/v) as eluent to afford **21a** (4.00 g, yield: 65%). ^{1}H NMR (400 MHz, DMSO-d_6) δ 10.23 (s, 1H), 8.25 (dd, J = 4.9, 1.9 Hz, 1H), 7.94 (dd, J = 7.6, 1.9 Hz, 1H), 7.47 (t, J = 5.9 Hz, 1H), 7.14 – 7.05 (m, 1H), 6.71 – 6.50 (m, 3H), 4.48 (d, J = 5.9 Hz, 2H).

Step 2: To a solution of **21a** (400 mg, 1.65 mmol) and Na_2CO_3 (520 mg, 4.95 mmol) in EtOH (10 mL) was added hydroxylamine hydrochloride (34.2 mg, 4.95 mmol). The reaction was stirred at 80 °C for 2 hours. The reaction was concentrated *in vacuo*, and the residue was purified by silica gel chromatography using a mixture of petroleum ether-ethyl acetate (90:10 to 80:20 v/v) as eluent to afford **21b** (280 mg, yield: 62%). ^{1}H NMR (400 MHz, DMSO-d_6) δ 10.76 (s, 1H), 9.94 (s, 1H), 8.62 (t, J = 5.9 Hz, 1H), 8.02 (dd, J = 4.9, 1.7 Hz, 1H), 7.82 (dd, J = 7.6, 1.7 Hz, 1H), 7.22 – 7.14 (m, 1H), 6.66 – 6.51 (m, 3H), 5.97 (s, 2H), 4.49 (d, J = 5.9 Hz, 2H).

Step 3: To a solution of **21b** (276 mg, 1.0 mmol) in triethyl orthoformate (10 mL) was added triethylamine (0.1 mL) and the reaction mixture was stirred at 80 °C for 4 h. The reaction mixture was then concentrated and purified by silica gel chromatography using a mixture of petroleum ether-ethyl acetate (100:0 to 75:25 v/v) as eluent to afford crude product, and further purification by using prep-HPLC to afford **21** (60 mg, 21%) as a white solid. ^{1}H NMR (400 MHz, DMSO-d_6) δ 10.44 (s, 1H), 9.75 (s, 1H), 8.32–8.29 (m, 2H), 7.58–7.56 (m, 1H), 7.25–7.23 (m, 1H), 6.81–6.78

(m, 1H), 6.64–6.56 (m, 2H), 4.65 (d, J = 4.0 Hz, 2H); ^{13}C NMR (101 MHz, DMSO-d_6) δ 166.6, 165.8, 162.4 (d, J = 242.8 Hz), 157.3 (d, J = 11.1 Hz), 155.3, 151.5, 138.7, 130.8 (d, J = 10.3 Hz), 122.5 (d, J = 2.8 Hz), 112.5, 105.7 (d, J = 21.1 Hz), 104.6, 102.9 (d, J = 23.7 Hz), 40.3. MS Calcd for $C_{14}H_{11}FN_4O_2$: 286.09; MS Found: 287.1 [M+H]$^+$.

Synthesis of 2-(((3-ethynylpyridin-2-yl)((2-(trimethylsilyl)ethoxy)methyl)amino)methyl)-5-fluorophenol (compound 22).

Step 1: To a solution of intermediate **S2** (460 mg, 1.55 mmol), ethynyltrimethylsilane (761 mg, 7.77 mmol) and Et_3N (470 mg, 4.65 mmol) in dry dimethylacetamide (5 mL) was added Pd(dppf)Cl_2 (113 mg, 0.155 mmol) under a nitrogen atmosphere. The reaction mixture was stirred at 100 °C with microwave irradiation for 1 hour. After cooling, the mixture was treated with K_2CO_3 and MeOH (10 mL) and then stirred at room temperature for 1 hour. The reaction was quenched with water (20 mL) and extracted with EtOAc (3×30 mL). The combined organic layer was washed with brine (50 mL), dried over anhydrous Na_2SO_4, filtered and concentrated *in vacuo*. The crude product was purified by silica gel chromatography using a mixture of petroleum ether-ethyl acetate (90:10 to 85:15 v/v) as eluent to afford **22a**. ^{1}H NMR (400 MHz, $CDCl_3$) δ 11.88 (br, 1H), 8.02 (dd, J = 5.3, 1.8 Hz, 1H), 7.54 (dd, J = 7.4, 1.8 Hz, 1H), 7.16-7.11 (m, 1H), 6.61-6.49 (m, 3H), 5.98 (t, J = 5.5 Hz, 1H), 4.45 (d, J = 6.6 Hz, 2H), 3.47 (s, 1H).

Step 2: To a solution of **22a** (800 mg, 3.3 mmol) in dry THF (10 mL) was added NaH (422 mg, 10.56 mmol) at 0 °C under nitrogen atmosphere. After 5 min, SEMCl (823 mg, 4.96 mmol) was added. The reaction was stirred for 30 min at room temperature. The mixture was quenched with saturated NH_4Cl and extracted with EtOAc (3×20 mL). The combined organic layer was washed with brine (20 mL), dried over anhydrous Na_2SO_4, filtered and concentrated *in vacuo*. The crude product was purified by silica gel chromatography using a mixture of petroleum ether-ethyl acetate (90:10 v/v) as eluent to afford **22b** (220 mg, yield: 18%).

Step 3: To a solution of **22b** (200 mg, 0.54 mmol) and NaN_3 (70.2 mg, 10.8 mmol) in dimethylacetamide (5 mL) was added CuI (10 mg, 0.054 mmol) under a nitrogen atmosphere. The reaction was stirred for 3 hours at 100 °C with microwave irradiation. After cooling to room temperature, the mixture was concentrated to dryness *in vacuo*. The residue was purified by silica gel chromatography using a mixture of petroleum ether-ethyl acetate (75:25 v/v) as eluent to afford **22c** (200 mg, yield: 89%).

Step 4: To a solution of **22c** (180 mg, 0.434 mmol) in EtOH (80%) was added conc. HCl (2 mL). The reaction was stirred at 80 °C for 16 hours. The reaction was quenched with saturated $NaHCO_3$ and extracted with EtOAc (3×30 mL). The combined organic layer was washed with brine (30 mL), dried over anhydrous Na_2SO_4, filtered and concentrated *in vacuo*. The crude product was obtained through purification by using silica gel chromatography with a mixture of petroleum ether-ethyl acetate (75:25 v/v) as eluent. Further purification by prep-HPLC to afforded compound **22** (12 mg, 9.7%) as a white solid. ^{1}H NMR (400 MHz, DMSO-d_6) δ 8.38 (br s, 1H), 8.06–8.05 (m, 2H), 7.26–7.23 (m, 1H), 6.69 (dd, J = 8.0, 4.0 Hz, 1H), 6.61–6.53 (m, 2H), 4.56 (s, 2H).
MS Calcd for $C_{14}H_{12}FN_5O$: 285.10; MS Found: 286.2 [M+H]$^+$.

Synthesis of 2-(((3-(2H-tetrazol-5-yl)pyridin-2-yl)amino)methyl)-5-fluorophenol (23). Solid sodium azide NaN_3 (321 mg, 4.94 mmol) was added to a solution of **21a** (400 mg, 1.65 mmol) in DMF and the reaction mixture was stirred at 120 °C for 16 hours. After cooling, saturated $NaHCO_3$ was added and the mixture was extracted with EtOAc (10 mL X 3). The organic layer was dried over Na_2SO_4, filtered, and concentrated in vacuo. The crude product was purified by silica gel chromatography (25-100% EtOAc petroleum ether to provide product compound 23 (52 mg, 11%) as a white solid. ^{1}H NMR (400 MHz, DMSO-d_6) δ 8.5 (br, 1H), 8.26 (dd, J = 8.0, 4.0 Hz, 1H), 8.18 (d, J = 4.0 Hz, 1H), 7.26 (t, J = 8.0 Hz, 1H), 6.81 (dd, J = 8.0, 4.0 Hz, 1H), 6.82 - 6.57 (m, 2H), 4.64 (s, 2H). MS (ESI+, m/z): Calcd for $C_{13}H_{11}FN_6O$: 286.1; found 287.3 $[M+H]^+$.

Synthesis of 5-fluoro-2-(((3-(furan-2-yl)pyridin-2-yl)amino)methyl)phenol (27).

Step 1: To a solution of 3-bromopyridin-2-amine (443 mg, 2.58 mmol), 2-(furan-2-yl)-4,4,5,5-tetramethyl-1,3,2-dioxaborolane (500 mg, 2.58 mmol) and K_2CO_3 (712 mg, 5.16 mmol) in dioxane (5 mL) and H_2O (0.5 mL) was added Pd(dppf)Cl_2 (189 mg, 0.258 mmol) under nitrogen atmosphere. The reaction was stirred at 120 °C with microwave for 1 hours. The mixture was partitioned between DCM (10 mL) and water (10 mL). The organic layer was separated. The aqueous layer was extracted with DCM (2×10 mL). The combined organic layer was washed with brine (20 mL), dried over anhydrous Na_2SO_4, filtered and concentrated *in vacuo*. The crude product was purified by silica gel chromatography using a mixture of petroleum ether-ethyl acetate (100:0 to 90:10 v/v) as eluent to afford **27a** (400 mg, yield: 97%). ^{1}H NMR (400 MHz, $CDCl_3$) δ 8.03 (dd, J = 4.9, 1.8 Hz, 1H), 7.71 (dd, J = 7.6, 1.8 Hz, 1H), 7.52 (dd, J = 1.8, 0.6 Hz, 1H), 6.75-6.69 (m, 1H), 6.62 (dd, J = 3.4, 0.6 Hz, 1H), 6.54-6.50 (m, 1H), 5.28 (br, 2H).

Step 2: To a solution of **27a** (288 mg, 2.06 mmol), 4-fluoro-2-hydroxybenzaldehyde (220 mg, 1.37 mmol) and AcOH (107 mg, 1.79 mmol) in MeOH (10 mL) was added borane 2-methylpyridine (190 mg, 1.79 mmol) under nitrogen atmosphere. The reaction was stirred at 60 °C overnight. The reaction mixture was quenched with saturated $NaHCO_3$ (10 mL), and the mixture was extracted with EtOAc (3×15 mL). The combined organic layer was washed with brine, dried over anhydrous Na_2SO_4, filtered and concentrated *in vacuo*. The crude product was purified by silica gel chromatography using a mixture of petroleum ether-ethyl acetate (100:0 to 90:10 v/v) as eluent to afford crude product. Further purification was performed by using prep-HPLC to afford **27** (60 mg, 15%) as a white solid. ^{1}H NMR (400 MHz, DMSO-d_6) δ 12.30 (s, 1H), 8.06–8.05 (m, 1H), 7.67–8.05 (m, 1H), 7.55–7.54 (m, 1H), 7.19–7.15 (m, 1H), 6.70–6.52 (m, 6H), 4.51 (d, J = 4.0 Hz, 2H). ^{13}C NMR (101 MHz, DMSO-d_6) δ 162.4 (d, J = 242.3 Hz), 157.4 (d, J = 11.4 Hz), 153.7, 150.5, 146.8, 143.2, 135.4, 130.9 (d, J = 10.3 Hz), 123.3 (d, J = 2.7 Hz), 112.7, 112.3, 111.3, 108.3, 105.6 (d, J = 21.0 Hz), 103.2 (d, J = 23.5 Hz), 40.5. MS Calcd for $C_{16}H_{13}FN_2O_2$: 284.10; MS Found: 285.2 $[M+H]^+$.

Synthesis of 6-amino-*N*-((2'-((4-fluoro-2-hydroxybenzyl)amino)-[2,3'-bipyridin]-5-yl)methyl)hexanamide (31)

Step1: A mixture of *tert*-butyl ((6-bromopyridin-3-yl)methyl)carbamate (**31a**, 100 mg, 0.348 mmol) was taken in a mixture of TFA and dichloromethane (1.25 mL, TFA/DCM = 1:4) and stirred at 25 °C for 4 hours. Subsequently, the solvent was removed by rotary evaporation

followed by high vacuum. This crude residue **31b** was used in next reaction without further purification. ^{1}H NMR (400 MHz, methanol-*d_4*) δ 8.47 (d, *J* = 2.5 Hz, 1H), 7.82 (dd, *J* = 8.3, 2.5 Hz, 1H), 7.72 (d, *J* = 8.3 Hz, 1H), 4.19 (s, 2H).

Step 2: A mixture of **31b** (105 mg, 0.348 mmol, 1.0 eq.), 6-((tert-butoxycarbonyl)amino)hexanoic acid (96.6 mg, 0.418 mmol, 1.20 eq.), 3-(((ethylimino)methylene)amino)-*N*,*N*-dimethylpropan-1-amine hydrochloride (EDC, 200 mg, 1.04 mmol, 3.0 eq.) DIPEA (145.4 µL, 0.836 mmol, 2.4 eq.) and *N*,*N*-dimethylpyridin-4-amine (DMAP, 42.6 mg, 0.348 mmol, 1.0 eq.) was mixed in 2.0 mL of DMF and stirred at 25 °C for 48 hours. Water (5 mL) was then added, and the mixture was extracted with EtOAc (5 mL). The organic layer was separated, and the aqueous layer was extracted with more EtOAc (2×5 mL). The combined organic layers were washed with 1M HCl (5 mL), sat. $NaHCO_3$ (5 mL), brine (5 mL), dried over anhydrous Na_2SO_4, filtered and concentrated *in vacuo*. The crude product was purified by silica gel chromatography using a mixture of hexane -ethyl acetate (90:10 to 0:100 v/v) as eluent to afford **31c** (73 mg, yield: 53%). ^{1}H NMR (400 MHz, $CDCl_3$) δ 8.30 (d, *J* = 2.5 Hz, 1H), 7.54 (dd, *J* = 8.2, 2.5 Hz, 1H), 7.46 (d, *J* = 8.1 Hz, 1H), 6.02 (s, 1H), 4.54 (s, 1H), 4.41 (d, *J* = 6.0 Hz, 2H), 3.10 (t, *J* = 7.0 Hz, 2H), 2.23 (t, *J* = 7.5 Hz, 2H), 1.71-1.63 (m, 2H), 1.53-1.46 (m, 2H), 1.43 (s, 9H), 1.38-1.30 (m, 2H).

Step 3: A mixture of **31c** (40 mg, 0.10 mmol), intermediate **S4** (44 mg, 0.12 mmol), Pd(dppf)Cl_2 (5.5 mg, 7.5 µmol) and Cs_2CO_3 (81 mg, 0.25 mmol) in 0.46 mL of mixed solvent (dioxane/H_2O = 10/1) was stirred at 60 °C for 4 hours. Subsequently, the mixture was extracted with EtOAc by three times, the combined organic phase was washed with sat. $NaHCO_3$, H_2O, brine, dried over Na_2SO_4 and concentrated *in vacuo*. The crude resideu purified by prep-HPLC to provide compound **31d** as white solid (16.2 mg, 28%) as white solid. ^{1}H NMR (400 MHz, methanol-*d_4*) δ 8.56 (d, *J* = 7.2 Hz, 1H), 8.30 (brs, 1H), 8.08-8.00 (m, 2H), 7.88-7.78 (m, 2H), 7.33-7.21 (m, 1H), 6.76-6.70 (m, 1H), 6.56-6.50 (m, 2H), 4.54 (s, 2H), 4.43 (s, 2H), 3.03 (t, *J* = 7.0 Hz, 2H), 2.28 (t, *J* = 7.3 Hz, 2H), 1.71-1.63 (m, 2H), 1.53-1.45 (m, 2H), 1.43 (s, 9H), 1.39-1.27 (m, 2H).

Step 4: In a 4-mL vial equipped with a stirring bar and a cap was placed 16.2 mg (27.8 µmol) of **31d** and the vial cooled to 0 °C. Next a solution of 4M HCl in dioxane (0.150 mL), dichloromethane (0.10 mL), and methanol (0.10 mL) were added and the reaction mixture was stirred for 4 hours at room temperature. At this time the reaction was judged complete and the reachtion mixture was concentrated in vacuo. The crude product was purified by preperative HPLC (0.1% formic acid MeCN/H_2O 0% to 50%) to afford **31** (14.3 mg, 97%) as white solid. ^{1}H NMR (400 MHz, methanol-*d_4*) δ 8.57 (s, 1H), 8.07-8.03 (m, 2H), 7.87-7.83 (m, 2H), 7.27 (t, *J* = 7.4 Hz, 1H), 6.74 (dd, *J* = 7.5, 5.2 Hz, 1H), 6.59-6.52 (m, 2H), 4.55 (s, 1H), 4.44 (s, 2H), 2.93 (t, *J* = 7.8 Hz, 2H), 2.32 (t, *J* = 7.4 Hz, 2H), 1.77-1.63 (m, 4H), 1.46-1.41 (m, 2H). ^{13}C NMR (101 MHz, methanol-*d_4*) δ 174.5, 163.2 (d, *J* = 243.2 Hz), 157.3, 155.0, 154.6, 146.6, 144.4, 137.7, 136.7, 133.3, 131.1, 121.9, 121.4, 117.5, 111.6, 105.6, (d, *J* = 21.6 Hz), 103.1 (d, *J* = 23.8 Hz), 40.0, 39.13, 39.05, 35.1, 26.9, 25.6, 24.8. MS (ESI^+, m/z): Calcd for $C_{24}H_{28}FN_5O_2$: 437.2; found 438.4 $[M+H]^+$.

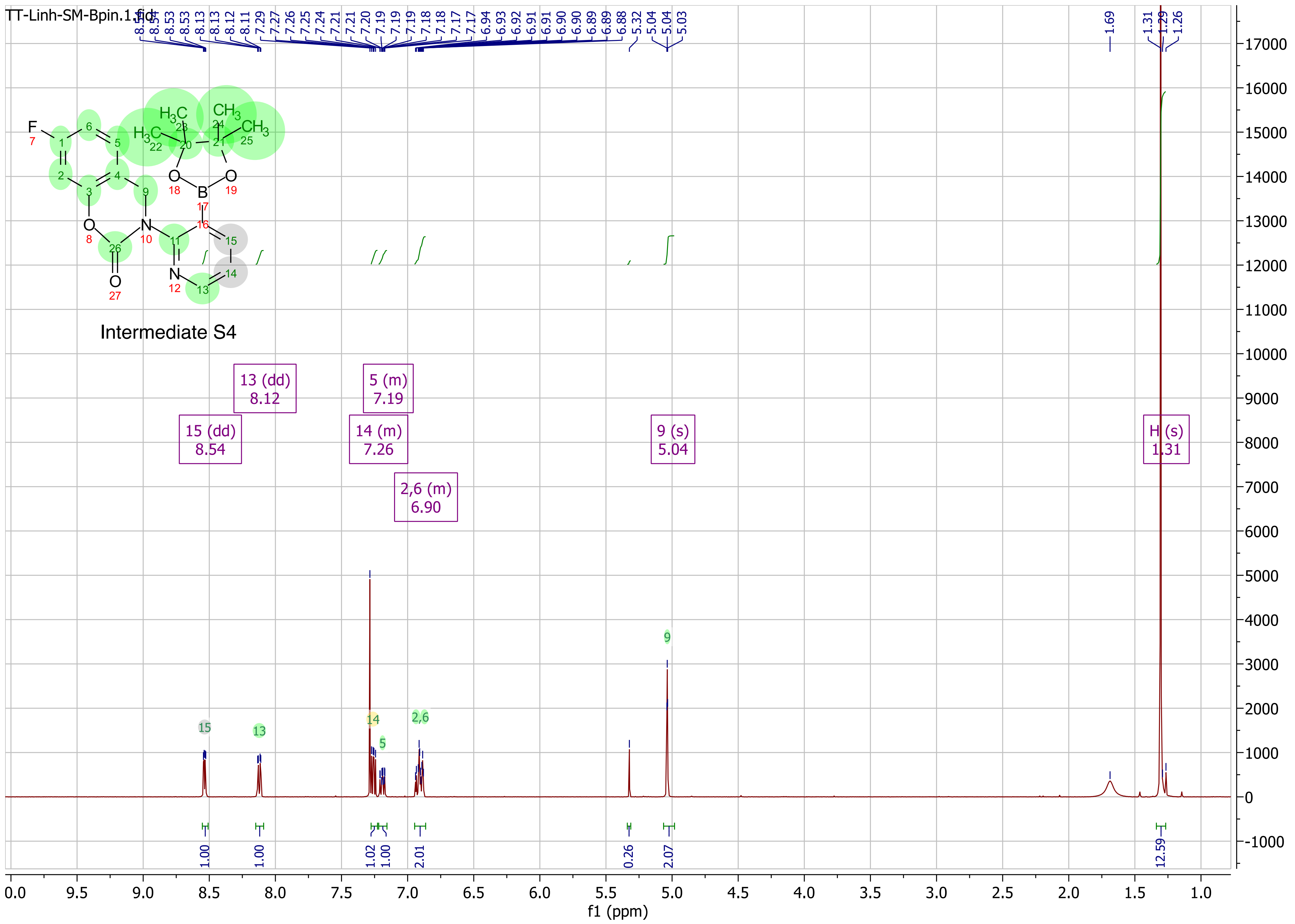

TT-Linh-SM-Bpin.1.fid
Intermediate S4
15 (dd) 8.54
13 (dd) 8.12
14 (m) 7.26
5 (m) 7.19
2,6 (m) 6.90
9 (s) 5.04
H (s) 1.31
8.54 8.54 8.53 8.53 8.13 8.13 8.12 8.11 7.29 7.27 7.26 7.25 7.24 7.21 7.21 7.20 7.19 7.19 7.19 7.18 7.18 7.17 7.17 6.94 6.93 6.92 6.91 6.91 6.90 6.90 6.89 6.89 6.88 5.32 5.04 5.04 5.03 1.69 1.31 1.29 1.26
1.00 1.00 1.02 1.00 2.01 0.26 2.07 12.59
f1 (ppm)

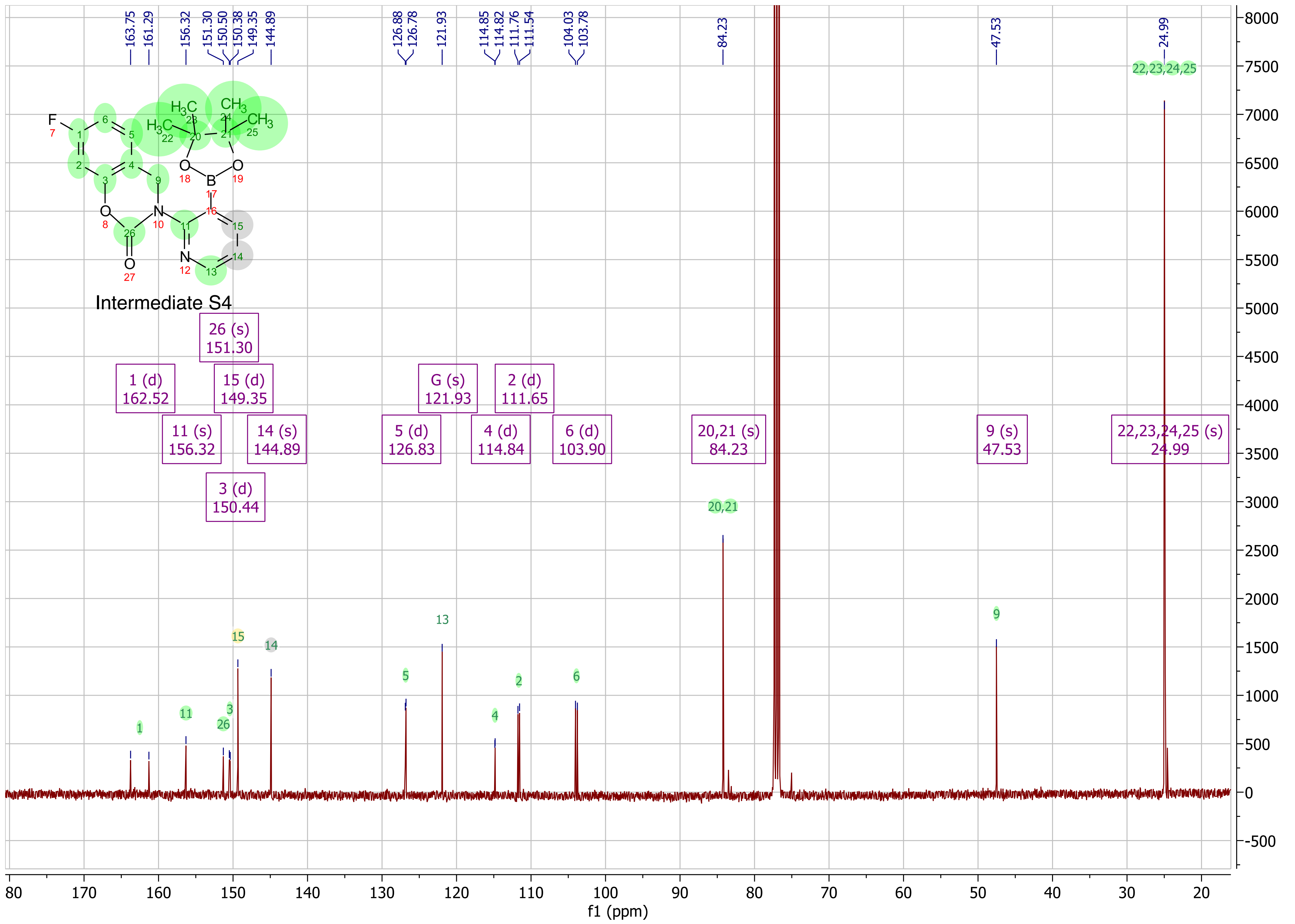

163.75
161.29
156.32
151.30
150.50
150.38
149.35
144.89
126.88
126.78
121.93
114.85
114.82
111.76
111.54
104.03
103.78
84.23
47.53
24.99
Intermediate S4
26 (s) 151.30
1 (d) 162.52
15 (d) 149.35
G (s) 121.93
2 (d) 111.65
11 (s) 156.32
14 (s) 144.89
5 (d) 126.83
4 (d) 114.84
6 (d) 103.90
20,21 (s) 84.23
9 (s) 47.53
22,23,24,25 (s) 24.99
3 (d) 150.44
f1 (ppm)

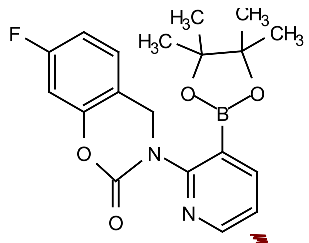

HMBC spectra
Of Intermediate S4

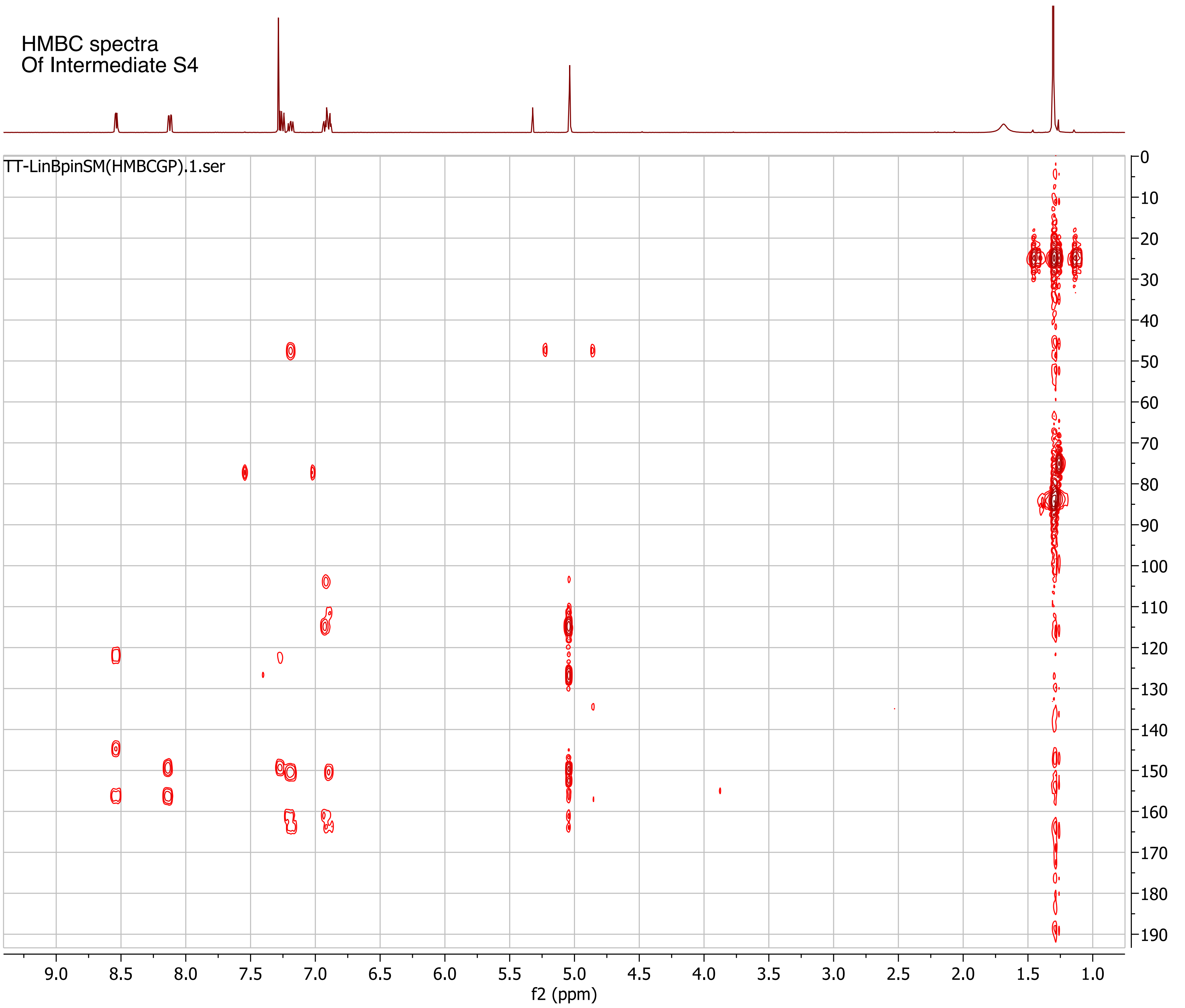

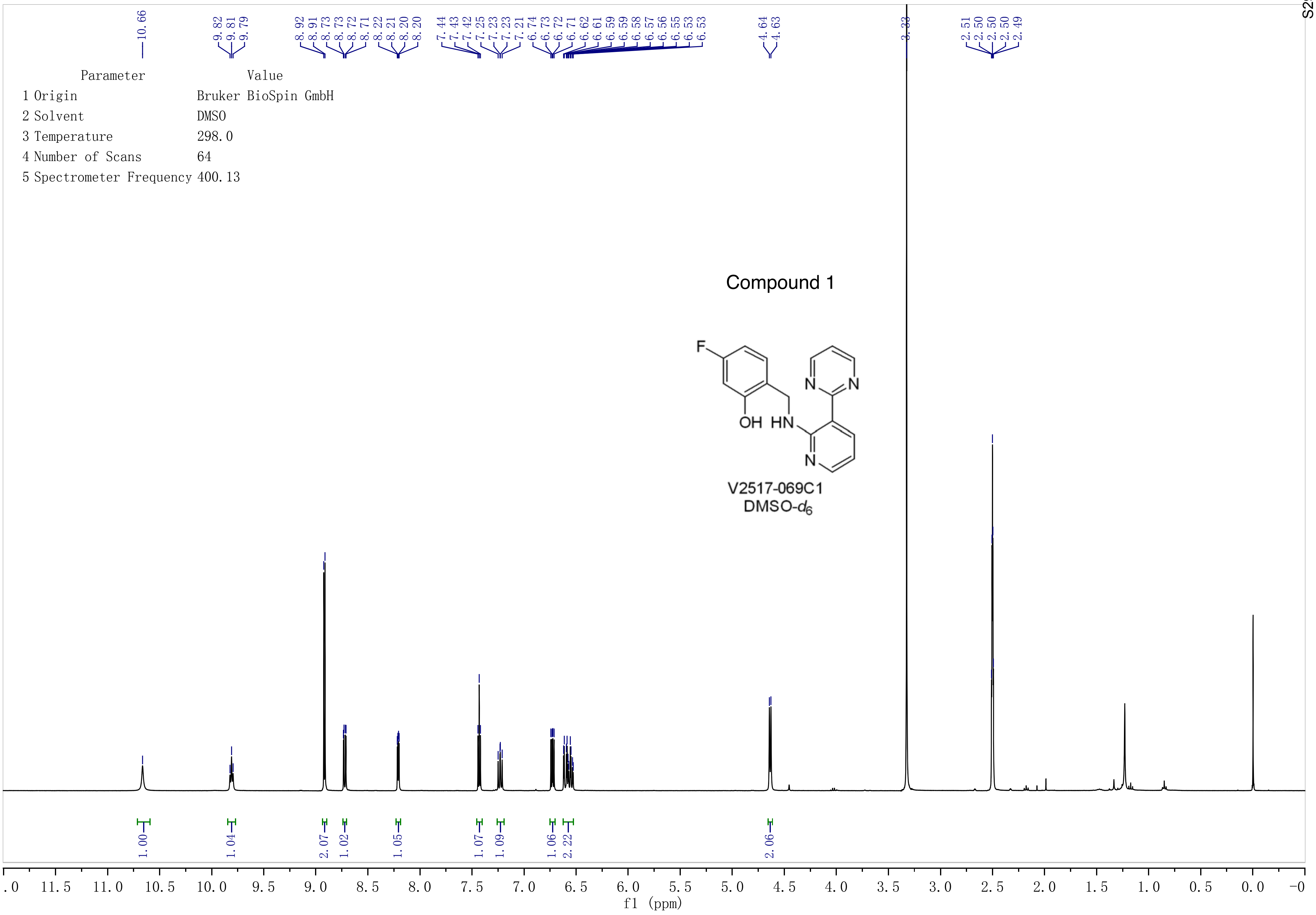

Parameter Value
1 Origin Bruker BioSpin GmbH
2 Solvent DMSO
3 Temperature 298.0
4 Number of Scans 64
5 Spectrometer Frequency 400.13
10.66
9.82 9.81 9.79
8.92 8.91 8.73 8.73 8.72 8.71 8.22 8.21 8.20 8.20
7.44 7.43 7.42 7.25 7.23 7.23 7.21
6.74 6.73 6.72 6.71 6.62 6.61 6.59 6.59 6.58 6.57 6.56 6.55 6.53 6.53
4.64 4.63
3.33
2.51 2.50 2.50 2.50 2.49
Compound 1
F
OH HN
N N
N
V2517-069C1
DMSO-d6
1.00 1.04 2.07 1.02 1.05 1.07 1.09 1.06 2.22 2.06
f1 (ppm)

SAMPLE INFORMATION

Sample Name:	V2517-069	Acquired By:	System
Sample Type:	Standard	Sample Set Name	20200807
Vial:	1:F,2	Acq. Method Set:	VIVA QC_WATERS BEH C18
Injection #:	1	Processing Method	Process standrads method
Injection Volume:	0.50 ul	Channel Name:	PDA Ch3 214nm@4.8nm, PDA
Run Time:	15.0 Minutes	Proc. Chnl. Descr.:	PDA Ch3 214nm@4.8nm, PDA

Date Acquired: 8/7/2020 6:06:26 PM CST
Date Processed: 8/8/2020 9:03:25 AM CST, 8/8/2020 9:03:45 AM CST, 8/8/2020 9:04:05 AM CST

Compound 1

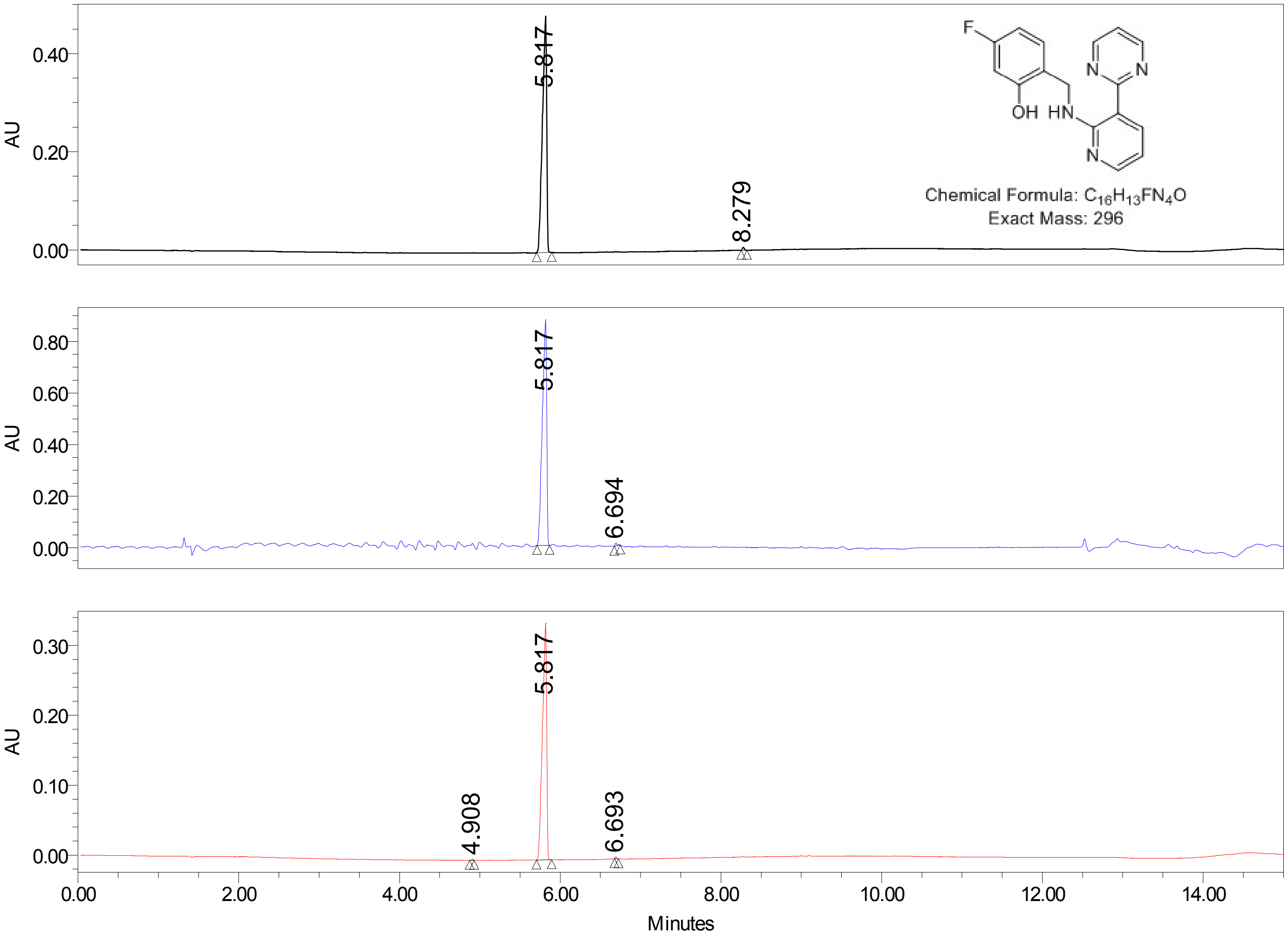

Channel: PDA Ch1 254nm@4.8nm; Processed Channel: PDA Ch1 254nm@4.8nm; Result Id: 6227; Processing Method: Process standrads method
Channel: PDA Ch3 214nm@4.8nm; Processed Channel: PDA Ch3 214nm@4.8nm; Result Id: 6229; Processing Method: Process standrads method
Channel: PDA Ch2 280nm@4.8nm; Processed Channel: PDA Ch2 280nm@4.8nm; Result Id: 6228; Processing Method: Process standrads method

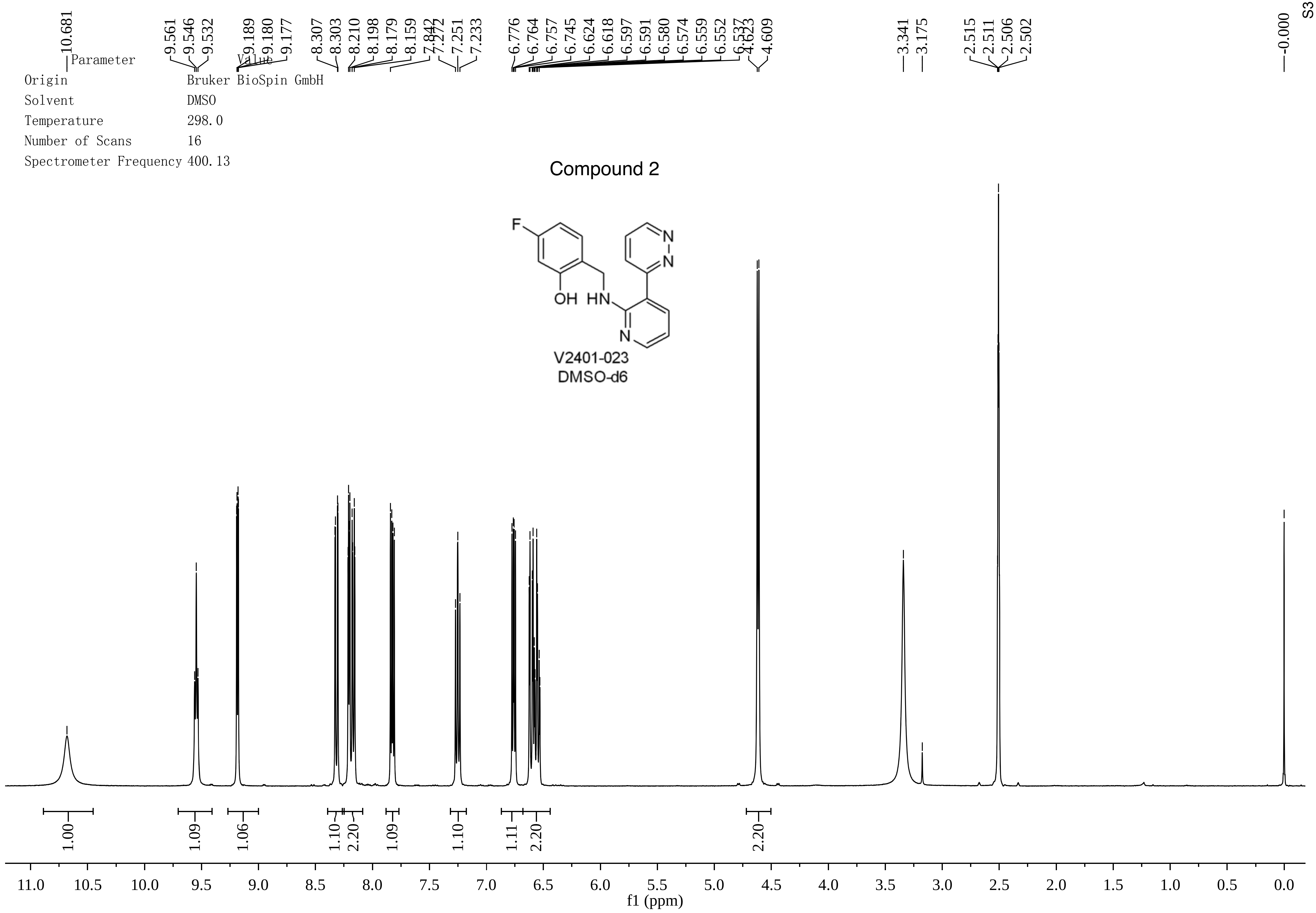

10.681
9.561
9.546
9.532
9.189
9.180
9.177
8.307
8.303
8.210
8.198
8.179
8.159
7.842
7.272
7.251
7.233
6.776
6.764
6.757
6.745
6.624
6.618
6.597
6.591
6.580
6.574
6.559
6.552
6.537
4.623
4.609
3.341
3.175
2.515
2.511
2.506
2.502
-0.000
Parameter
Value
Origin
Bruker BioSpin GmbH
Solvent
DMSO
Temperature
298.0
Number of Scans
16
Spectrometer Frequency
400.13
Compound 2
F
OH
HN
N
N
N
V2401-023
DMSO-d6
1.00
1.09
1.06
1.10
2.20
1.09
1.10
1.11
2.20
2.20
11.0
10.5
10.0
9.5
9.0
8.5
8.0
7.5
7.0
6.5
6.0
5.5
5.0
4.5
4.0
3.5
3.0
2.5
2.0
1.5
1.0
0.5
0.0
f1 (ppm)

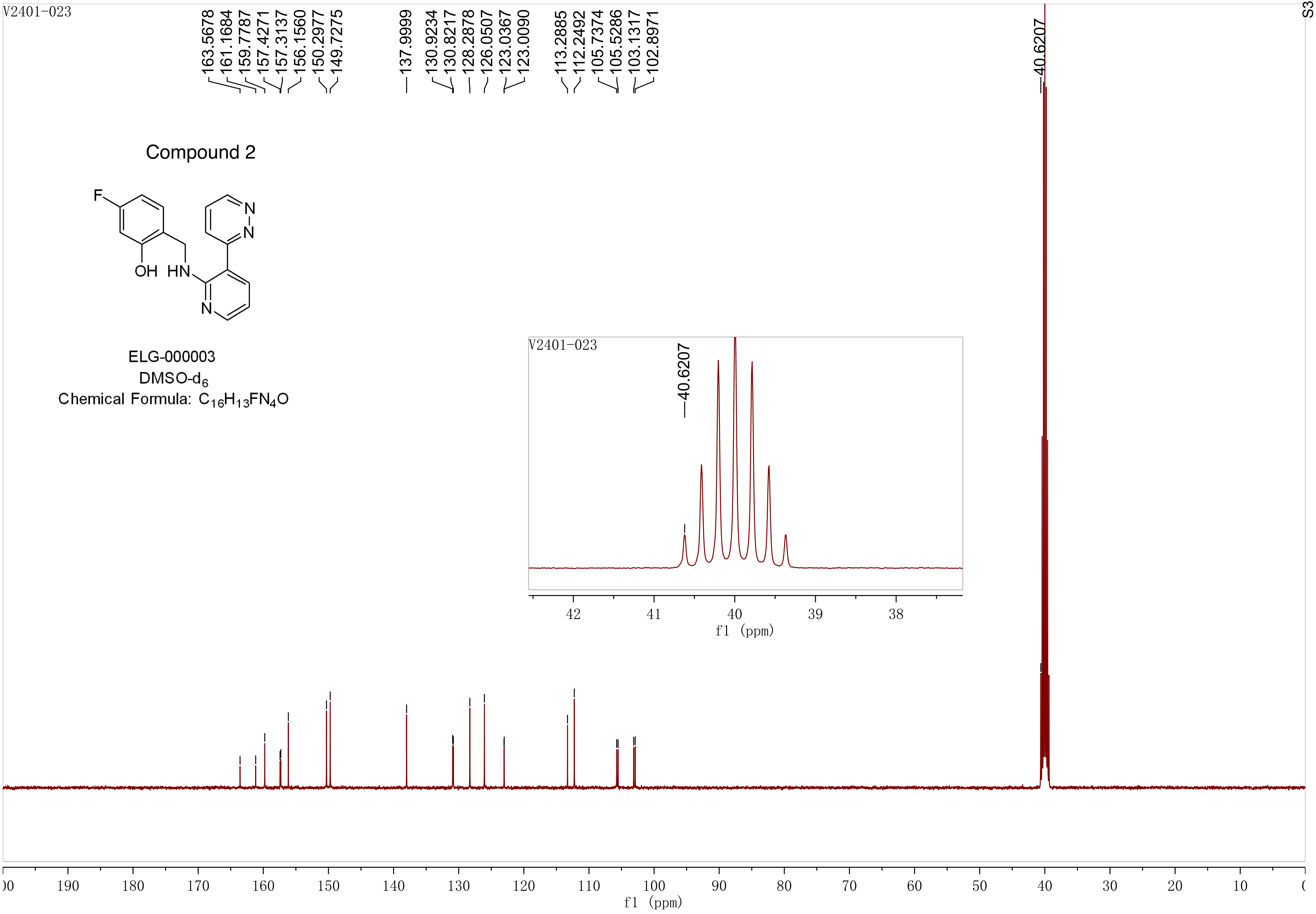
V2401-023
163.5678
161.1684
159.7787
157.4271
157.3137
156.1560
150.2977
149.7275
137.9999
130.9234
130.8217
128.2878
126.0507
123.0367
123.0090
113.2885
112.2492
105.7374
105.5286
103.1317
102.8971
40.6207
Compound 2
F
OH
HN
N
N
N
ELG-000003
DMSO-d6
Chemical Formula: C16H13FN4O
V2401-023
40.6207
42
41
40
39
38
f1 (ppm)
190
180
170
160
150
140
130
120
110
100
90
80
70
60
50
40
30
20
10
f1 (ppm)

Injection Summary Report

SAMPLE INFORMATION

Sample Name:	V2401-023	Acquired By:	System
Sample Type:	Standard	Sample Set Name	20200617
Vial:	2:D,8	Acq. Method Set:	VIVA QC_WATERS BEH C18
Injection #:	1	Processing Method	214, Process standrads method
Injection Volume:	0.50 ul	Channel Name:	PDA Ch3 214nm@4.8nm, PDA
Run Time:	15.0 Minutes	Proc. Chnl. Descr.:	PDA Ch3 214nm@4.8nm, PDA

Date Acquired: 6/17/2020 3:08:12 PM CST
Date Processed: 6/17/2020 3:29:56 PM CST, 6/17/2020 3:30:06 PM CST, 6/17/2020 3:30:22 PM CST

Compound 2

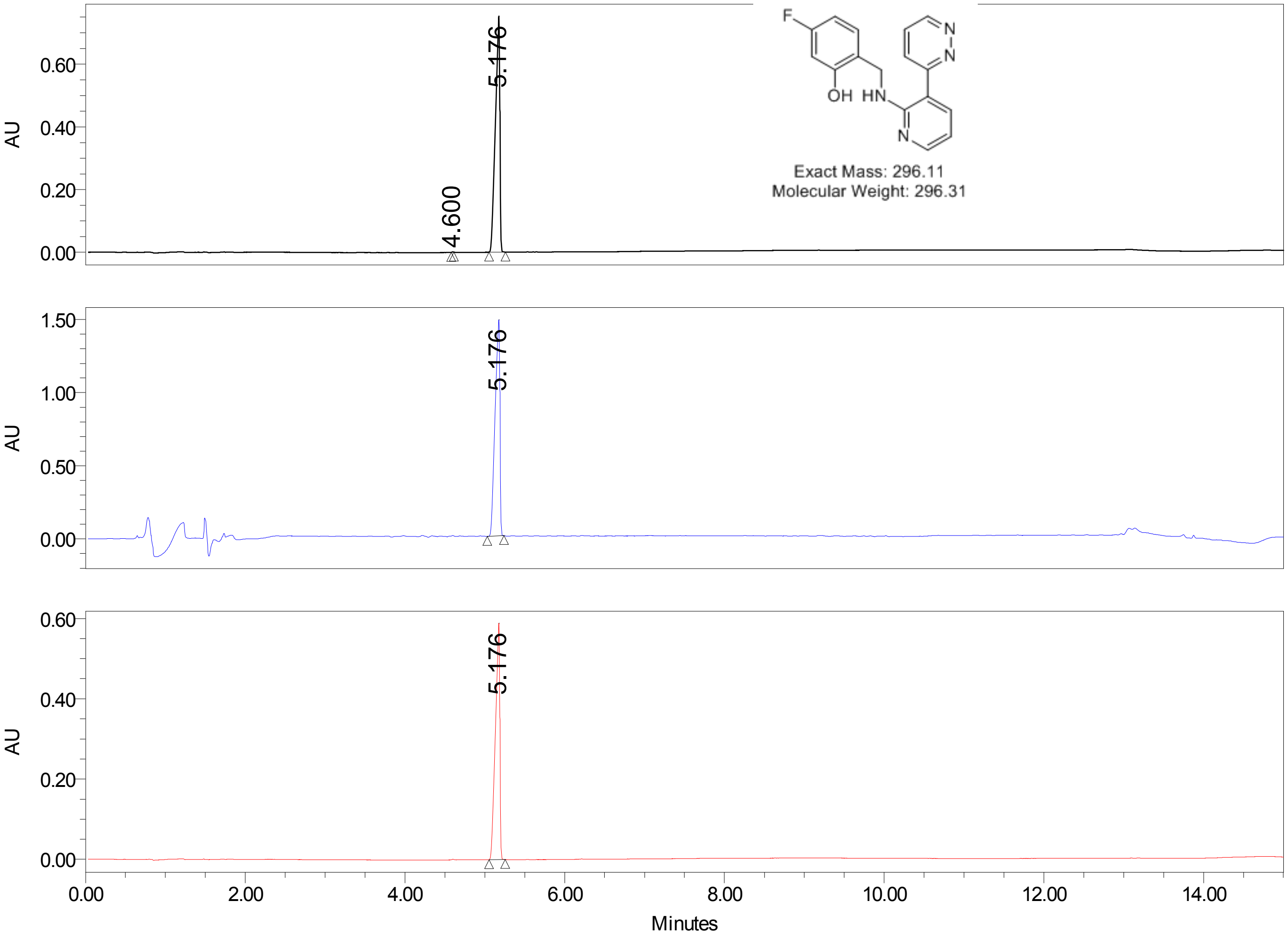

Channel: PDA Ch1 254nm@4.8nm; Processed Channel: PDA Ch1 254nm@4.8nm; Result Id: 14782; Processing Method: Process standrads method
Channel: PDA Ch3 214nm@4.8nm; Processed Channel: PDA Ch3 214nm@4.8nm; Result Id: 14784; Processing Method: 214
Channel: PDA Ch2 280nm@4.8nm; Processed Channel: PDA Ch2 280nm@4.8nm; Result Id: 14783; Processing Method: Process standrads method

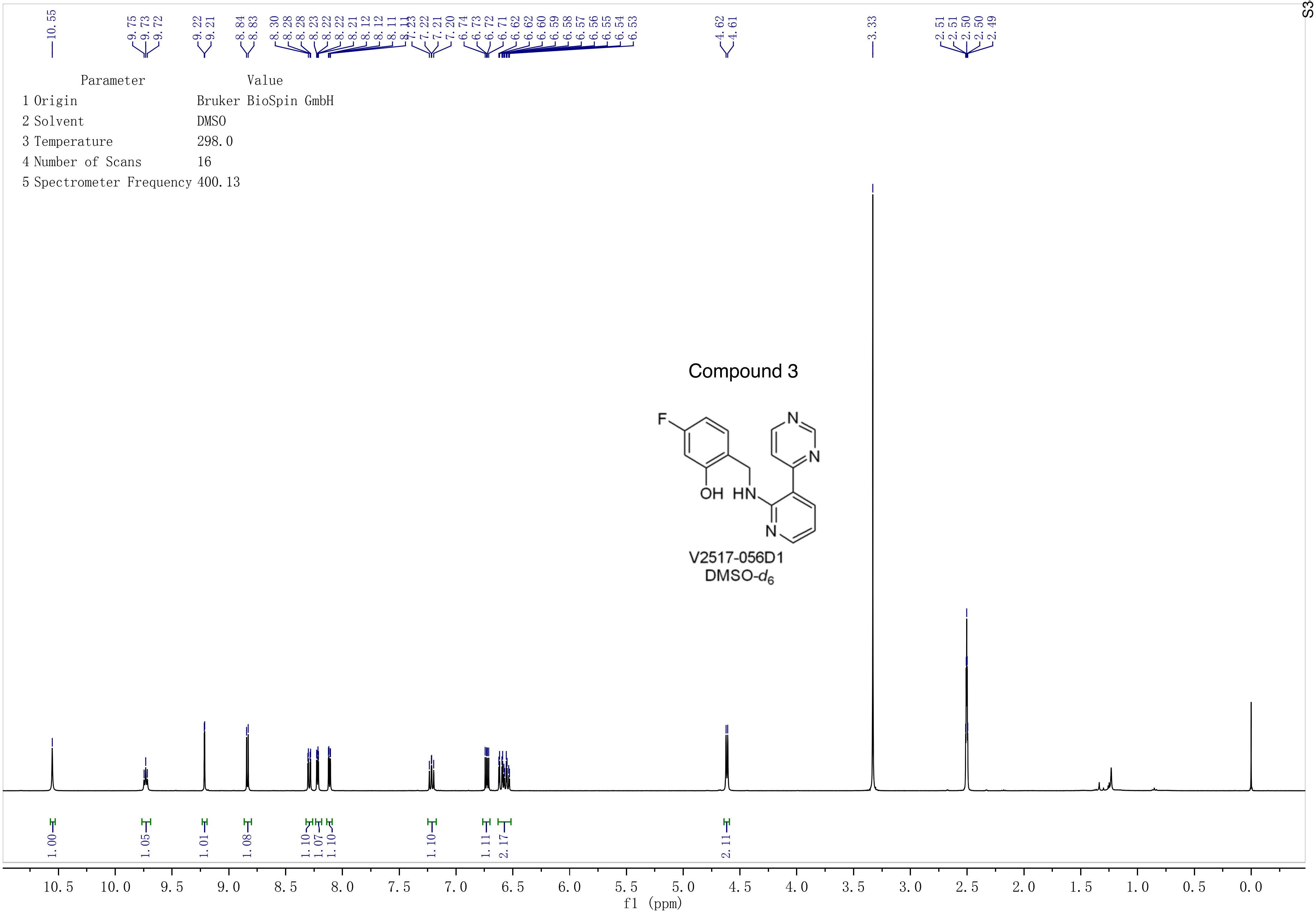

10.55
9.75 9.73 9.72
9.22 9.21
8.84 8.83
8.30 8.28 8.28 8.23 8.22 8.22 8.21 8.12 8.12 8.11 8.11
7.23 7.22 7.21 7.20
6.74 6.73 6.72 6.71 6.62 6.62 6.60 6.59 6.58 6.57 6.56 6.55 6.54 6.53
4.62 4.61
3.33
2.51 2.51 2.50 2.50 2.49
Parameter Value
1 Origin Bruker BioSpin GmbH
2 Solvent DMSO
3 Temperature 298.0
4 Number of Scans 16
5 Spectrometer Frequency 400.13
Compound 3
F
OH HN
N
N
N
V2517-056D1
DMSO-d6
1.00 1.05 1.01 1.08 1.10 1.07 1.10 1.10 1.11 2.17 2.11
10.5 10.0 9.5 9.0 8.5 8.0 7.5 7.0 6.5 6.0 5.5 5.0 4.5 4.0 3.5 3.0 2.5 2.0 1.5 1.0 0.5 0.0
f1 (ppm)

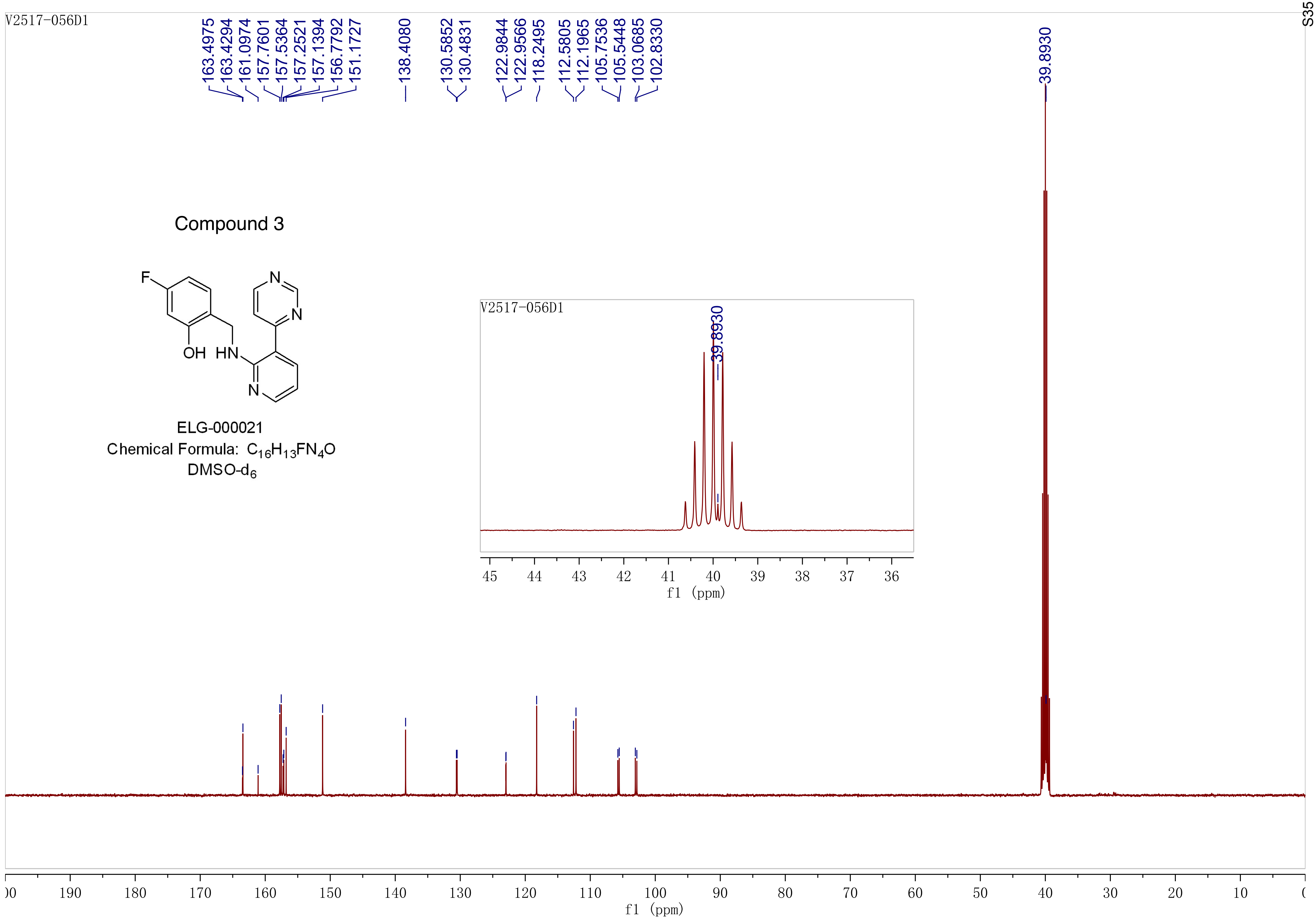
V2517-056D1
163.4975
163.4294
161.0974
157.7601
157.5364
157.2521
157.1394
156.7792
151.1727
138.4080
130.5852
130.4831
122.9844
122.9566
118.2495
112.5805
112.1965
105.7536
105.5448
103.0685
102.8330
39.8930
Compound 3
F
OH
HN
N
N
N
ELG-000021
Chemical Formula: C16H13FN4O
DMSO-d6
V2517-056D1
39.8930
45 44 43 42 41 40 39 38 37 36
f1 (ppm)
190 180 170 160 150 140 130 120 110 100 90 80 70 60 50 40 30 20 10
f1 (ppm)

Injection Summary Report

SAMPLE INFORMATION

Sample Name:	V2517-056D1	Acquired By:	System
Sample Type:	Standard	Sample Set Name	20200801
Vial:	2:A,3	Acq. Method Set:	VIVA QC_WATERS BEH C18
Injection #:	1	Processing Method	214, Process standrads method
Injection Volume:	0.20 ul	Channel Name:	PDA Ch3 214nm@4.8nm, PDA
Run Time:	15.0 Minutes	Proc. Chnl. Descr.:	PDA Ch3 214nm@4.8nm, PDA

Date Acquired: 8/3/2020 9:55:43 AM CST
Date Processed: 8/3/2020 10:16:41 AM CST, 8/3/2020 10:16:50 AM CST, 8/3/2020 10:17:09 AM CST

Compound 3

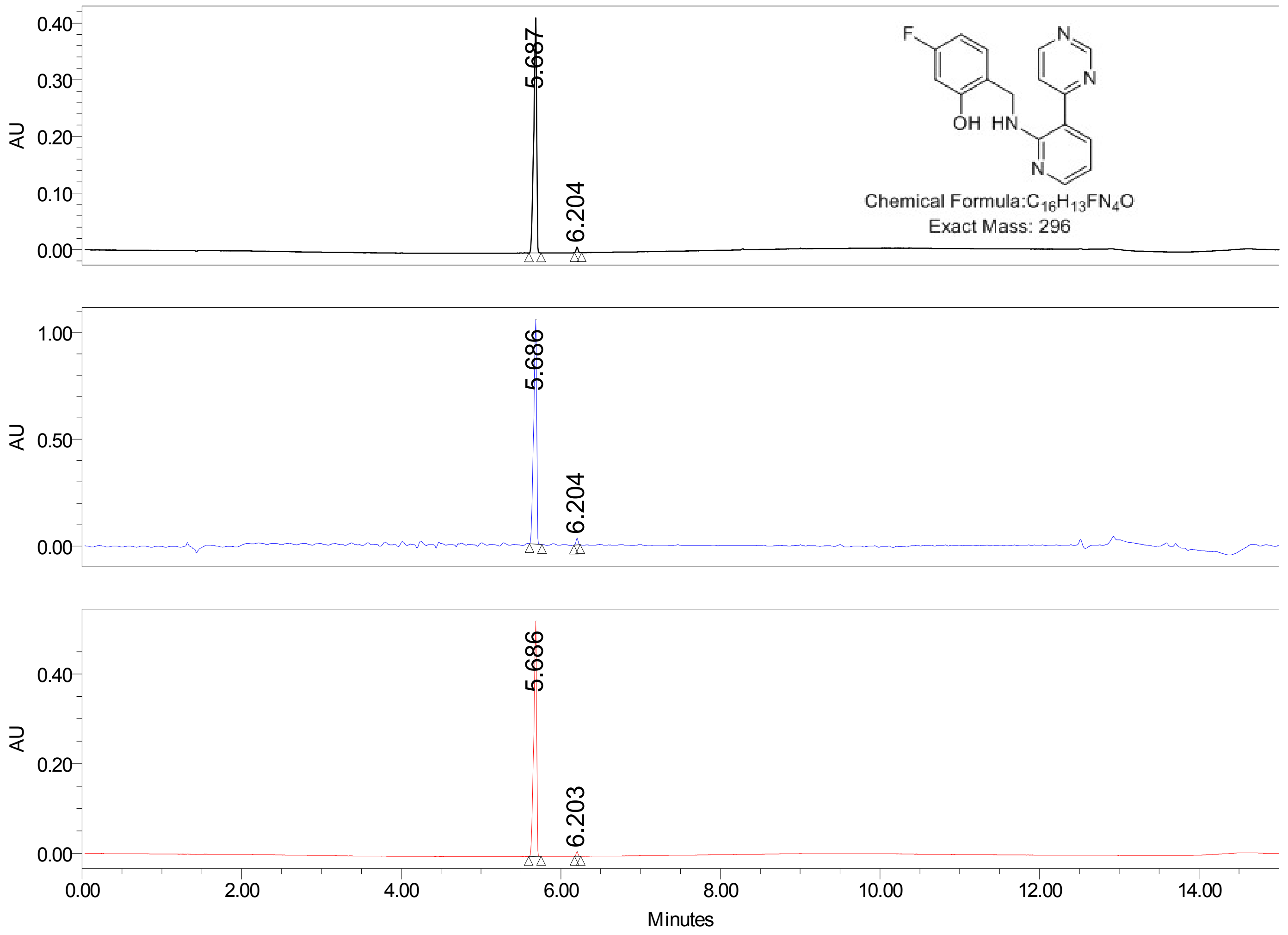

Channel: PDA Ch1 254nm@4.8nm; Processed Channel: PDA Ch1 254nm@4.8nm; Result Id: 4902; Processing Method: Process standrads method

Channel: PDA Ch3 214nm@4.8nm; Processed Channel: PDA Ch3 214nm@4.8nm; Result Id: 4904; Processing Method: 214

Channel: PDA Ch2 280nm@4.8nm; Processed Channel: PDA Ch2 280nm@4.8nm; Result Id: 4903; Processing Method: Process standrads method

Compound 4

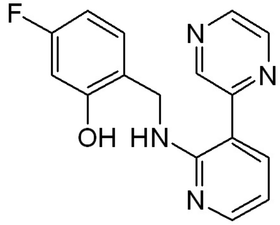

V2398-018
DMSO

Parameter	Value
1 Title	V2398-018
2 Origin	Bruker BioSpin GmbH
3 Solvent	DMSO
4 Temperature	298.0
5 Number of Scans	16
6 Spectrometer Frequency	400.13

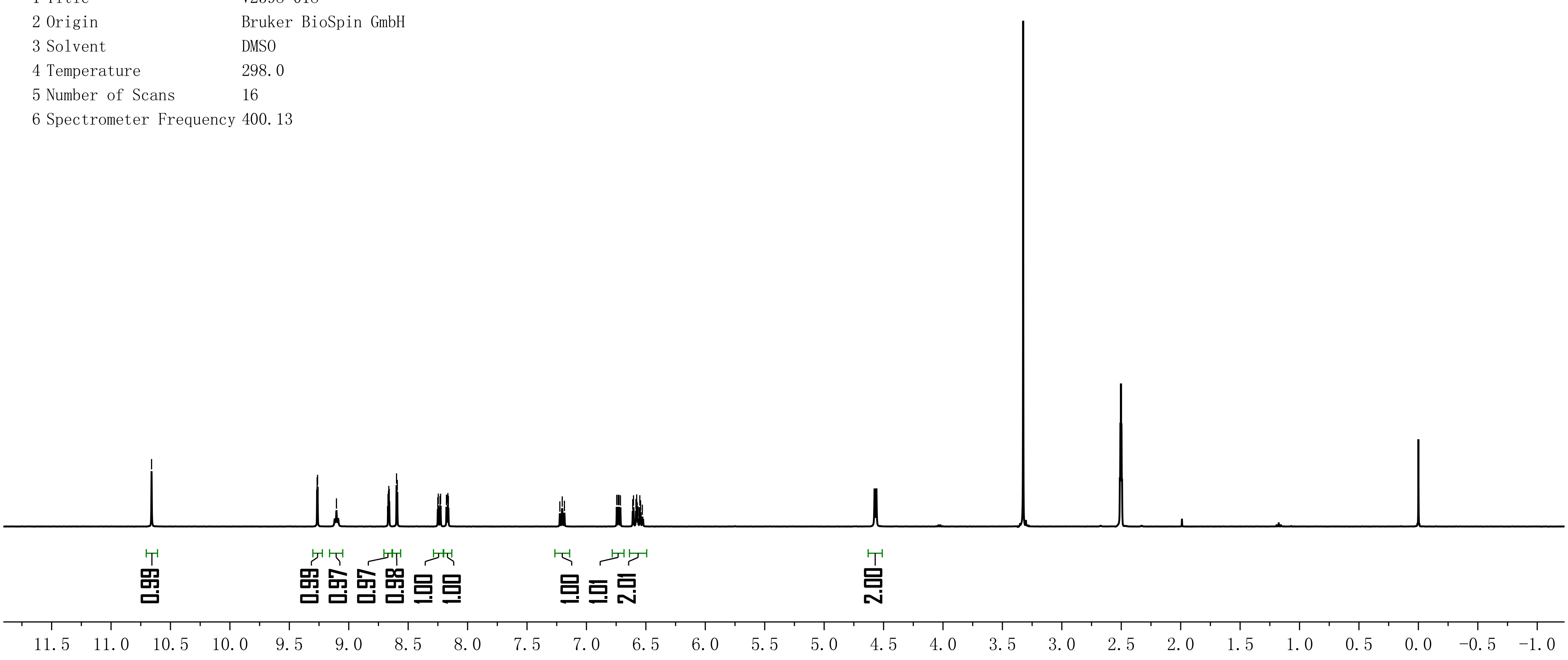

Injection Summary Report

SAMPLE INFORMATION

Sample Name:	V2398-018	Acquired By:	System
Sample Type:	Standard	Sample Set Name	20200617
Vial:	2:D,1	Acq. Method Set:	VIVA QC_WATERS BEH C18
Injection #:	1	Processing Method	214, Process standrads method
Injection Volume:	0.50 ul	Channel Name:	PDA Ch3 214nm@4.8nm, PDA
Run Time:	15.0 Minutes	Proc. Chnl. Descr.:	PDA Ch3 214nm@4.8nm, PDA

Date Acquired: 6/17/2020 9:33:44 AM CST
Date Processed: 6/17/2020 10:09:00 AM CST, 6/17/2020 10:09:10 AM CST, 6/17/2020 10:09:41 AM

Compound 4

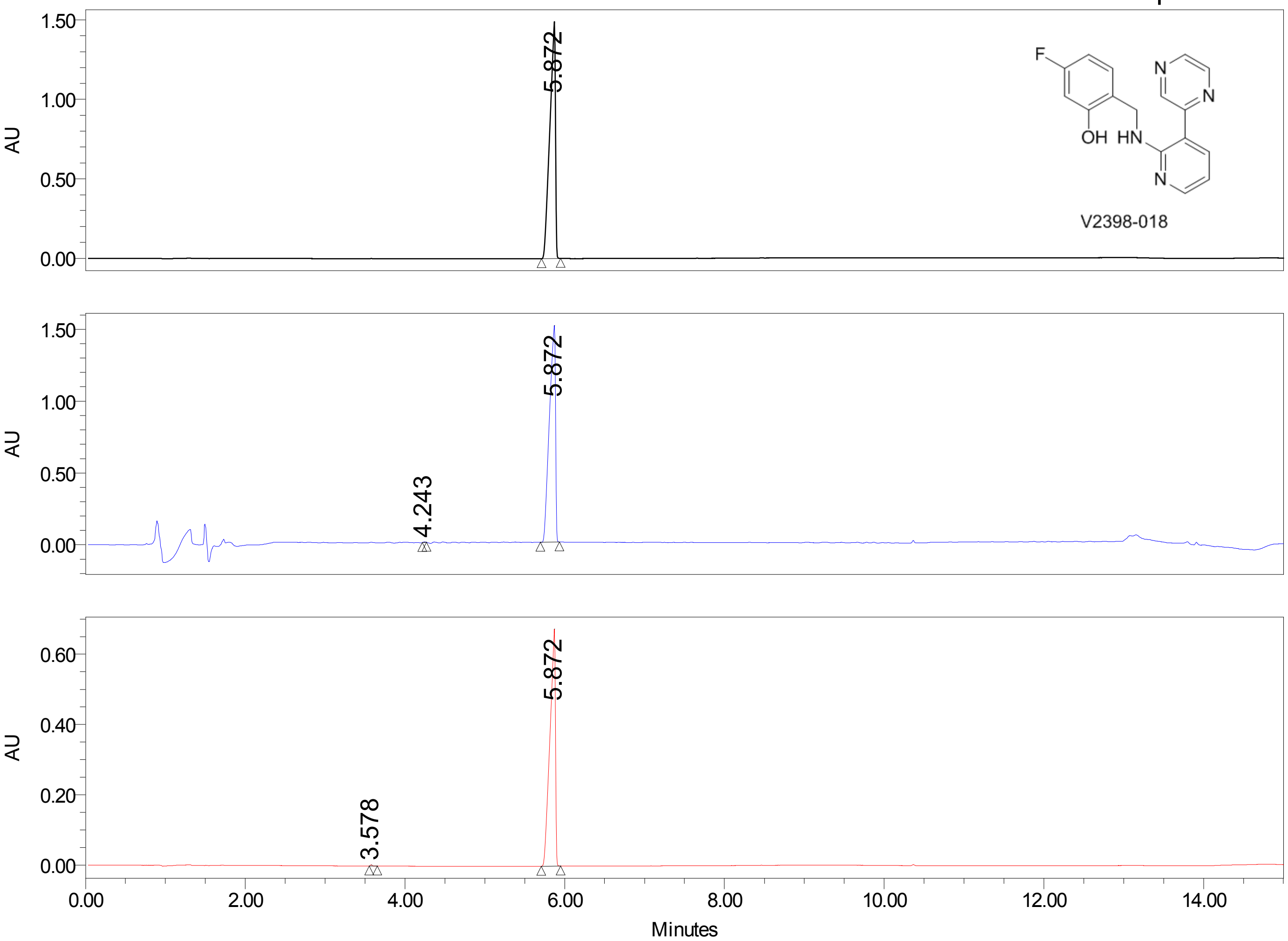

Channel: PDA Ch1 254nm@4.8nm; Processed Channel: PDA Ch1 254nm@4.8nm; Result Id: 14704; Processing Method: Process standrads method

Channel: PDA Ch3 214nm@4.8nm; Processed Channel: PDA Ch3 214nm@4.8nm; Result Id: 14706; Processing Method: 214

Channel: PDA Ch2 280nm@4.8nm; Processed Channel: PDA Ch2 280nm@4.8nm; Result Id: 14705; Processing Method: Process standrads method

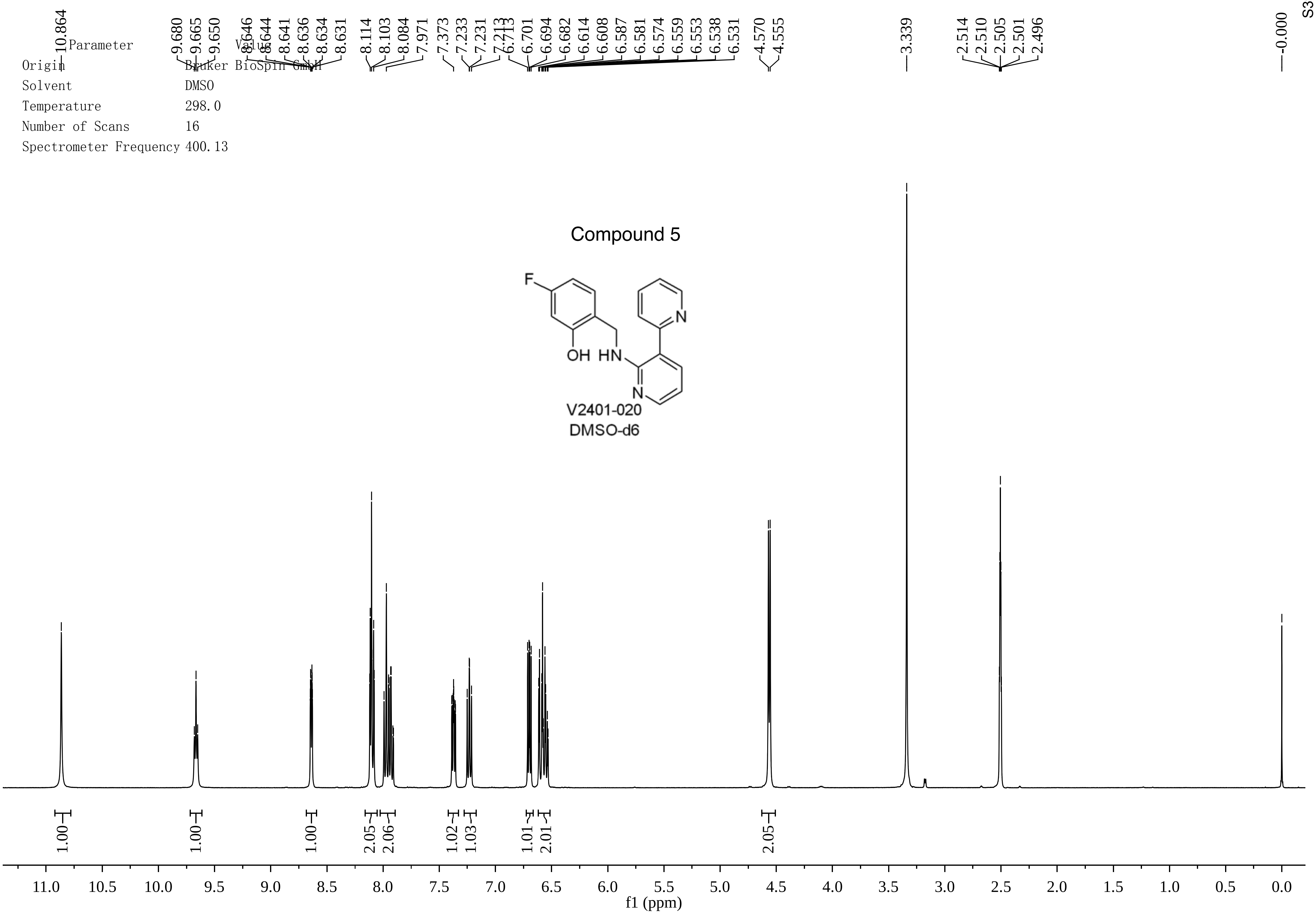
Parameter Value
Origin Bruker BioSpin GmbH
Solvent DMSO
Temperature 298.0
Number of Scans 16
Spectrometer Frequency 400.13
Compound 5
V2401-020
DMSO-d6
F
OH HN
N
N
10.864
9.680
9.665
9.650
8.646
8.644
8.641
8.636
8.634
8.631
8.114
8.103
8.084
7.971
7.373
7.233
7.231
7.213
6.713
6.701
6.694
6.682
6.614
6.608
6.587
6.581
6.574
6.559
6.553
6.538
6.531
4.570
4.555
3.339
2.514
2.510
2.505
2.501
2.496
-0.000
1.00
1.00
1.00
2.05
2.06
1.02
1.03
1.01
2.01
2.05
11.0 10.5 10.0 9.5 9.0 8.5 8.0 7.5 7.0 6.5 6.0 5.5 5.0 4.5 4.0 3.5 3.0 2.5 2.0 1.5 1.0 0.5 0.0
f1 (ppm)

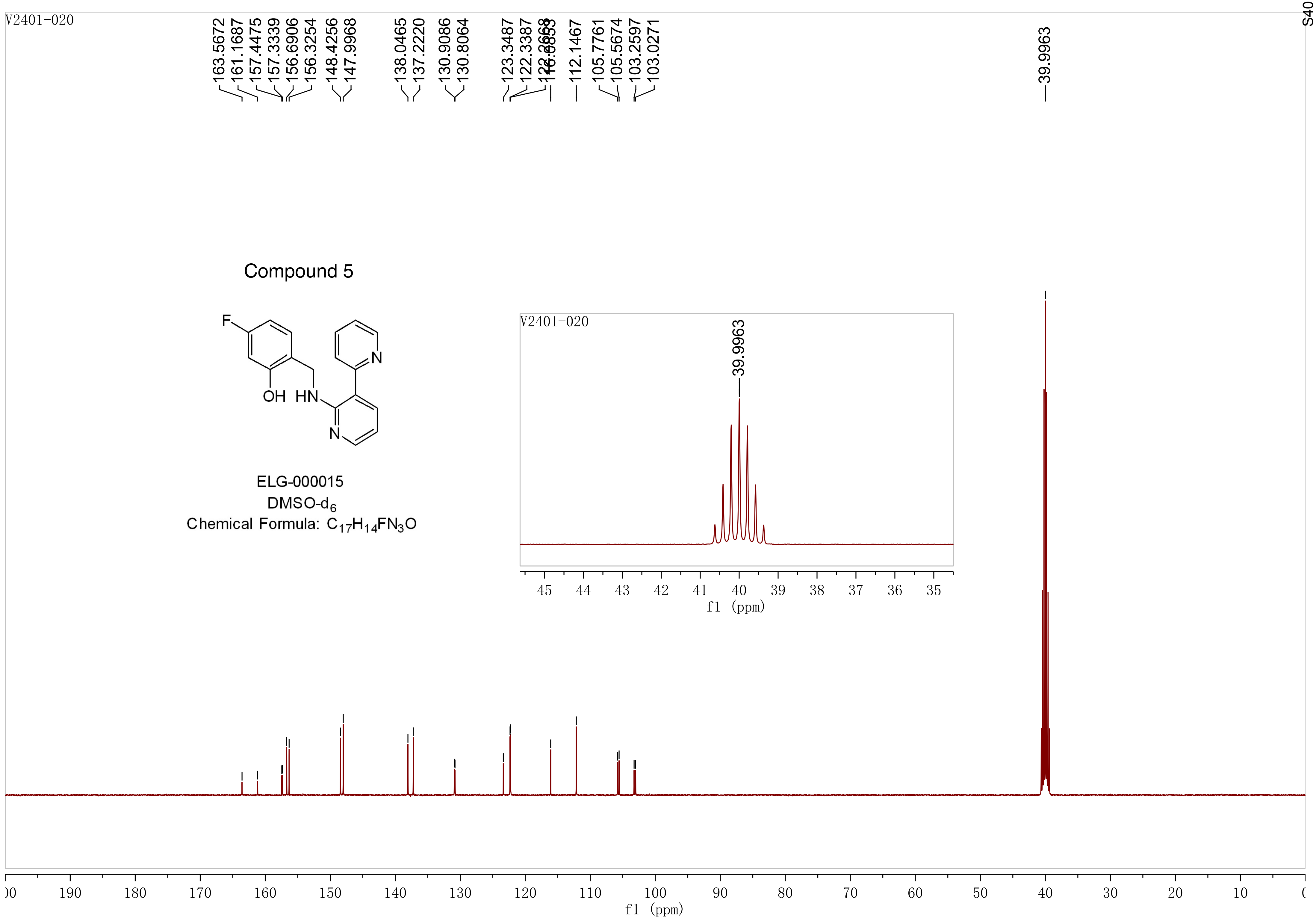
V2401-020
163.5672
161.1687
157.4475
157.3339
156.6906
156.3254
148.4256
147.9968
138.0465
137.2220
130.9086
130.8064
123.3487
122.3387
122.2668
116.6853
112.1467
105.7761
105.5674
103.2597
103.0271
39.9963
Compound 5
F
OH
HN
N
N
ELG-000015
DMSO-d6
Chemical Formula: C17H14FN3O
V2401-020
39.9963
45 44 43 42 41 40 39 38 37 36 35
f1 (ppm)
190 180 170 160 150 140 130 120 110 100 90 80 70 60 50 40 30 20 10
f1 (ppm)

Injection Summary Report

SAMPLE INFORMATION

Sample Name:	V2401-020	Acquired By:	System
Sample Type:	Standard	Sample Set Name	20200622
Vial:	1:C,5	Acq. Method Set:	VIVA QC_WATERS BEH C18
Injection #:	1	Processing Methoc	214, Process standrads method
Injection Volume:	0.50 ul	Channel Name:	PDA Ch3 214nm@4.8nm, PDA
Run Time:	15.0 Minutes	Proc. Chnl. Descr.:	PDA Ch3 214nm@4.8nm, PDA

Date Acquired: 6/22/2020 12:59:38 PM CST
Date Processed: 6/22/2020 1:20:44 PM CST, 6/22/2020 1:20:56 PM CST, 6/22/2020 1:21:11 PM CST

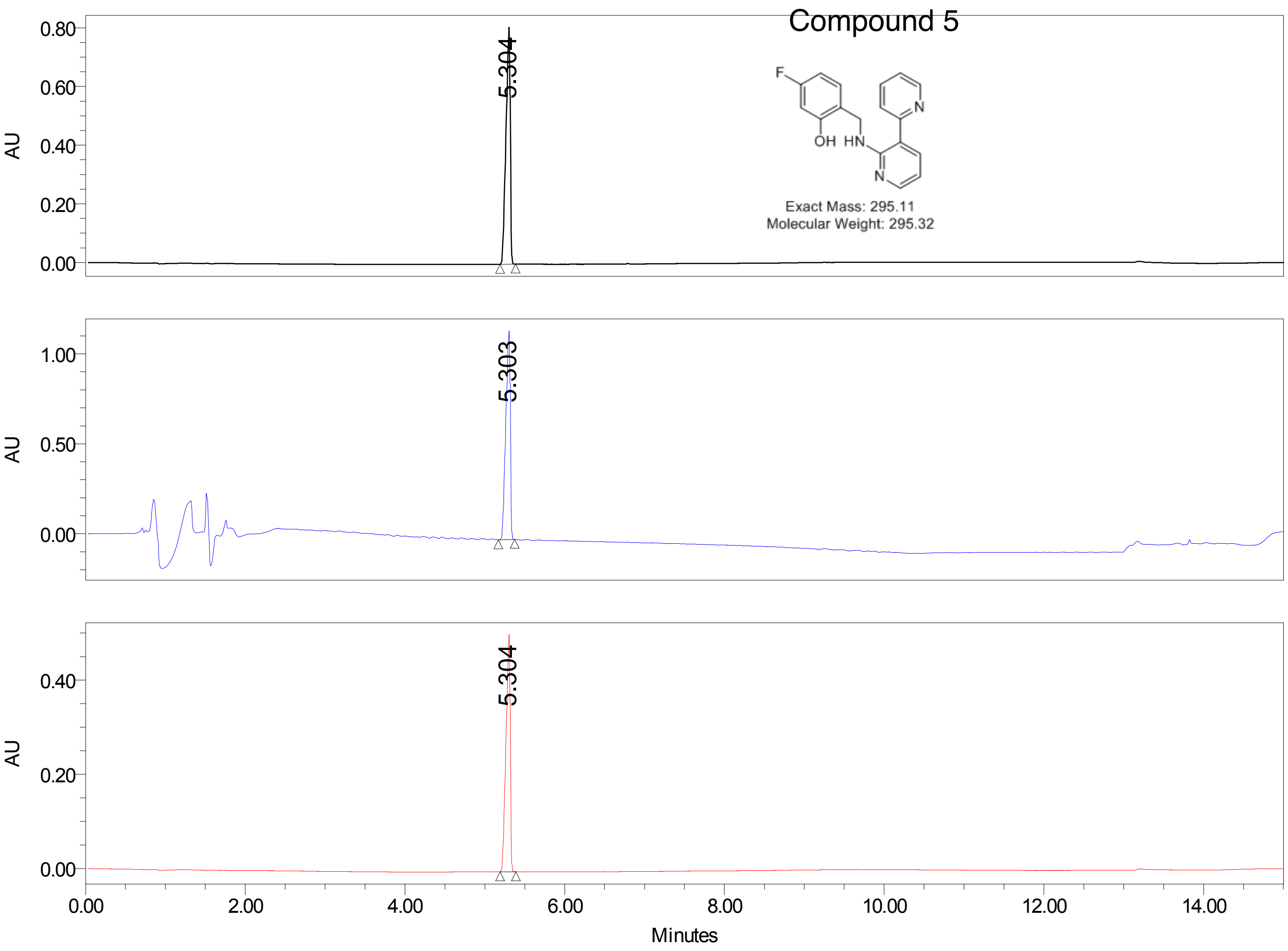

Channel: PDA Ch1 254nm@4.8nm; Processed Channel: PDA Ch1 254nm@4.8nm; Result Id: 15571; Processing Method: Process standrads method
Channel: PDA Ch3 214nm@4.8nm; Processed Channel: PDA Ch3 214nm@4.8nm; Result Id: 15573; Processing Method: 214
Channel: PDA Ch2 280nm@4.8nm; Processed Channel: PDA Ch2 280nm@4.8nm; Result Id: 15572; Processing Method: Process standrads method

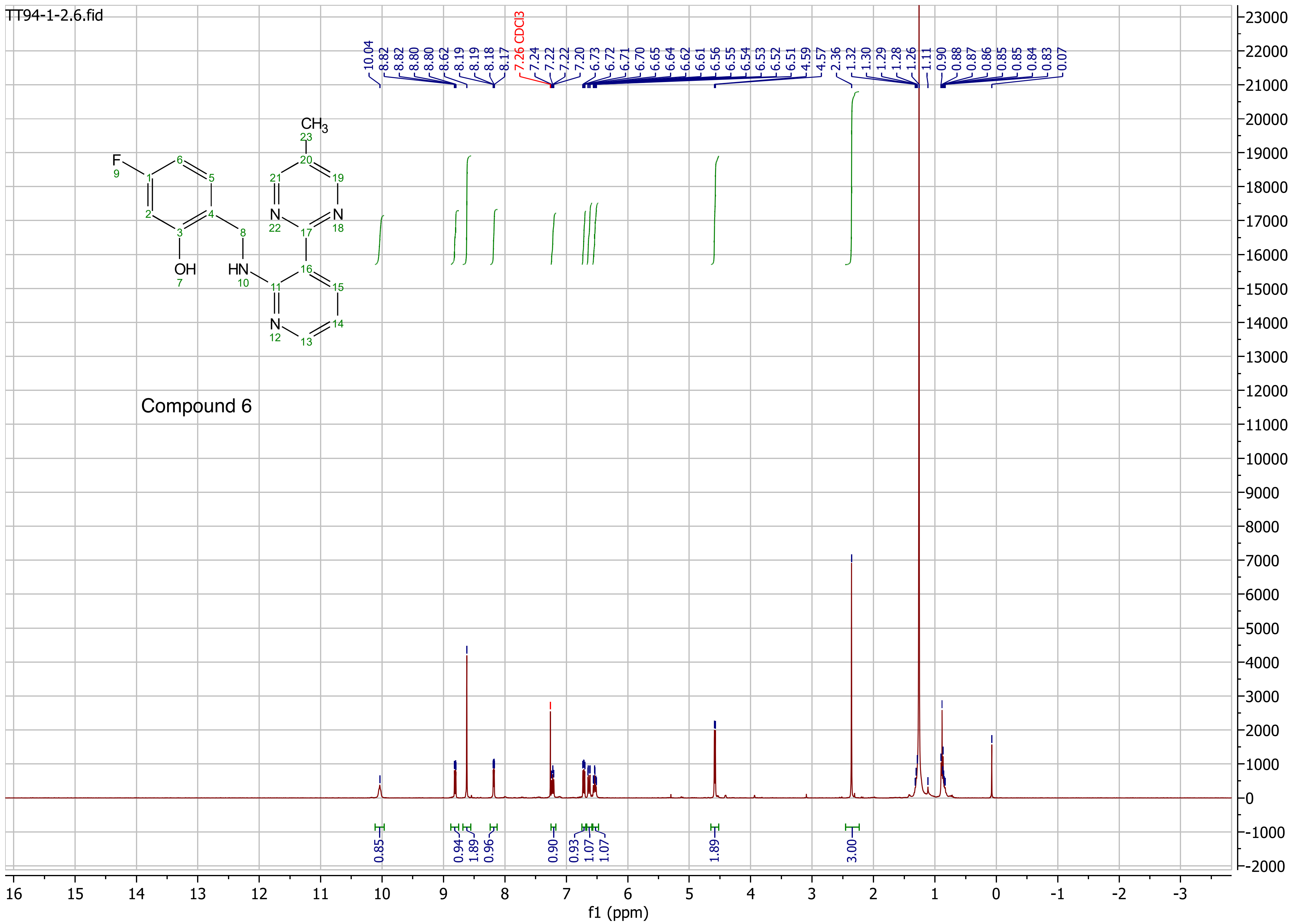
TT94-1-2.6.fid
Compound 6
F
OH
HN
N
CH3
10.04
8.82
8.82
8.80
8.80
8.62
8.19
8.19
8.18
8.17
7.26 CDCl3
7.24
7.22
7.22
7.20
6.73
6.72
6.71
6.70
6.65
6.64
6.62
6.61
6.56
6.55
6.54
6.53
6.52
6.51
4.59
4.57
2.36
1.32
1.30
1.29
1.28
1.26
1.11
0.90
0.88
0.87
0.86
0.85
0.85
0.84
0.83
0.07
0.85
0.94
1.89
0.96
0.90
0.93
1.07
1.07
1.89
3.00
f1 (ppm)

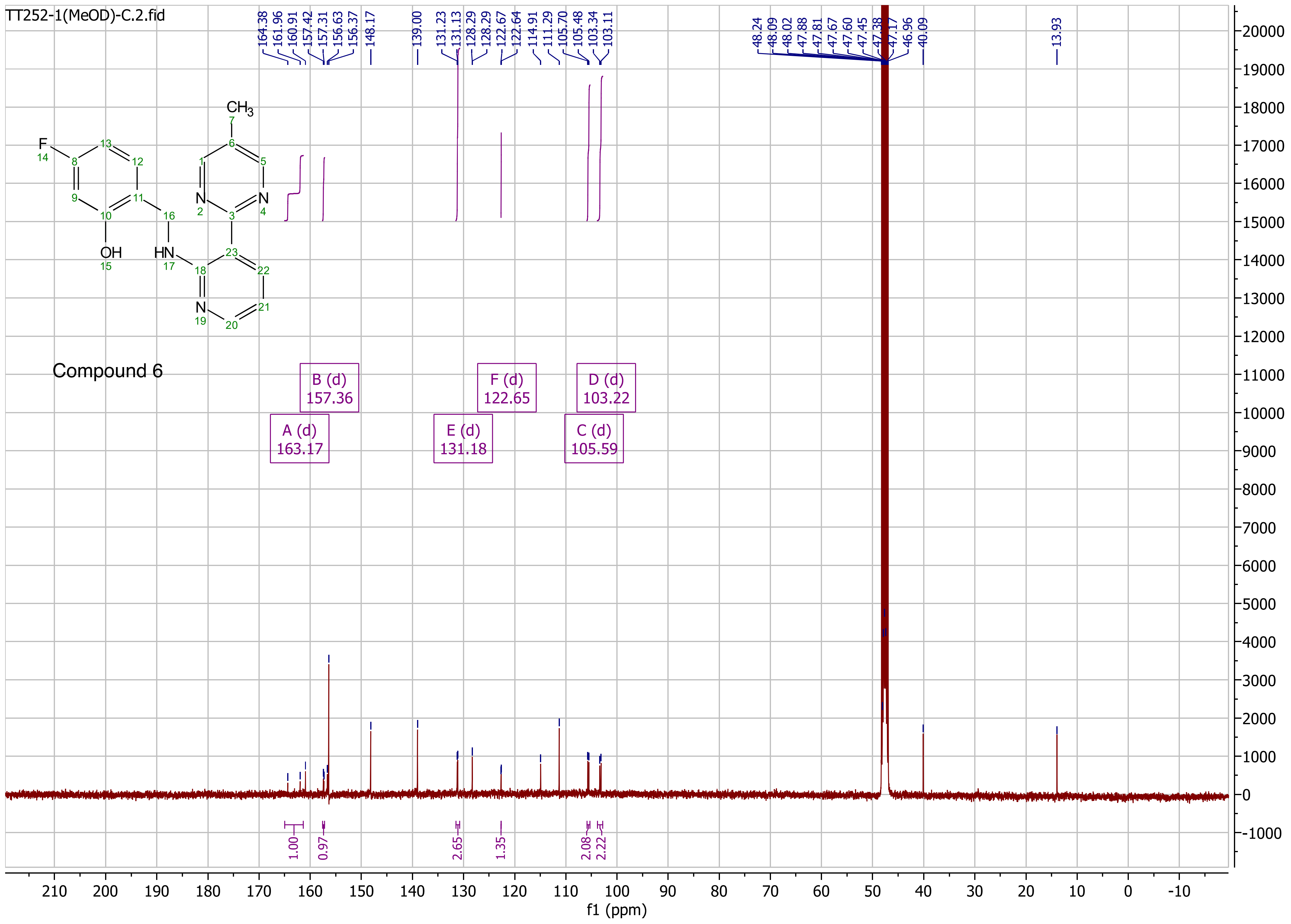
TT252-1(MeOD)-C.2.fid
164.38
161.96
160.91
157.42
157.31
156.63
156.37
148.17
139.00
131.23
131.13
128.29
128.29
122.67
122.64
114.91
111.29
105.70
105.48
103.34
103.11
48.24
48.09
48.02
47.88
47.81
47.67
47.60
47.45
47.38
47.17
46.96
40.09
13.93
Compound 6
A (d) 163.17
B (d) 157.36
E (d) 131.18
F (d) 122.65
C (d) 105.59
D (d) 103.22
1.00
0.97
2.65
1.35
2.08
2.22
f1 (ppm)

Compound 6

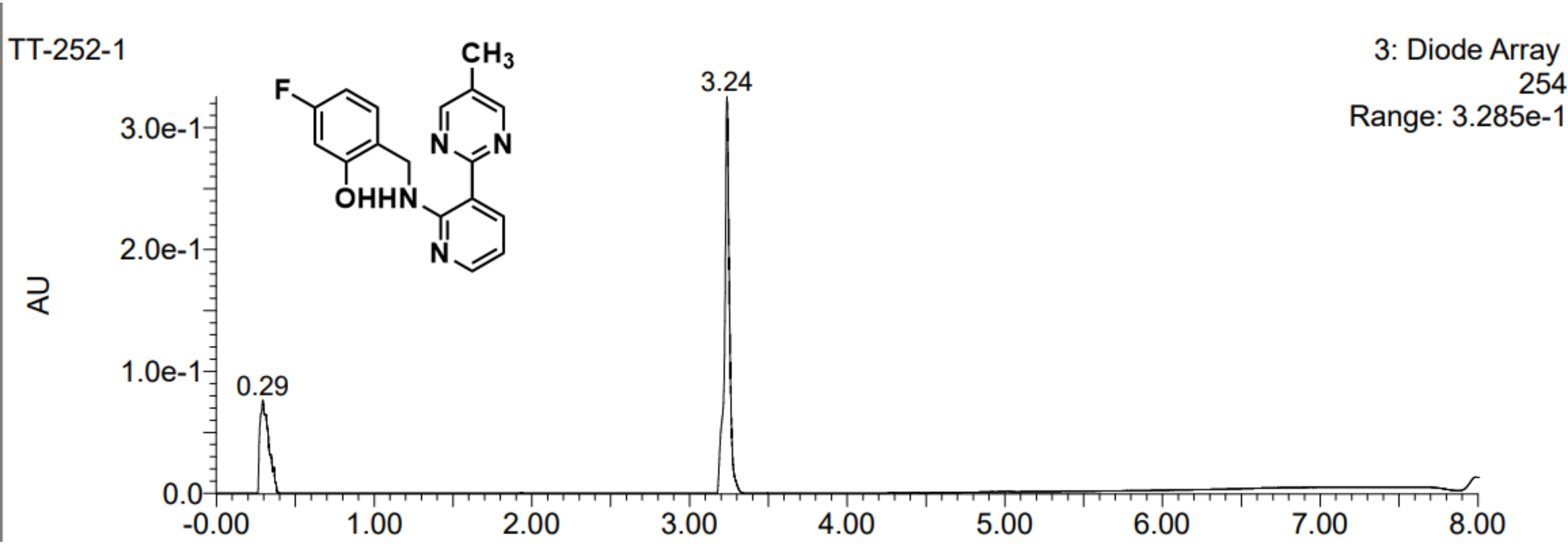
TT-252-1
3: Diode Array
254
Range: 3.285e-1
AU
3.0e-1
2.0e-1
1.0e-1
0.0
3.24
0.29
-0.00
1.00
2.00
3.00
4.00
5.00
6.00
7.00
8.00
F
OHHN
N
N
N
CH3

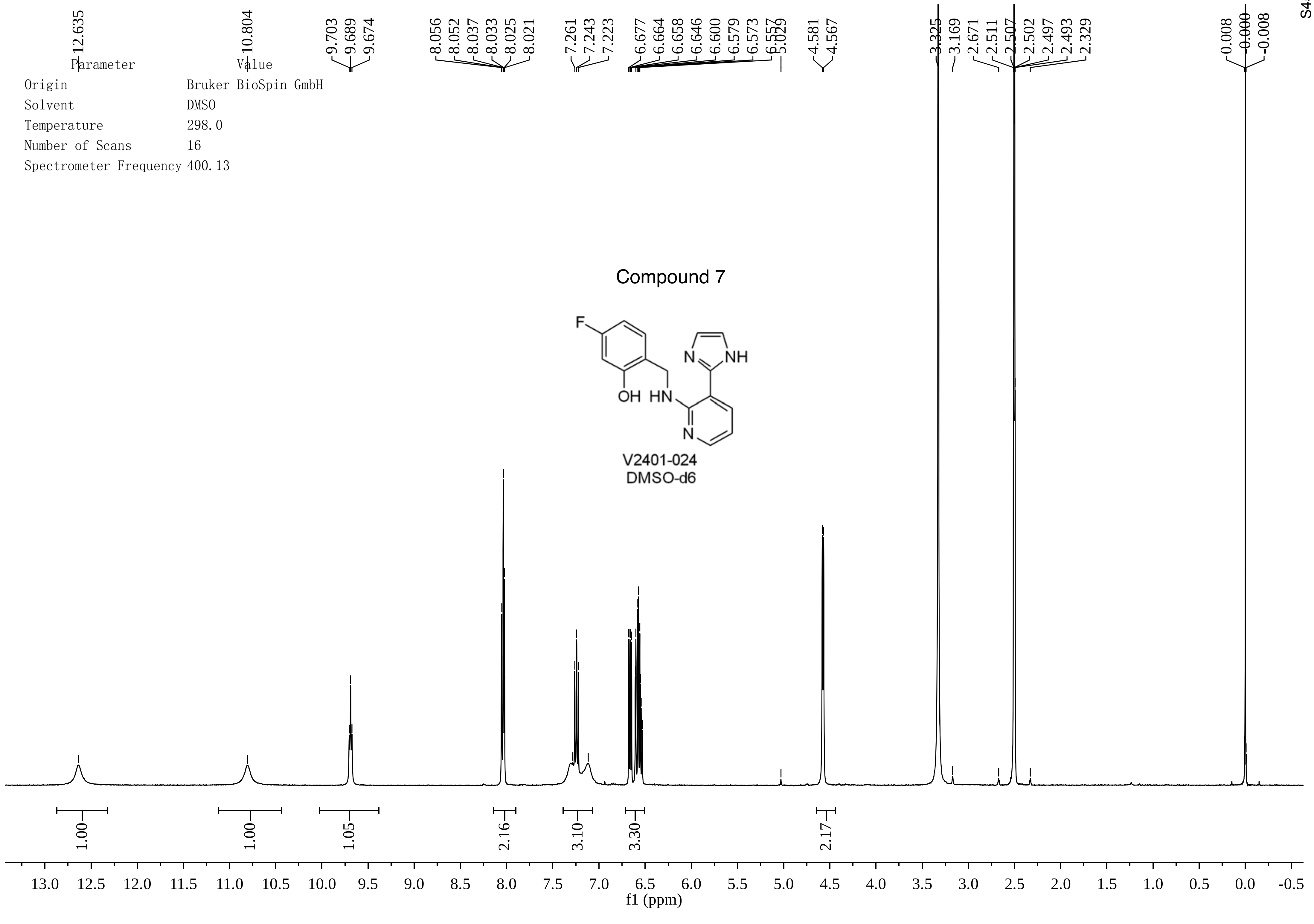

Parameter Value
Origin Bruker BioSpin GmbH
Solvent DMSO
Temperature 298.0
Number of Scans 16
Spectrometer Frequency 400.13
12.635
10.804
9.703
9.689
9.674
8.056
8.052
8.037
8.033
8.025
8.021
7.261
7.243
7.223
6.677
6.664
6.658
6.646
6.600
6.579
6.573
6.557
5.029
4.581
4.567
3.325
3.169
2.671
2.511
2.507
2.502
2.497
2.493
2.329
0.008
0.000
-0.008
Compound 7
F
OH
HN
N
NH
V2401-024
DMSO-d6
1.00
1.00
1.05
2.16
3.10
3.30
2.17
13.0 12.5 12.0 11.5 11.0 10.5 10.0 9.5 9.0 8.5 8.0 7.5 7.0 6.5 6.0 5.5 5.0 4.5 4.0 3.5 3.0 2.5 2.0 1.5 1.0 0.5 0.0 -0.5
f1 (ppm)

Compound 7

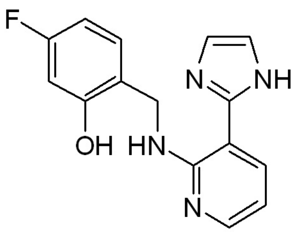

ELG-000002
DMSO-d_6
Chemical Formula: $C_{15}H_{13}FN_4O$

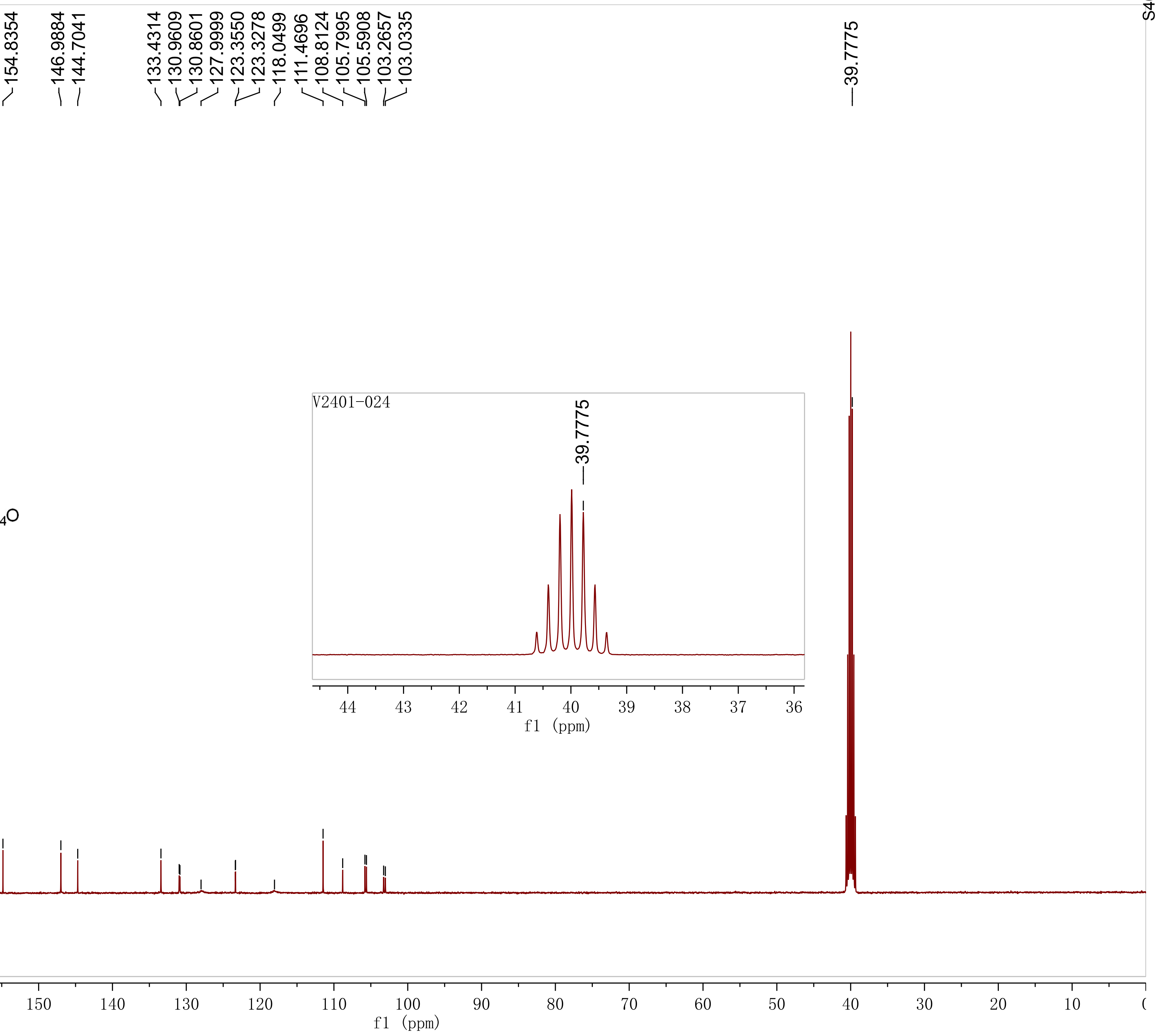

Injection Summary Report

SAMPLE INFORMATION

Sample Name:	V2401-024	Acquired By:	System
Sample Type:	Standard	Sample Set Name	20200617
Vial:	2:D,6	Acq. Method Set:	VIVA QC_WATERS BEH C18
Injection #:	1	Processing Method	214, Process standrads method
Injection Volume:	0.50 ul	Channel Name:	PDA Ch3 214nm@4.8nm, PDA
Run Time:	15.0 Minutes	Proc. Chnl. Descr.:	PDA Ch3 214nm@4.8nm, PDA

Date Acquired: 6/17/2020 2:15:43 PM CST
Date Processed: 6/17/2020 3:07:48 PM CST, 6/17/2020 3:08:06 PM CST, 6/17/2020 3:08:21 PM CST

Compound 7

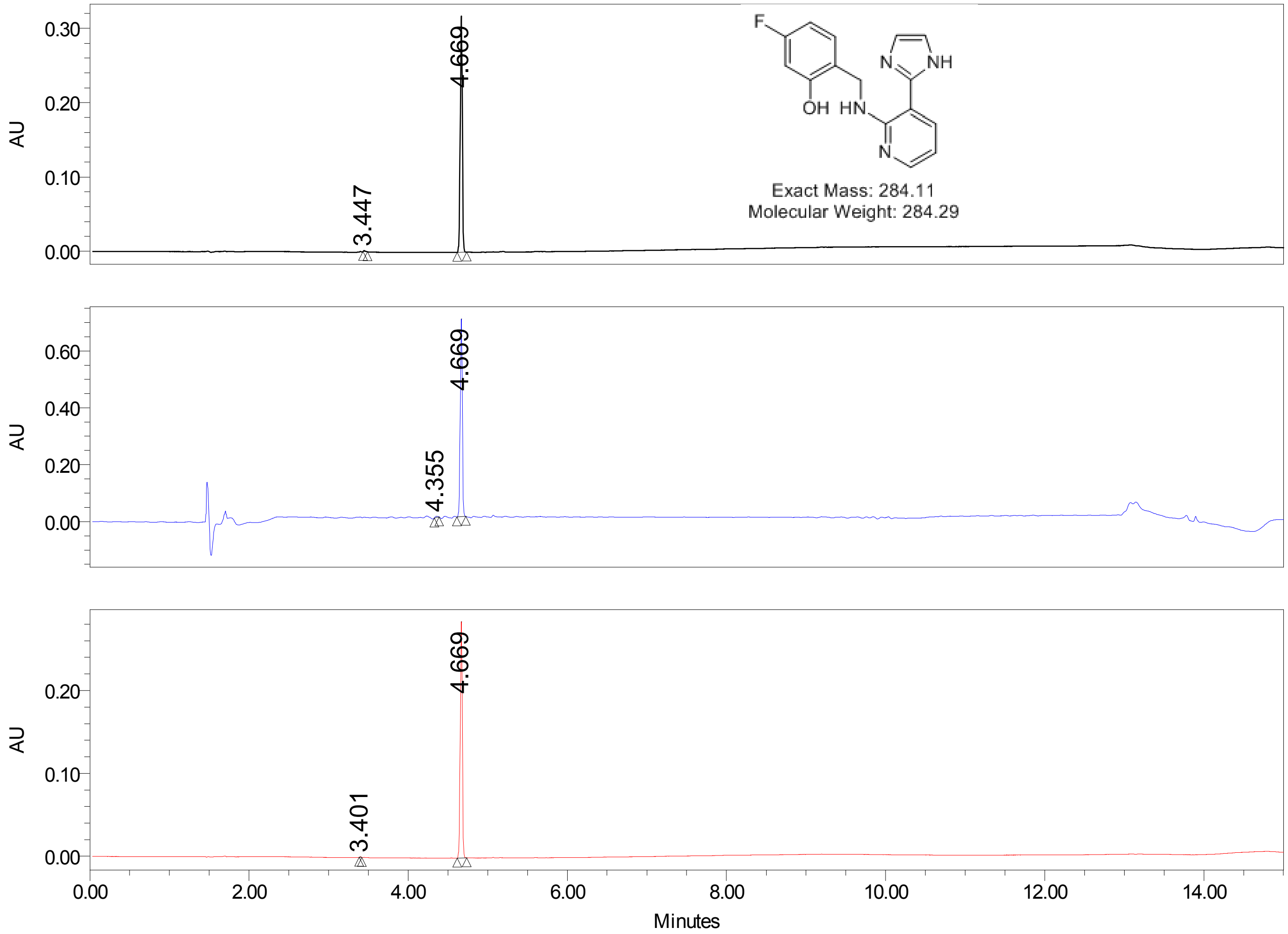

Channel: PDA Ch1 254nm@4.8nm; Processed Channel: PDA Ch1 254nm@4.8nm; Result Id: 14766; Processing Method: Process standrads method

Channel: PDA Ch3 214nm@4.8nm; Processed Channel: PDA Ch3 214nm@4.8nm; Result Id: 14768; Processing Method: 214

Channel: PDA Ch2 280nm@4.8nm; Processed Channel: PDA Ch2 280nm@4.8nm; Result Id: 14767; Processing Method: Process standrads method

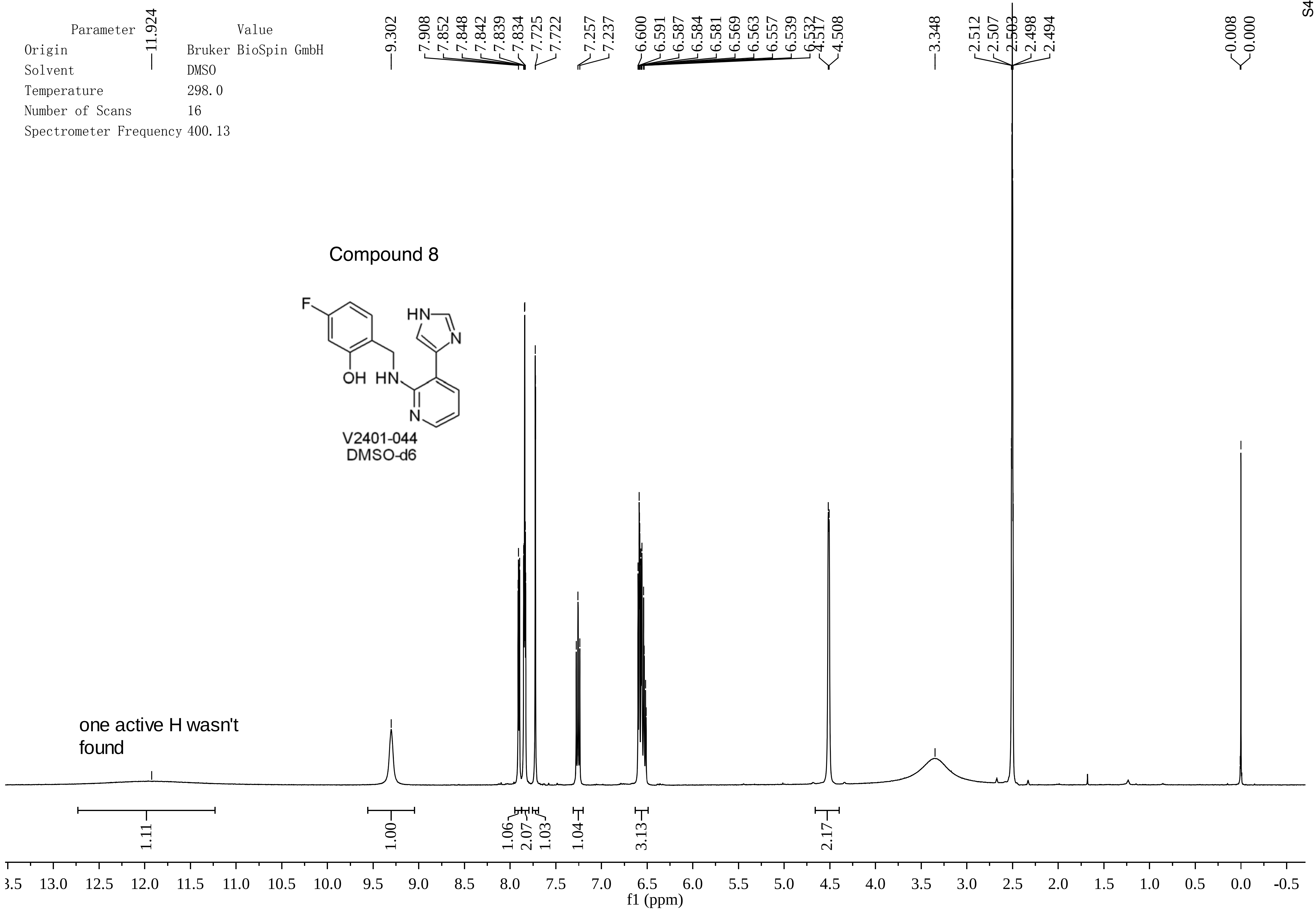
Parameter Value
Origin Bruker BioSpin GmbH
Solvent DMSO
Temperature 298.0
Number of Scans 16
Spectrometer Frequency 400.13
Compound 8
V2401-044
DMSO-d6
one active H wasn't found
11.924
9.302
7.908
7.852
7.848
7.842
7.839
7.834
7.725
7.722
7.257
7.237
6.600
6.591
6.587
6.584
6.581
6.569
6.563
6.557
6.539
6.532
4.517
4.508
3.348
2.512
2.507
2.503
2.498
2.494
0.008
0.000
1.11
1.00
1.06
2.07
1.03
1.04
3.13
2.17
f1 (ppm)

Injection Summary Report

SAMPLE INFORMATION

Sample Name:	V2401-044	Acquired By:	System
Sample Type:	Standard	Sample Set Name	20200629
Vial:	1:A,5	Acq. Method Set:	VIVA QC_WATERS BEH C18
Injection #:	1	Processing Method	214, Process standrads method
Injection Volume:	0.50 ul	Channel Name:	PDA Ch3 214nm@4.8nm, PDA
Run Time:	15.0 Minutes	Proc. Chnl. Descr.:	PDA Ch3 214nm@4.8nm, PDA

Date Acquired: 6/29/2020 9:28:57 AM CST
Date Processed: 6/29/2020 9:44:57 AM CST, 6/29/2020 9:45:23 AM CST, 6/29/2020 9:45:42 AM CST

Compound 8

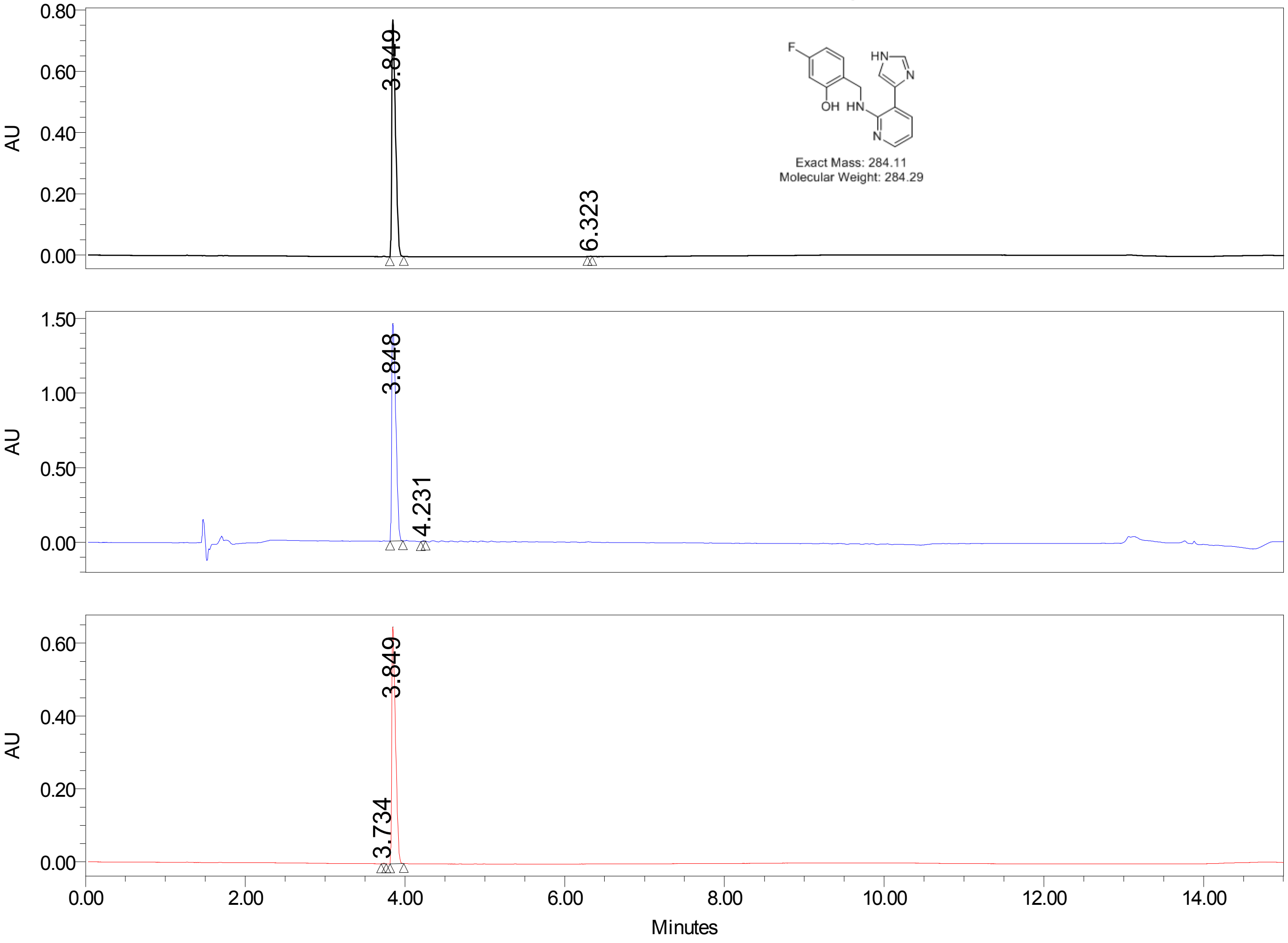

Channel: PDA Ch1 254nm@4.8nm; Processed Channel: PDA Ch1 254nm@4.8nm; Result Id: 16290; Processing Method: Process standrads method

Channel: PDA Ch3 214nm@4.8nm; Processed Channel: PDA Ch3 214nm@4.8nm; Result Id: 16292; Processing Method: 214

Channel: PDA Ch2 280nm@4.8nm; Processed Channel: PDA Ch2 280nm@4.8nm; Result Id: 16291; Processing Method: Process standrads method

Parameter	Value
Origin	Bruker BioSpin GmbH
Solvent	DMSO
Temperature	298.0
Number of Scans	16
Spectrometer Frequency	400.13

Compound 9

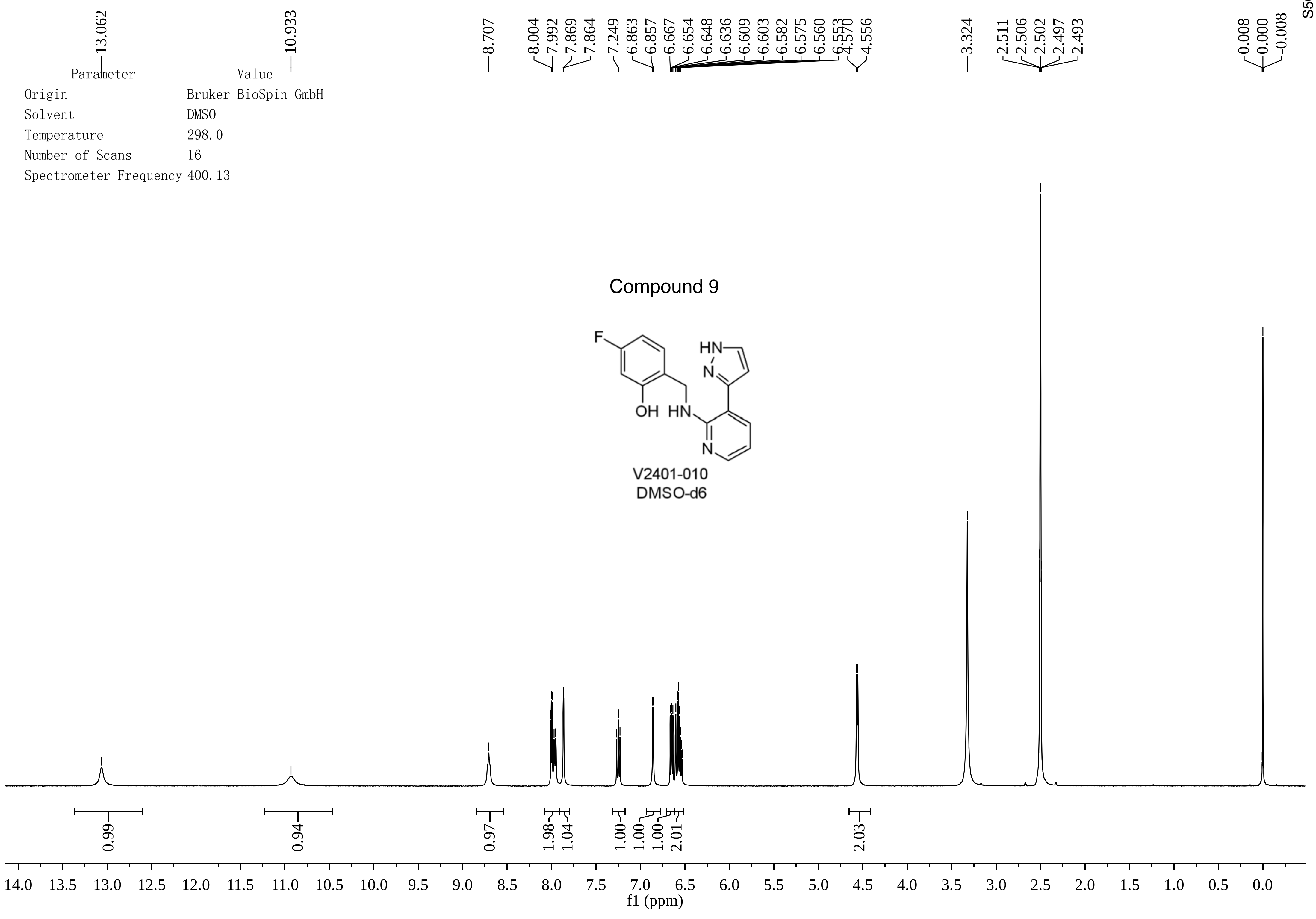

Injection Summary Report

SAMPLE INFORMATION

Sample Name:	V2401-010	Acquired By:	System
Sample Type:	Standard	Sample Set Name	20200608
Vial:	2:E,3	Acq. Method Set:	VIVA QC_WATERS BEH C18
Injection #:	1	Processing Method	VIVA Process standrads method,
Injection Volume:	0.50 ul	Channel Name:	PDA Ch3 214nm@4.8nm, PDA
Run Time:	15.0 Minutes	Proc. Chnl. Descr.:	PDA Ch3 214nm@4.8nm, PDA

Date Acquired: 6/8/2020 9:50:58 AM CST
Date Processed: 6/8/2020 10:23:14 AM CST, 6/8/2020 10:23:33 AM CST, 6/8/2020 10:23:48 AM CST

Compound 9

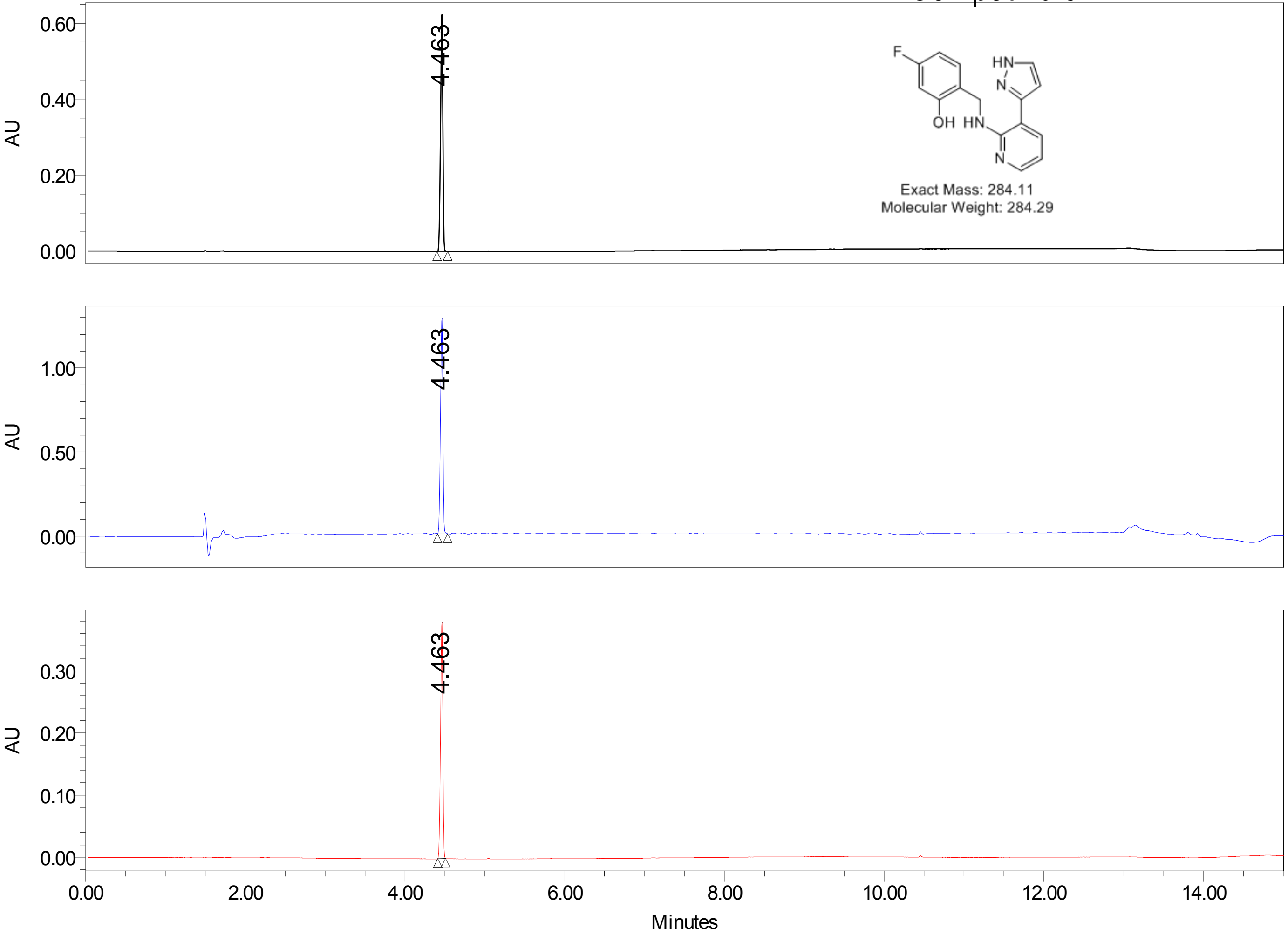

Channel: PDA Ch1 254nm@4.8nm; Processed Channel: PDA Ch1 254nm@4.8nm; Result Id: 13539; Processing Method: VIVA Process standrads method

Channel: PDA Ch3 214nm@4.8nm; Processed Channel: PDA Ch3 214nm@4.8nm; Result Id: 13541; Processing Method: 214 nm standrads method

Channel: PDA Ch2 280nm@4.8nm; Processed Channel: PDA Ch2 280nm@4.8nm; Result Id: 13540; Processing Method: VIVA Process standrads method

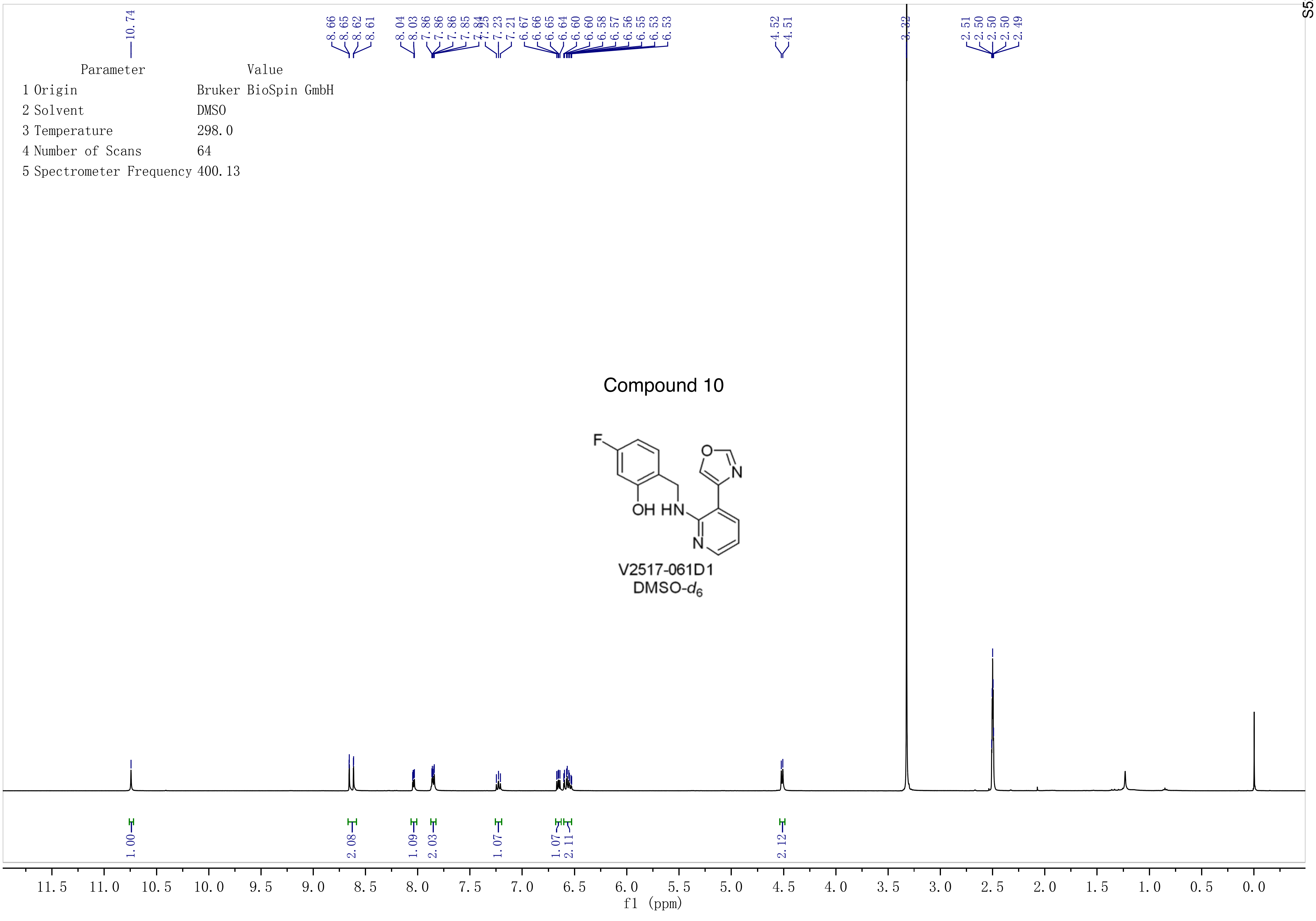
10.74
8.66
8.65
8.62
8.61
8.04
8.03
7.86
7.86
7.86
7.85
7.84
7.25
7.23
7.21
6.67
6.66
6.65
6.64
6.60
6.60
6.58
6.57
6.56
6.55
6.53
6.53
4.52
4.51
3.32
2.51
2.50
2.50
2.50
2.49
Parameter
Value
1 Origin
Bruker BioSpin GmbH
2 Solvent
DMSO
3 Temperature
298.0
4 Number of Scans
64
5 Spectrometer Frequency
400.13
Compound 10
F
OH HN
O
N
N
V2517-061D1
DMSO-d6
1.00
2.08
1.09
2.03
1.07
1.07
2.11
2.12
11.5
11.0
10.5
10.0
9.5
9.0
8.5
8.0
7.5
7.0
6.5
6.0
5.5
5.0
4.5
4.0
3.5
3.0
2.5
2.0
1.5
1.0
0.5
0.0
f1 (ppm)

Compound 10

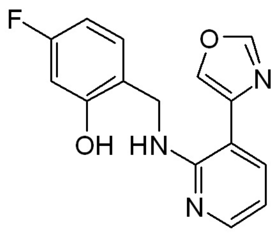

ELG-000023

Chemical Formula: $C_{15}H_{12}FN_3O_2$

DMSO-d_6

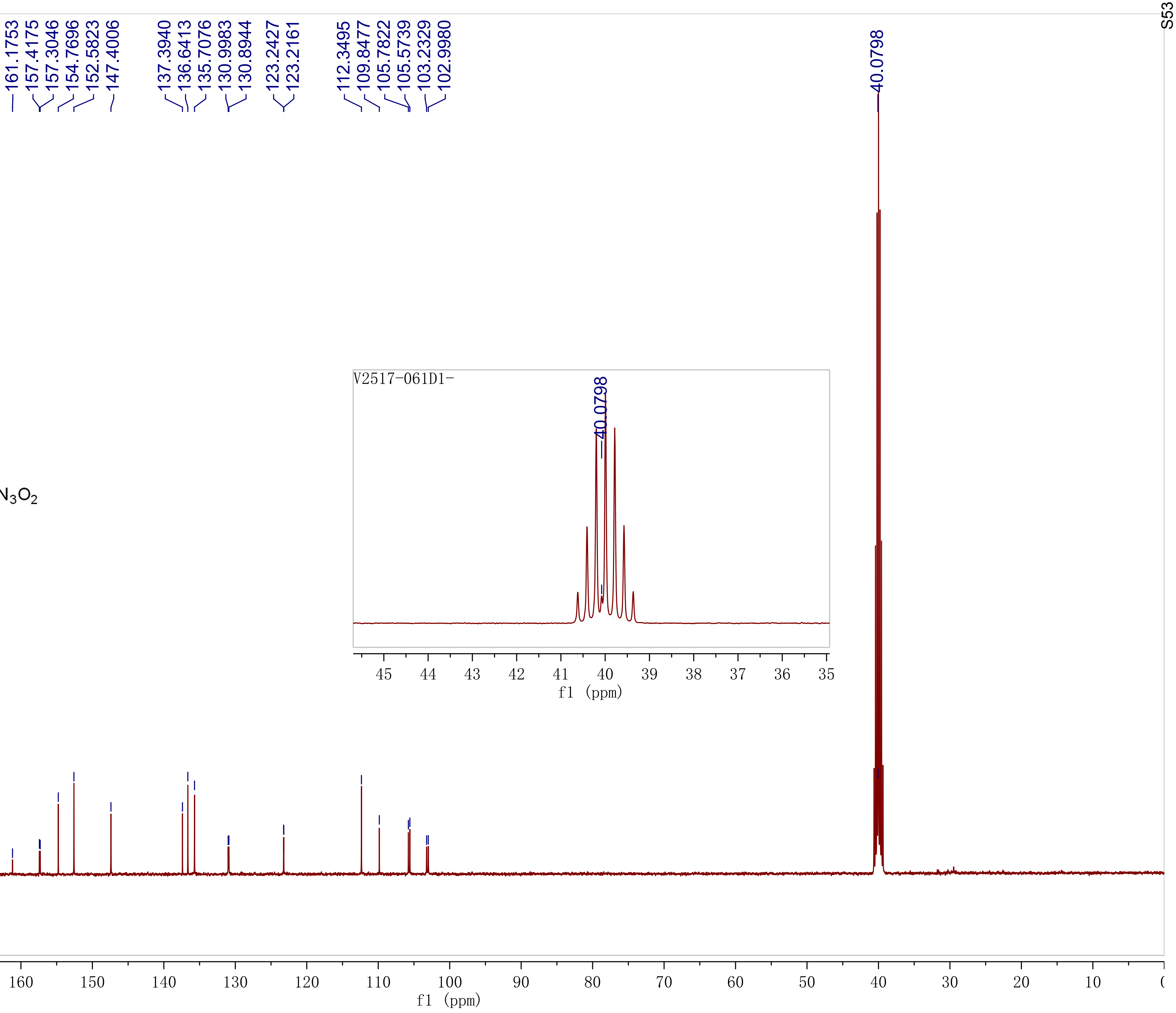

Injection Summary Report

SAMPLE INFORMATION

Sample Name:	V2517-061D1	Acquired By:	System
Sample Type:	Standard	Sample Set Name	20200805
Vial:	2:E,3	Acq. Method Set:	VIVA QC_WATERS BEH C18
Injection #:	1	Processing Method	214, Process standrads method
Injection Volume:	0.50 ul	Channel Name:	PDA Ch3 214nm@4.8nm, PDA
Run Time:	15.0 Minutes	Proc. Chnl. Descr.:	PDA Ch3 214nm@4.8nm, PDA

Date Acquired: 8/5/2020 10:48:55 AM CST
Date Processed: 8/5/2020 11:10:56 AM CST, 8/5/2020 11:11:06 AM CST, 8/5/2020 11:11:18 AM CST

Compound 10

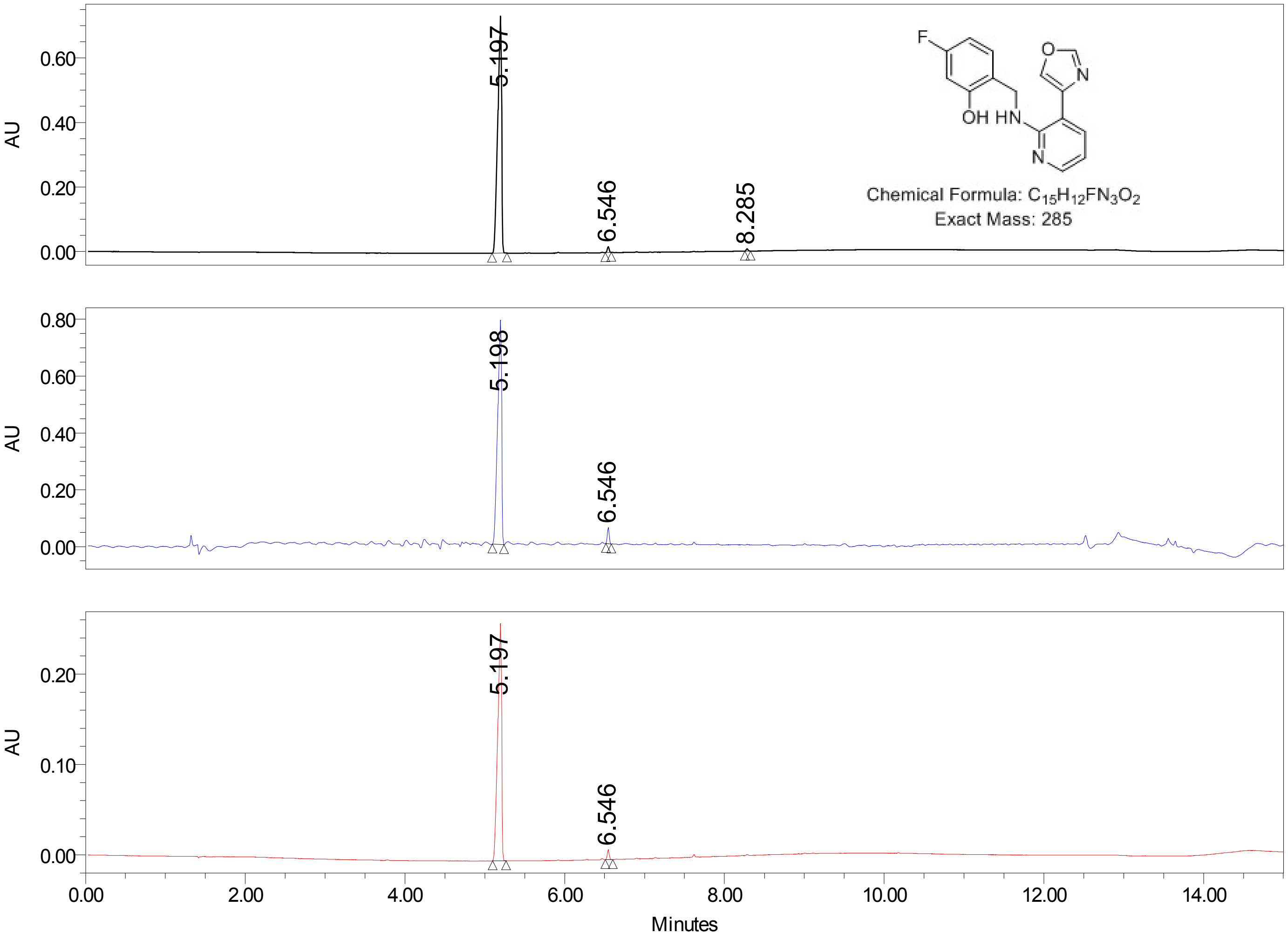

Channel: PDA Ch1 254nm@4.8nm; Processed Channel: PDA Ch1 254nm@4.8nm; Result Id: 5464; Processing Method: Process standrads method
Channel: PDA Ch3 214nm@4.8nm; Processed Channel: PDA Ch3 214nm@4.8nm; Result Id: 5466; Processing Method: 214
Channel: PDA Ch2 280nm@4.8nm; Processed Channel: PDA Ch2 280nm@4.8nm; Result Id: 5465; Processing Method: Process standrads method

Parameter	Value
1 Origin	Bruker BioSpin GmbH
2 Solvent	DMSO
3 Temperature	298.0
4 Number of Scans	64
5 Spectrometer Frequency	400.13

Compound 11

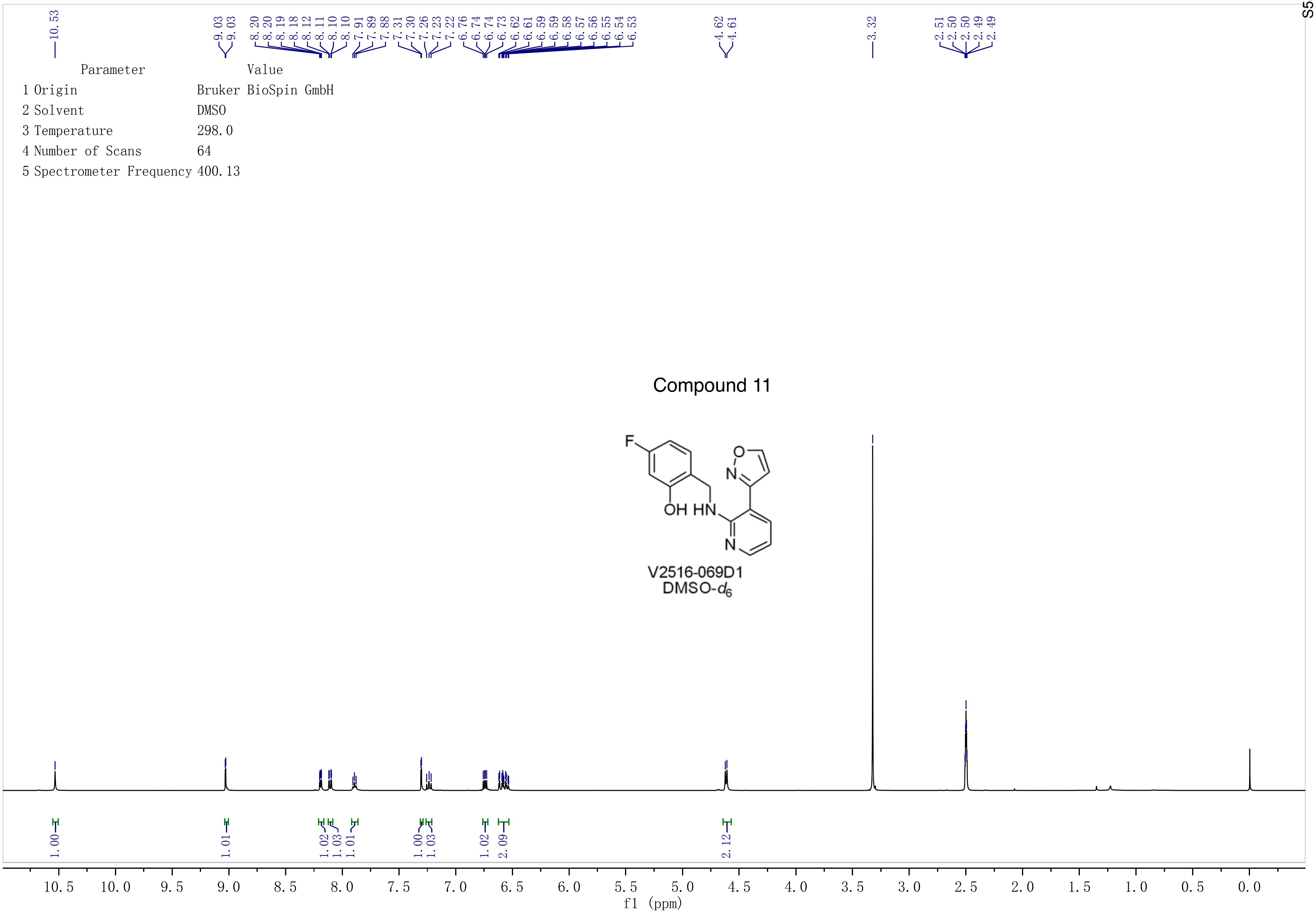

Compound 11

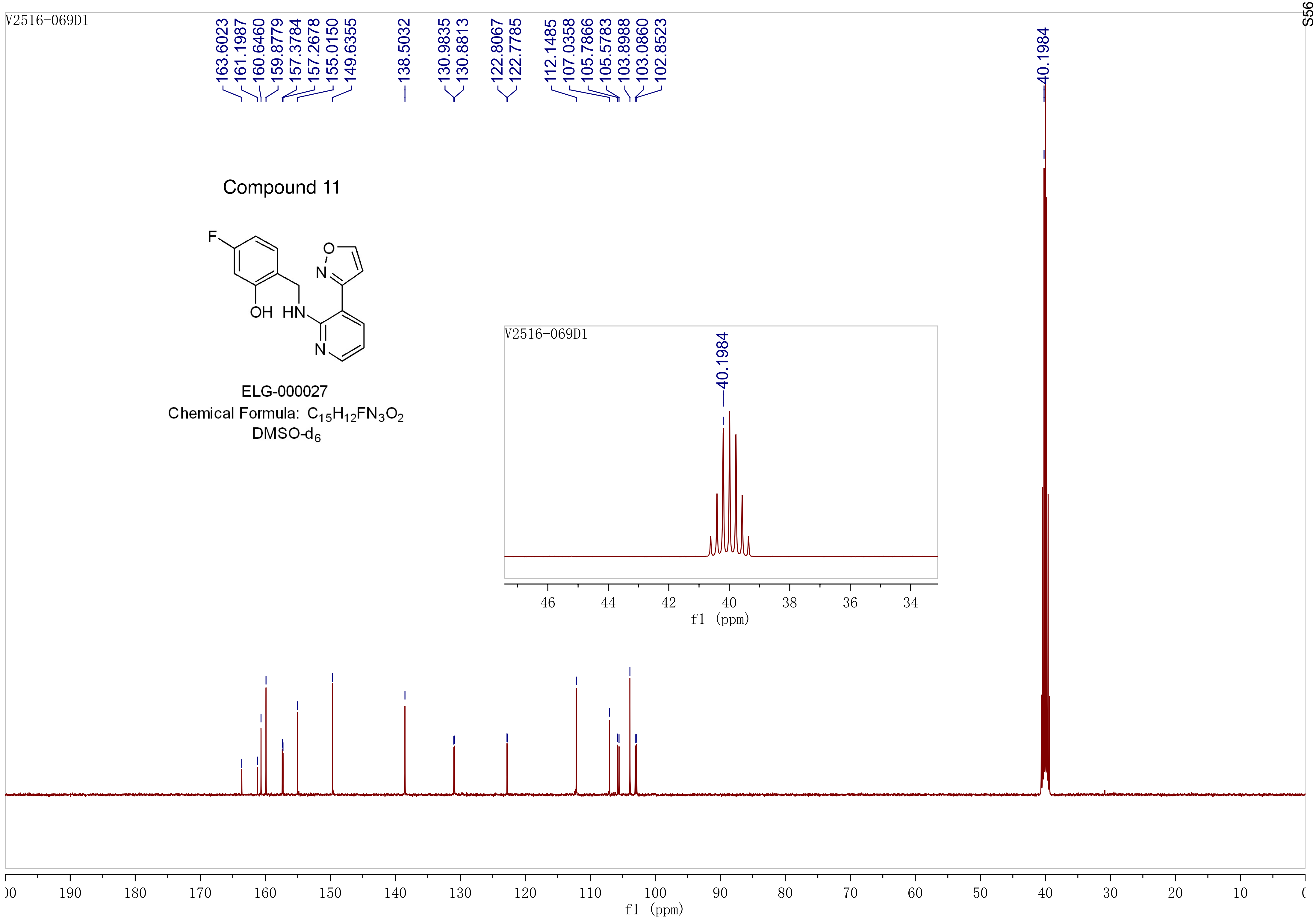
V2516-069D1
163.6023
161.1987
160.6460
159.8779
157.3784
157.2678
155.0150
149.6355
138.5032
130.9835
130.8813
122.8067
122.7785
112.1485
107.0358
105.7866
105.5783
103.8988
103.0860
102.8523
40.1984
F
O
N
OH
HN
N
ELG-000027
Chemical Formula: $C_{15}H_{12}FN_3O_2$
DMSO-d_6
f1 (ppm)

SAMPLE INFORMATION

Sample Name:	V2516-069D1-2	Acquired By:	System
Sample Type:	Standard	Sample Set Name	20200821
Vial:	1:A,4	Acq. Method Set:	VIVA QC_WATERS BEH C18
Injection #:	1	Processing Method	214, Process standrads method
Injection Volume:	0.50 ul	Channel Name:	PDA Ch3 214nm@4.8nm, PDA
Run Time:	15.0 Minutes	Proc. Chnl. Descr.:	PDA Ch3 214nm@4.8nm, PDA

Date Acquired: 8/21/2020 9:47:00 AM CST

Date Processed: 8/21/2020 10:09:47 AM CST, 8/21/2020 10:09:59 AM CST, 8/21/2020 10:10:14 AM

Compound 11

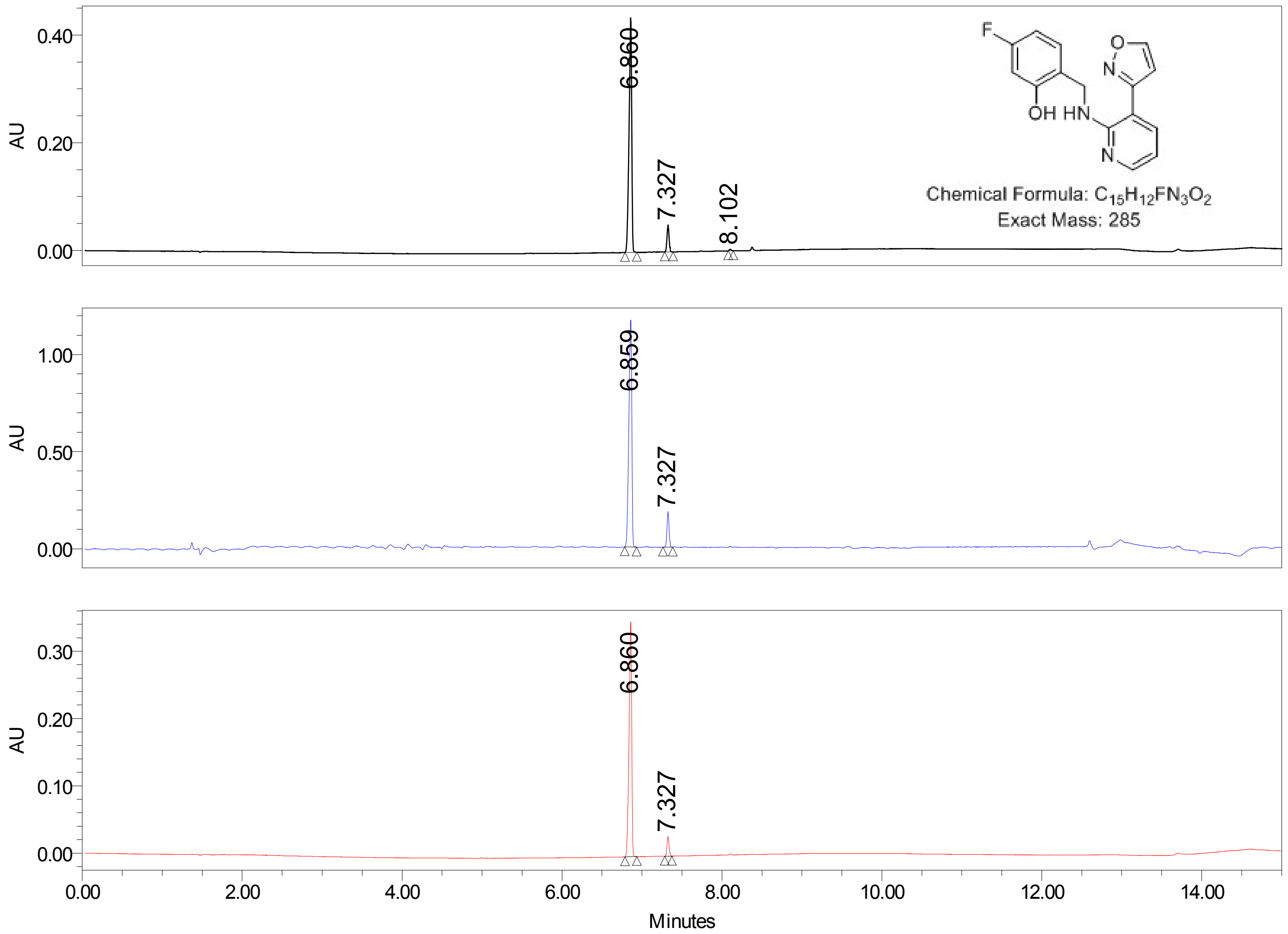

Channel: PDA Ch1 254nm@4.8nm; Processed Channel: PDA Ch1 254nm@4.8nm; Result Id: 8305; Processing Method: Process standrads method

Channel: PDA Ch3 214nm@4.8nm; Processed Channel: PDA Ch3 214nm@4.8nm; Result Id: 8307; Processing Method: 214

Channel: PDA Ch2 280nm@4.8nm; Processed Channel: PDA Ch2 280nm@4.8nm; Result Id: 8306; Processing Method: Process standrads method

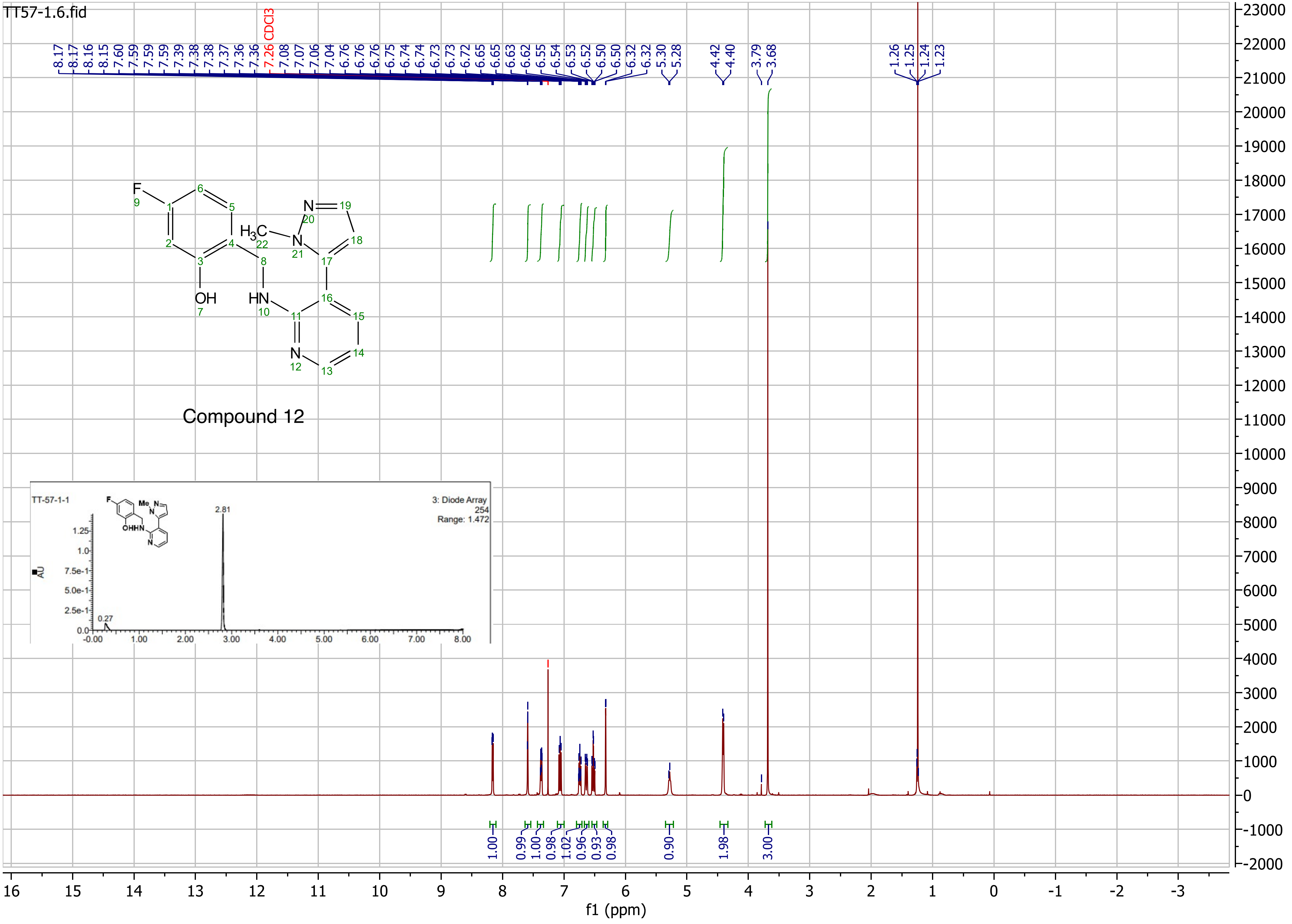
TT57-1.6.fid
8.17
8.17
8.16
8.15
7.60
7.59
7.59
7.59
7.39
7.38
7.38
7.37
7.36
7.36
7.26 CDCl3
7.08
7.07
7.06
7.04
6.76
6.76
6.76
6.75
6.74
6.74
6.73
6.73
6.72
6.65
6.65
6.63
6.62
6.55
6.54
6.53
6.52
6.50
6.50
6.32
6.32
5.30
5.28
4.42
4.40
3.79
3.68
1.26
1.25
1.24
1.23
Compound 12
TT-57-1-1
3: Diode Array
254
Range: 1.472
2.81
0.27
AU
1.00
0.99
1.00
0.98
1.02
0.96
0.93
0.98
0.90
1.98
3.00
f1 (ppm)

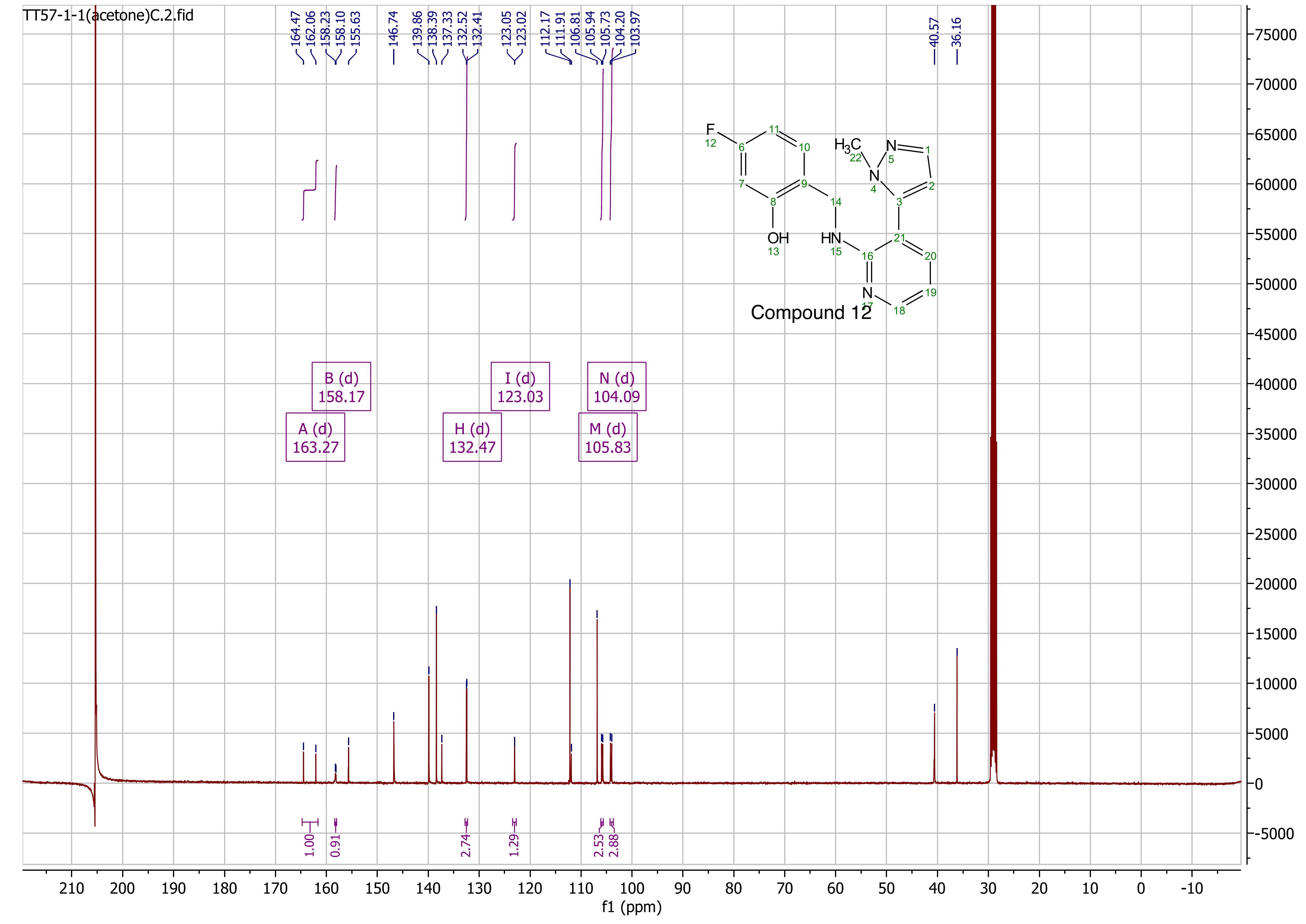
TT57-1-1(acetone)C.2.fid
164.47
162.06
158.23
158.10
155.63
146.74
139.86
138.39
137.33
132.52
132.41
123.05
123.02
112.17
111.91
106.81
105.94
105.73
104.20
103.97
40.57
36.16
F
OH
HN
H_3C
N
Compound 12
B (d) 158.17
A (d) 163.27
H (d) 132.47
I (d) 123.03
N (d) 104.09
M (d) 105.83
1.00
0.91
2.74
1.29
2.53
2.88
f1 (ppm)

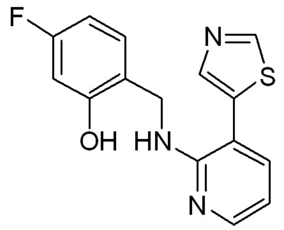

Compound 13

V2398-026
DMSO

Parameter	Value
1 Title	V2398-26-04
2 Origin	Bruker BioSpin GmbH
3 Solvent	DMSO
4 Temperature	298.0
5 Number of Scans	16
6 Spectrometer Frequency	400.13

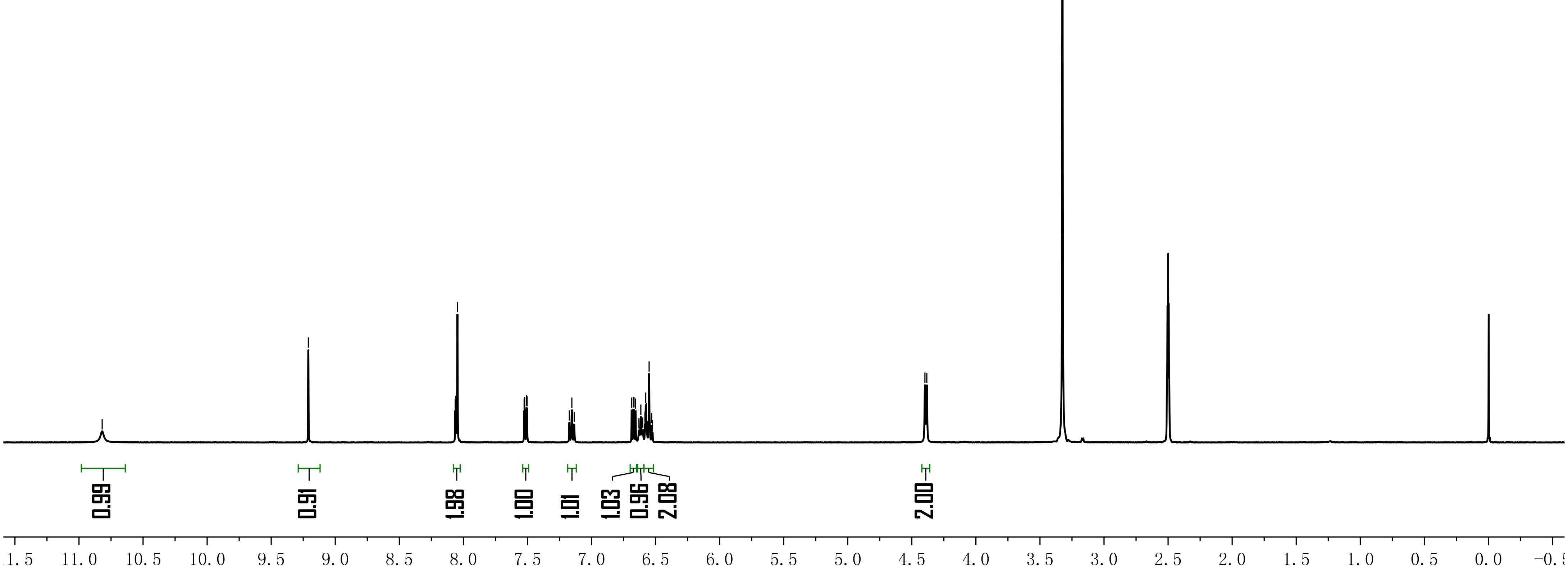

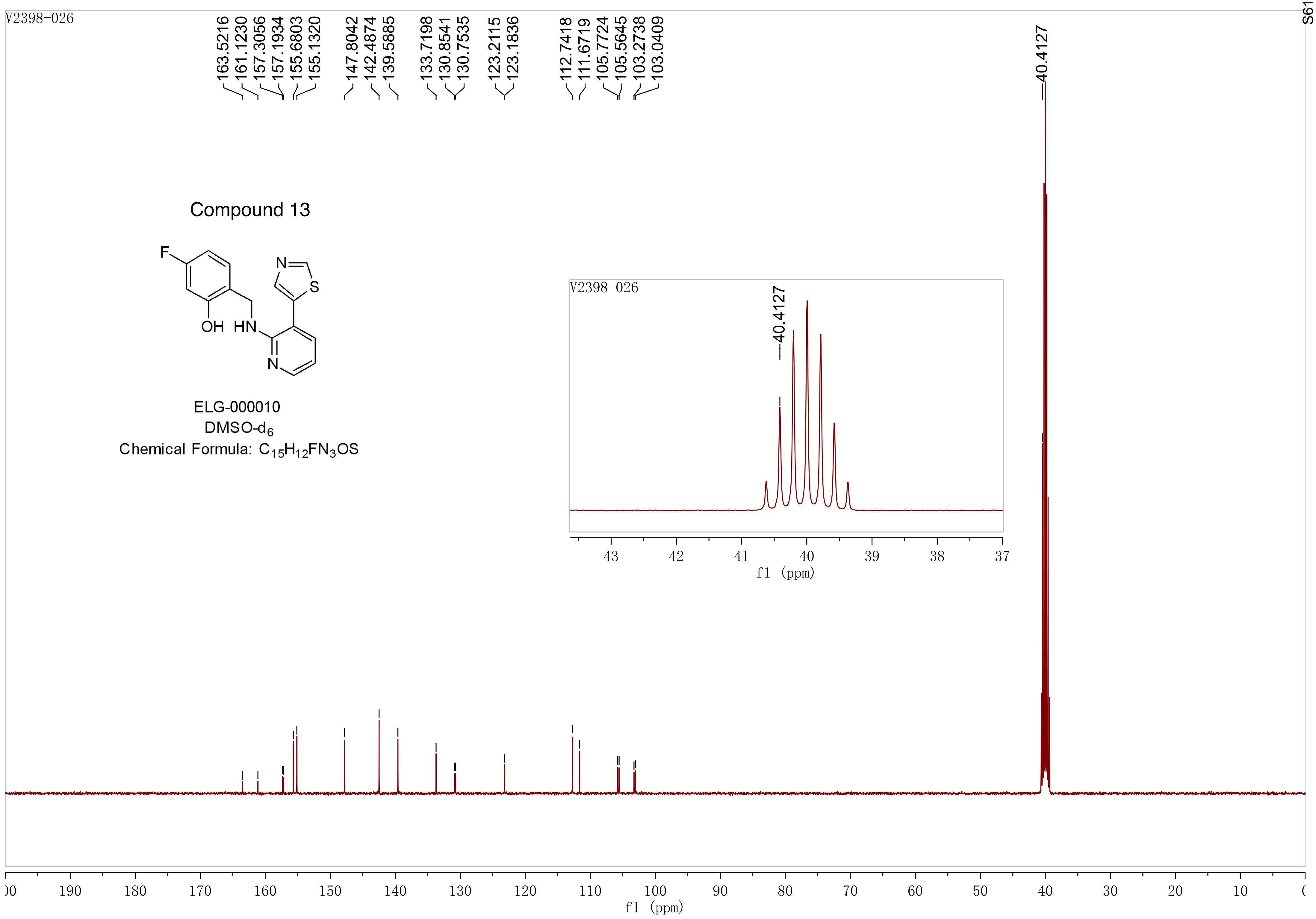

V2398-026
163.5216
161.1230
157.3056
157.1934
155.6803
155.1320
147.8042
142.4874
139.5885
133.7198
130.8541
130.7535
123.2115
123.1836
112.7418
111.6719
105.7724
105.5645
103.2738
103.0409
40.4127
Compound 13
F
N
S
OH
HN
N
ELG-000010
DMSO-d6
Chemical Formula: C15H12FN3OS
V2398-026
40.4127
43
42
41
40
39
38
37
f1 (ppm)
190
180
170
160
150
140
130
120
110
100
90
80
70
60
50
40
30
20
10
f1 (ppm)

Injection Summary Report

SAMPLE INFORMATION

Sample Name:	V2398-026	Acquired By:	System
Sample Type:	Standard	Sample Set Name	20200624
Vial:	2:A,1	Acq. Method Set:	VIVA QC_WATERS BEH C18
Injection #:	1	Processing Method	214, Process standrads method
Injection Volume:	0.50 ul	Channel Name:	PDA Ch3 214nm@4.8nm, PDA
Run Time:	15.0 Minutes	Proc. Chnl. Descr.:	PDA Ch3 214nm@4.8nm, PDA

Date Acquired: 6/24/2020 9:47:09 AM CST
Date Processed: 6/24/2020 10:25:20 AM CST, 6/24/2020 10:25:33 AM CST, 6/24/2020 10:25:46 AM

Compound 13

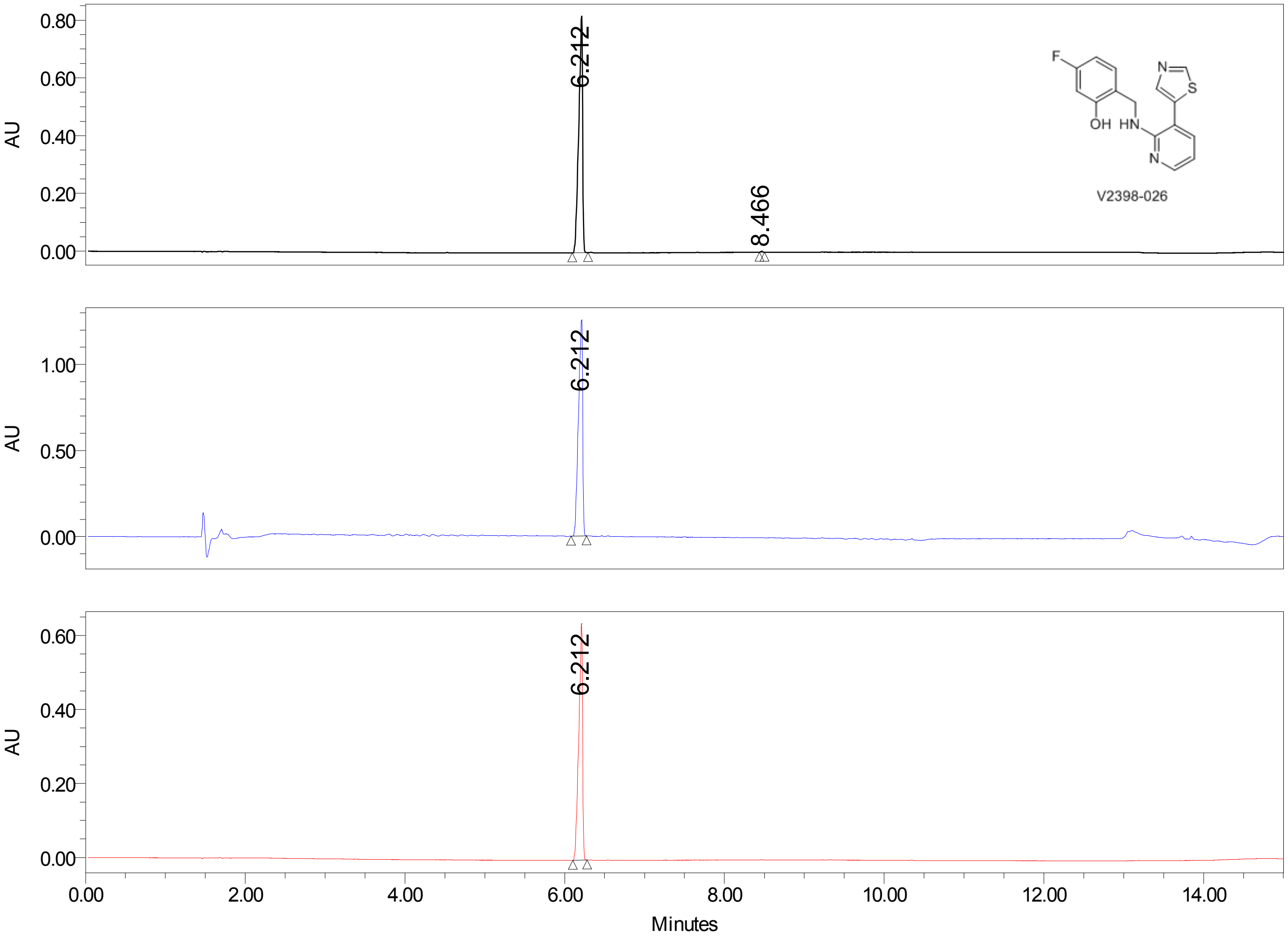

Channel: PDA Ch1 254nm@4.8nm; Processed Channel: PDA Ch1 254nm@4.8nm; Result Id: 16005; Processing Method: Process standrads method

Channel: PDA Ch3 214nm@4.8nm; Processed Channel: PDA Ch3 214nm@4.8nm; Result Id: 16007; Processing Method: 214

Channel: PDA Ch2 280nm@4.8nm; Processed Channel: PDA Ch2 280nm@4.8nm; Result Id: 16006; Processing Method: Process standrads method

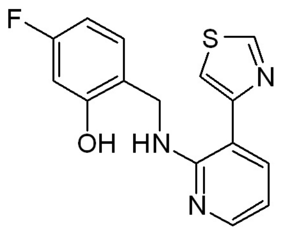

Compound 14

V2398-022
DMSO

	Parameter	Value
1	Title	V2398-022
2	Origin	Bruker BioSpin GmbH
3	Solvent	DMSO
4	Temperature	298.0
5	Number of Scans	16
6	Spectrometer Frequency	400.13

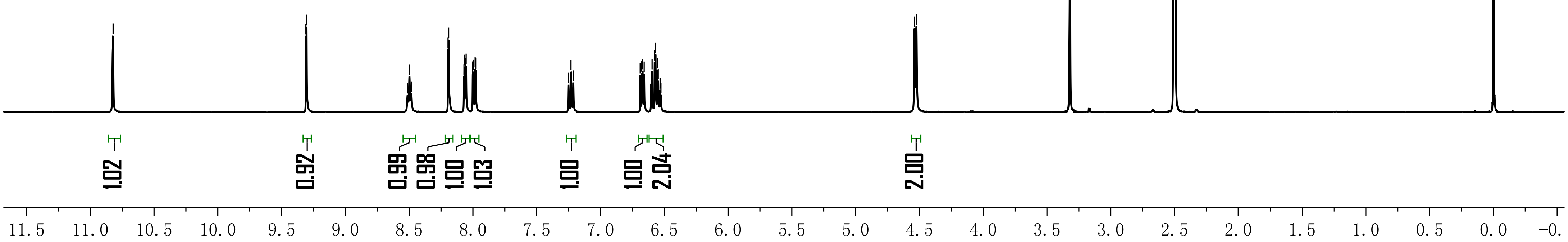

Compound 14

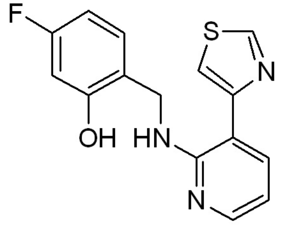

ELG-000011
DMSO-d_6
Chemical Formula: $C_{15}H_{12}FN_3OS$

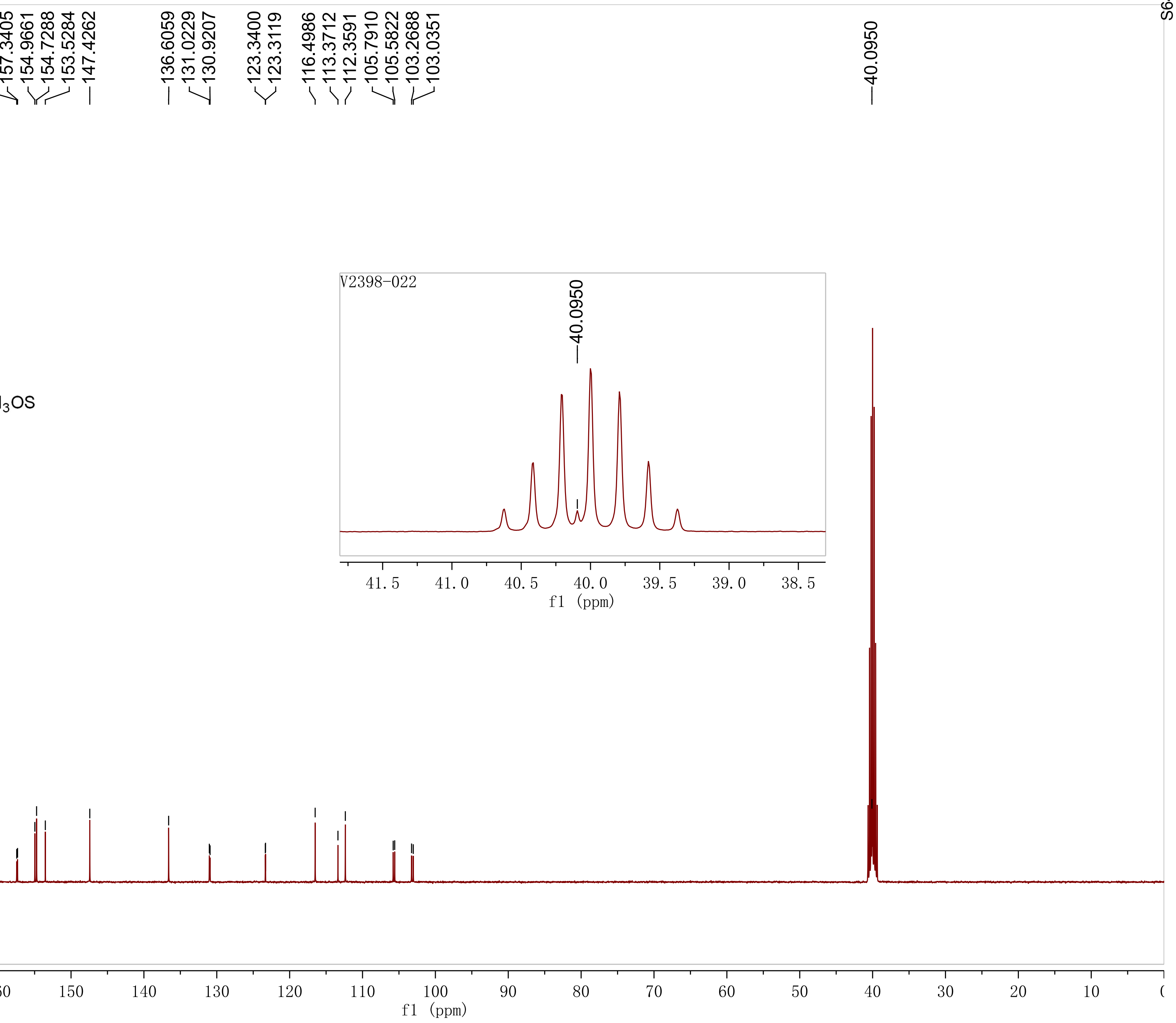

SAMPLE INFORMATION

Sample Name:	V2398-022	Acquired By:	System
Sample Type:	Standard	Sample Set Name	20200618
Vial:	2:F,6	Acq. Method Set:	VIVA QC_WATERS BEH C18
Injection #:	1	Processing Method	214, Process standrads method
Injection Volume:	0.50 ul	Channel Name:	PDA Ch3 214nm@4.8nm, PDA
Run Time:	15.0 Minutes	Proc. Chnl. Descr.:	PDA Ch3 214nm@4.8nm, PDA

Date Acquired: 6/18/2020 1:49:22 PM CST
Date Processed: 6/18/2020 2:22:25 PM CST, 6/18/2020 2:22:37 PM CST, 6/18/2020 2:22:56 PM CST

Compound 14

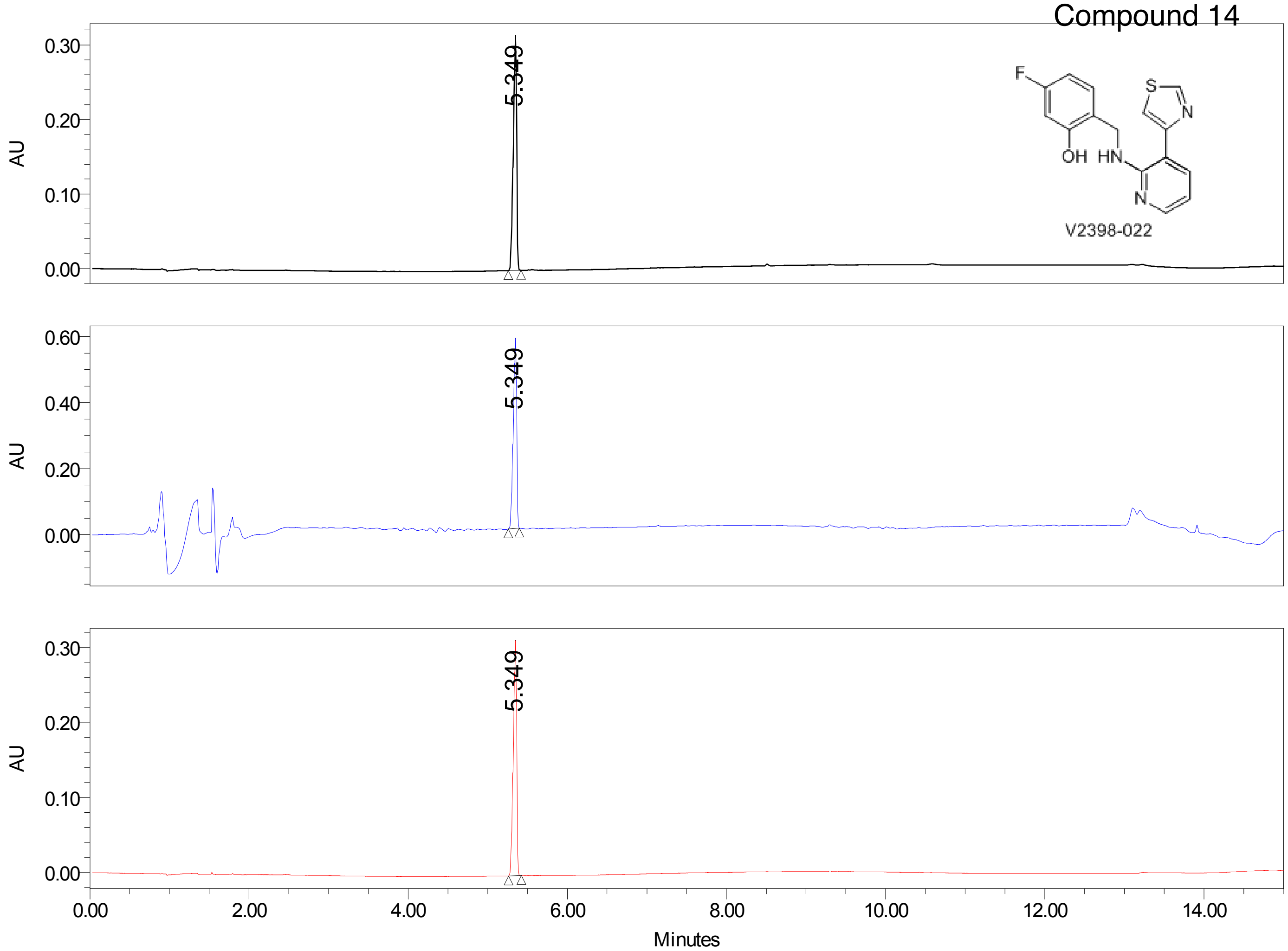

Channel: PDA Ch1 254nm@4.8nm; Processed Channel: PDA Ch1 254nm@4.8nm; Result Id: 14946; Processing Method: Process standrads method

Channel: PDA Ch3 214nm@4.8nm; Processed Channel: PDA Ch3 214nm@4.8nm; Result Id: 14948; Processing Method: 214

Channel: PDA Ch2 280nm@4.8nm; Processed Channel: PDA Ch2 280nm@4.8nm; Result Id: 14947; Processing Method: Process standrads method

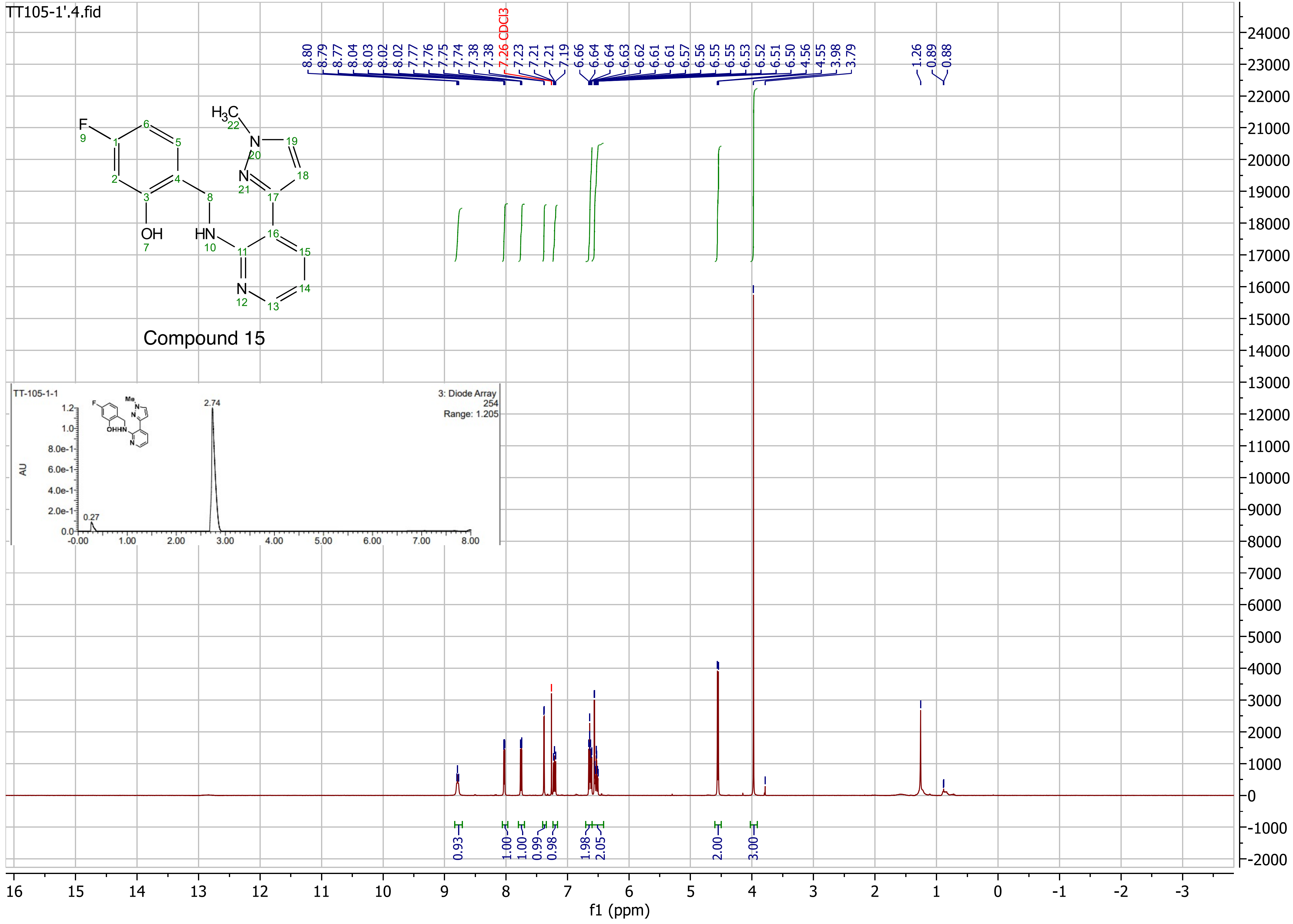

TT105-1'.4.fid
8.80
8.79
8.77
8.04
8.03
8.02
8.02
7.77
7.76
7.75
7.74
7.38
7.38
7.26 CDCl3
7.23
7.21
7.21
7.19
6.66
6.64
6.64
6.63
6.62
6.61
6.61
6.57
6.56
6.55
6.55
6.53
6.52
6.51
6.50
4.56
4.55
3.98
3.79
1.26
0.89
0.88
Compound 15
TT-105-1-1
3: Diode Array
254
Range: 1.205
2.74
0.27
AU
0.93
1.00
1.00
0.99
0.98
1.98
2.05
2.00
3.00
f1 (ppm)

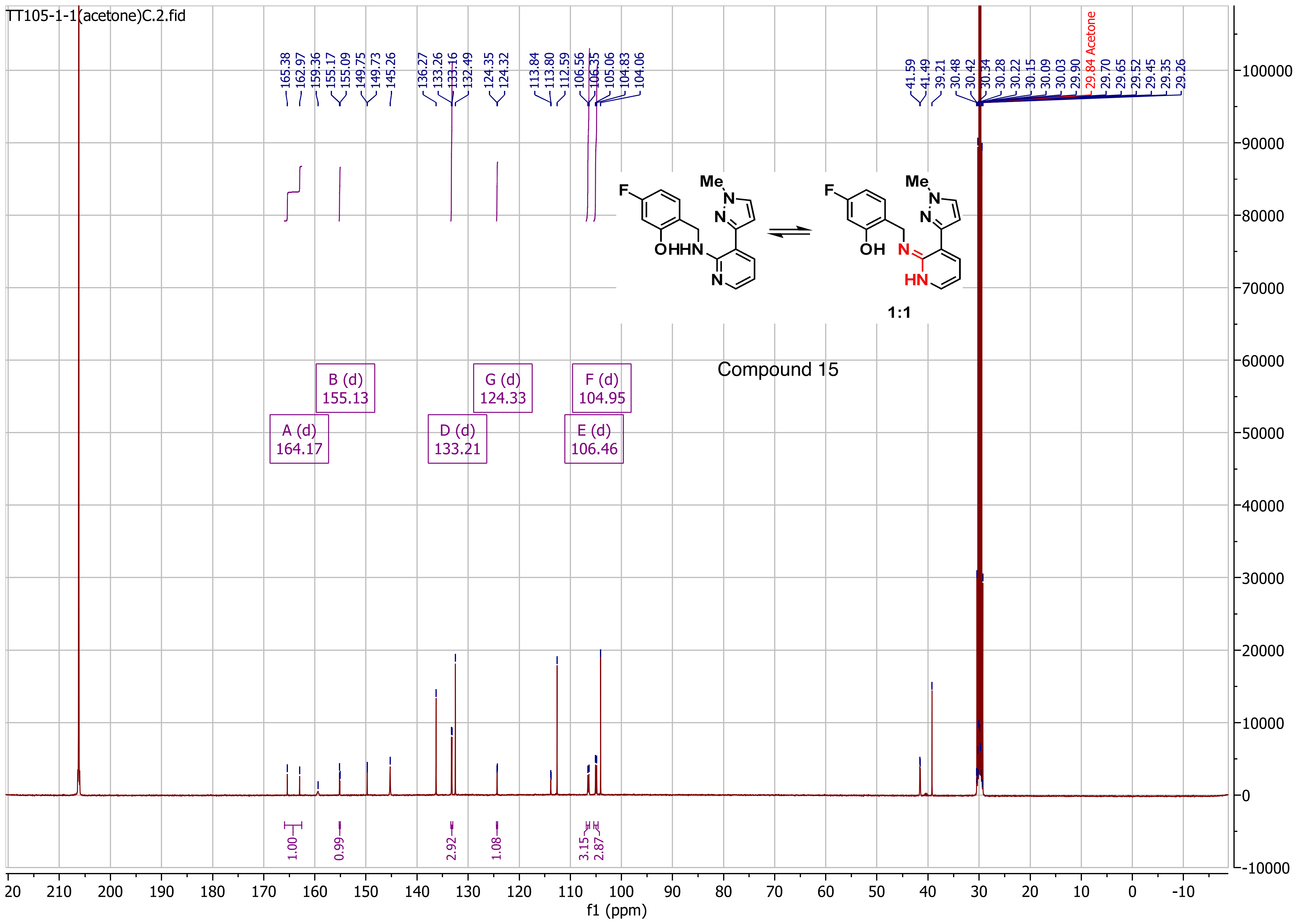
TT105-1-1(acetone)C.2.fid
165.38
162.97
159.36
155.17
155.09
149.75
149.73
145.26
136.27
133.26
133.16
132.49
124.35
124.32
113.84
113.80
112.59
106.56
106.35
105.06
104.83
104.06
41.59
41.49
39.21
30.48
30.42
30.34
30.28
30.22
30.15
30.09
30.03
29.90
29.84 Acetone
29.70
29.65
29.52
29.45
29.35
29.26
A (d) 164.17
B (d) 155.13
D (d) 133.21
G (d) 124.33
E (d) 106.46
F (d) 104.95
1.00
0.99
2.92
1.08
3.15
2.87
1:1
Compound 15
f1 (ppm)

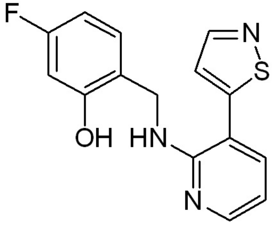

Compound 16

V2398-028
DMSO

Parameter	Value
1 Title	V2398-028
2 Origin	Bruker BioSpin GmbH
3 Solvent	DMSO
4 Temperature	298.0
5 Number of Scans	16
6 Spectrometer Frequency	400.13

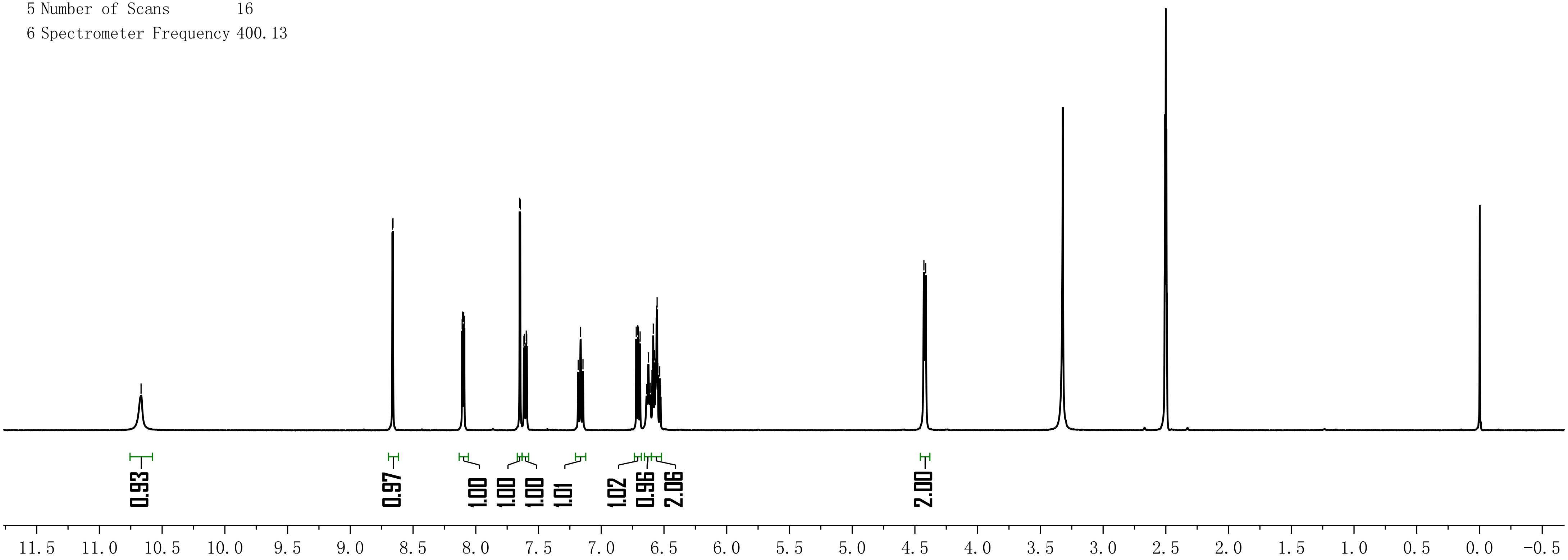

Injection Summary Report

SAMPLE INFORMATION

Sample Name:	V2398-028	Acquired By:	System
Sample Type:	Standard	Sample Set Name	20200623
Vial:	1:F,5	Acq. Method Set:	VIVA QC_WATERS BEH C18
Injection #:	1	Processing Methoc	Process standrads method
Injection Volume:	0.50 ul	Channel Name:	PDA Ch3 214nm@4.8nm, PDA
Run Time:	15.0 Minutes	Proc. Chnl. Descr.:	PDA Ch3 214nm@4.8nm, PDA

Date Acquired: 6/23/2020 3:37:36 PM CST
Date Processed: 6/23/2020 3:56:32 PM CST, 6/23/2020 3:57:03 PM CST, 6/23/2020 3:57:16 PM CST

Compound 16

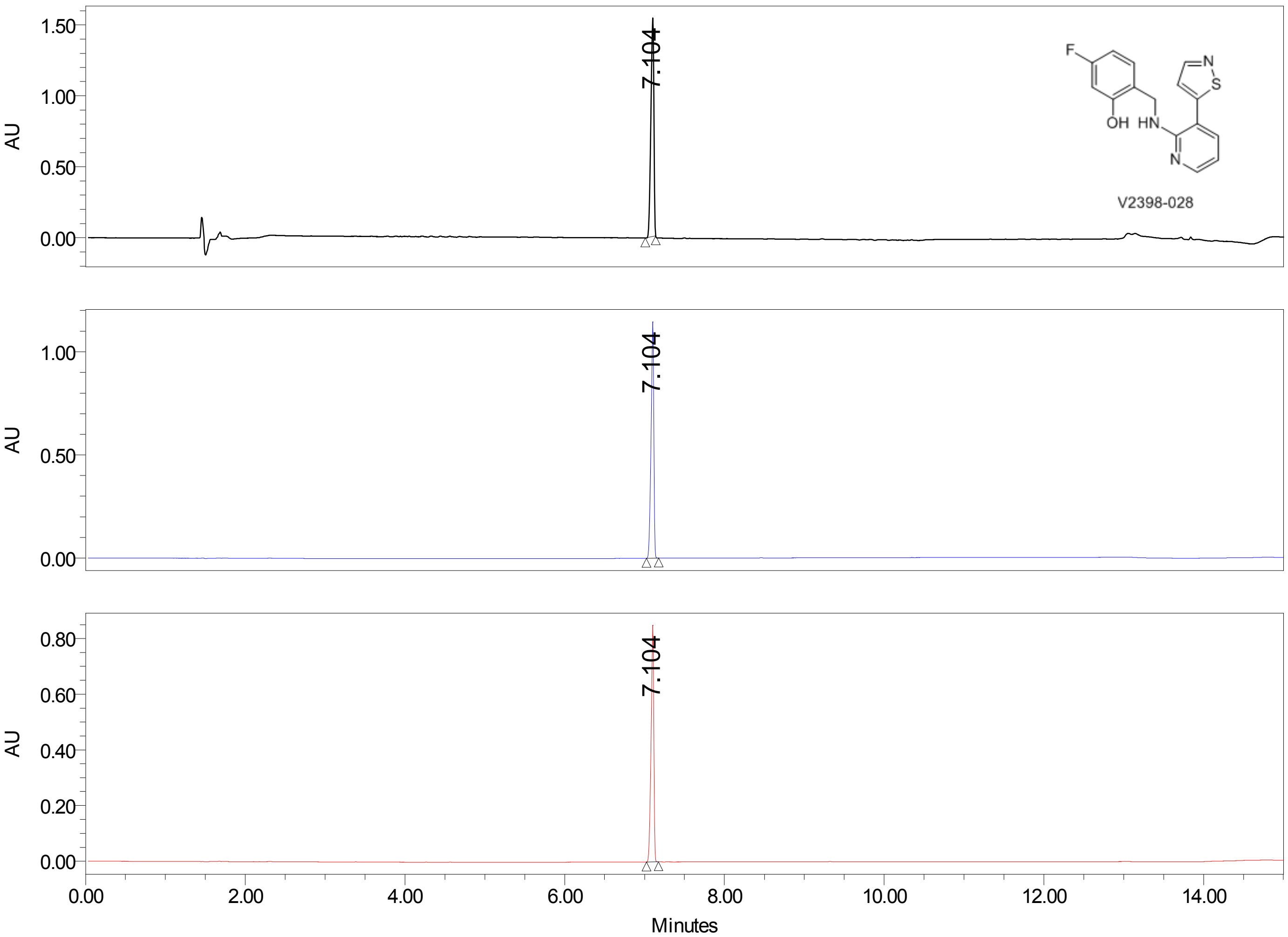

Channel: PDA Ch3 214nm@4.8nm; Processed Channel: PDA Ch3 214nm@4.8nm; Result Id: 15847; Processing Method: Process standrads method

Channel: PDA Ch1 254nm@4.8nm; Processed Channel: PDA Ch1 254nm@4.8nm; Result Id: 15849; Processing Method: Process standrads method

Channel: PDA Ch2 280nm@4.8nm; Processed Channel: PDA Ch2 280nm@4.8nm; Result Id: 15848; Processing Method: Process standrads method

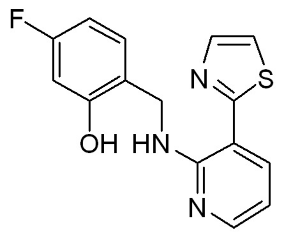

Compound 17

V2398-021
DMSO

Parameter	Value
1 Title	V2398-021
2 Origin	Bruker BioSpin GmbH
3 Solvent	DMSO
4 Temperature	298.0
5 Number of Scans	16
6 Spectrometer Frequency	400.13

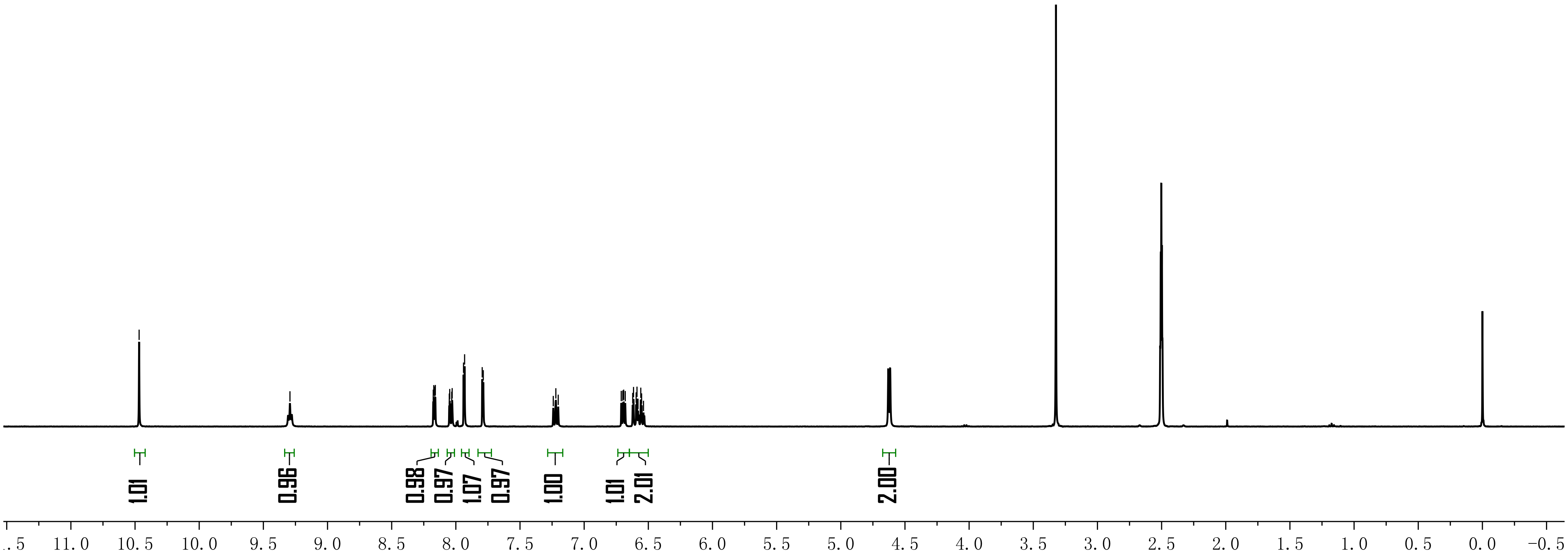

Compound 17

ELG-000013
Chemical Formula: $C_{15}H_{12}FN_3OS$
DMSO-d_6

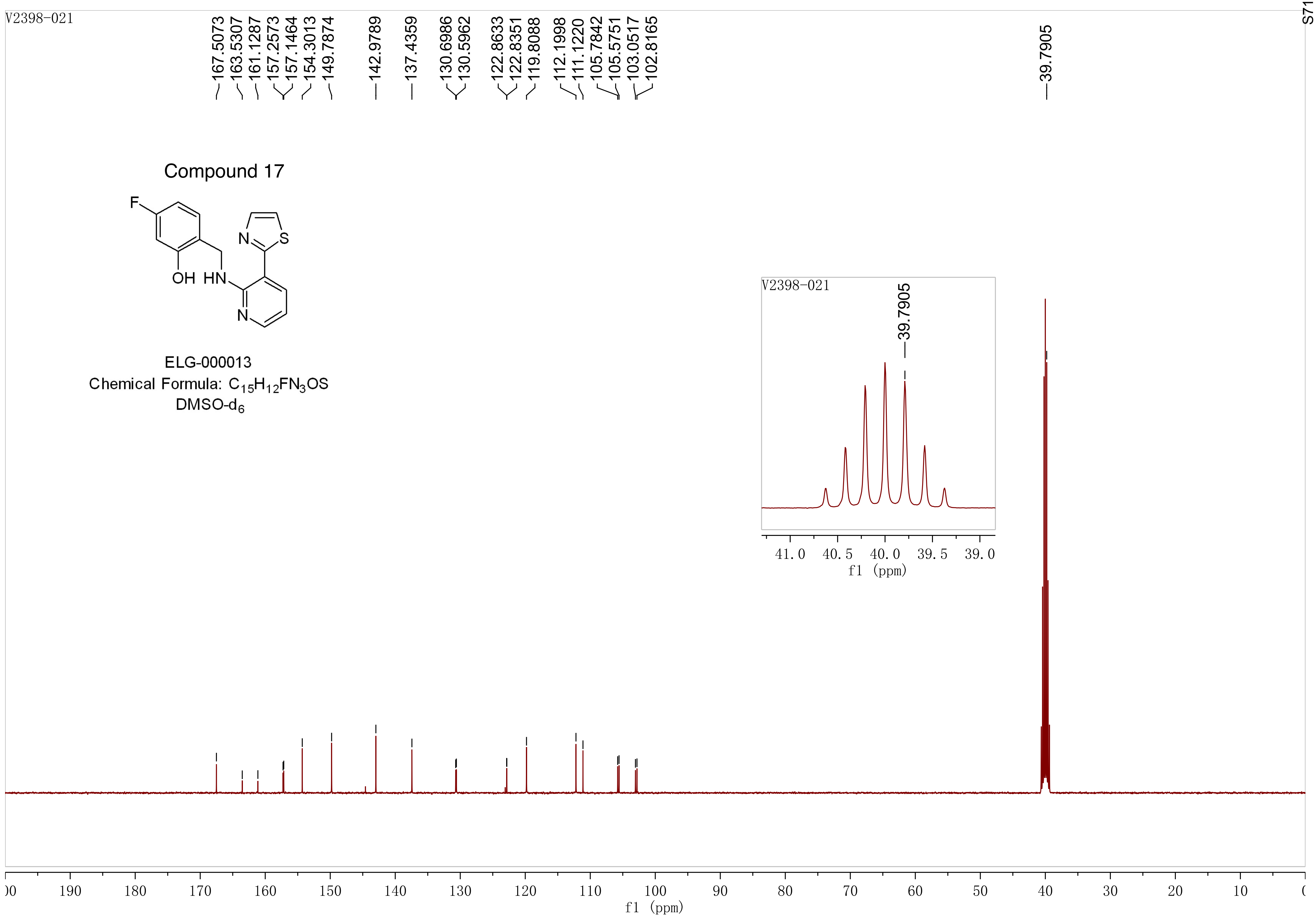

Injection Summary Report

SAMPLE INFORMATION

Sample Name:	V2398-021	Acquired By:	System
Sample Type:	Standard	Sample Set Name	20200618
Vial:	2:F,8	Acq. Method Set:	VIVA QC_WATERS BEH C18
Injection #:	1	Processing Method	214, Process standrads method
Injection Volume:	0.50 ul	Channel Name:	PDA Ch3 214nm@4.8nm, PDA
Run Time:	15.0 Minutes	Proc. Chnl. Descr.:	PDA Ch3 214nm@4.8nm, PDA

Date Acquired: 6/18/2020 2:39:07 PM CST
Date Processed: 6/18/2020 3:09:42 PM CST, 6/18/2020 3:09:52 PM CST, 6/18/2020 3:10:06 PM CST

Compound 17

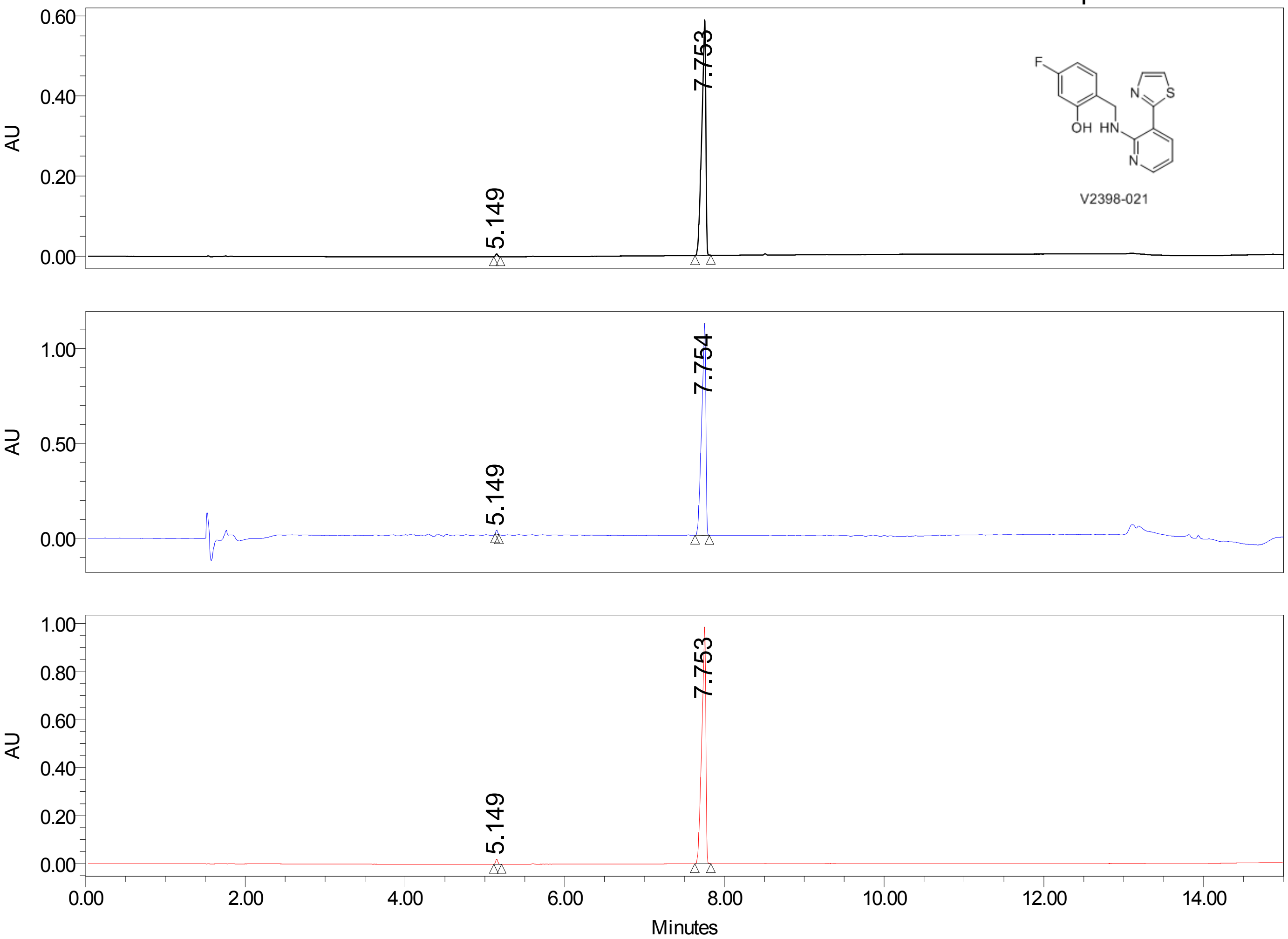

Channel: PDA Ch1 254nm@4.8nm; Processed Channel: PDA Ch1 254nm@4.8nm; Result Id: 14969; Processing Method: Process standrads method
Channel: PDA Ch3 214nm@4.8nm; Processed Channel: PDA Ch3 214nm@4.8nm; Result Id: 14978; Processing Method: 214
Channel: PDA Ch2 280nm@4.8nm; Processed Channel: PDA Ch2 280nm@4.8nm; Result Id: 14970; Processing Method: Process standrads method

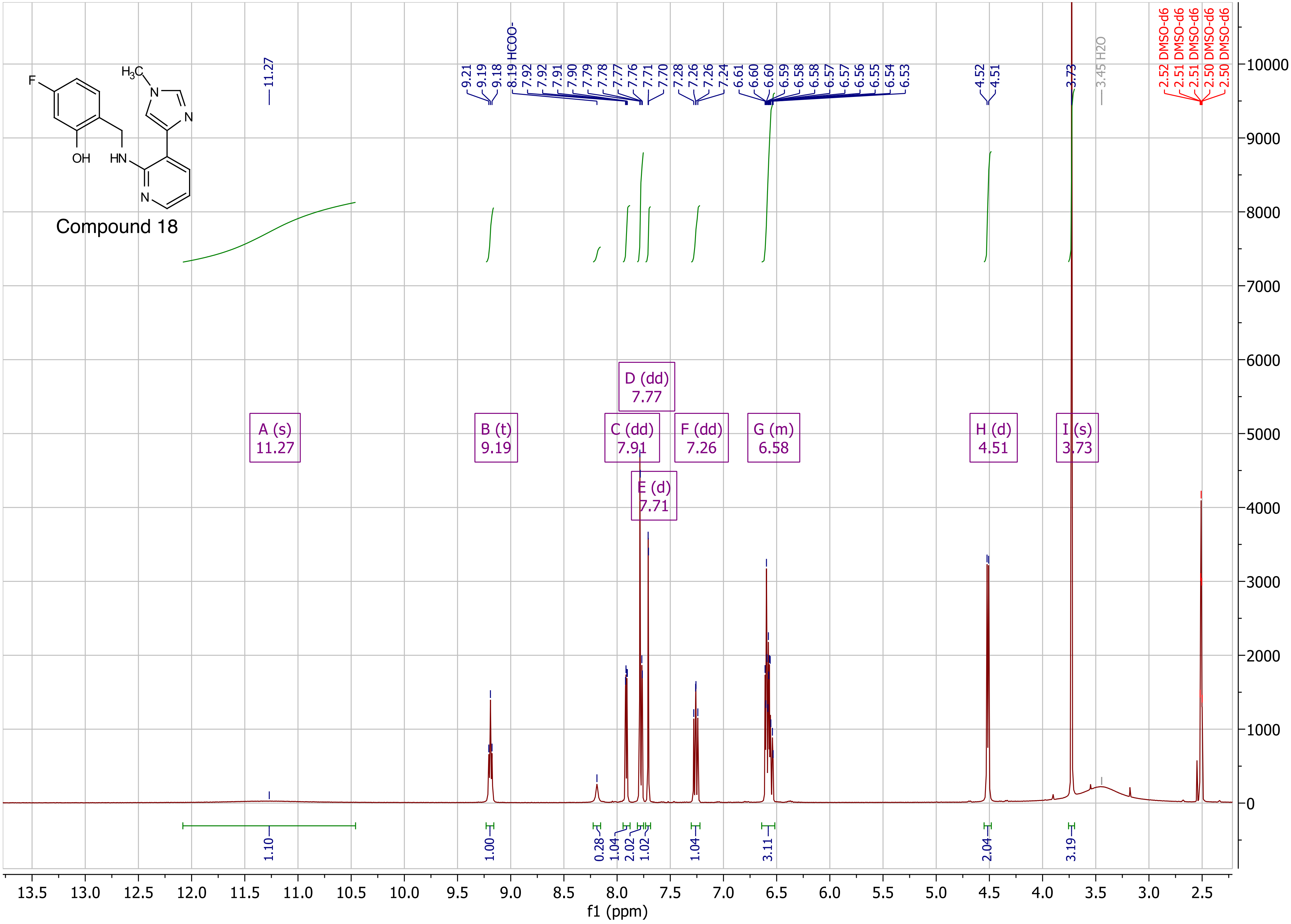
Compound 18
F
OH
HN
N
H3C
N
N
11.27
9.21
9.19
9.18
8.19 HCOO-
7.92
7.92
7.91
7.90
7.79
7.78
7.77
7.76
7.71
7.70
7.28
7.26
7.26
7.24
6.61
6.60
6.60
6.59
6.58
6.58
6.57
6.57
6.56
6.55
6.54
6.53
4.52
4.51
3.73
3.45 H2O
2.52 DMSO-d6
2.51 DMSO-d6
2.51 DMSO-d6
2.50 DMSO-d6
2.50 DMSO-d6
A (s) 11.27
B (t) 9.19
C (dd) 7.91
D (dd) 7.77
E (d) 7.71
F (dd) 7.26
G (m) 6.58
H (d) 4.51
I (s) 3.73
1.10
1.00
0.28
1.04
2.02
1.02
1.04
3.11
2.04
3.19
f1 (ppm)

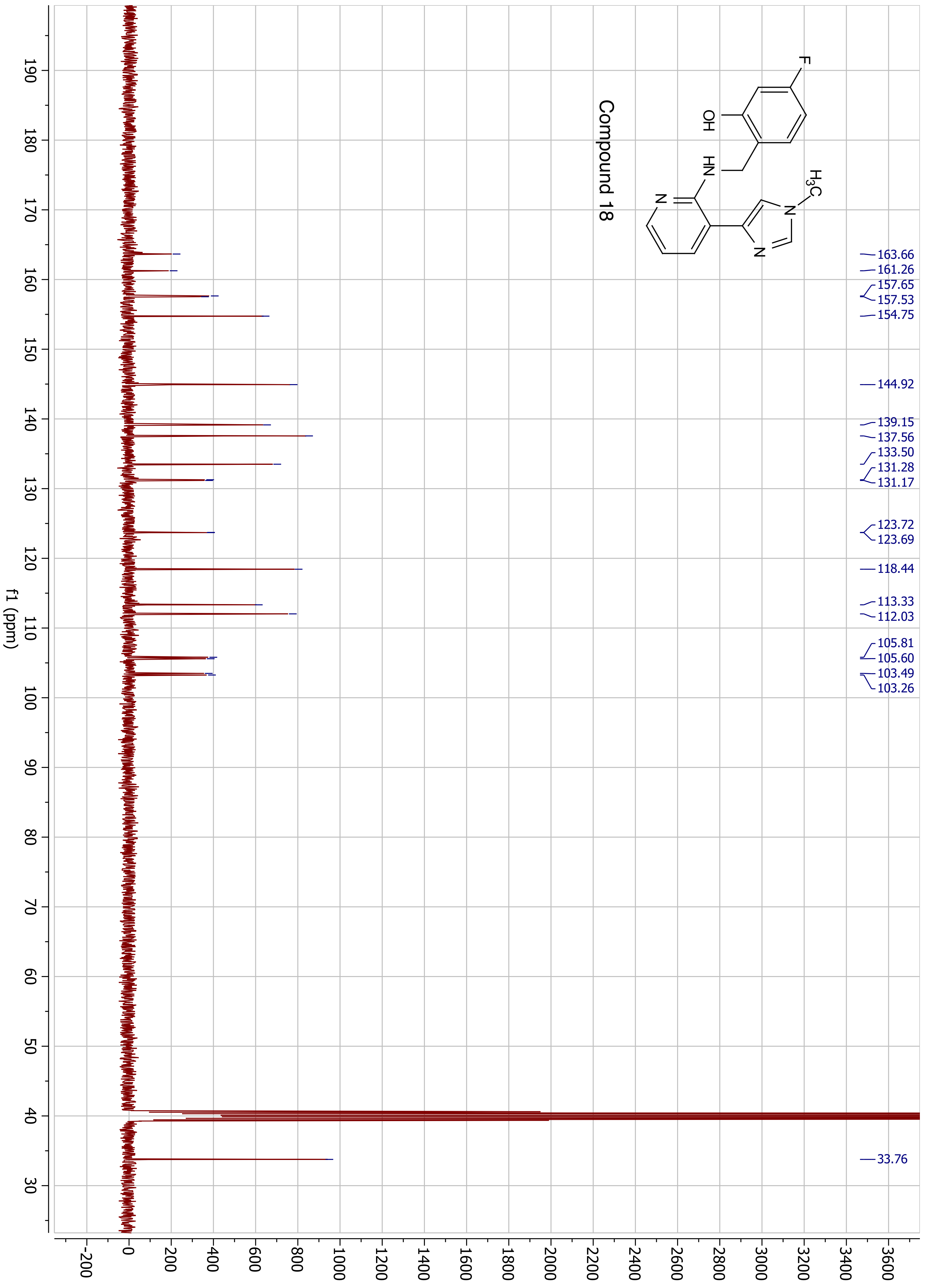
Compound 18
OH
HN
N
F
H_3C
N
N
163.66
161.26
157.65
157.53
154.75
144.92
139.15
137.56
133.50
131.28
131.17
123.72
123.69
118.44
113.33
112.03
105.81
105.60
103.49
103.26
33.76
f1 (ppm)

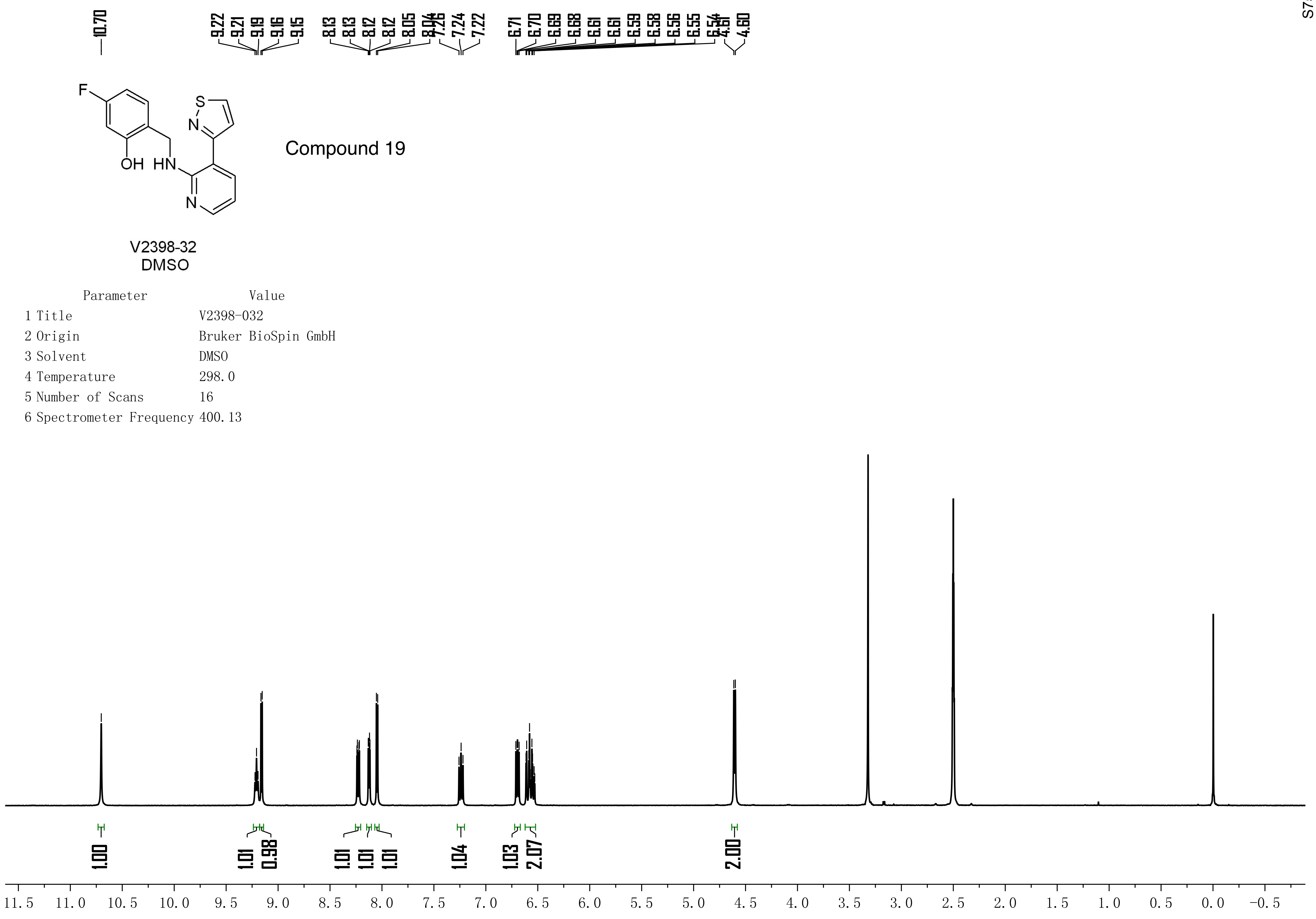

10.70
9.22
9.21
9.19
9.16
9.15
8.13
8.13
8.12
8.12
8.05
8.04
7.26
7.24
7.22
6.71
6.70
6.69
6.68
6.61
6.61
6.59
6.58
6.56
6.55
6.54
4.61
4.60
F
S
N
OH
HN
N
Compound 19
V2398-32
DMSO
Parameter Value
1 Title V2398-032
2 Origin Bruker BioSpin GmbH
3 Solvent DMSO
4 Temperature 298.0
5 Number of Scans 16
6 Spectrometer Frequency 400.13
1.00
1.01
0.98
1.01
1.01
1.01
1.04
1.03
2.07
2.00
11.5
11.0
10.5
10.0
9.5
9.0
8.5
8.0
7.5
7.0
6.5
6.0
5.5
5.0
4.5
4.0
3.5
3.0
2.5
2.0
1.5
1.0
0.5
0.0
-0.5

Compound 19

ELG-000017

DMSO-d_6

Chemical Formula: $C_{15}H_{12}FN_3OS$

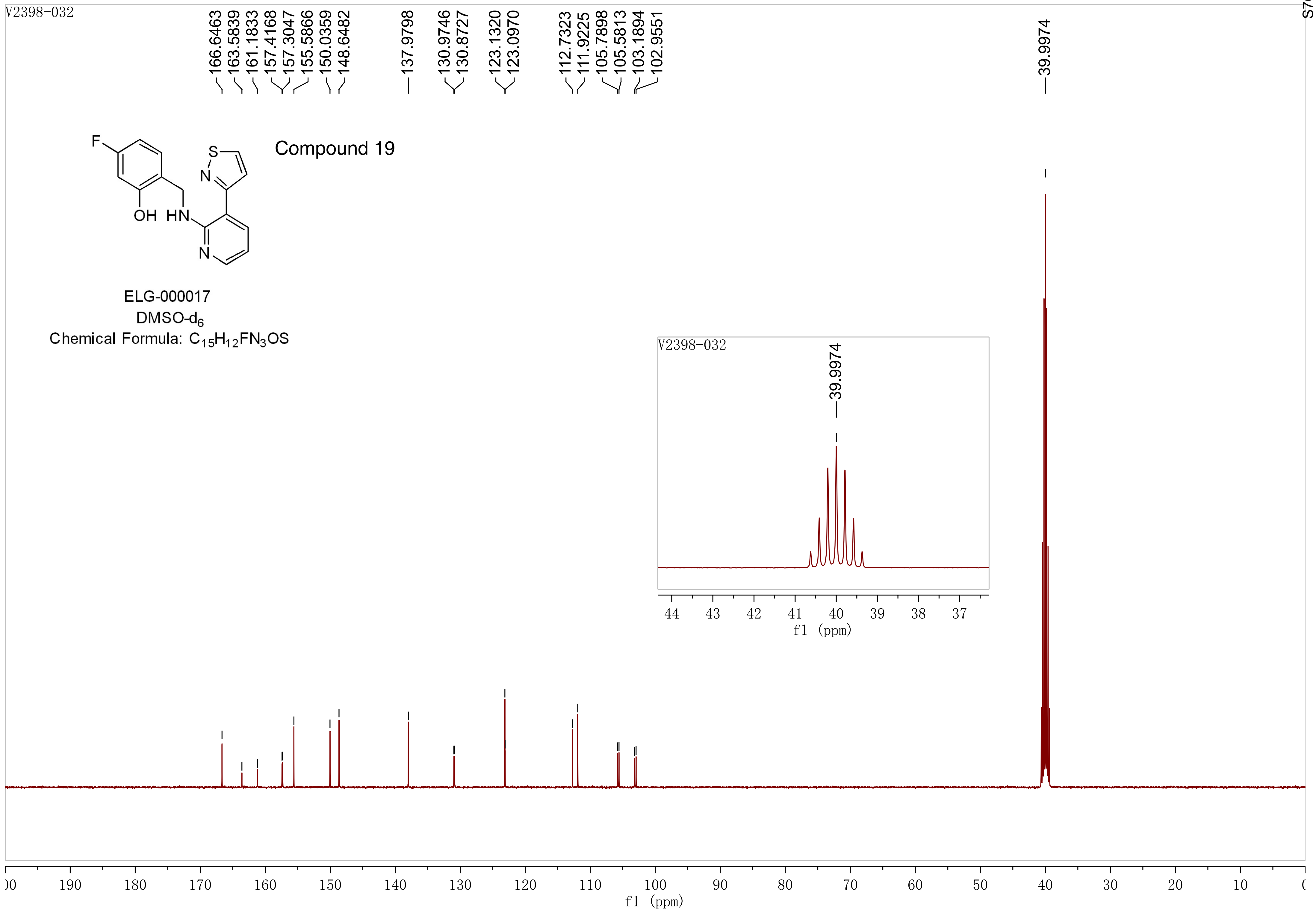

Injection Summary Report

SAMPLE INFORMATION

Sample Name:	V2398-032	Acquired By:	Systen
Sample Type:	Standard	Sample Set Name	20200
Vial:	1:A,6	Acq. Method Set:	VIVA (
Injection #:	1	Processing Method	214, P
Injection Volume:	0.50 ul	Channel Name:	PDA C
Run Time:	15.0 Minutes	Proc. Chnl. Descr.:	PDA C
Date Acquired:	6/29/2020 9:44:53 AM CST		
Date Processed:	6/29/2020 10:01:20 AM CST, 6/29/2020 10:01:33 AM CST, (		

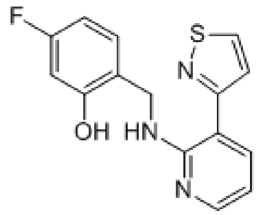

V2398-032

Compound 19

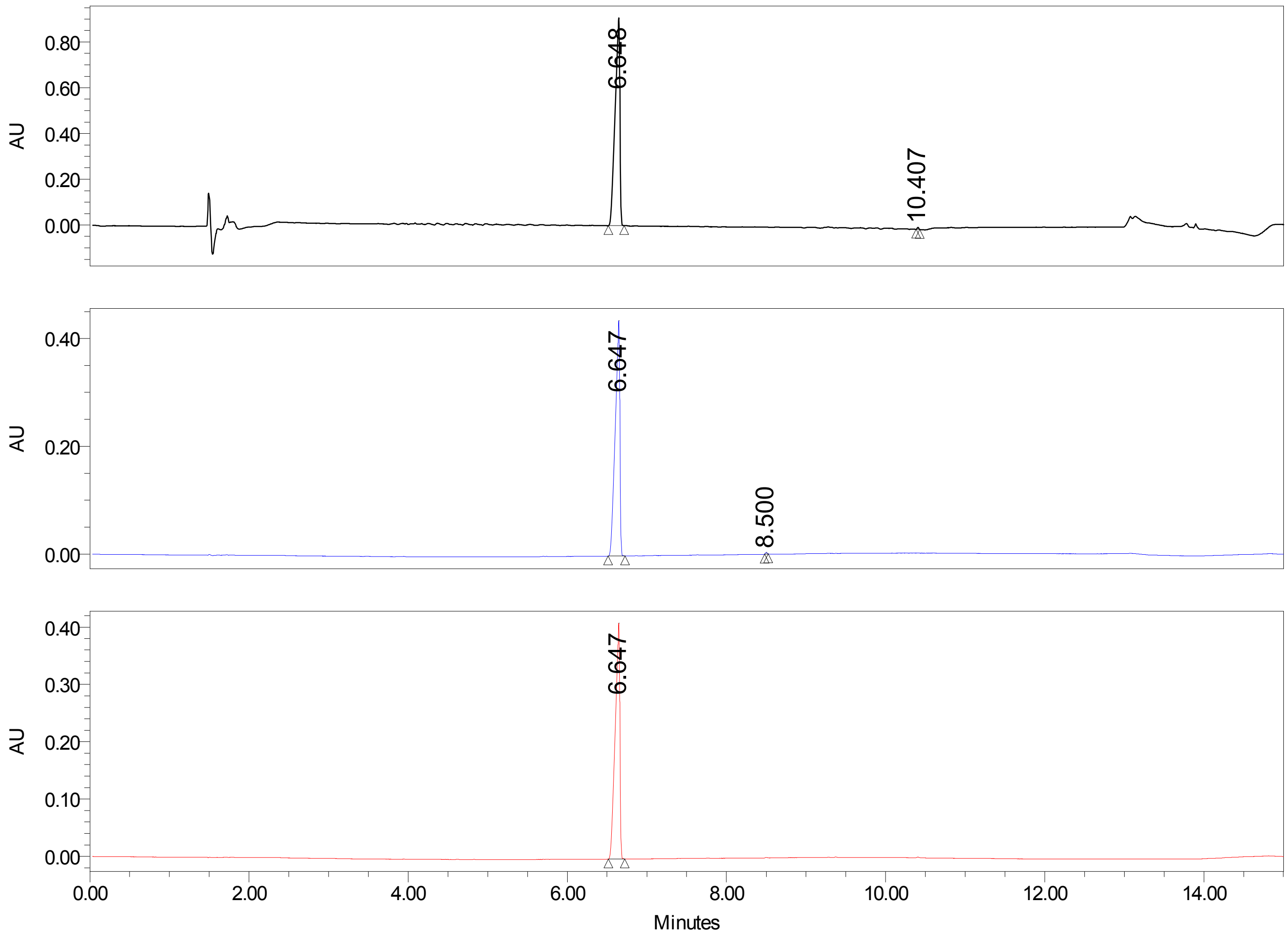

Channel: PDA Ch3 214nm@4.8nm; Processed Channel: PDA Ch3 214nm@4.8nm; Result Id: 16293; Processing Method: 214

Channel: PDA Ch1 254nm@4.8nm; Processed Channel: PDA Ch1 254nm@4.8nm; Result Id: 16295; Processing Method: Process standrads method

Channel: PDA Ch2 280nm@4.8nm; Processed Channel: PDA Ch2 280nm@4.8nm; Result Id: 16294; Processing Method: Process standrads method

Parameter	Value
Origin	Bruker BioSpin GmbH
Solvent	DMSO
Temperature	298.0
Number of Scans	16
Spectrometer Frequency	400.13

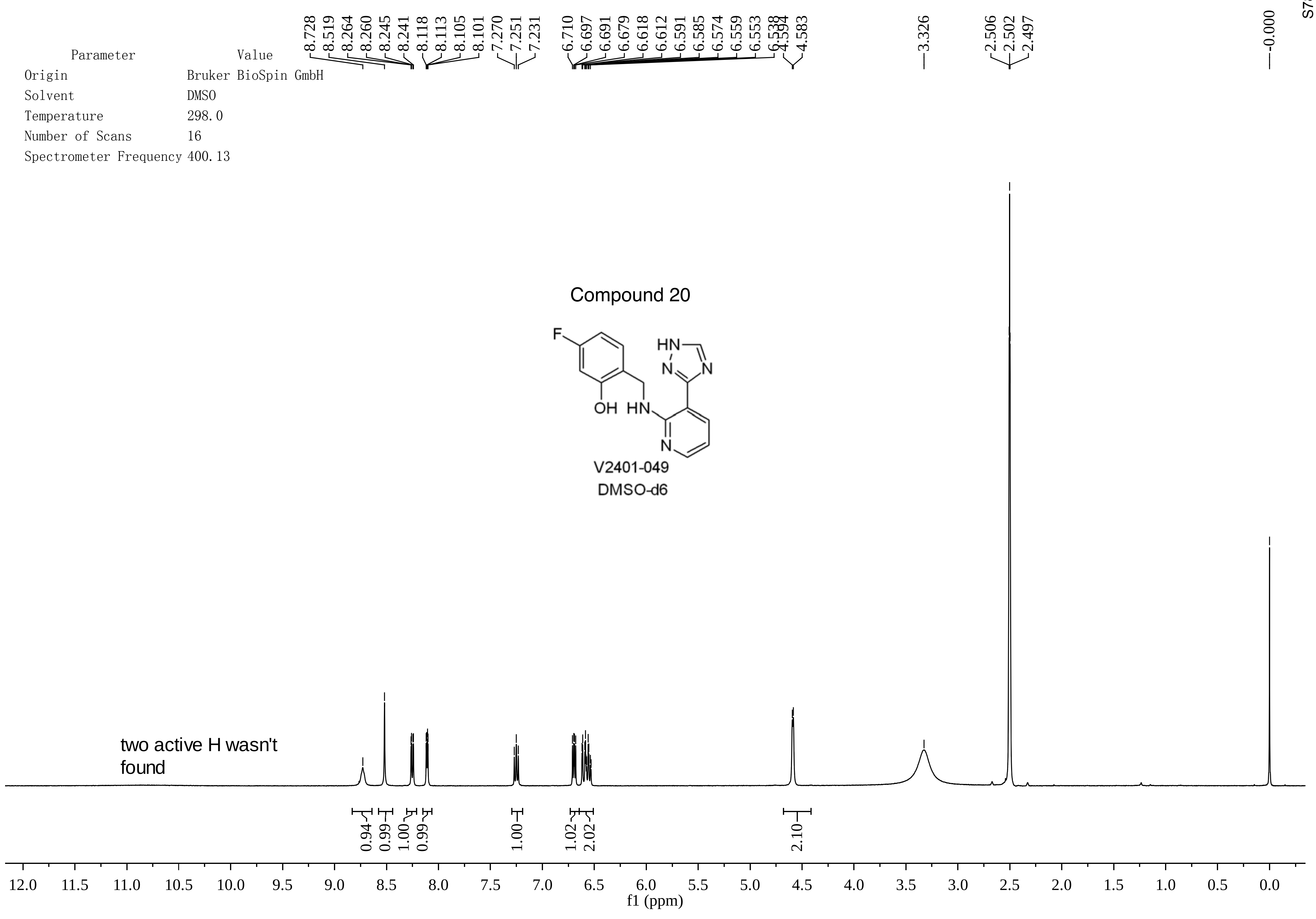

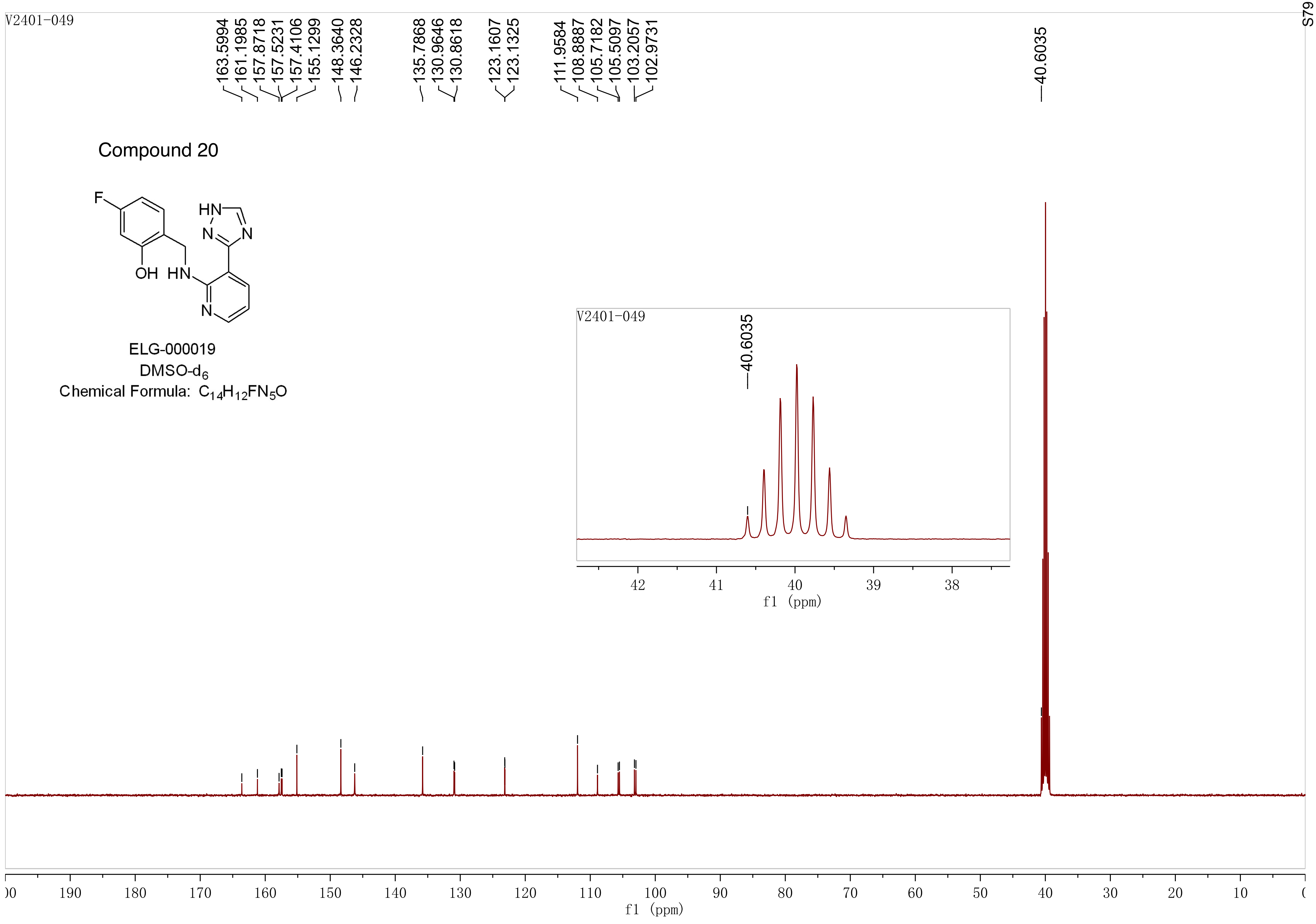
V2401-049
163.5994
161.1985
157.8718
157.5231
157.4106
155.1299
148.3640
146.2328
135.7868
130.9646
130.8618
123.1607
123.1325
111.9584
108.8887
105.7182
105.5097
103.2057
102.9731
40.6035
Compound 20
ELG-000019
DMSO-d6
Chemical Formula: C14H12FN5O
V2401-049
40.6035
42
41
40
39
38
f1 (ppm)
190
180
170
160
150
140
130
120
110
100
90
80
70
60
50
40
30
20
10
f1 (ppm)

Injection Summary Report

SAMPLE INFORMATION

Sample Name:	V2401-049	Acquired By:	System
Sample Type:	Standard	Sample Set Name	20200703
Vial:	1:E,1	Acq. Method Set:	VIVA QC_WATERS BEH C18
Injection #:	1	Processing Method	214, Process standrads method
Injection Volume:	0.50 ul	Channel Name:	PDA Ch3 214nm@4.8nm, PDA
Run Time:	15.0 Minutes	Proc. Chnl. Descr.:	PDA Ch3 214nm@4.8nm, PDA

Date Acquired: 7/3/2020 9:31:54 AM CST
Date Processed: 7/3/2020 9:51:50 AM CST, 7/3/2020 9:52:06 AM CST, 7/3/2020 9:52:43 AM CST

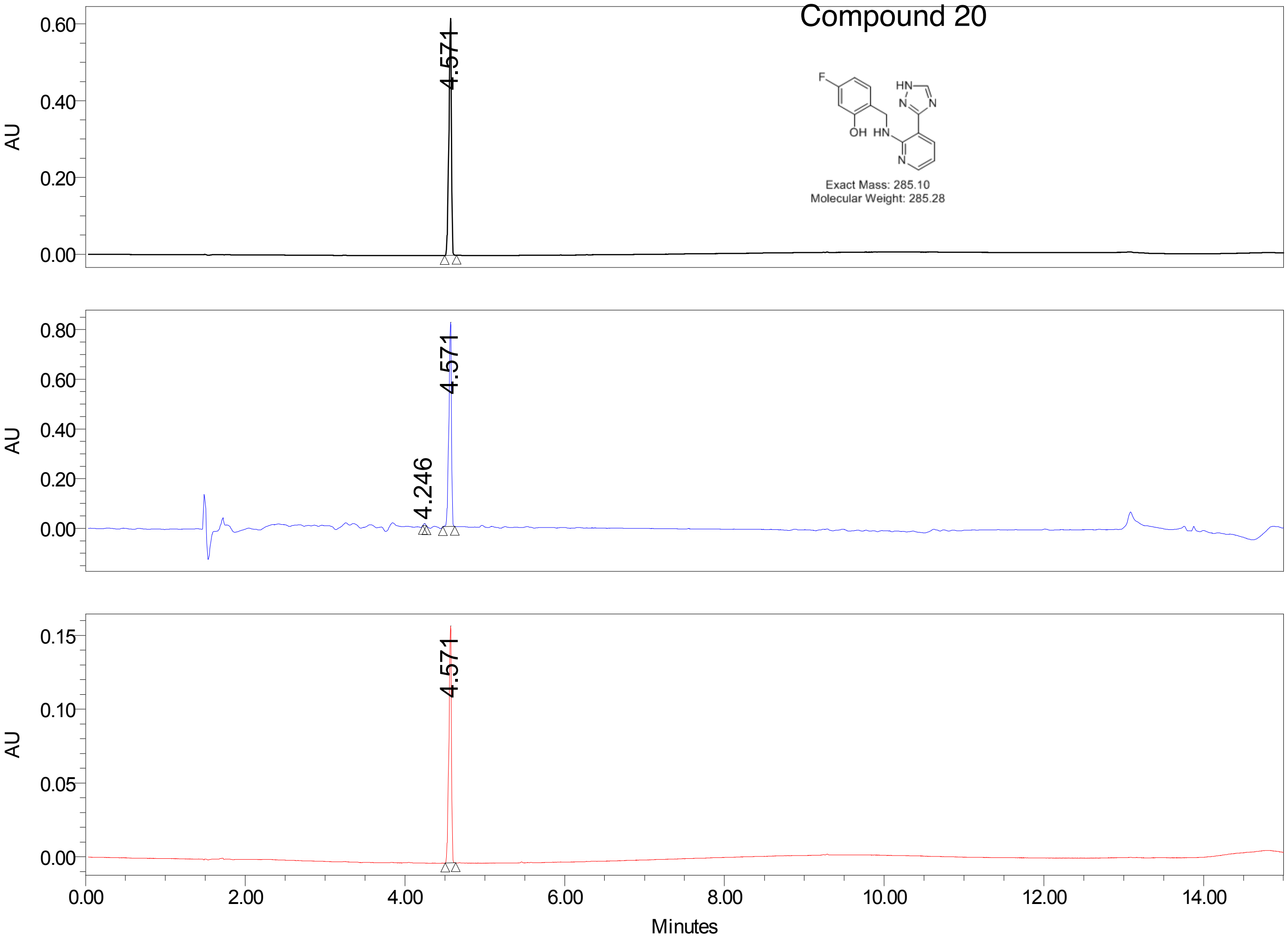

Channel: PDA Ch1 254nm@4.8nm; Processed Channel: PDA Ch1 254nm@4.8nm; Result Id: 16687; Processing Method: Process standrads method
Channel: PDA Ch3 214nm@4.8nm; Processed Channel: PDA Ch3 214nm@4.8nm; Result Id: 16689; Processing Method: 214
Channel: PDA Ch2 280nm@4.8nm; Processed Channel: PDA Ch2 280nm@4.8nm; Result Id: 16688; Processing Method: Process standrads method

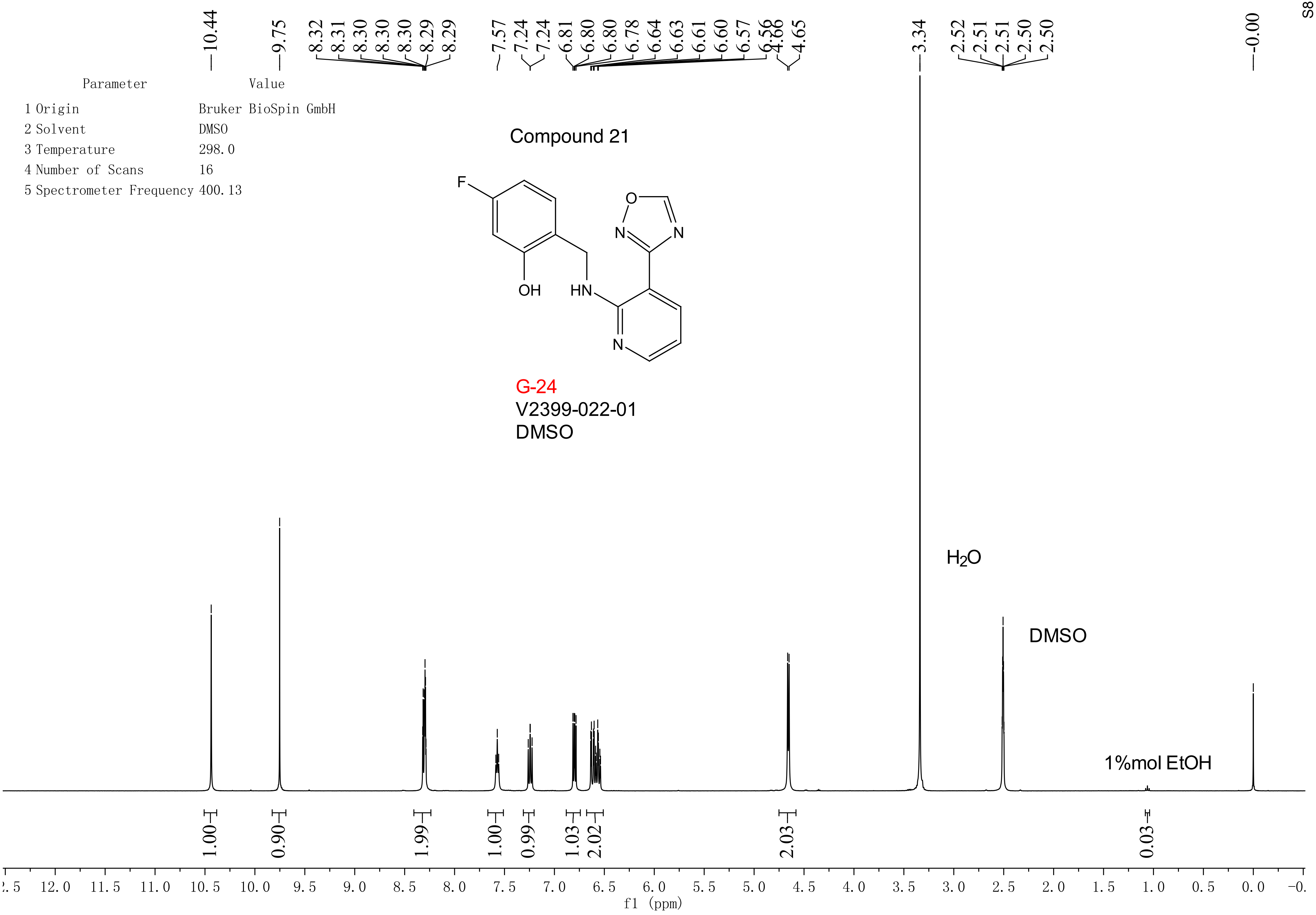
10.44
9.75
8.32
8.31
8.30
8.30
8.30
8.29
8.29
7.57
7.24
7.24
6.81
6.80
6.80
6.78
6.64
6.63
6.61
6.60
6.57
6.56
4.66
4.65
3.34
2.52
2.51
2.51
2.50
2.50
-0.00
Parameter Value
1 Origin Bruker BioSpin GmbH
2 Solvent DMSO
3 Temperature 298.0
4 Number of Scans 16
5 Spectrometer Frequency 400.13
Compound 21
F
OH
HN
N
O
N
N
G-24
V2399-022-01
DMSO
H2O
DMSO
1%mol EtOH
1.00
0.90
1.99
1.00
0.99
1.03
2.02
2.03
0.03
f1 (ppm)

Compound 21

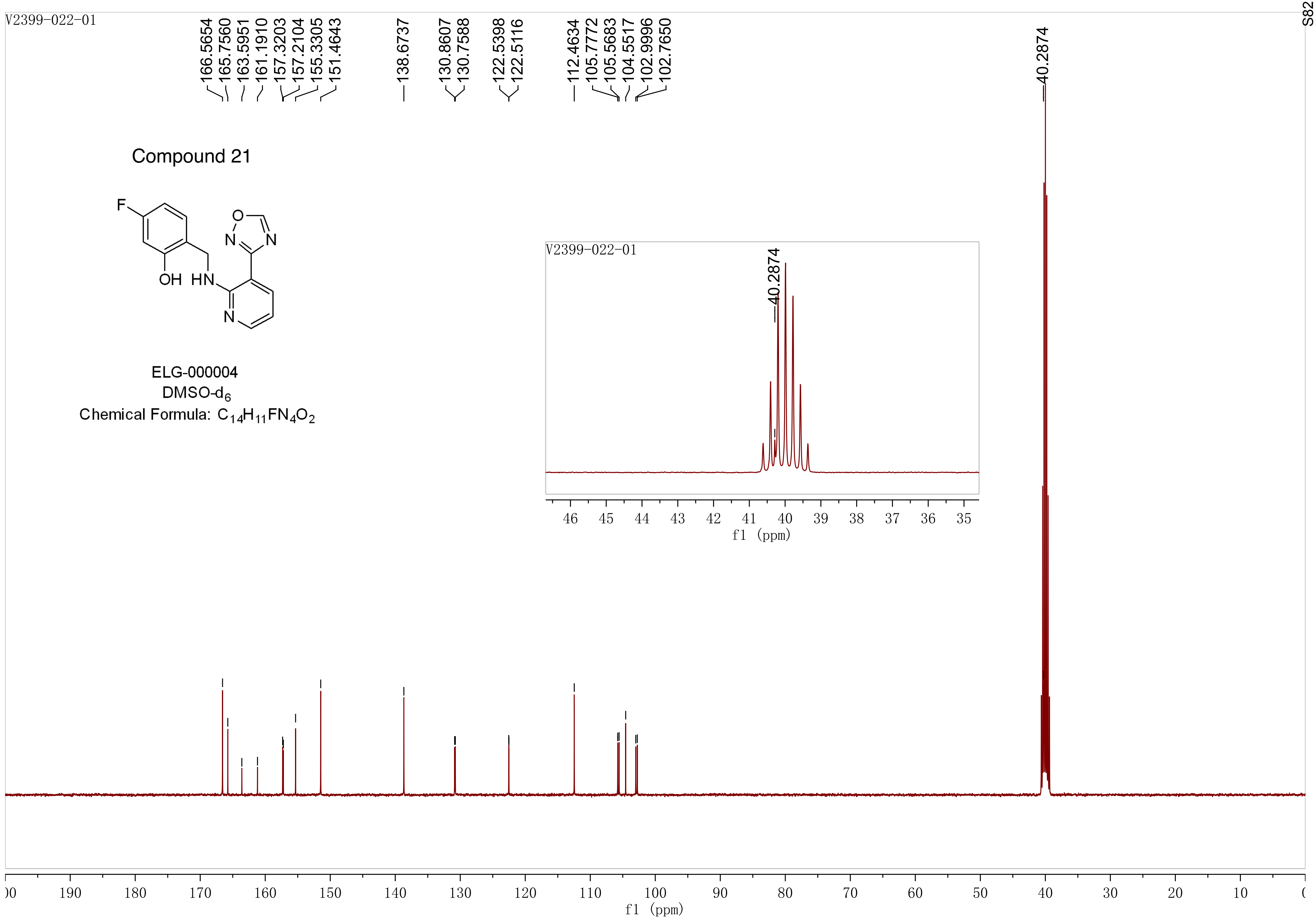

ELG-000004

DMSO-d_6

Chemical Formula: $C_{14}H_{11}FN_4O_2$

SAMPLE INFORMATION

Sample Name:	V2399-022-01	Acquired By:	System
Sample Type:	Standard	Sample Set Name	20200610
Vial:	1:C,7	Acq. Method Set:	VIVA QC_WATERS BEH C18
Injection #:	1	Processing Method	214, Process standrads method
Injection Volume:	0.50 ul	Channel Name:	PDA Ch3 214nm@4.8nm, PDA
Run Time:	15.0 Minutes	Proc. Chnl. Descr.:	PDA Ch3 214nm@4.8nm, PDA

Date Acquired: 6/10/2020 3:50:16 PM CST
Date Processed: 6/10/2020 4:12:48 PM CST, 6/10/2020 4:13:20 PM CST, 6/10/2020 4:14:02 PM CST

Compound 21

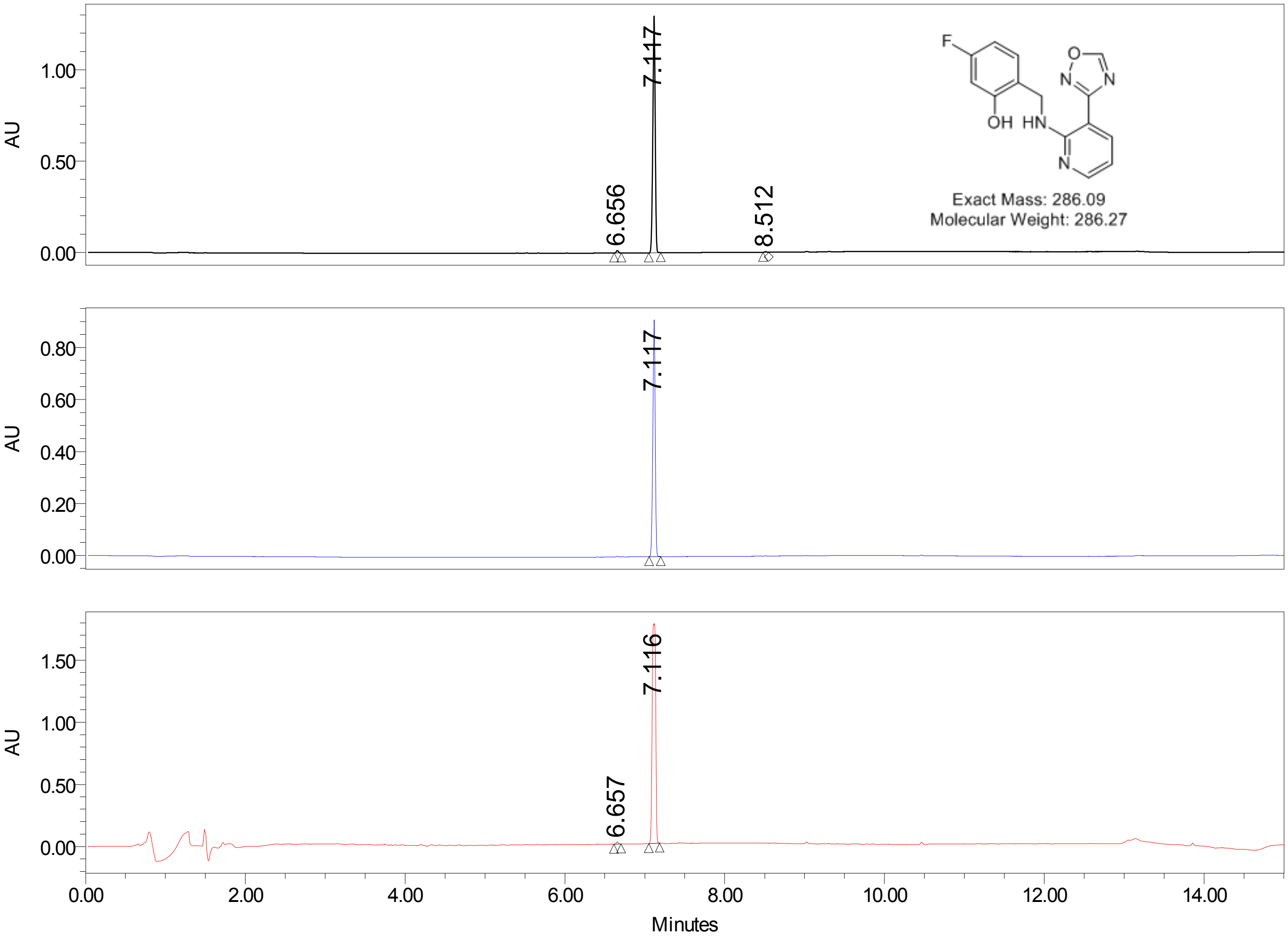

Channel: PDA Ch1 254nm@4.8nm; Processed Channel: PDA Ch1 254nm@4.8nm; Result Id: 13998; Processing Method: Process standrads method

Channel: PDA Ch2 280nm@4.8nm; Processed Channel: PDA Ch2 280nm@4.8nm; Result Id: 14000; Processing Method: Process standrads method

Channel: PDA Ch3 214nm@4.8nm; Processed Channel: PDA Ch3 214nm@4.8nm; Result Id: 13999; Processing Method: 214

Compound 22

Parameter	Value
1 Origin	Bruker BioSpin GmbH
2 Solvent	DMSO
3 Temperature	298.0
4 Number of Scans	16
5 Spectrometer Frequency	400.13

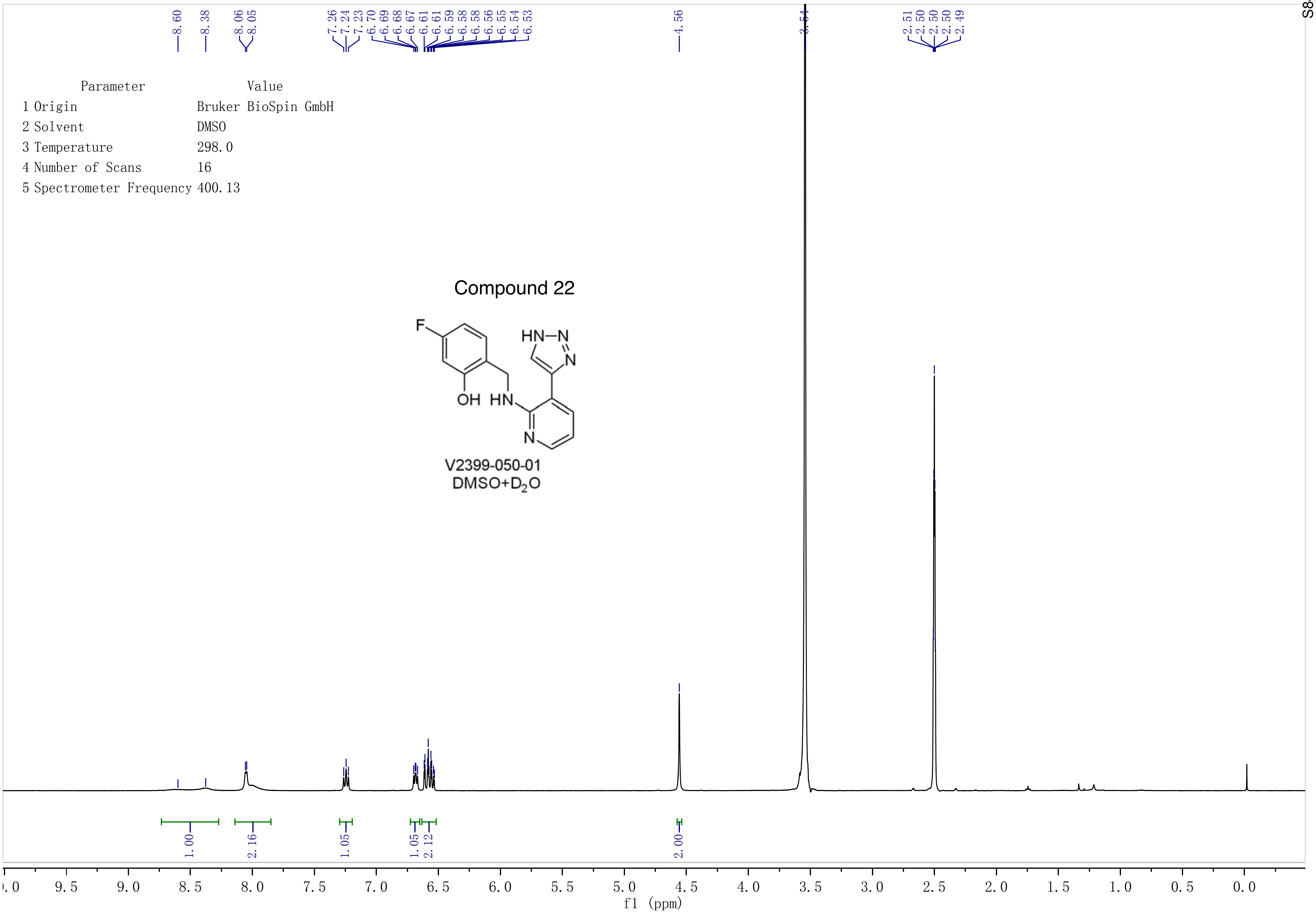

SAMPLE INFORMATION

Sample Name:	V2399-050-01	Acquired By:	System
Sample Type:	Standard	Sample Set Name	20200701
Vial:	1:B,8	Acq. Method Set:	VIVA QC_WATERS BEH C18
Injection #:	1	Processing Method	214, Process standrads method
Injection Volume:	0.90 ul	Channel Name:	PDA Ch3 214nm@4.8nm, PDA
Run Time:	15.0 Minutes	Proc. Chnl. Descr.:	PDA Ch3 214nm@4.8nm, PDA

Date Acquired: 7/1/2020 9:45:51 AM CST
Date Processed: 7/1/2020 10:33:34 AM CST, 7/1/2020 10:33:44 AM CST, 7/1/2020 10:34:02 AM CST

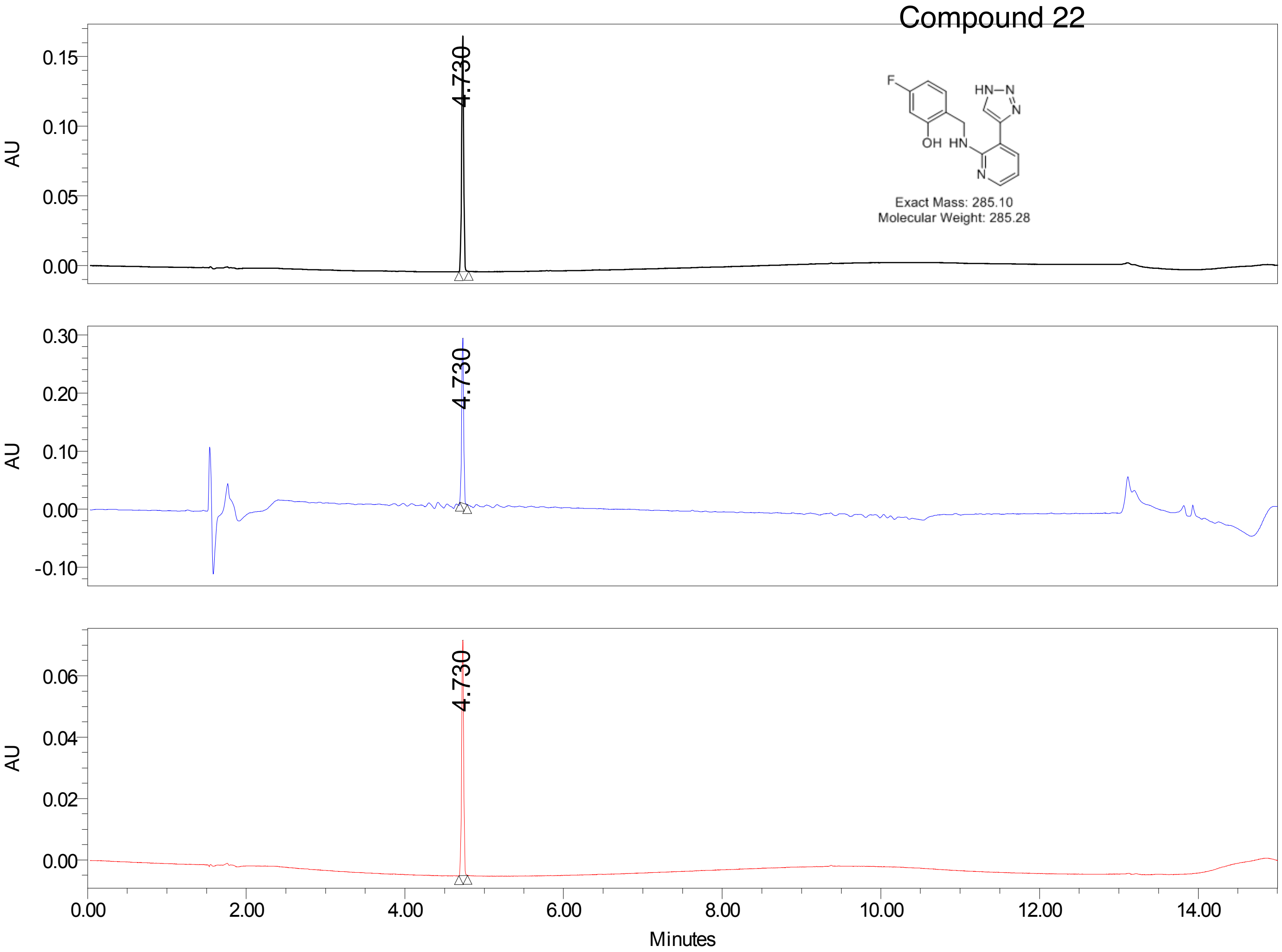

Channel: PDA Ch1 254nm@4.8nm; Processed Channel: PDA Ch1 254nm@4.8nm; Result Id: 16453; Processing Method: Process standrads method

Channel: PDA Ch3 214nm@4.8nm; Processed Channel: PDA Ch3 214nm@4.8nm; Result Id: 16455; Processing Method: 214

Channel: PDA Ch2 280nm@4.8nm; Processed Channel: PDA Ch2 280nm@4.8nm; Result Id: 16454; Processing Method: Process standrads method

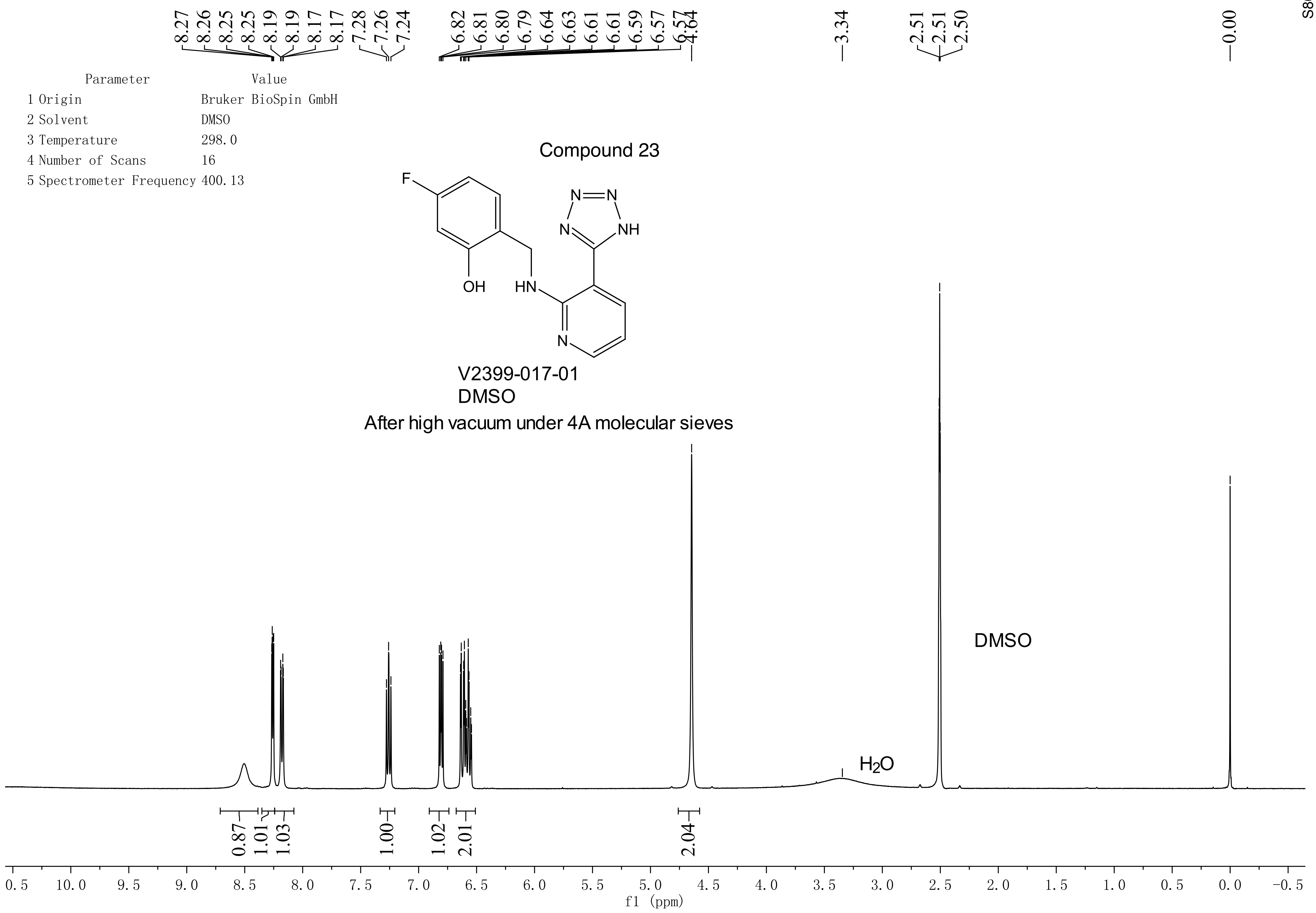
8.27
8.26
8.25
8.25
8.19
8.19
8.17
8.17
7.28
7.26
7.24
6.82
6.81
6.80
6.79
6.64
6.63
6.61
6.61
6.59
6.57
6.57
4.64
3.34
2.51
2.51
2.50
0.00
Parameter Value
1 Origin Bruker BioSpin GmbH
2 Solvent DMSO
3 Temperature 298.0
4 Number of Scans 16
5 Spectrometer Frequency 400.13
Compound 23
F
N=N
N
NH
OH
HN
N
V2399-017-01
DMSO
After high vacuum under 4A molecular sieves
DMSO
H2O
0.87
1.01
1.03
1.00
1.02
2.01
2.04
0.5 10.0 9.5 9.0 8.5 8.0 7.5 7.0 6.5 6.0 5.5 5.0 4.5 4.0 3.5 3.0 2.5 2.0 1.5 1.0 0.5 0.0 −0.5
f1 (ppm)

Injection Summary Report

SAMPLE INFORMATION

Sample Name:	V2399-017-01	Acquired By:	System
Sample Type:	Standard	Sample Set Name	20200609
Vial:	1:A,7	Acq. Method Set:	VIVA QC_WATERS BEH C18
Injection #:	1	Processing Method	214, Process standrads method
Injection Volume:	0.50 ul	Channel Name:	PDA Ch3 214nm@4.8nm, PDA
Run Time:	15.0 Minutes	Proc. Chnl. Descr.:	PDA Ch3 214nm@4.8nm, PDA

Date Acquired: 6/9/2020 3:27:29 PM CST
Date Processed: 6/9/2020 4:40:09 PM CST, 6/9/2020 4:40:21 PM CST, 6/9/2020 4:40:41 PM CST

Compound 23

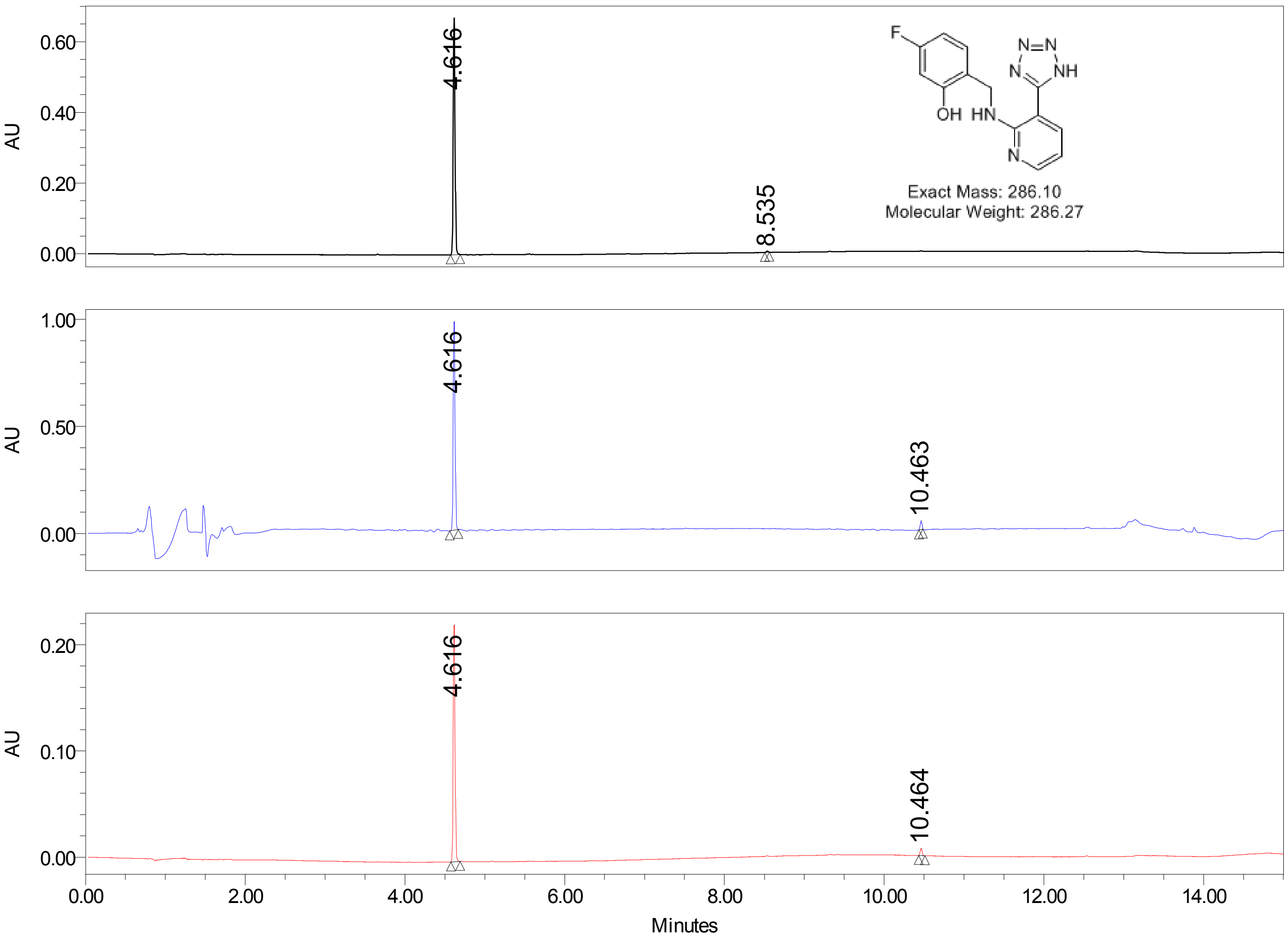

Channel: PDA Ch1 254nm@4.8nm; Processed Channel: PDA Ch1 254nm@4.8nm; Result Id: 13793; Processing Method: Process standrads method
Channel: PDA Ch3 214nm@4.8nm; Processed Channel: PDA Ch3 214nm@4.8nm; Result Id: 13795; Processing Method: 214
Channel: PDA Ch2 280nm@4.8nm; Processed Channel: PDA Ch2 280nm@4.8nm; Result Id: 13794; Processing Method: Process standrads method

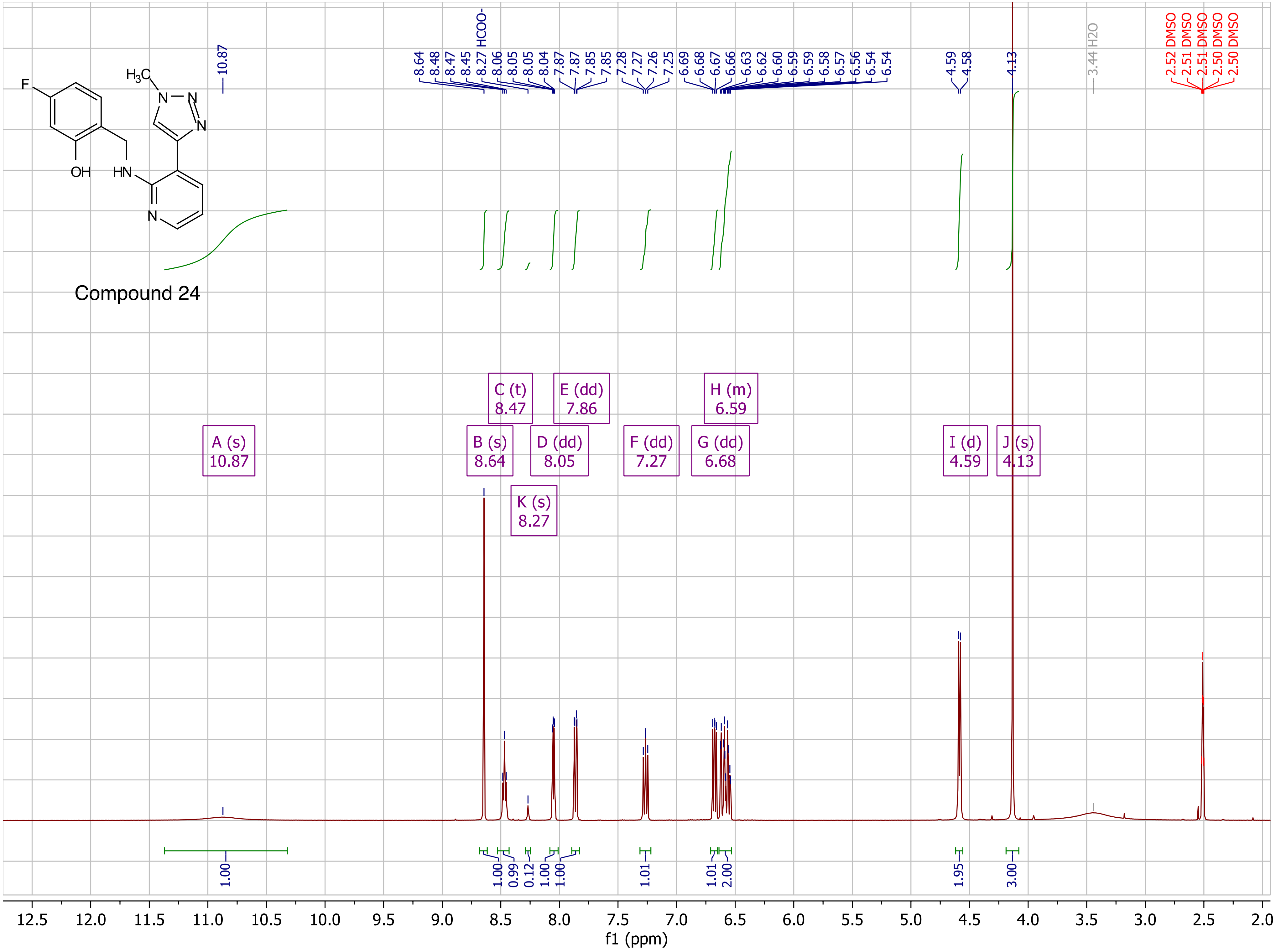
Compound 24
OH
HN
N
F
H_3C
10.87
8.64
8.48
8.47
8.45
8.27 HCOO-
8.06
8.05
8.05
8.04
7.87
7.87
7.85
7.85
7.28
7.27
7.26
7.25
6.69
6.68
6.67
6.66
6.63
6.62
6.60
6.59
6.59
6.58
6.57
6.56
6.54
6.54
4.59
4.58
4.13
3.44 H2O
2.52 DMSO
2.51 DMSO
2.51 DMSO
2.50 DMSO
2.50 DMSO
A (s) 10.87
B (s) 8.64
C (t) 8.47
D (dd) 8.05
E (dd) 7.86
F (dd) 7.27
G (dd) 6.68
H (m) 6.59
I (d) 4.59
J (s) 4.13
K (s) 8.27
1.00
1.00
0.99
0.12
1.00
1.00
1.01
1.01
2.00
1.95
3.00
f1 (ppm)

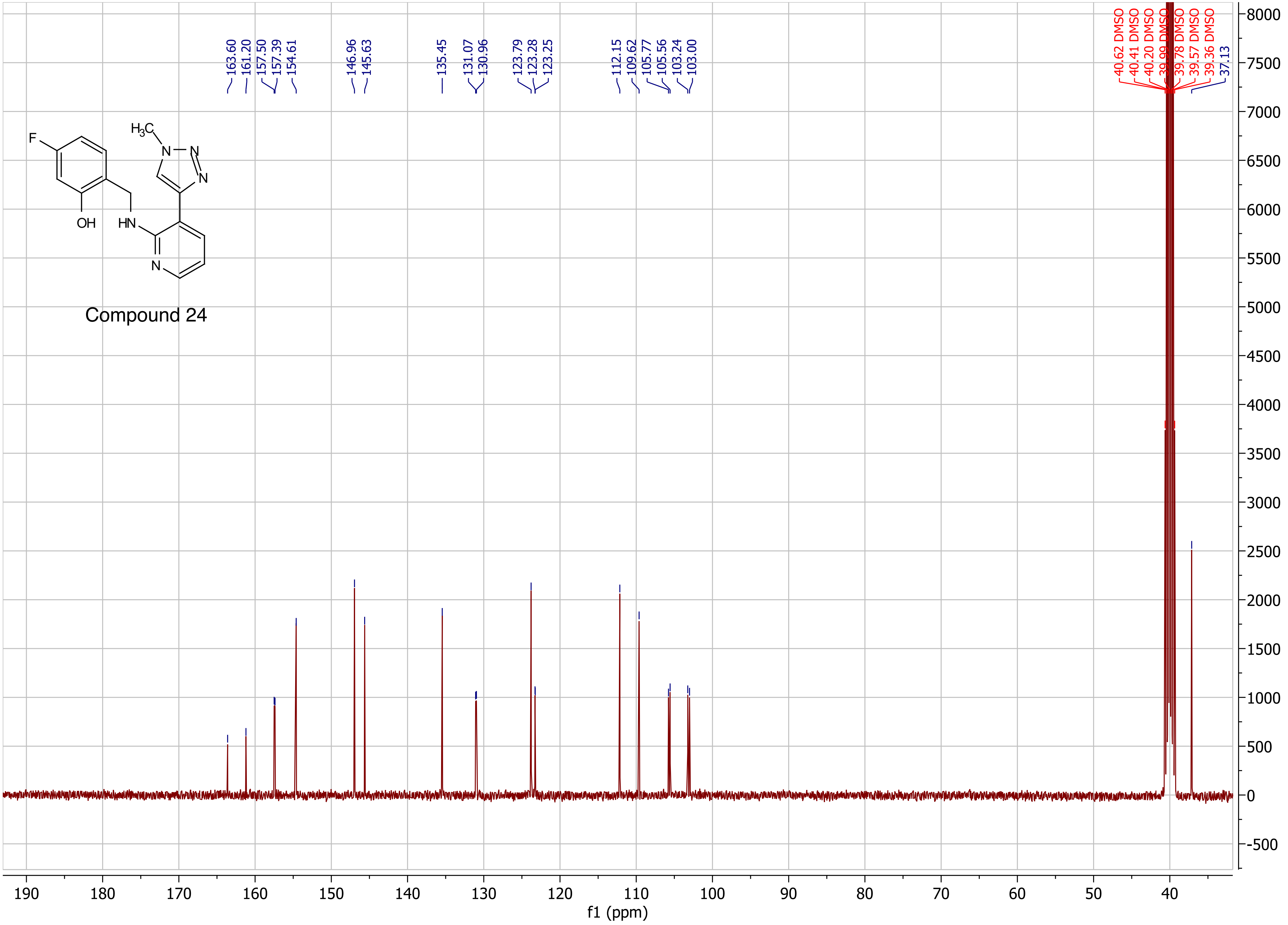
Compound 24
F
OH
HN
N
H3C
N
N
N
163.60
161.20
157.50
157.39
154.61
146.96
145.63
135.45
131.07
130.96
123.79
123.28
123.25
112.15
109.62
105.77
105.56
103.24
103.00
40.62 DMSO
40.41 DMSO
40.20 DMSO
39.99 DMSO
39.78 DMSO
39.57 DMSO
39.36 DMSO
37.13
f1 (ppm)
190 180 170 160 150 140 130 120 110 100 90 80 70 60 50 40
8000 7500 7000 6500 6000 5500 5000 4500 4000 3500 3000 2500 2000 1500 1000 500 0 -500

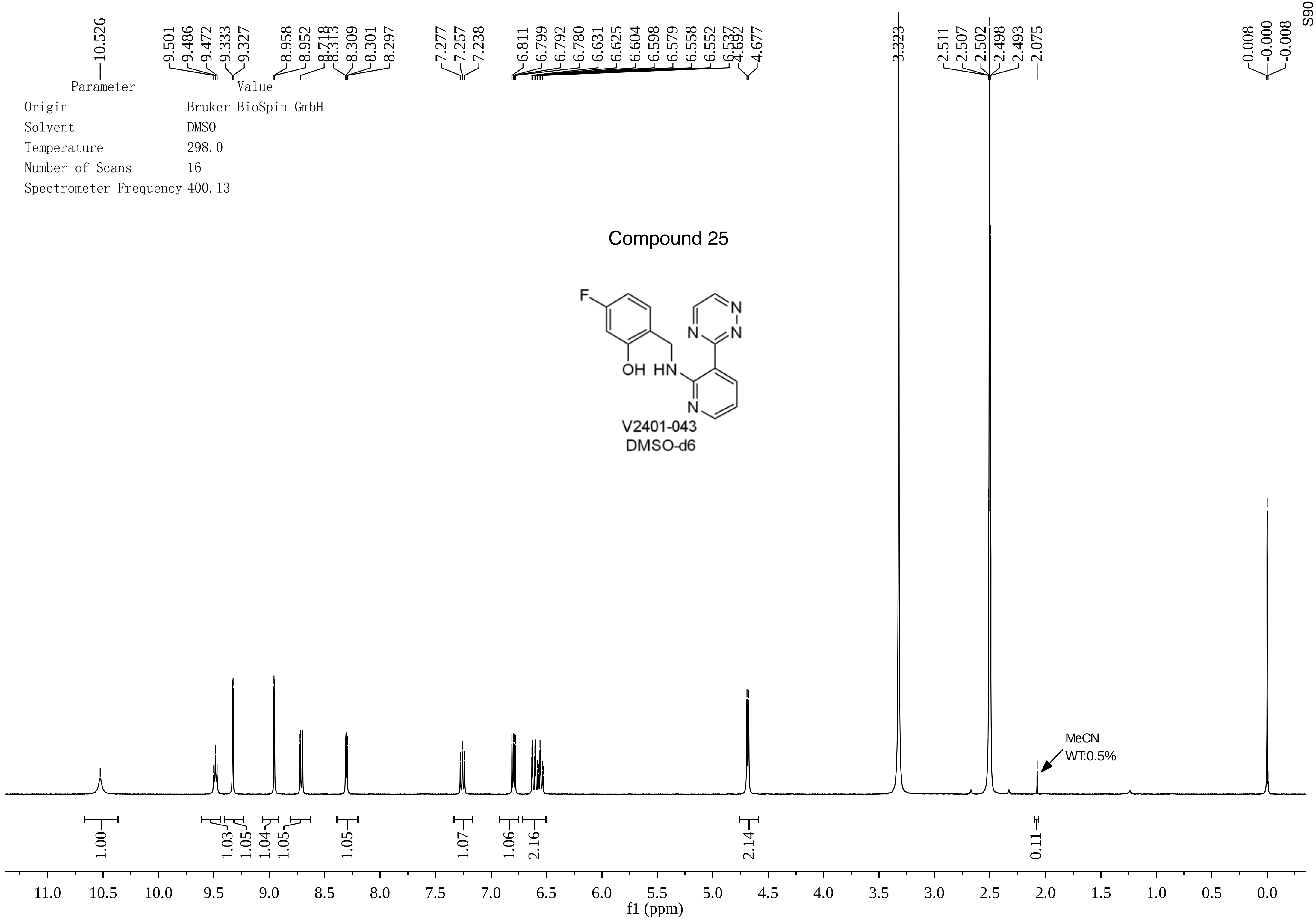
Parameter Value
Origin Bruker BioSpin GmbH
Solvent DMSO
Temperature 298.0
Number of Scans 16
Spectrometer Frequency 400.13
Compound 25
V2401-043
DMSO-d6
MeCN
WT:0.5%
10.526
9.501
9.486
9.472
9.333
9.327
8.958
8.952
8.718
8.313
8.309
8.301
8.297
7.277
7.257
7.238
6.811
6.799
6.792
6.780
6.631
6.625
6.604
6.598
6.579
6.558
6.552
6.537
4.692
4.677
3.323
2.511
2.507
2.502
2.498
2.493
2.075
0.008
-0.000
-0.008
1.00
1.03
1.05
1.04
1.05
1.05
1.07
1.06
2.16
2.14
0.11
f1 (ppm)

Injection Summary Report

SAMPLE INFORMATION

Sample Name:	V2401-043	Acquired By:	System
Sample Type:	Standard	Sample Set Name	20200629
Vial:	1:A,7	Acq. Method Set:	VIVA QC_WATERS BEH C18
Injection #:	1	Processing Method	214, Process standrads method
Injection Volume:	0.50 ul	Channel Name:	PDA Ch3 214nm@4.8nm, PDA
Run Time:	15.0 Minutes	Proc. Chnl. Descr.:	PDA Ch3 214nm@4.8nm, PDA

Date Acquired: 6/29/2020 10:11:43 AM CST
Date Processed: 6/29/2020 10:33:46 AM CST, 6/29/2020 10:34:06 AM CST, 6/29/2020 10:34:38 AM

Compound 25

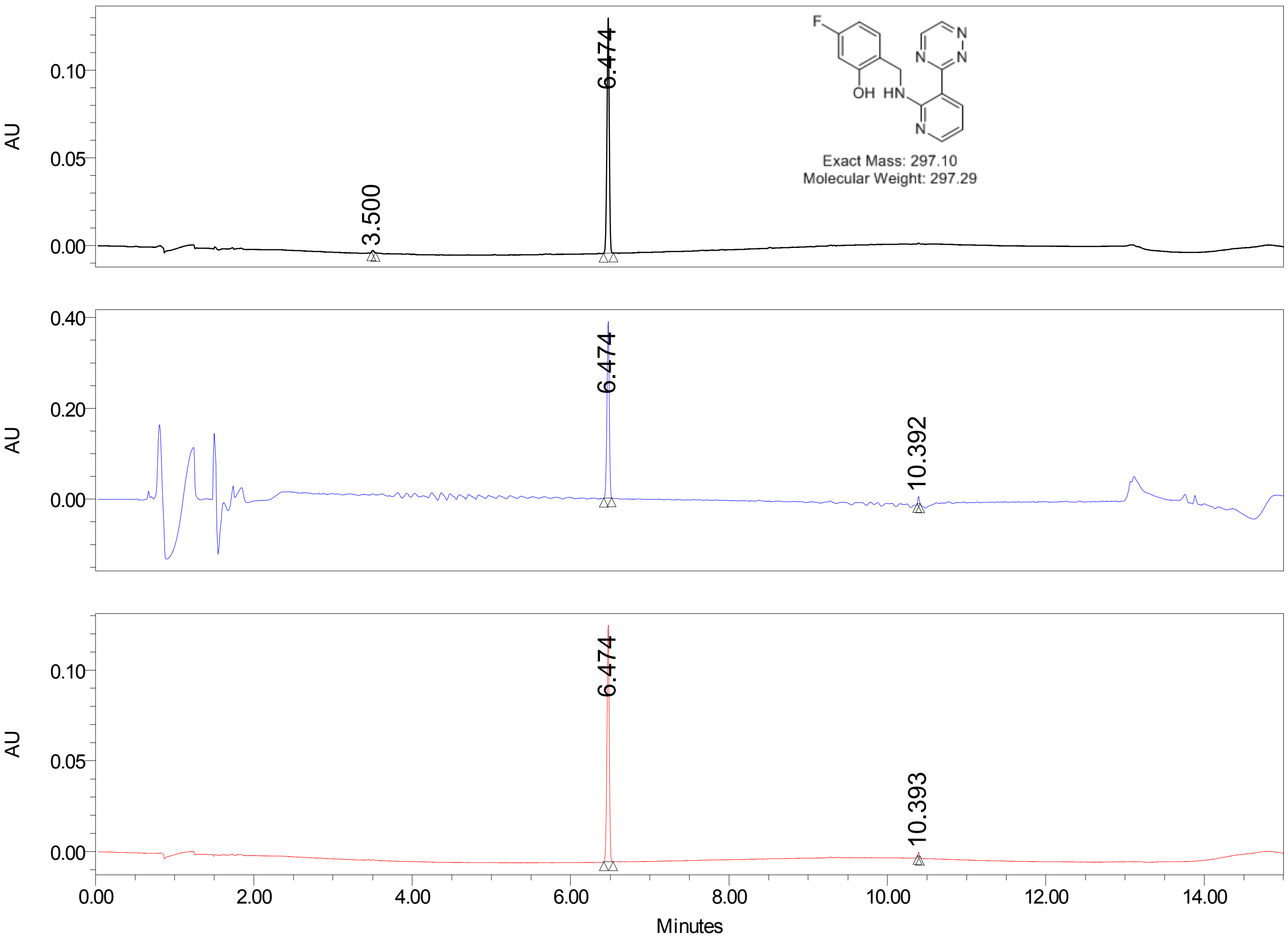

Channel: PDA Ch1 254nm@4.8nm; Processed Channel: PDA Ch1 254nm@4.8nm; Result Id: 16304; Processing Method: Process standrads method

Channel: PDA Ch3 214nm@4.8nm; Processed Channel: PDA Ch3 214nm@4.8nm; Result Id: 16306; Processing Method: 214

Channel: PDA Ch2 280nm@4.8nm; Processed Channel: PDA Ch2 280nm@4.8nm; Result Id: 16305; Processing Method: Process standrads method

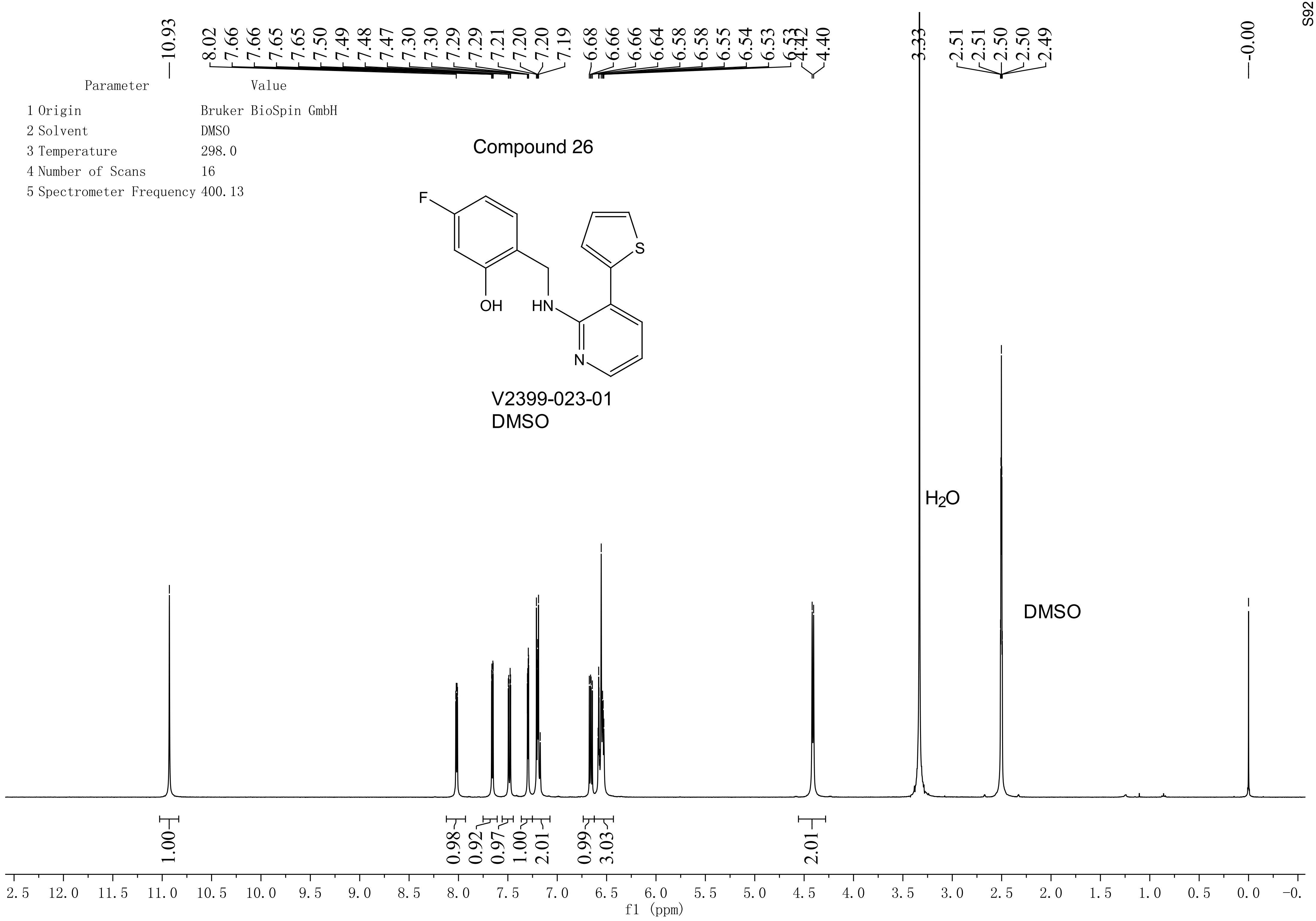

Parameter Value
1 Origin Bruker BioSpin GmbH
2 Solvent DMSO
3 Temperature 298.0
4 Number of Scans 16
5 Spectrometer Frequency 400.13
Compound 26
F
OH
HN
N
S
V2399-023-01
DMSO
10.93
8.02 7.66 7.66 7.65 7.65 7.50 7.49 7.48 7.47 7.30 7.30 7.29 7.29 7.21 7.20 7.20 7.19
6.68 6.66 6.66 6.64 6.58 6.58 6.55 6.54 6.53 6.53
4.42 4.40
3.33
2.51 2.51 2.50 2.50 2.49
-0.00
H2O
DMSO
1.00
0.98 0.92 0.97 1.00 2.01
0.99 3.03
2.01
2.5 12.0 11.5 11.0 10.5 10.0 9.5 9.0 8.5 8.0 7.5 7.0 6.5 6.0 5.5 5.0 4.5 4.0 3.5 3.0 2.5 2.0 1.5 1.0 0.5 0.0 -0.
f1 (ppm)

Compound 26

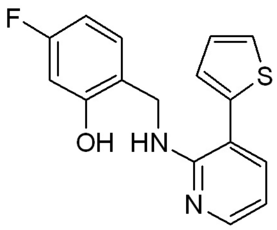

ELG-000005
DMSO-d_6
Chemical Formula: $C_{16}H_{13}FN_2OS$

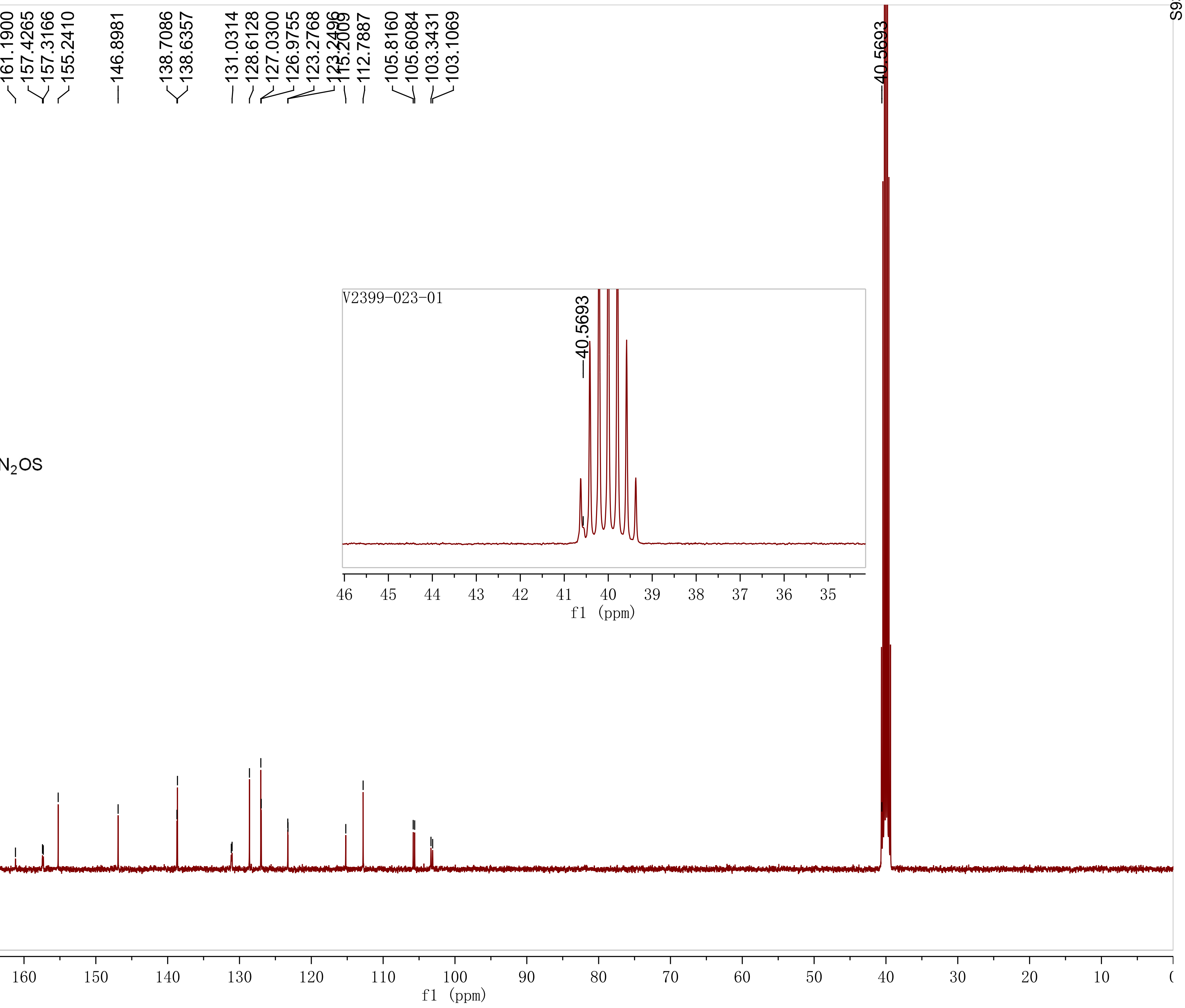

Injection Summary Report

SAMPLE INFORMATION

Sample Name:	V2399-023-01	Acquired By:	System
Sample Type:	Standard	Sample Set Name	20200611
Vial:	1:E,3	Acq. Method Set:	VIVA QC_WATERS BEH C18
Injection #:	1	Processing Method	214, Process standrads method
Injection Volume:	0.50 ul	Channel Name:	PDA Ch3 214nm@4.8nm, PDA
Run Time:	15.0 Minutes	Proc. Chnl. Descr.:	PDA Ch3 214nm@4.8nm, PDA

Date Acquired: 6/11/2020 2:48:01 PM CST
Date Processed: 6/11/2020 3:05:42 PM CST, 6/11/2020 3:05:52 PM CST, 6/11/2020 3:06:11 PM CST

Compound 26

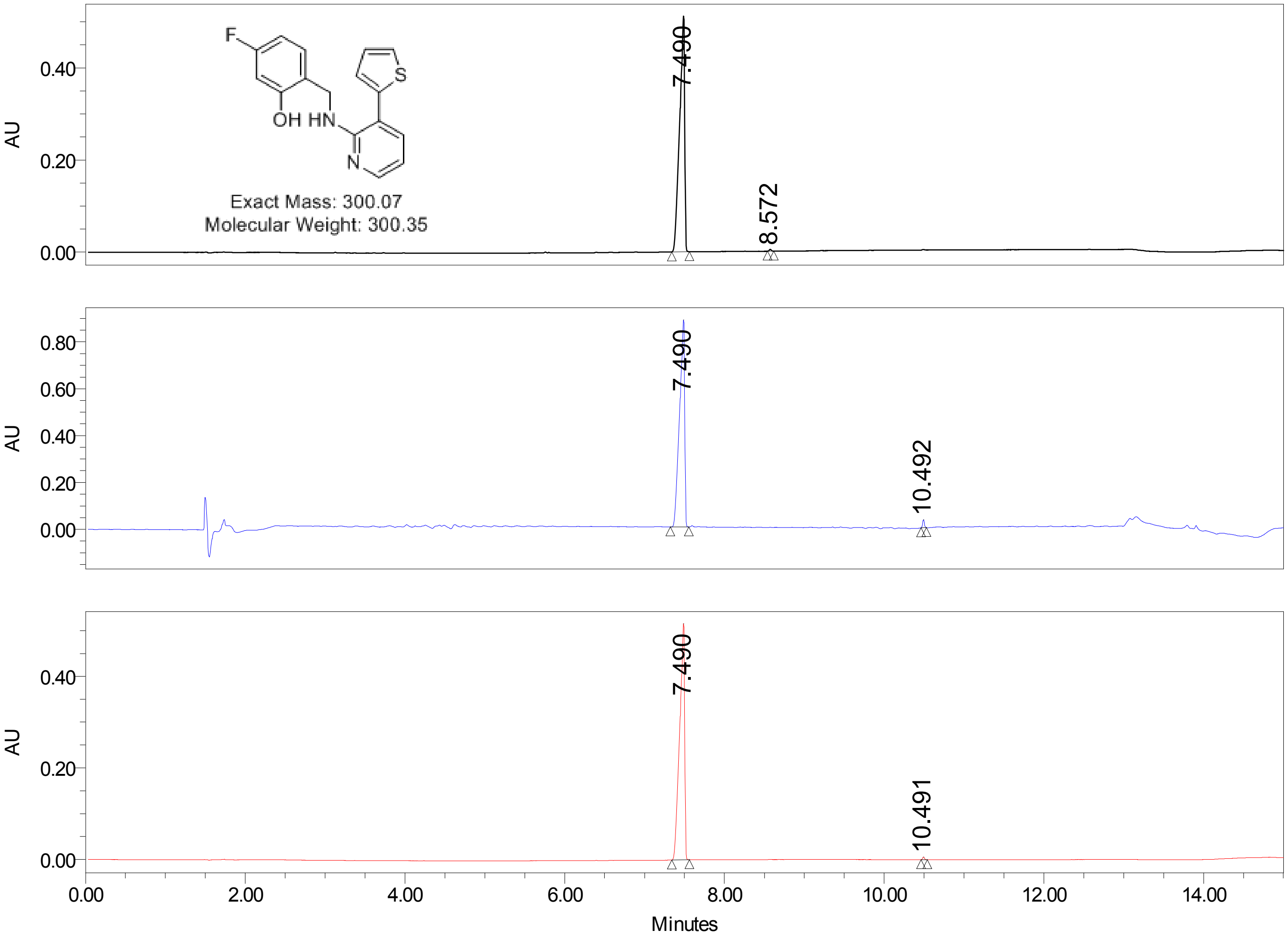

Channel: PDA Ch1 254nm@4.8nm; Processed Channel: PDA Ch1 254nm@4.8nm; Result Id: 14175; Processing Method: Process standrads method

Channel: PDA Ch3 214nm@4.8nm; Processed Channel: PDA Ch3 214nm@4.8nm; Result Id: 14177; Processing Method: 214

Channel: PDA Ch2 280nm@4.8nm; Processed Channel: PDA Ch2 280nm@4.8nm; Result Id: 14176; Processing Method: Process standrads method

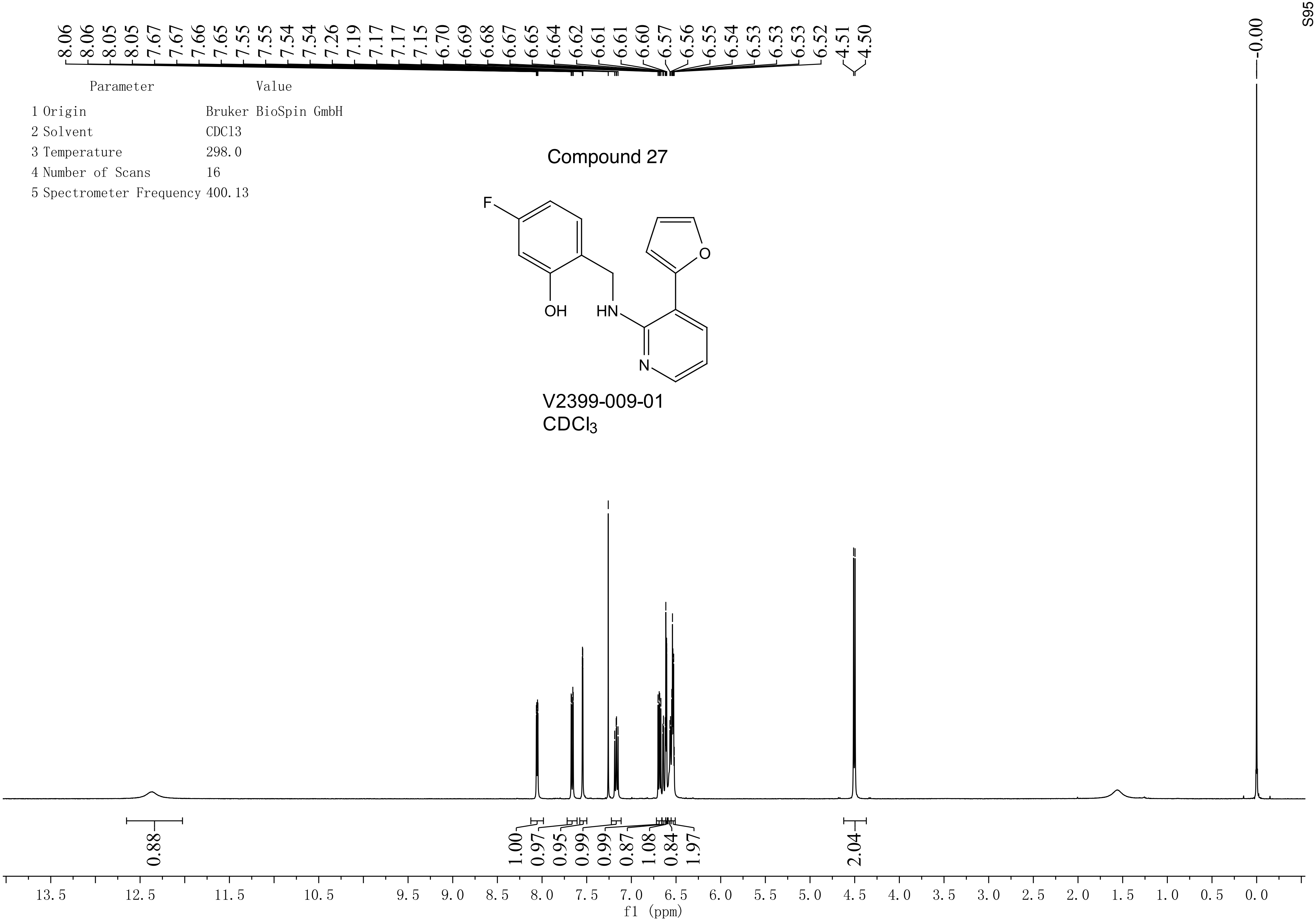
8.06 8.06 8.05 8.05 7.67 7.67 7.66 7.65 7.55 7.55 7.54 7.54 7.26 7.19 7.17 7.17 7.15 6.70 6.69 6.68 6.67 6.65 6.64 6.62 6.61 6.61 6.60 6.57 6.56 6.55 6.54 6.53 6.53 6.53 6.52 4.51 4.50
-0.00
Parameter Value
1 Origin Bruker BioSpin GmbH
2 Solvent CDCl3
3 Temperature 298.0
4 Number of Scans 16
5 Spectrometer Frequency 400.13
Compound 27
F
OH
HN
N
O
V2399-009-01
CDCl3
0.88
1.00 0.97 0.95 0.99 0.99 0.87 1.08 0.84 1.97
2.04
13.5 12.5 11.5 10.5 9.5 9.0 8.5 8.0 7.5 7.0 6.5 6.0 5.5 5.0 4.5 4.0 3.5 3.0 2.5 2.0 1.5 1.0 0.5 0.0
f1 (ppm)

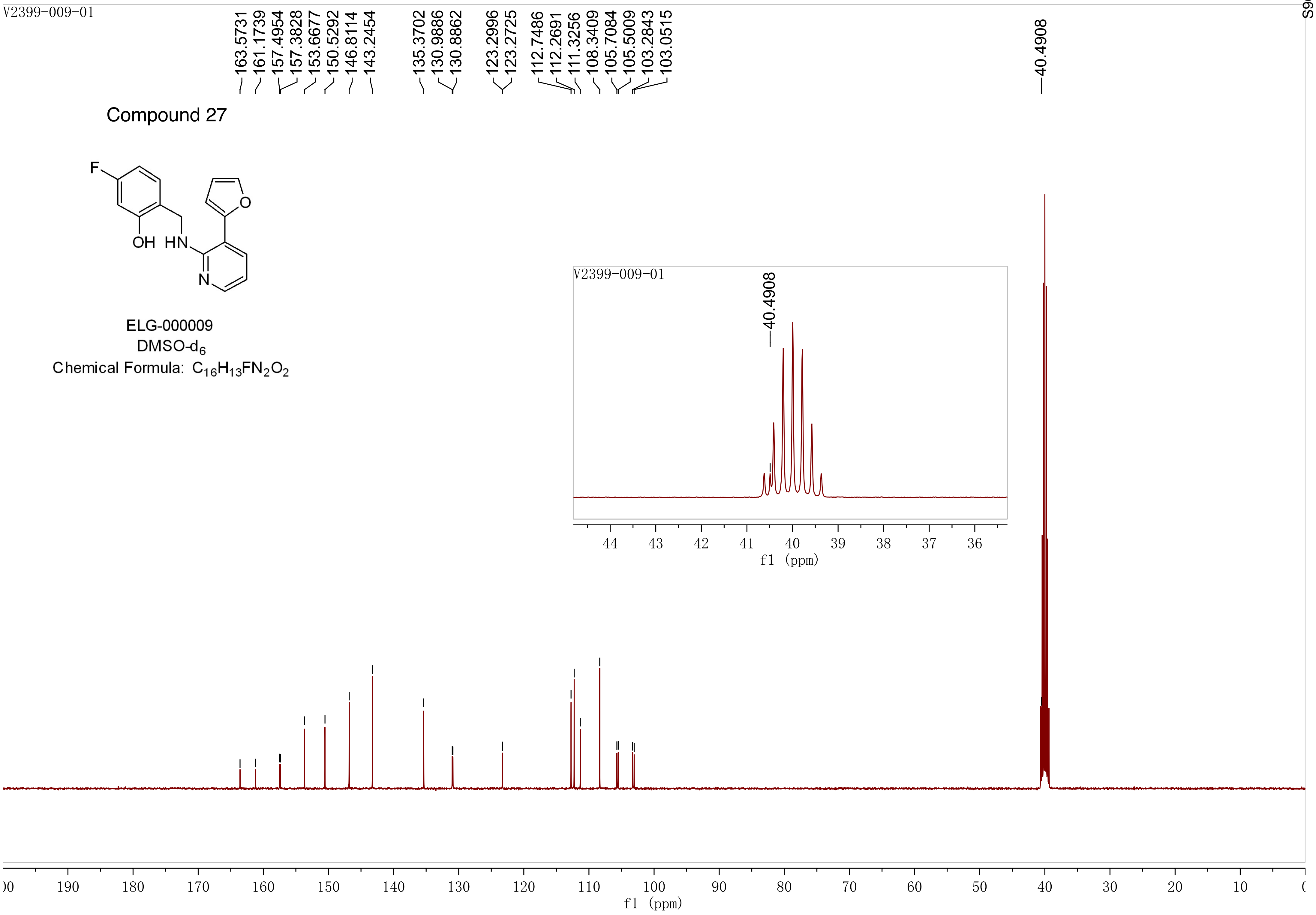
V2399-009-01
163.5731
161.1739
157.4954
157.3828
153.6677
150.5292
146.8114
143.2454
135.3702
130.9886
130.8862
123.2996
123.2725
112.7486
112.2691
111.3256
108.3409
105.7084
105.5009
103.2843
103.0515
40.4908
Compound 27
F
OH HN
N
O
ELG-000009
DMSO-d6
Chemical Formula: C16H13FN2O2
V2399-009-01
40.4908
44 43 42 41 40 39 38 37 36
f1 (ppm)
190 180 170 160 150 140 130 120 110 100 90 80 70 60 50 40 30 20 10
f1 (ppm)

Injection Summary Report

SAMPLE INFORMATION

Sample Name:	V2399-009-01	Acquired By:	System
Sample Type:	Standard	Sample Set Name	20200608
Vial:	2:F,4	Acq. Method Set:	VIVA QC_WATERS BEH C18
Injection #:	1	Processing Method	214, Process standrads method
Injection Volume:	0.50 ul	Channel Name:	PDA Ch3 214nm@4.8nm, PDA
Run Time:	15.0 Minutes	Proc. Chnl. Descr.:	PDA Ch3 214nm@4.8nm, PDA

Date Acquired: 6/8/2020 5:00:55 PM CST
Date Processed: 6/9/2020 8:38:44 AM CST, 6/9/2020 8:38:57 AM CST, 6/9/2020 8:39:08 AM CST

Compound 27

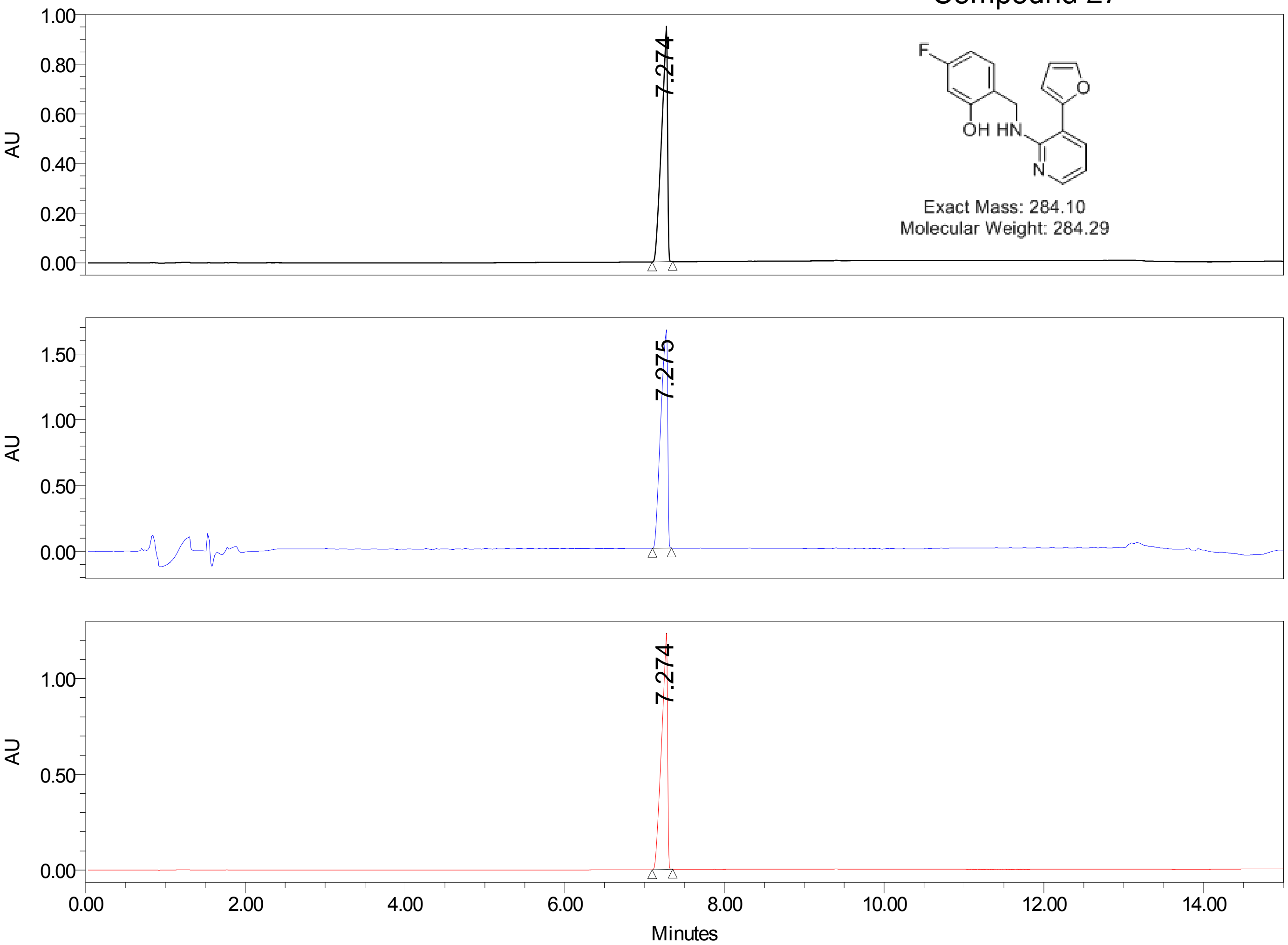

Channel: PDA Ch1 254nm@4.8nm; Processed Channel: PDA Ch1 254nm@4.8nm; Result Id: 13654; Processing Method: Process standrads method

Channel: PDA Ch3 214nm@4.8nm; Processed Channel: PDA Ch3 214nm@4.8nm; Result Id: 13656; Processing Method: 214

Channel: PDA Ch2 280nm@4.8nm; Processed Channel: PDA Ch2 280nm@4.8nm; Result Id: 13655; Processing Method: Process standrads method

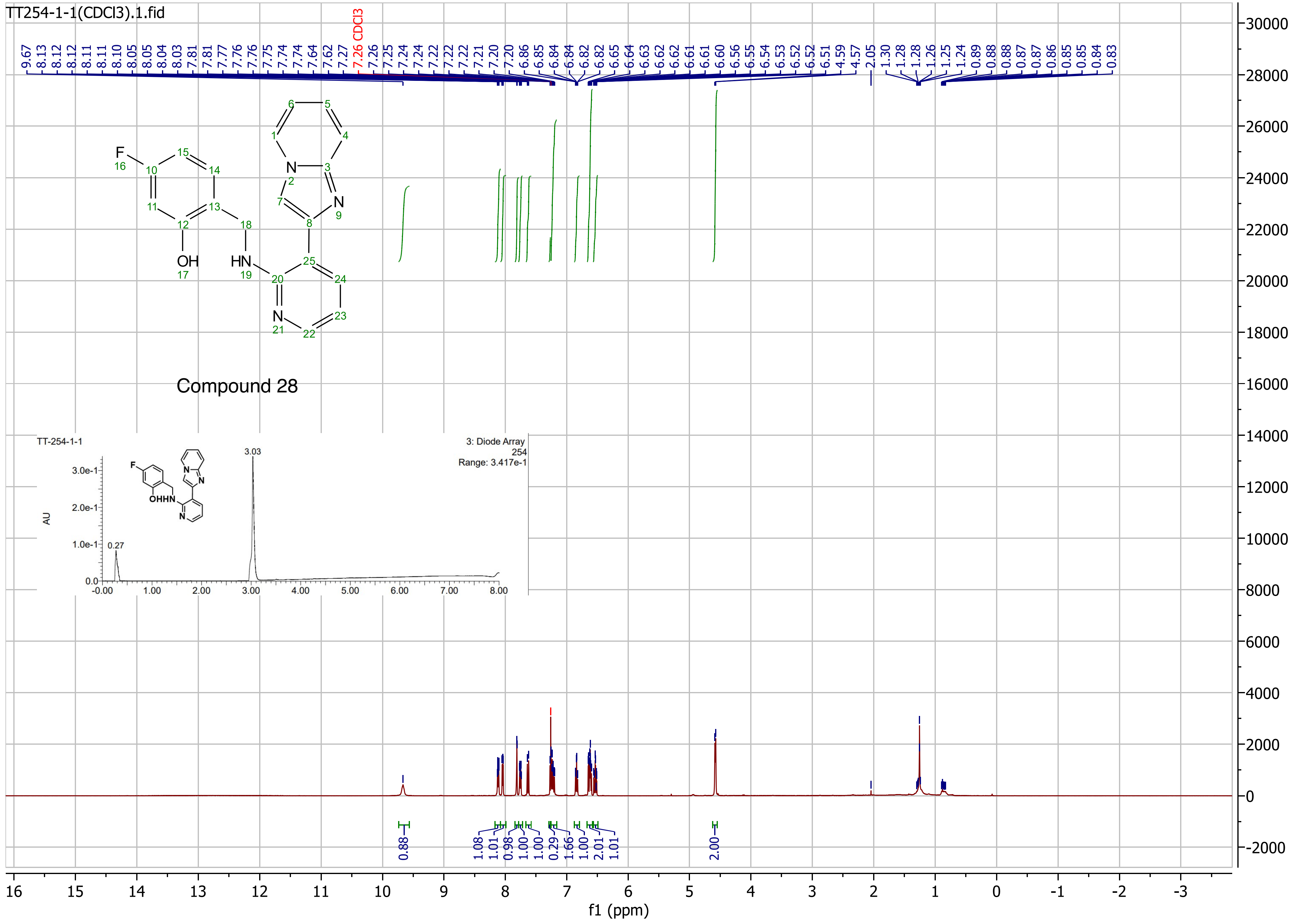

TT254-1-1(CDCl3).1.fid
9.67 8.13 8.12 8.12 8.11 8.11 8.10 8.05 8.05 8.04 8.03 7.81 7.81 7.77 7.76 7.76 7.75 7.74 7.74 7.64 7.62 7.27 7.26 CDCl3 7.26 7.25 7.24 7.24 7.22 7.22 7.22 7.21 7.20 7.20 6.86 6.85 6.84 6.84 6.82 6.82 6.65 6.64 6.63 6.62 6.62 6.61 6.61 6.60 6.56 6.55 6.54 6.53 6.52 6.52 6.51 4.59 4.57 2.05 1.30 1.28 1.28 1.26 1.25 1.24 0.89 0.88 0.88 0.87 0.87 0.86 0.85 0.85 0.84 0.83
Compound 28
TT-254-1-1
3: Diode Array
254
Range: 3.417e-1
AU
0.27
3.03
0.88 1.08 1.01 0.98 1.00 1.00 0.29 1.66 1.00 2.01 1.01 2.00
f1 (ppm)

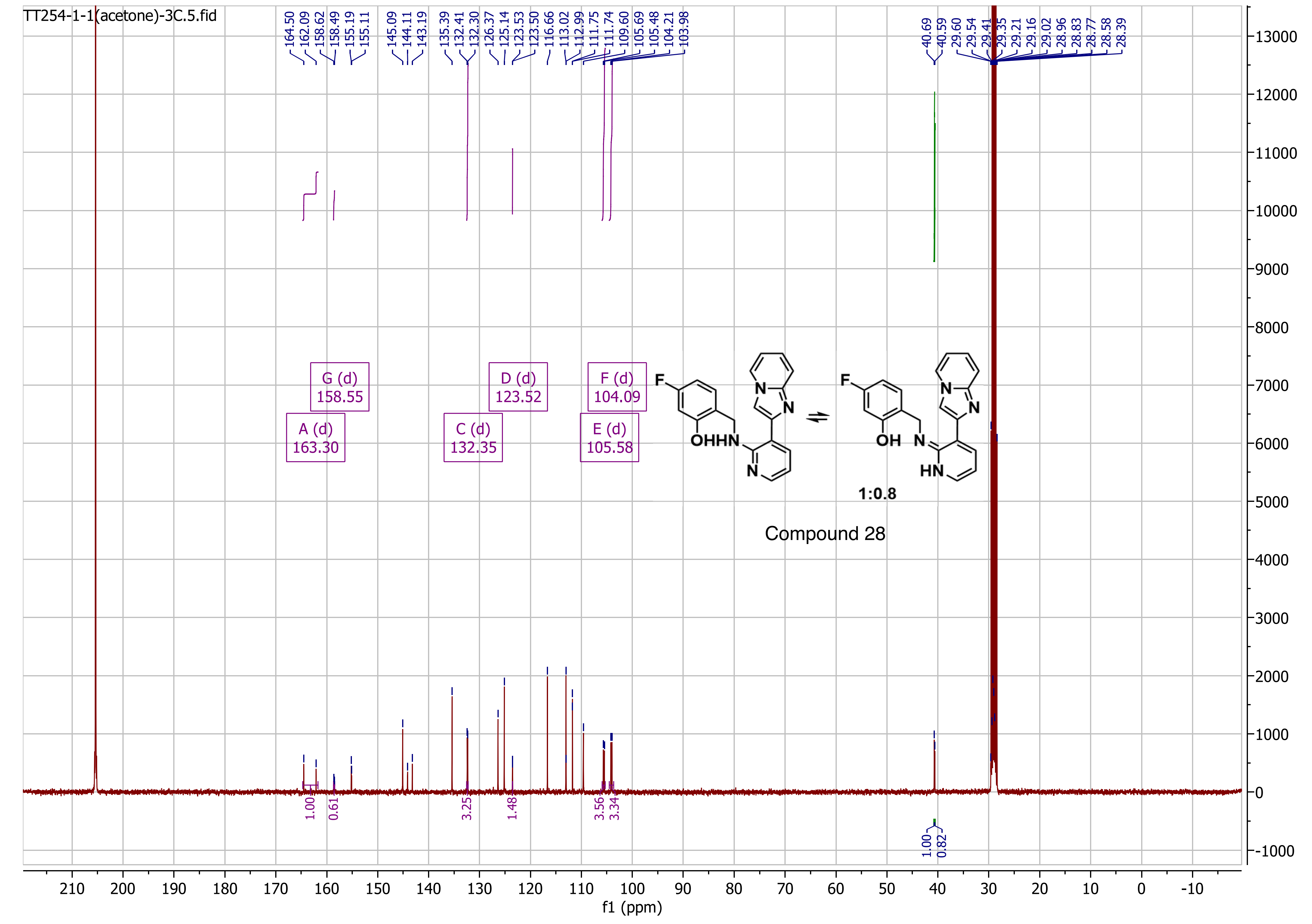
TT254-1-1(acetone)-3C.5.fid
164.50
162.09
158.62
158.49
155.19
155.11
145.09
144.11
143.19
135.39
132.41
132.30
126.37
125.14
123.53
123.50
116.66
113.02
112.99
111.75
111.74
109.60
105.69
105.48
104.21
103.98
40.69
40.59
29.60
29.54
29.41
29.35
29.21
29.16
29.02
28.96
28.83
28.77
28.58
28.39
G (d) 158.55
A (d) 163.30
D (d) 123.52
C (d) 132.35
F (d) 104.09
E (d) 105.58
F
OHHN
N
OH
HN
1:0.8
Compound 28
1.00
0.61
3.25
1.48
3.56
3.34
1.00
0.82
f1 (ppm)

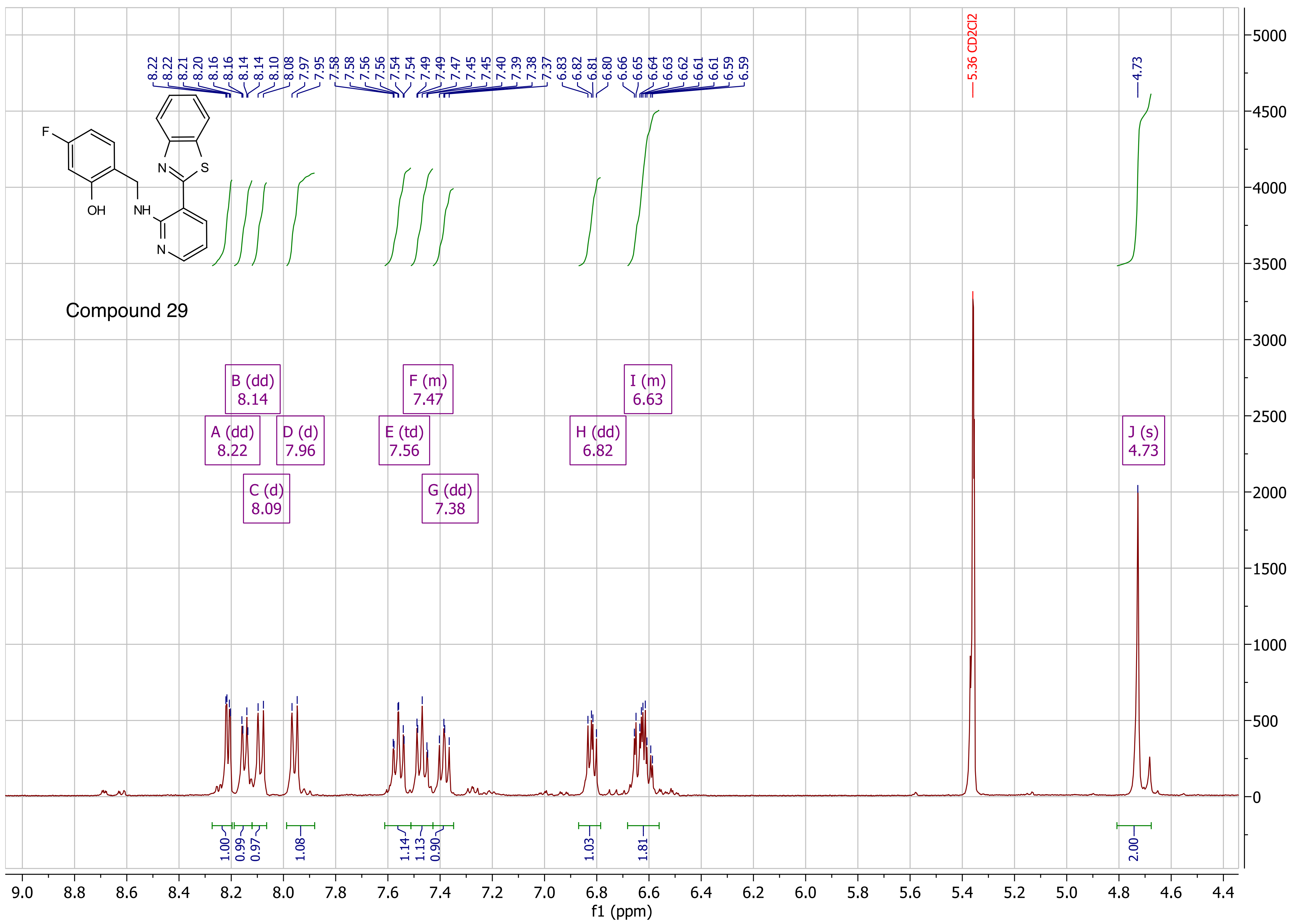
Compound 29
OH
NH
N
N
S
F
8.22
8.22
8.21
8.20
8.16
8.16
8.14
8.14
8.10
8.08
7.97
7.95
7.58
7.58
7.56
7.56
7.54
7.54
7.49
7.49
7.47
7.45
7.45
7.40
7.39
7.38
7.37
6.83
6.82
6.81
6.80
6.66
6.65
6.64
6.63
6.62
6.61
6.61
6.59
6.59
5.36 CD2Cl2
4.73
A (dd) 8.22
B (dd) 8.14
C (d) 8.09
D (d) 7.96
E (td) 7.56
F (m) 7.47
G (dd) 7.38
H (dd) 6.82
I (m) 6.63
J (s) 4.73
1.00
0.99
0.97
1.08
1.14
1.13
0.90
1.03
1.81
2.00
f1 (ppm)

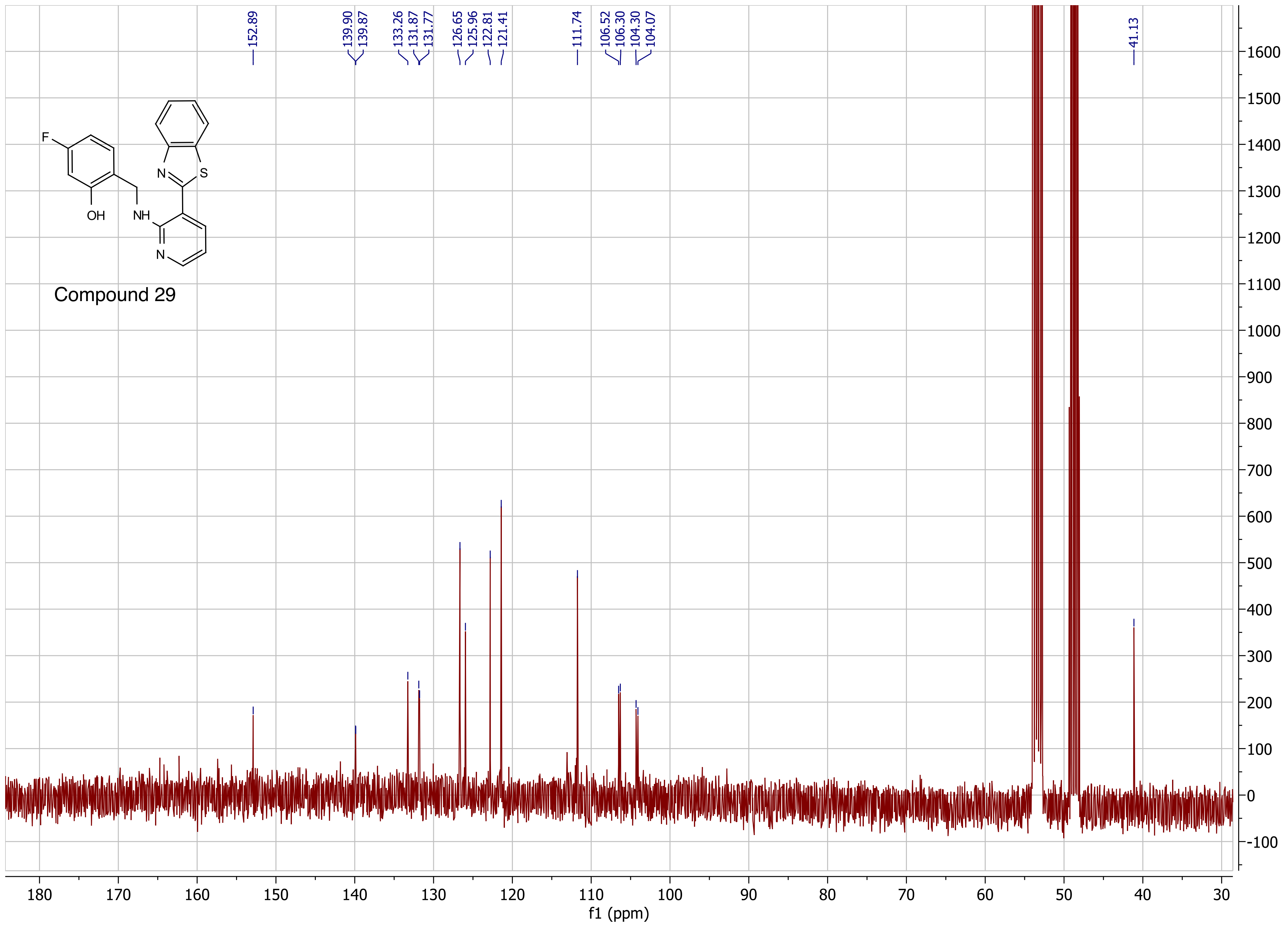
152.89
139.90
139.87
133.26
131.87
131.77
126.65
125.96
122.81
121.41
111.74
106.52
106.30
104.30
104.07
41.13
F
OH
NH
N
S
N
Compound 29
180 170 160 150 140 130 120 110 100 90 80 70 60 50 40 30
f1 (ppm)
1600 1500 1400 1300 1200 1100 1000 900 800 700 600 500 400 300 200 100 0 -100

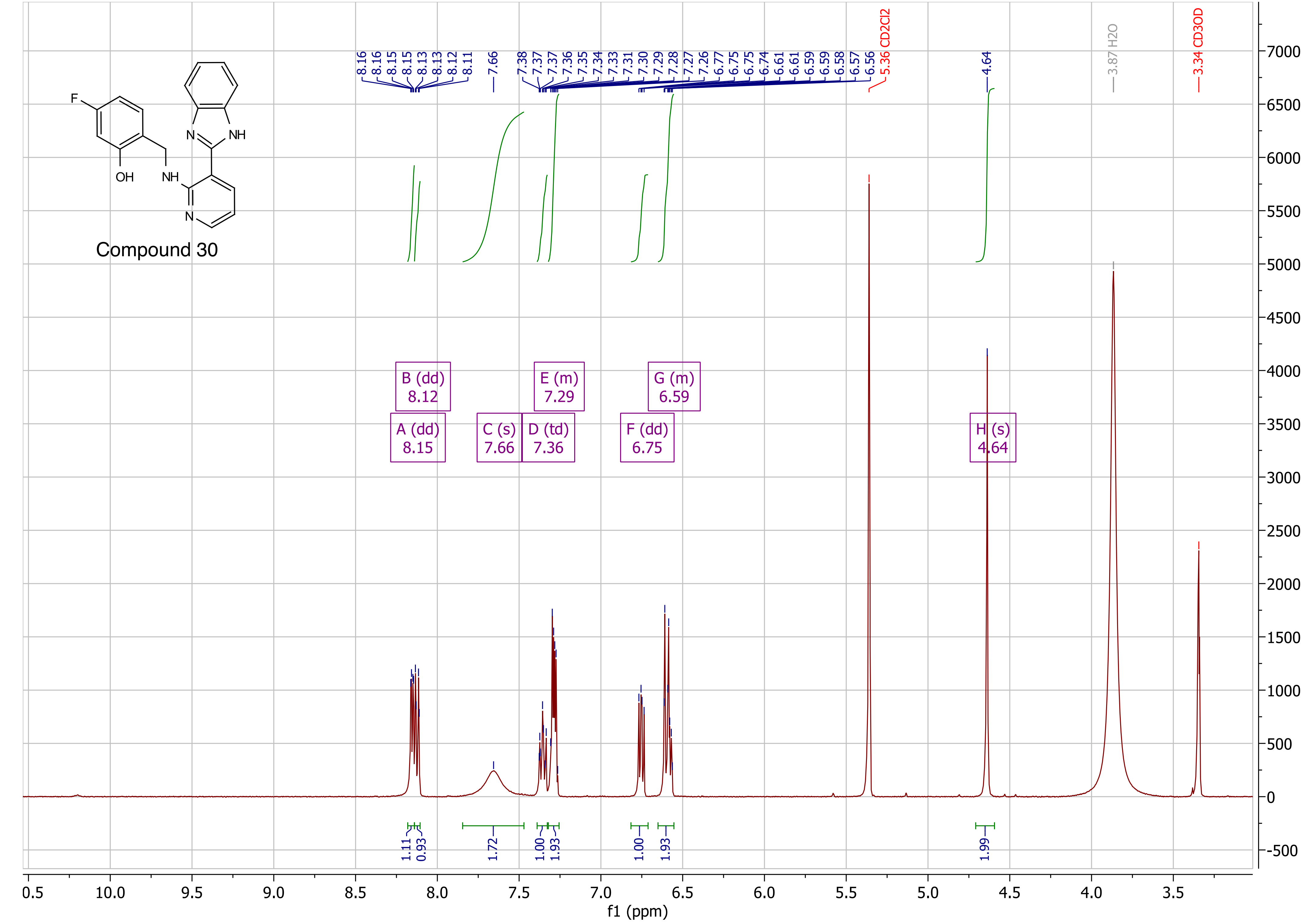
Compound 30
F
OH
NH
N
N
NH
8.16
8.16
8.15
8.15
8.13
8.13
8.12
8.11
7.66
7.38
7.37
7.37
7.36
7.35
7.34
7.33
7.31
7.30
7.29
7.28
7.27
7.26
6.77
6.75
6.75
6.74
6.61
6.61
6.59
6.59
6.58
6.57
6.56
5.36 CD2Cl2
4.64
3.87 H2O
3.34 CD3OD
B (dd) 8.12
A (dd) 8.15
C (s) 7.66
D (td) 7.36
E (m) 7.29
F (dd) 6.75
G (m) 6.59
H (s) 4.64
1.11
0.93
1.72
1.00
1.93
1.00
1.93
1.99
f1 (ppm)

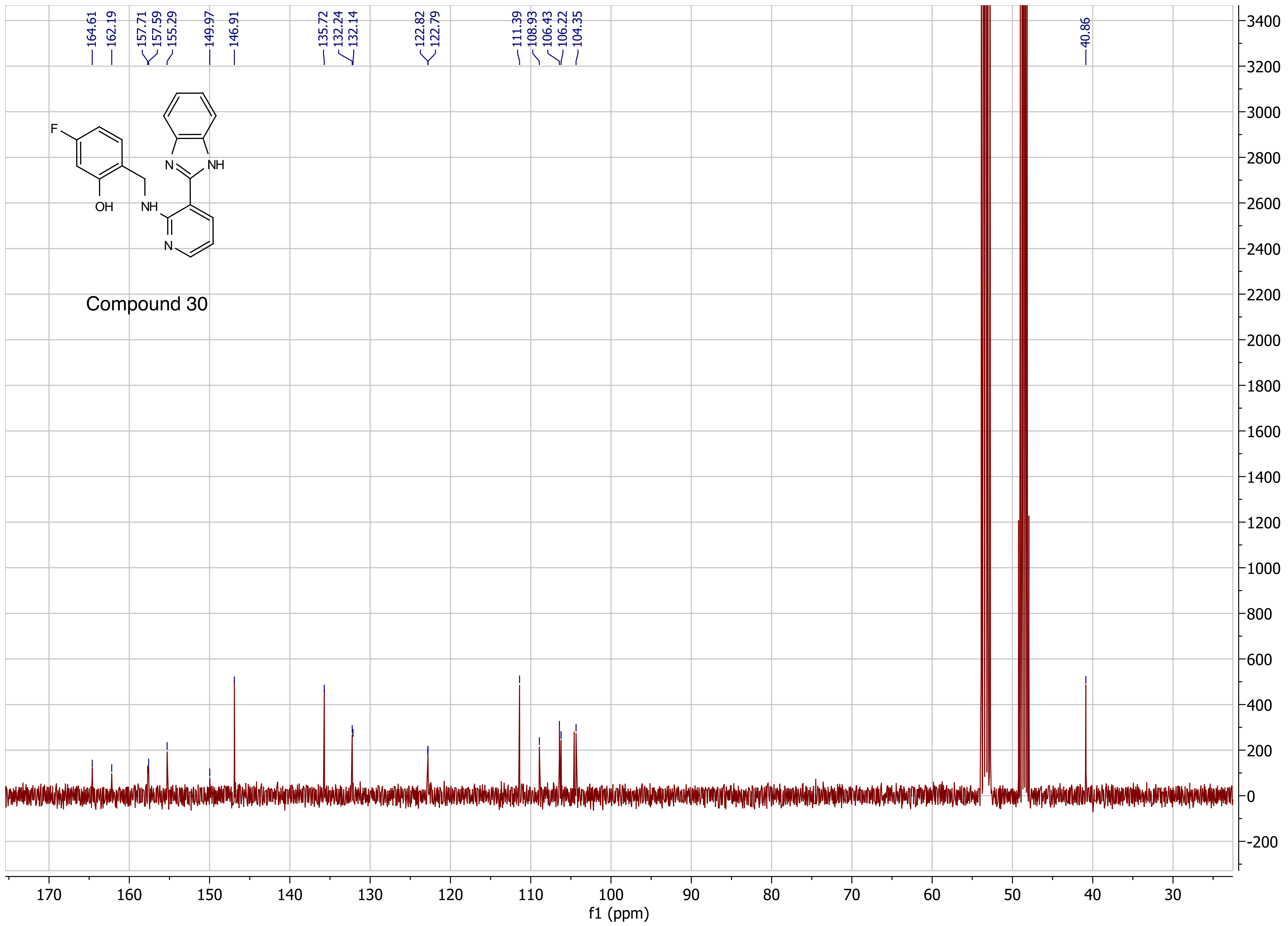

164.61
162.19
157.71
157.59
155.29
149.97
146.91
135.72
132.24
132.14
122.82
122.79
111.39
108.93
106.43
106.22
104.35
40.86
F
OH
NH
N
NH
N
Compound 30
170
160
150
140
130
120
110
100
90
80
70
60
50
40
30
f1 (ppm)
3400
3200
3000
2800
2600
2400
2200
2000
1800
1600
1400
1200
1000
800
600
400
200
0
-200

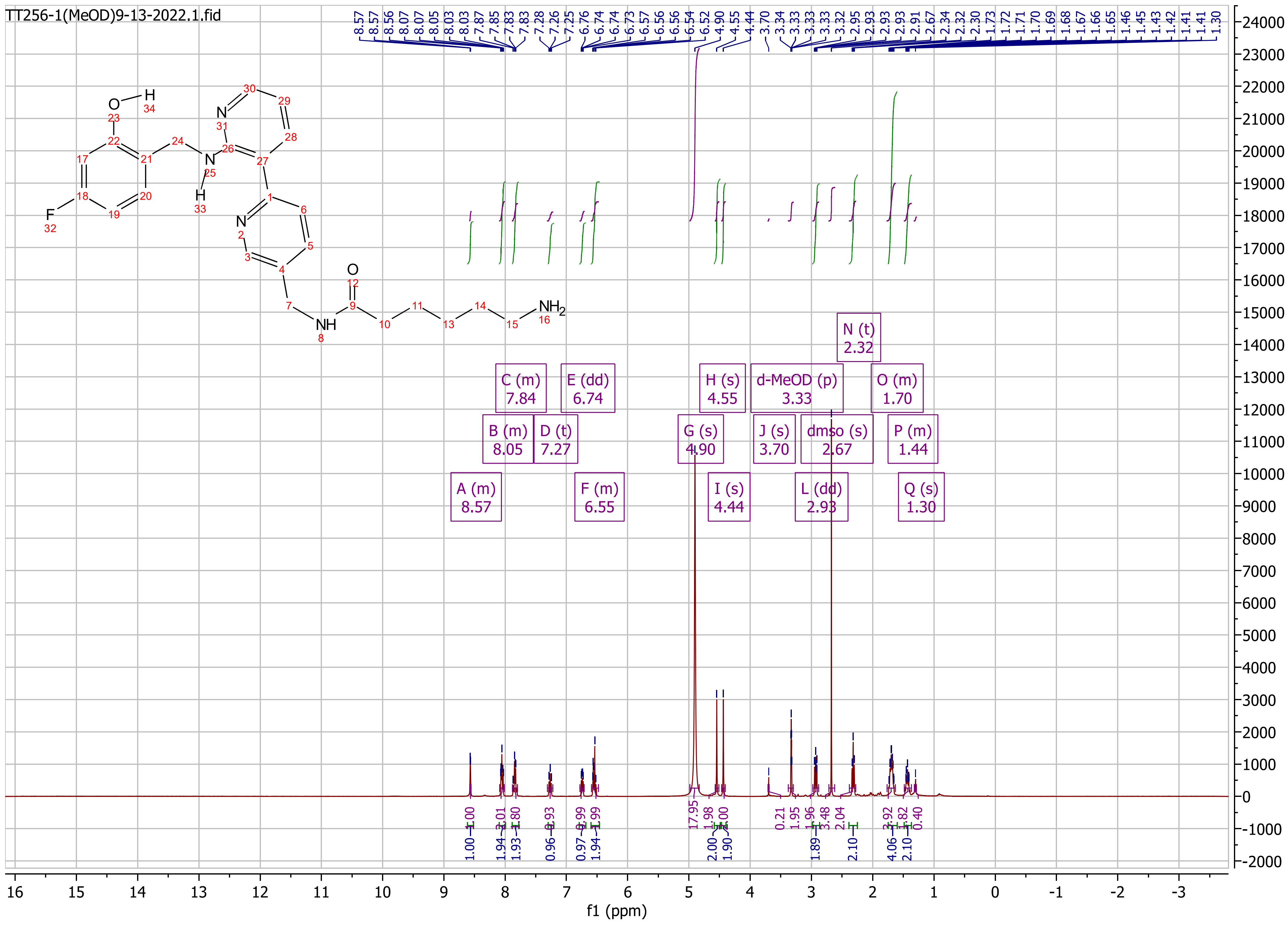
TT256-1(MeOD)9-13-2022.1.fid
A (m) 8.57
B (m) 8.05
C (m) 7.84
D (t) 7.27
E (dd) 6.74
F (m) 6.55
G (s) 4.90
H (s) 4.55
I (s) 4.44
J (s) 3.70
d-MeOD (p) 3.33
dmso (s) 2.67
L (dd) 2.93
N (t) 2.32
O (m) 1.70
P (m) 1.44
Q (s) 1.30
f1 (ppm)

TT256-1(MeOD)C.2.fid

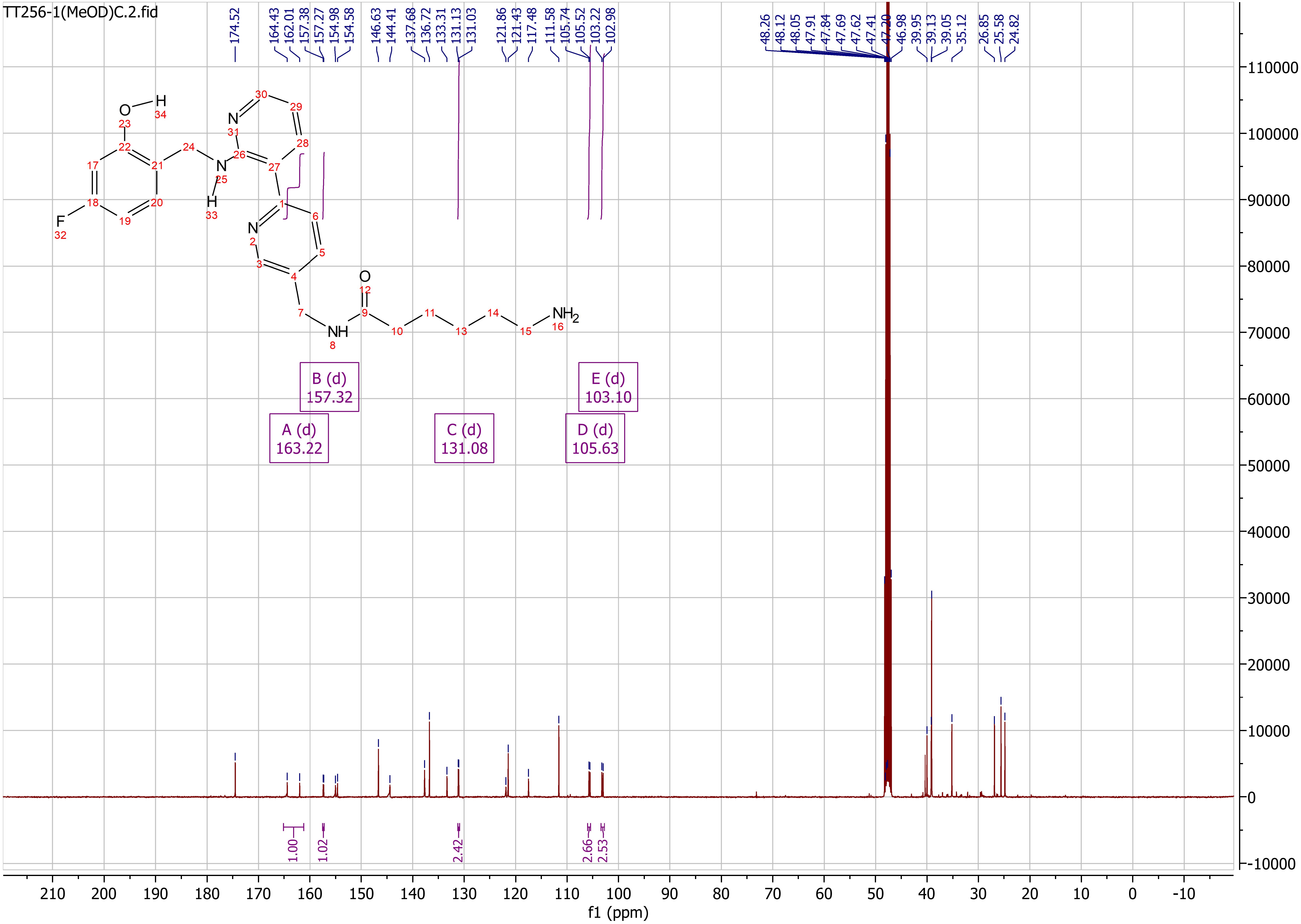

References

(1) Giannetti, A. M. From Experimental Design to Validated Hits a Comprehensive Walk-through of Fragment Lead Identification Using Surface Plasmon Resonance. *Meth. Enzymol.* **2011**, *493*, 169–218.
